# Discovery and Analyses of Caulimovirid-like Sequences in Upland Cotton (*Gossypium hirsutum*)

**DOI:** 10.3390/v15081643

**Published:** 2023-07-28

**Authors:** Nina Aboughanem-Sabanadzovic, Thomas W. Allen, James Frelichowski, Jodi Scheffler, Sead Sabanadzovic

**Affiliations:** 1 Institute for Genomics, Biocomputing and Biotechnology, Mississippi State University, 2 Research Park, Mailstop 9627, Mississippi, MS 39762, USA; nja62@msstate.edu; 2 Delta Research and Extension Center, Mississippi State University, 82 Stoneville Road, P.O. Box 197, Stoneville, MS 38776, USA; tallen@drec.msstate.edu; 3 USDA-ARS Plains Area, 2881 F&B Road, College Station, TX 77845, USA; james.frelichowski@usda.gov; 4 USDA-ARS Mid-South Area, 141 Experiment Station Road, Stoneville, MS 38776, USA; jodi.scheffler@usda.gov; 5 Department of Biochemistry, Molecular Biology, Entomology and Plant Pathology, Mississippi State University, 100 Twelve Lane, Mail Stop 9775, Mississippi, MS 39762, USA

**Keywords:** virus, pararetrovirus, cotton, *Caulimoviridae*, genome integration, episomal form, endogenous form

## Abstract

Analyses of Illumina-based high-throughput sequencing data generated during characterization of the cotton leafroll dwarf virus population in Mississippi (2020–2022) consistently yielded contigs varying in size (most frequently from 4 to 7 kb) with identical nucleotide content and sharing similarities with reverse transcriptases (RTases) encoded by extant plant pararetroviruses (family *Caulimoviridiae*). Initial data prompted an in-depth study involving molecular and bioinformatic approaches to characterize the nature and origins of these caulimovirid-like sequences. As a result, here, we report on endogenous viral elements (EVEs) related to extant members of the family *Caulimoviridae,* integrated into a genome of upland cotton (*Gossypium hirsutum*), for which we propose the provisional name “endogenous cotton pararetroviral elements” (eCPRVE). Our investigations pinpointed a ~15 kbp-long locus on the A04 chromosome consisting of head-to-head orientated tandem copies located on positive- and negative-sense DNA strands (eCPRVE+ and eCPRVE-). Sequences of the eCPRVE+ comprised nearly complete and slightly decayed genome information, including ORFs coding for the viral movement protein (MP), coat protein (CP), RTase, and transactivator/viroplasm protein (TA). Phylogenetic analyses of major viral proteins suggest that the eCPRVE+ may have been initially derived from a genome of a cognate virus belonging to a putative new genus within the family. Unexpectedly, an identical 15 kb-long locus composed of two eCPRVE copies was also detected in a newly recognized species *G. ekmanianum*, shedding some light on the relatively recent evolution within the cotton family.

## 1. Introduction

Endogenous viral elements (EVEs) are virus-derived sequences that are frequently detected in genomes of numerous eukaryotic organisms. The most studied and widespread EVEs originate from a class of RNA viruses called retroviruses that undergo a reverse transcription step and, as an indispensable part of a life cycle, stably integrate their DNA in the host nuclear genome (endogenous retroviral elements, ERVEs) [1]. The integration step is mediated by a retrovirus-encoded integrase. If this phenomenon affects germline cells, these EVEs eventually become fixed in a particular host species and are vertically transmitted throughout subsequent generations. Furthermore, after initial integration, EVEs may undergo amplification and become rearranged, distributed over the host genome, and often reprogrammed. As an example, it is commonly known that ~8% of the human genome is composed of more than 100,000 DNA pieces that were clearly derived from ancient retroviruses [2].

However, additional viruses, either with DNA or RNA genomes, may integrate in the host chromosomes, although in a much more serendipitous manner ([3,4,5], among others). In the plant world, the most frequently observed EVEs originate from the integration of a group of viruses called “plant pararetroviruses” [6,7,8].

Plant pararetroviruses are reverse-transcribing DNA viruses belonging to the family *Caulimoviridae* (order *Ortervirales*) [9], which currently comprises 11 genera: *Badnavirus*, *Caulimovirus*, *Cavemovirus*, *Dioscovirus*, *Petuvirus*, *Rosadnavirus*, *Ruflodivirus*, *Solendovirus*, *Soymovirus*, *Tungrovirus*, and *Vaccinivirus* [10]. Virions of caulimovirids (the general terminology for family members) are non-enveloped and either isometric or bacilliform in shape. They are comprised of a non-covalently closed circular dsDNA genome of approximately 6 to 9.8 kbp in size that, like retroviruses, replicate through RNA intermediates. Depending on the genus they belong to, caulimovirid genomes may comprise one to nine open reading frames (ORFs). Despite their diverse, genus-dependent genome organization, all caulimovirids code for several common proteins/domains—a movement protein (MP), a coat protein (CP), a pepsin-like aspartic protease (AP), and reverse transcriptase (RTase) with a bound RNase H1 (RH1) [10]. Additionally, the genome of members of several genera code for so-called transactivator/viroplasm protein (*syn.* inclusion body matrix protein). Unlike retroviruses, integration into a host genome is not an indispensable phase in a caulimovirid lifecycle. Nevertheless, their complete or partial genomic DNA can occasionally become captured by the host genome during a so-called “illegitimate recombination” phenomenon (non-homologous DNA end-joining) that can take place during the somatic DNA repair or meiotic recombination [11] and, as in the case of retroviruses, if integrated into the host germline it becomes stable and can be vertically inherited.

EVEs resembling currently known caulimovirids are widespread in vascular plants, both monocots and dicots ([12,13,14,15,16,17], among others), and were recently reported also in ferns, as well as in additional earlier-diverging terrestrial flora [18]. Recent advances in genomic sequencing, along with a growing field of public databases datamining, have contributed to a better understanding of the magnitude and distribution of this endogenization phenomenon in plants. The size, number, and distribution of the EVEs vary, so they may range from short and dispersed viral elements to longer insertions, sometimes representative of near-complete genome sequences. Most of these sequences are considered “genomic fossils” or “fingerprints” of past infections by ancient caulimovirids.

Indeed, an overwhelming majority of the thousands of currently known caulimovirid-like EVEs in a broad range of plants are replication-defective, due to accumulated mutations, fragmentation, and rearrangements. Therefore, these EVEs cannot induce infections in their hosts, but represent invaluable scientific material for understanding the diversity, distribution, and macroevolution of ancient and extant caulimovirids. Additionally, EVEs can be used as molecular markers to elucidate evolutionary histories of plants. However, in a few specific cases, such as in banana, petunia, and tobacco, endogenous sequences can be excised and activated under certain stress conditions (temperature, in vitro tissue culture, age) and reassembled to form virulent an episomal/exogenous form of the virus [8,19].

Cotton is an economically important crop worldwide and represents the largest source of natural textile fiber. The two allotetraploids, *Gossypium hirsutum* (upland cotton) and *G. barbadense* (i.e., Pima cotton), originated from an allopolyplodization event approximately 1–2 million years ago (mya) and account for 99% of the annual world output [20,21]. Upland cotton is cultivated worldwide because of its high-yield potential, while Pima cotton is known for its superior fiber quality. As with other agricultural crops, cotton is affected by various pathogens, including viruses, some of which may cause serious economic losses (for example, in the cases of begomoviruses in Pakistan and India or the cotton leafroll dwarf virus in South America) [22,23,24]. The evolution of cotton has been well studied and its phylogeny has been described through morphological traits and genomic evaluation. Currently, it is agreed that allopolyploid tetraploid cotton had a monophyletic origin [25,26] with the combination of two diploids, an ancestral “A genome”, and an ancestral “D genome” approximately 1.8–2.0 mya. After polyploidization, tetraploid *Gossypium* diverged into seven currently recognized tetraploid species [20,27,28,29]. The two tetraploid species, *G. ekmanianum* and *G. stephensii*, were previously classified as *G. hirsutum* until, very recently, they were approved as unique species [26,30]. Both the diploid and tetraploid species contain virus-derived endogenous elements with up to 50% of their genomes as retrotransposon elements, including Ty1/Copia retrotransposons and one class of Gypsy elements that has deranged sequences that could allow excision of the element [31,32].

As previously mentioned, numerous examples of EVEs have now been identified in plants. The evaluation of these EVEs has contributed to refining the evolutionary phylogenies of several plant species, including alfalfa, banana, and eggplant [15,33,34,35,36]. Given the wealth of endogenous viral fragments in cotton, it should be possible to evaluate the divergence of the tetraploid cotton species based on EVEs, and this will be explored, in part, in this study. Among the wealth of available current information related to EVEs, we focus on the in-depth, lab- and computer-based analyses of a specific caulimovirid-like sequences referred to as the “endogenous cotton pararetroviral element” (eCPRVE), which was discovered during analyses of high-throughput sequencing results that were obtained from cotton samples collected in Mississippi and a few neighboring states in the southeastern United States. 

## 2. Materials and Methods

### 2.1. Plant Materials

Depending on the experiment, the materials used in this study consisted of cotton tissue from true leaves, cotyledons, petioles, and young roots collected from a range of commercially available cotton cultivars and germplasm lines belonging to various *Gossypium* spp. A detailed list of the materials is presented in Tables S1 and S2 (Supplementary Materials).

### 2.2. PCR Detection and Survey

Total DNA was extracted and purified from foliar or root tissue from 59 field or chamber-grown cotton samples using the Fungal and Plant DNA extraction kit (Norgen Biotek Corp., Thorold, ON, Canada), following the protocol recommended by the manufacturer. After elution, an aliquot was submitted for PCR with primers eCPRV2085F 5′AAATGGTTCTTAATTGGGCTAGGTTGTA3′ and eCPRV2830R 5′TCCTTAATATCCTGACCATCTTCAAGAGT3′ designed on sequences of the initial contig. For detection, amplification was performed with Phire Hot Start II DNA Polymerase (ThermoFischer Scientific, Witham, MA, USA). PCR amplification cycling conditions were as follows: Initial denaturation at 98 °C for 15s, followed by 35 cycles 98 °C for 5 s, 61°C for 5′, and 68 °C for 15 s, with final extension at 68 °C for 15s. Simultaneously, each sample was also submitted to PCR with NADH dehydrogenase subunit 5-specific primers Nad5-480F 5′TTGGTCACCCGATGCTATGGAG3′ and Nad5-820R 5′ACATGGCATGAATCACCGAACCT3′ designed in this study, under the same cycling conditions, to verify the quality/quantity of extracted DNAs. PCR products were analyzed in 1.5% TAE (Tris-Acetate-EDTA) agarose gel electrophoresis and the results were visualized by GelRed staining and exposure to UV lights. The results were documented with a GelDoc system (Bio-Rad, Hercules, CA, USA).

### 2.3. High Throughput Sequencing and Sequence Analyses

Total nucleic acids were extracted from 0.1 g of cotton using the Sigma Spectrum Plant Total RNA extraction kit (Sigma–Aldrich, St Louis, MO, USA), following the “protocol A” option (lysate-to-binding solution ratio 1:1), as described by the manufacturer. The integrity and quantity of preparations were determined in agarose electrophoresis and/or by spectrophotometric reading with a Qubit 3.0 fluorometer (Invitrogen, Waltham, IL, USA) prior to ribodepletion, cDNA library preparation, and custom-based, 2 × 150 pair-end sequencing performed on an Illumina HiSeq3000 instrument at the Roy J Carver Biotechnology Center (University of Illinois, Urbana-Champaign, IL, USA). The raw sequencing output, approximately 40–50 million reads/sample, was initially filtered for the quality and assembled de novo by SPAdes v.3.15.5 [37]. Hundreds of thousands of contigs per sample, longer than 1 kb, were compared with sequences that were publicly available in GenBank using “cloud”-based BLASTx searches [38]. A total of 21 field-collected upland cotton samples, along with three different *Gossypium* sp. samples (*G. raimondii*, *G. sturtianum*, and *G. darwinii*), were submitted to HTS and analyzed for caulimovirid-like sequences.

### 2.4. EVE Sequence Analyses

Contigs matching pararetroviral RTases were analyzed for the presence of open reading frames with Geneious Prime 2019.2.3. Amino-acid sequences of caulimovirid RTase, MP and CP were retrieved from the NCBI/GenBank and aligned with corresponding putative products of eCPRVE using MAFFT v. 7.407 [39].

In the case of the RTase and MP datasets, ambiguous and/or poorly aligned regions were removed from the final alignments by TrimAI v.1.4.1 [40]. Best-fit substitution models were determined for each aligned dataset, according to the Bayesian information criterion determined by ModelFinder [41]. For datasets comprising RTase, MP, and CP sequences, the best-fitting models were LG+F+I+G, LG+F+G, and VT+F+I+G, respectively. The maximum likelihood phylogenies for the three datasets were inferred by IQ-Tree v. 1.16.12 [42], with ultrafast bootstrapping performed by UFBoot2 [43] and visualized with iTOL v5 [44].

### 2.5. In Silico Mining for eCPRVE and Related EVEs

Searches for eCPRVE sequences were performed as follows: (1) a BLASTn search against *Gossypium* spp. genome database available at the CottonGen cotton database resource (https://www.cottongen.org [45], most recently accessed on 1 June 2023), by using original 6.1 kb EVE sequence as a query, and (2) various general and organism-specific BLAST searches in the GenBank/NCBI of the putative proteins encoded by the eCPRVE.

## 3. Results

### 3.1. Original Discovery and Analyses of a Caulimovirid-like Sequences from Cotton

An original contig—referred to as DEC02-76, of 6091 nt in size (Figure S1) and a mean coverage of 77x—was obtained in September of 2021, while analyzing HTS data of an ongoing investigation on cotton leafroll dwarf virus (CLRDV) in Mississippi [46,47]. When compared with the protein sequences available in GenBank through BLASTx searches, the sequences of RTases encoded by figwort mosaic virus (FwMV) and several additional recognized or putative members of the family *Caulimoviridae* resulted as top matches.

Computer-assisted sequence analyses of the contig suggested the presence of several putative ORFs with the arrangement reminiscent of a near-complete and slightly decayed/deranged caulimovirid genome (Figure S2). Indeed, the sequence started with a Met-tRNA primer binding site that was previously reported as characteristic for genomes of all extant family *Caulimoviridae* members. Putative products of the two 5′ proximal ORFs, with estimated molecular masses of 14.4 and 24.4 kDa, respectively, did not have any statistically supported hits when blasted against the reference protein database. A protein encoded by the ORF3 (~41K) showed significant similarity with the N-terminal portion of movement proteins of several caulimovirids—in particular, with the blueberry red ringspot virus (BRRV, genus *Soymovirus*), as well as with the Rudbeckia flower distortion virus (RuFDV, genus *Ruflodivirus*), sharing 40–44% identical amino acid sequences over 60–66% of the entire protein. The small 13K polypeptide, putatively encoded by the ORF4, did not exhibit any similarity with any previously reported proteins. Products of two in-frame ORFs (denoted as 5a and 5b in Figure S2) matched amino-acid sequences of coat proteins encoded by the members of the family *Caulimoviridae*, indicating that, unlike functional caulimovirid genomes, this cistron is disrupted by a stop codon in the case of contig DEC02-76. Finally, in silico translated products of the three 3′ proximal ORFs (two in-frame, separated by a single stop codon, and a third shifted to a different frame due to indels) contained signature motifs and shared identity with viral reverse transcriptases (Figure S1). Presence of unexpected stop codons, frameshifting, and additional mutations instead of contiguous ORFs suggested that contig DEC02-76 derives from a partial genome sequence of a putative pararetrovirus integrated in the host genome, referred to as the “endogenous cotton pararetroviral element” (eCPRVE), rather than from a genome of an episomal, replication-competitive form of the virus.

**Figure 1:**
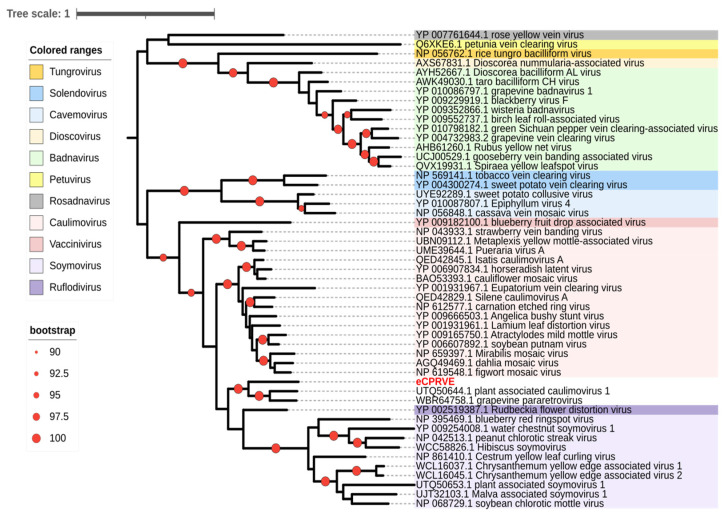
Maximum likelihood phylogenetic tree reconstructed with IQtree [42] from the amino acid alignments of reverse-transcriptases encoded by genomes of recognized and putative members of the family *Caulimoviridae* and manually adjusted sequences of the endogenous cotton pararetroviral element—eCPRVE (red font). The representative tree was visualized with iTOL v5 [44]. Different colors denote the 11 currently recognized genera in the family. Clades with statistical support of >90% are indicated by a red circle.

Phylogenetic analyses of manually refined protein sequences of the viral RTase, CP, and MP placed them in the extant family *Caulimoviridae*. All three putative proteins grouped with orthologs encoded by a few unclassified members of the family, plant-associated caulimovirus 1 and grapevine pararetrovirus, forming a highly supported clade that is related to, but distinct from, those corresponding to the 11 current genera in the family (Figure 1 and Figure S3). Accordingly, based on the results of phylogenetic analyses and pairwise comparison with orthologs encoded by the recognized caulimovirids, the creation of a novel taxon within the family may be warranted to classify the putative cognate virus and to fully reflect the currently known diversity of these viruses.

### 3.2. Additional Lab- and Computer-Based Studies to Reveal the Nature of Caulimovirid-like Sequences

**Figure 2:**
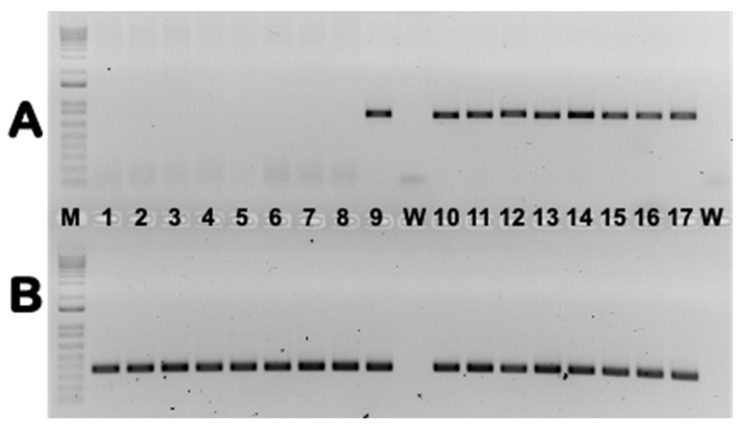
PCR results in 1.5% TAE agarose gels visualized by GelRed. The eCPRVE-specific primers used in reactions are presented in the upper gel portion (**A**), while the PCR results obtained with Nad5-specific primers from the same samples are presented in the lower part (**B**). Lanes 1–8: various non-upland cotton samples; lanes 10–16: diverse upland cotton genotypes; lanes 9 and 17: positive control (cultivar PhytoGen 490 W3FE); lane W: water control; lane M: 1 kb Plus DNA Ladder.

Additional experiments were designed to verify whether contig DEC02-76 contains sequences of an EVE in upland cotton, as suggested by observation of ORF interruptions and frameshifts during the initial analyses. In PCR experiments carried out on 59 samples from 10 different *Gossypium* genotypes/species, a distinct band of an expected size of ~780 bp was consistently generated from samples of upland cotton (*G. hirsutum*) (Figure 2 and Table S1). In addition to modern, currently grown cultivars of upland cotton that are commercially available in the southeastern United States, such as Deltapine 2115 B3XF, PhytoGen 443 W3FE, and Armor 9371 B3XF, we included some old cultivars that were popular in the United States in the late 19th century or the beginning of the 20th century (i.e., “Kekchi”, “Lone Star”, “Dixie Triumph”, and “Macha”), as well as cultivars originally collected from various parts of the world, including Brazil, Uzbekistan, Australia, and several African countries.

While most PCR assays were performed on DNAs extracted from foliar tissue collected from field-grown plants, several samples were tested as young seedlings grown in isolation under artificial conditions in a growth chamber, or as young roots. Independent of cultivar, geographic origin, or tissue used, all tested upland cotton (*G. hirsutum*) samples tested positive, indicating the ubiquitous presence of caulimovirid-like sequences. No visible bands were observed in PCR products from any of the “other-than-upland cotton” tested, except for the sample of *G. barbadense* “Sea Island”. Curiously, none of the 14 additional *G. barbadense* accessions assayed in this work generated a PCR product (Table S1).

Additionally, we performed custom-based HTS on the total RNAs extracted from 21 field samples collected in Mississippi (18 samples), Louisiana (one sample), Tennessee (one sample), and Arkansas (one sample). A majority, but not all, of these samples were co-infected with CLRDV. Assembly and analyses of HTS data revealed at least one contig with a top hit with RTase of FwMV in each library. Interestingly, the coverage (the average number of reads per base) of these contigs was rather uniform among the different sample libraries and ranged from 35x to 89x, indicating rather stable concentration of the templates independent of the host genotype, the time of tissue collection, the type of the tissue, and additional variables. Therefore, a total of 21 caulimovirid-like contigs were obtained. They greatly varied in size, from 3802 to 13,742 bp (Figure S4), but had mutually conserved nucleotide sequences (99.8–100%) and the number/organization of ORFs, indicating their origin from a sole template. Analyses of the longest contig, 13.7 kb-long, revealed that the 7 kb-long portion of the sequences located upstream of the EVE sequences derived from the cotton genome characterized by low A/T content (32%) and coding capacity for products sharing similarities with RTases of bacterial and plant Ty1/Copia retrotransposons. No contigs similar to DEC02-76 sequences were identified in assembled data of Illumina sequencing for any of the three non-upland cottons.

### 3.3. In Silico Search of Cotton Genomes and Analyses of the eCPRVE

The next goal of this study was to pinpoint the place of insertion/integration and the number of DEC02-67-like EVEs inserted in the genome of *G. hirsutum*, and possibly in additional cotton genomes. For this purpose, genome sequences from a total of 55 cotton genotypes/cultivars belonging to 27 species in the genus *Gossypium* were screened for the presence of EVEs, using our DEC02-76 contig as a query.

**Figure 3:**
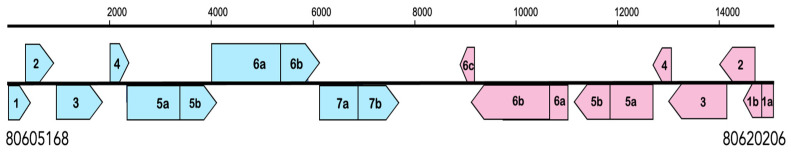
Schematic representation of a tandem insertion of contig DEC02-76 sequences into chromosome A04 of the *Gossypium hirsutum* nuclear genome. Numbers below the figure indicate positions of extreme 5′ and 3′ terminal nucleotides on the A04 chromosome of the *G. hirsutum* TM1 UTX v2 genome used as a reference. Notice the slightly different organization between the two EVE copies and the presence of ORFs 7a and 7b in the sense copy (eCPRVE+).

Analyses of upland cotton genomes available in the CottonGen database [45] revealed the presence of tandem, full-size copies of DEC02-76-like eCPRVEs integrated in the ~15 kb-long locus in the A04 chromosome (nucleotide position 80,605,168–80,620,206 in the TM-1 UTX v2 genome used as a reference). The two copies are present on different DNA strands: a sense copy (eCPRVE+) and an antisense copy (eCPRVE−), and are oriented head-to-head (Figure 3). Mutual sequence identity between the two eCPRVE copies was 98.98%, resulting in slightly different organization. Alignment of the two eCPRVE copies/variants with our HTS-generated data indicated that all 21 contigs originated exclusively from a negative-sense copy (eCPRVE−).

However, in addition to six ORFs observed during analyses of the contig DEC02-76 (corresponding to the antisense EVE copy), the positive-sense copy contained a pair of additional large in-frame ORFs (ORFs 7a and 7b, Figure 3) separated by a single stop codon, coding for N- and C-terminal halves of the transactivator/viroplasm-like (TA) protein characteristic for members of several genera in the *Caulimoviridae*, along with two small 3′ proximal ORFs coding for peptides with an unknown function (Figure 3 and unpublished data). This tandem insertion was present in all analyzed genomes of upland cotton with available data in the CottonGen database (Table S2).

In addition to chromosome A04, another eCPRVE was revealed on chromosome D03 of the TM1 UTX v2 genome data and all additional upland cotton genomes analyzed in this study (a total of 11, as shown in Table S2). The length of this insert was 3960 nt and it shared 84% nt sequences with the two eCRPVE copies present on chromosome A04.

**Figure 4:**
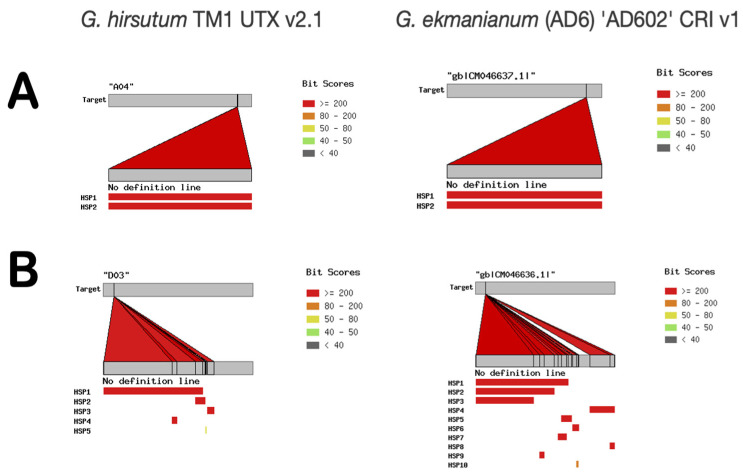
Schematic representation of BLAST+ search results of the representative genomes of *Gossypium hirsutum* and *G. ekmanianum* for endogenous cotton pararetroviral elements. Panels (**A**,**B**) contain results of BLAST+ analyses of chromosomes A04 and D03, respectively. Red bars represent genome sequences with high similarity with the query. The analysis was performed in the CottonGen database (cottongen.org) with contig DEC02-76 as a query.

Curiously, a conserved ~15 kb locus with a tandem insertion (double red bars, as shown in Figure 4A), virtually identical to the one in upland cotton, was also identified in the genome of a newly recognized species *G. ekmanianum*. The two loci on chromosome A04 in upland cotton and *G. ekmanianum* differed in only 40 SNPs over the 15 kb length and shared 99.73% identical sequences. No genomes of any of the other 25 related cotton species analyzed in this work contained similar loci.

However, noticeable differences between the two closely related *Gossypium* spp. were detected during the analyses of eCPRVEs located on the D03 chromosome (Figure 4B). Instead of a single ~4 kb EVE, as was the case in upland cotton, this specific chromosome in *G. ekmanianum* harbored a triplet of eCPRVE-like EVEs (Figure 4B). These three copies of 3987, 3393, and 2499 nt were homologous to ~4 kb insert in the corresponding chromosome in *G. hirsutum*. These loci contain interrupted and incomplete ORFs for putative caulimovirid MPs and CPs. Finally, *G. ekmanianum* apparently contains another small eCPRVE of ~2 kb on chromosome A12 that was not observed in *G. hirsutum* (not presented).

A few EVEs of ~1 to ~2.3 kb in size with 75–80% identical nt content with the original eCRPVE were observed in genomes of two additional tetraploid cotton species (*G. tomentosum* and *G. mustelinum*), in addition to several, more decayed EVEs with origins from caulimovirid-like genes coding for MP, RTase, or CP. However, no larger insertions with (near) identical sequences with eCPRVEs were detected in the additional analyzed cotton species/genotypes. Finally, BLAST results of diploid cotton genomes suggested lack of any loci with significant homology with the studied DEC02-76-like eCPRVE.

## 4. Discussion and Conclusions

In this study, we detected and characterized ~6.1 kb contig in HTS-generated data from a cotton field sample coinfected with CLRDV. Despite its close resemblance to near-complete genomes of extant viruses belonging to the family *Caulimoviridae*, the presence of interruptions in ORFs coding for three major putative viral proteins (movement and coat proteins and reverse transcriptase), and other evidence gathered in this work, made us suspect that this particular contig derives from an integrated form of incomplete pararetroviral genome sequences, referred to here as “endogenous cotton pararetroviral elements” (eCPRVEs). The initial suspicion was further supported by the presence of contigs with identical nucleotide content in all 21 sequenced upland cotton libraries, of which some were longer than the original 6.1 kb contig. Analysis of a few of the longest contigs revealed the presence of host chromosome sequences attached upstream to the sequences of eCPRVEs. Consequently, analyses of all 21 contigs showed conserved disruptions (single stop codon in each of the two ORFs coding for putative viral CP and RTase proteins) and a frameshift in the 3′ proximal portion of the RTase-coding cistron caused by an indel. Furthermore, analyses of the Illumina-based data from the three additional different *Gossypium* spp. did not reveal any contigs with features comparable to the ones generated from *G. hirsutum,* indicating its association only with upland cotton.

Indeed, results of PCR experiments performed on DNA extracted from 59 samples belonging to 10 different *Gossypium* species confirmed that the studied eCRPVE is mainly present in *G. hirsutum* germplasm, where it in fact appears to be universally present in upland cotton cultivars, either those cultivated more than a hundred years ago or in modern (currently grown, commercially available) cotton production lines, as well as wild cotton germplasm and landraces. The only exception was a positive result of a single sample of “Sea Island”, reportedly belonging to *G. barbadense*. However, it is known that there are many versions of this particular cultivar that have been saved, since it was a widely grown cultivar in the early 1800s and there are numerous reports that it has significant *G. hirsutum* introgressed into its genome [48], which may explain the unexpected results and calls for testing of additional sources of “Sea Island”.

In silico mining of the genomes of 55 *Gossypium* and *Gossypoides* spp. (22 tetraploid and 33 diploids) revealed that sequences of the original contig DEC02-76 originated from a ~15 kb-long locus on the A04 chromosome in all 12 analyzed *G. hirsutum* genomic datasets. The locus is comprised of two head-to-head oriented copies with nearly identical nt content, located on positive and negative DNA strands. Nevertheless, a copy located on a positive strand DNA (eCPRVE+) provided a further hint about complete genome organization of a potential cognate virus, including the presence of an additional interrupted ORF (Figure 3, magenta) coding for transactivator/viroplasm associated protein (TA, a.k.a. inclusion body matrix protein), a multifunctional protein known to be a major component of electron-dense inclusion bodies associated with active infections of numerous family members. Accordingly, the putative cognate virus genome might also comprise this ORF. 

The locus was delimited on 5′ and 3′ by the presence of conserved nucleotide motifs of the Met-tRNA primer binding site. Curiously, a mirror-copy of the locus originally discovered in genomes of upland cotton was also present in the A04 chromosome of one additional species, *G. ekmanianum*. *G. ekmanianum* is a recently recognized new species originally from the Dominican Republic and is the closest known relative to upland cotton. None of the 33 diploid cotton genomes had statistically significant hits indicating the absence of eCRPVE-like integrants.

The presence of distinct and unique EVEs can be used as a molecular marker to infer evolutionary histories between the cognate virus and the host. Indeed, the eCPRV discovered and characterized in this work is a good example. Previous studies have estimated that hybridization of “A” and “D” genomes occurred approximately 1–2 mya [27], followed by the initial divergence between allopolyploids beginning approximately 1.8 mya and their separation into two major clades: one including Pima cotton along with *G. darwinii*, while the other clade comprises upland cotton along with two new species *G. ekmanianum* and *G. stephensii*. A recent study has estimated divergence of these three sister species from the most recent common ancestor approximately 0.75 mya [49].

**Figure 5:**
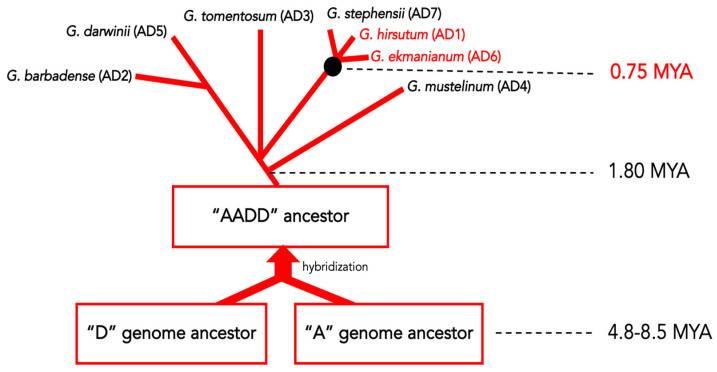
Schematic representation of the evolutionary history of tetraploid *Gossypium* spp. with an estimated timeline. The possible original eCPRVE integration event is depicted by the black circle. The two species containing identical 15 kb loci with tandem copies of the eCPRVE are reported in red font.

Therefore, taking into account the presence of an identical ~15 kb-long insert composed of tandem repeat of eCPRVEs in the same position on chromosome A04, solely in the genomes of two closely related and relatively recently diverged species—*G. hirsutum* and *G. ekmanianum*—and not in any of additional *Gossypium* tetraploids that were analyzed, it is plausible to hypothesize that these EVEs originated from a single integration/endogenization of a cognate pararetrovirus into the genome of their last common ancestor, rather than from two separate and independent events. Furthermore, a high level of conservation between eCPRVE copies from *G. hirsutum* and *G. ekmanianum* suggest an integration immediately predating the speciation event in the *G. hirsutum* clade, estimated at 0.75 mya (Figure 5). Nevertheless, it is assumed that the degree of sequence degradation/preservation directly correlates to the approximate age of integration. Therefore, a different scenario involving multiple independent and more recent integration events cannot be excluded, due to the unusually low degree of genetic decay observed for studied eCPRVEs in these two cotton species. Similar studies on eCPRVEs in *G. stephensii*, another recently described sister species to upland cotton from Wake Atoll [30], are indispensable to better understand the relatively recent evolution of cotton. Unfortunately, there was no such genomic data available at the time of this study.

The impact of EVEs on the host may be diverse. The integration of EVEs into or near host genes may interfere with transcription and function, resulting in an altered plant phenotype, or an even more detrimental outcome. EVEs that result in these types of responses will eventually be rejected from the host population by forces of purifying selection. Most of the previously reported EVEs are thought to be neutral and eventually decay over time, due to the accumulation of mutations and indels, resulting in the ultimate disruption and fragmentation. As a result, most of the previously reported EVEs have, in fact, been rendered inactive within host plant material. However, there is growing scientific evidence suggesting that EVEs that are not rejected after integration confer an evolutionary advantage to their hosts. Indeed, it is known that several EVEs in animal genomes have been co-opted as cellular genes [50]. Accordingly, there is evidence that EVEs in plants can contribute to resistance to their cognate viruses by RNAi or additional mechanisms [17,51,52,53]. For example, EVE segments reported in the putative gene Cg1g024630 of pummelo have been reported to be related to the CTV resistance gene locus in trifoliate orange (*Poncirus trifoliata*. We are currently evaluating whether this is the case for the eCPRVEs reported in this study.

At this point, it is unclear whether the 15 kb-long tandem insert of caulimovirid-like sequences reported in this study is transcriptionally active or not, and whether any of the putative ORFs are expressed in planta. Understanding the relationships of eCPRVE with its host, along with a comprehensive search for the episomal/infectious form of a putative cognate virus, is part of an ongoing study.

## Data Availability

The data presented in this study are openly available in GenBank. Accession number OR269951 was assigned to original sequences DEC02-76 deriving from BioProject PRJNA990948. Nucleotide sequences of other 15 contigs were deposited under Accession Numbers OR269936-OR26950.

## Supplementary Materials

The following supporting information can be downloaded at: https://www.mdpi.com/article/10.3390/v15081643/s1, Figure S1: nucleotide sequences of the original contig DEC02-76 (for peer-review), Figure S2: Organization of the contig DEC02-76 compared to a putative functional cognate caulimovirid genome, Figure S3: Maximum likelihood tree reconstructed on amino acid alignments of movement and coat proteins; Table S1: List of cotton germplasm tested in PCR with eCPRVE-specific primers, Table S2: List of *Gossypium* and *Gossypoides* spp. screened for the presence of eCPRVE sequences, Table S3: Percentage (%) nt identity among 21 eCPRVE contigs generated in this work by HTS. Figure S4: Clustal Omega alignment of 21 caulimovirid-like contigs generated with HTS.
