# Supplementary material for: Discovery and Analyses of Caulimovirid-like Sequences in Upland Cotton (Gossypium hirsutum)
Source: Viruses. 2023 Jul 28;15(8):1643. doi: 10.3390/v15081643 (PMC10458927; doi:10.3390/v15081643)
Supplement: Supplementary file 1 [file viruses-15-01643-s001.zip › viruses-2480933-supplementary.pdf]

# Discovery and analyses of caulimovirid-like sequences in upland cotton (*Gossypium hirsutum*)

**Nina Aboughanem-Sabanadzovic [1], Thomas W. Allen [2], James Frelichowski[3], Jodi Scheffler [4] and Sead Sabanad-zovic**

## Supplementary Materials

**Figure S1:** Nucleotide sequences of the original contig "DEC02-76"

**>DEC02-76_6091nt**

TGGTATCAGAGTAAATCACGATCTCAGGACAAATAAAATGGATGAAAATCCTGAATATAAAGGATATAAGGATGTAAAAGAAATCAAAGAACAATT
AAGCCTAATAATGATTCAAATAAACAGGACTCAAGAGCAAATAATAAAGCTCCAAGAGACTGTTAATCTGATAGCAACAGGAACACTGATGCTTTC
AGAAGAACAATCTCAAAAAACTGAGGAAAACTTTAAACAATTAAAAGATTTAATTGAACCTATTAAATCAGGATATTTATTAATTGAAAAATTAAT
AAAAGACTCAAGACCACAAATACAGAGGAGGTATGAGCAATTGAAACAATTTACAAAATGGTTCTTAATTGGGCTAGGTTGTATATTGATCTTTGC
CATCCAAAGAAGCTTGAATTGAGTATTCAGTTTAAGCCAAAAAATGAATACAGTAAATGCTTTAACAAGTATCTACAAGGATTACATTACTGGTTT
AATAAAAACCAGAAAATACCATTAATAATTCCAGGATTCAGCTATAGTTATAATTTCCAACATCATGGTGGAAATCTTAACTACAAATATAATTTC
AATTATAAAATGAAACAACAGGAAATGGATTTCAAACAATTGAATCCACTCGCTAATGAAATTCAGGAACTATTAAGACAAGGAATTATTAATAAT
GAACAATTAACAGAAGCTTTAAGATATTTAAAATATCTTTCAGAGCACACTAATGAAAGACAAAAATTAAGCTATGATGAATTGAATAAACTATTC
AACATATTTGGAGAAAGAATTGATAAAAGTATCAAAGATTTCAAGGAAGAAGTTGAAAGACAGTTTAAAAATTTAACTCAAGATAAAAAAGATATT
CAAGAAATAAAAGAATTATTAAAAAATAATAATCAGGATCTTGAAGAAATAAAGAAAAGACTTTCAGCAGTAGAAAAGAAATTATGAGTACTAATT
CAGAAATTAGTACAAACATAGGAAATAATCTCGATAACGAGGTTATAATCAGGAATATTGATGACAATGAAACCTATCAAAGAGAAATATTAGTTA
ACGCTAATAAATTATCTAAGATAGAGAAAAAAGGAAAGATAACTCTTGAAGATGGTCAGGATATTAAGGATAATCTAAGTCAACAATTCAAAAGAT
TGTTTGACAGAGAAAACATACTATTTTTAGGAAAATTTGTAGAGGAACTTCCTATACAAATTAAGCAAGCTACAGGAAACACTGTGTTACCTTTCA
TAACACAGCAAAGTTTAGAGGAAAAATTATCAAAGATACCTAAAAAGGATAGGACCAAAATAAAATATATACATTTTGGAAAAGCACAAATAATAA
TAAAACCAACGATAAAATCAGGAATAGACACACCCATTGAAATAATAGTGTATGACAGGAGAATAACGAGTAATAATATTAATGAAATAATAATAG
GACGACTCGAAGGAAACTTAGGATATCCAGCAGTAAAATTTGATGTTAGCCTACAAATAGGAATTCCAATAATATCAAATTATCTAGGAAAGTCTA
TAGGATTAAGTTTCAGATACTTAAGACAAGACTTAATGAATCAGGATGATTACCCATTCAGCATTTTATATGCTGTAGGATATGGATTGAGTAATT
CGCATTACTCAGTTAATTTCAAGACCAGCAACAAGATAGAAATTGAACAAATATTTGCAGAAACAAGTACTAAGCTAATAGAAATCCCCAGAAGTC
ATTTTAGTTTACCTAAATTAACGAATGGAGACAGGATTGAGGAAACTTCTGAAAATATTTTCAGAAATTATAATGATAGCACTGCACTTGTGCCAA
AACAAGGATTTAGCTTAAATAGGAGTCAAAGTCAAAGCTTTAGACTACCTAGACAGGAGTATGAAATACCAGTAGAGCACCCTTCTAGTATTCCTA
TAATATTAGAAGAACCTACTAATATTCATACAGTAATAAACACACAAAAGGATTTAAATGAAGTTAAAGAAATGATAAGAAAAATTGATAATAAAT
TATGAGCAGGTCAGAATTTGAATTCAGACCAGAGGAAAGCATTTCACAATATCTCTCTAGGAATTTACCAAAACTCCTTCAAGATAATAGTGAAAT
AGCAGAAAATCTAAAAGGATTAACAATAATAATAAATAAACTTACAGAAAATCAAGAAAAATATGATGAAAATATGAAAAGATTTGAGGACTTATT
AAGAGAAATAGATAGGAAGATTACTAATCTACCATCTAGTTCATCACCTAAGAAGAAGACAATTAAGTGGACCAAACCCGATCCACCATTCCCAAT
ACCTACTAAGATACCACCTCTACCAGATAAAAAACCACAATAGAATCTGTTAGAAGACAAGAAGAATTAGGAGAAAGGATTAGGAACTGTAAAAAT
CAAAGAGAATTAGATTCTTTAATAGAGGAAACCTGTAATATGGAATTTGGTTTTCAAATCCAGGATGACATCGAAGAATATATATTCGACGATGAA
GATTATGAGGATGAATATGATCATGATGAATTAATAAAAATGGAAAAAGATAAAATAAAGATTGAAGAAATATCAGATGATGAAAAATACTTAGAT
GCCCCTCTAAGTAAAACTGAACAAGGAGAATCTAGTAACAGTAAAGTTAAATATGAAGATGAAGAAGGAATTAACTTAAAAGATGAGGAATTTAGT
GATGATGAAGTAAATGAATATTTCGACCCTTCATTAGATCCACTAAAAGATGGAATAGAAAACATTGATAATGATGGATTAAGCAGATTAGAAACT
AGGAAATCTAGTAAAAGAAGAGGAACTTCAGGATACTATCATTCTTATGATAGTAAATACAAGACTAAAATACCCCCACAATACCAGCTAAGCCAT
AGCGAAAATGCGTATAATAATAGGTGGTTAAACCTAGATTGTACGTTAGATAAAACCAATGAATTAGACGGTTGGTACCGTCAAATGAGCTTTTTA
AGCTTAAGGAACATAGAAACAGTAGCAGACCTAGAACCGTTCTTGGAACATTTCATGACTGGAAATGTACGAGCATGGTGGAATTCTGAAAGAGGA
GAAGCATTAAAATCTACTTATCTAACTTCAGATGACACAATACAAACTAGATTAACGAAGATTAAAGCCTTAATTTCTAACGAATTTATAGGTTTA
AATCAAATTAATCTAAAAGCAGTTTAGGAAAAAGAATCAGAGAAAGCAGAATATATTCTCAACAATATCAAGATCTGTGATCTATGTTATTGGAAG
AATTTTGTCTGTGAGTATGAAAAATGGTTCTACAAATTAGGAAAACGAGAAAAGATGGATCAATATGAAAAGCAATTCTATCTCAAGTTTCCACCA
ACATGGAAAGATGAGTTAGAAAAAATATTTGAAACAGAAAAAGATGCAAGATTAACCAATAATTTGGCAGGAAGAGTACAATGTTTACAGGAATTA
ATGGAACAAAAATGTAAAGAAAAATATTTTAATGAGGATGTAACAAGATTCACTAATAAGAAACCCTGTTGTGATCCTTCGCTAATCGACATTCCT
ACTAGATGGGGATGTAGACCGCAAAAGGATTATAGGAAATCTTATAAGACTTATAAGAAAAAATACAGGAGATATAGGAAGAGGTTTTCACCTCGA
AAATATAAAAAATATAAAGGCAATACAGGAAGAAAAAAGGATTTAGAAAGAAAGGACGCCATAAAAGGAAAAATAGTTATGATGAGCTTCGCGAT
AACAATTATCGTGATAGTAATAAAAAACAGCAGGATTGTCCCAGAAAGAAAAAGGATTGCAGATGTTGGCTCTGCAAAGAGGAAGGACATTATGCG
AATGAATGTCCACAAAGATATAAAAAAGAGAATAATAAAATAATAAGACAGTTAGAATACATAAACAGTATATGATATGAACCAGTAGAGTCAGAC
CCCGACTCAGATGAATATGTGTATGAATATGACACAGAAGAATAAATAAGAGAGTTTTCTGATGATCCCGGATCAGAATGGGAACTCATAGATTAA
GACTCGATAAAATTTATCTAGAACAATATCAGGATTATGAATATAATATAACAAATCCTAATAGCACCTACATCAAAGTAGGATTAAAATTCAGAG
GATTTAGGTATTATCATTTACATGCCTATATAGATACTGGAGCCAGTATTTGTGTAGCTCATAAGGATGTAATACCAGCAGAAAAATGGGAGGATA
CGCTGTTTGACATTAATGTTCAAATAGCTGATAAGAGTATAATCAAGATTAATAAAGTTGCGAGAGATATCTATCTCGAGTTGCAAGGAACGTTGT
TTAAGGTCCAAACCTTATATCAACAAAACACAGGAATGGATATTTTAATATGAAATAATTTCTTACATTTATATCATCCTTTTGTTCAACATTTGG
ATTATATAACATTAAGAAATAATAACAGGACTATTAAAATCCCTAAAGTTAGGAAAGCCTACAGTTGGCATCGACCCGACTTCCTATCAAAGTTAC
GCATACCGCGAAAGCGTGGTGGGAATAGGAAAAATGAAAATTCAAAAATAAAACAGGATGACTTACCAAAAGTCAATACTACGCCTAATAAAGAAA
ATGAGGAGTCACAGGAAAATTTAAATCTAGAACAAATGTTCATGATTGAGGATAAAGTCAGACAAATATTAAAAGAAATAAGTTCATTGAATCCAC
TAGATTCAAGGATAACGTGTTGTAATCTTTTTAAAAGATCACGACTCGAAGCAGAGATAAAACTAAAAGATGAAAAGAAAGTCATTAGGGTAAAAC
CAATGATTTATACATCTCAGGATAAGATTGAGTTTAATAAACAAATCATGGAACTACTAGAGTTAGATTTAATCCGACCTAGTAAATCACCACACA
GTTCACCAGCATTTCTCGTAGAGAATCATGCTGAGAAGAAAAGGAATAAGAAAAGAATGGTAATAAATTATAAAGCATTAAACAAAGAAACAATTG
ATGATGGGTATTATTTACCTAAAAAAGATGAATTAATAAAGTTAATCTCAGGAAAACAATGGTATAGCAGTTTCGACTGTAAGTCAGGATTCTGGC
AGGTACCATTAAAGGAAAGTTGTAAAAAACTAACAGCTTTTAGCTGTCCCTCAGGACAATTTGAGTGGAATGTACTTCCATTTGGATTAAAACAAG
CGCCAGGAATATTTCAAAGAAAAATGGATGATGCATTAGAATTAAAAAATATAACAGACAAGAGCAAACAATTTGTAAGTCTTTATGTAGATGATA
TTATCGTTTACAGTAACACAAGACAAGAACATGATAATCATCTTTTACAAACATTGTTAAGATGTAAGAAAAATGGTATTGTTCTCAGTACCAAAA
AGACACAATTATACTTAAACAAGATTAATTTTCTAGGACTTGAAATAACTGAAGGAACACATAAATTACAACCACACATACTAATAAATTTACATA
AATTCCCAGAGAAAATATGTGATAAAAAACAATTACAAAGATTTTTGGGTTGCTTGACGTACGCAGAGTGCTATATAGCTAAGTTGGCTGAAATCA
GGAAGCCACTCCAAAAGAAACTCAAAAAAGATTATGTATGGCAATGGACCCAAGAGGATACCGCCTACATAAGGAAAATAAAAAACAAATTGAAAG
ATTTTCCAACGTTATACCAACCACAGGATGAGGATTTAATGATACTTGAAACAGATGCTAGCCAAGAATATTGGTCTGGTGTTTTAAAAGCTAAAT
CATTAAAAAATGATAATCAGGAAATGCTTTGTAGATATACCTCAGGTACATTTACAGGAGCTGAACTAAATTATCACAGTAATGAAAAGGAATGGC
TCGCTGTAAAGAAAGCCATAGGAAAATTCAGGATTTACTACCTAAGGAGTTTGTTGTCCGAACGGATAATAAACAATTTGGACCTTTTATAAGGAA
CAATATTACAGGAGACTATAAACAAGGACGATTATTAAGATGGCAACAGTGGTTTAATTACTACAAGTTCACCATCGAACACATTCGAGGAGAAGA
GAATTATCTCGCAGATCTATTGACTCGAGAGTTCGCTTCATAA

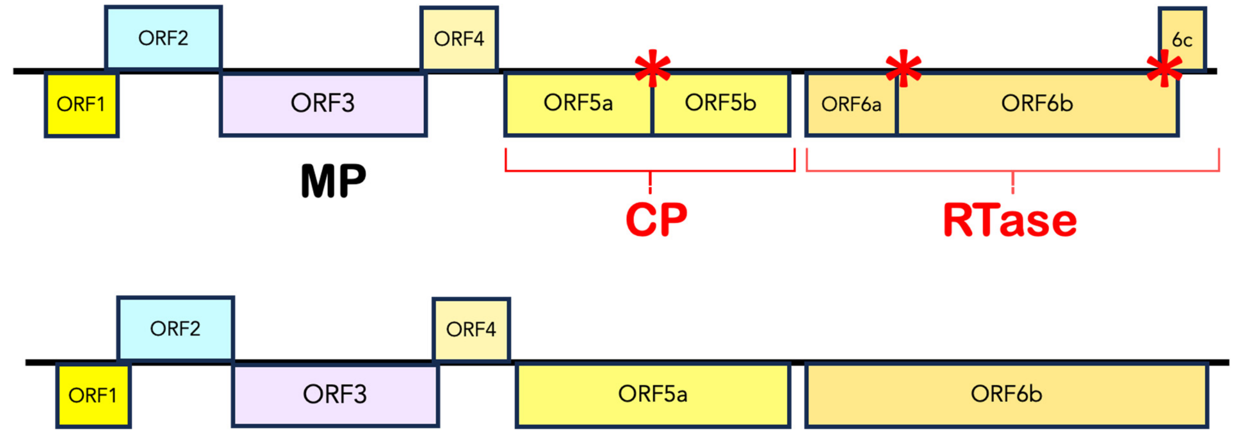


**Figure S2.** Organization of the contig DEC02-76 (up) compared to a putative functional cognate caulimovirid (down). Notice the interruptions of the open reading frames (ORFs) coding for putative viral coat protein and reverse transcriptase.

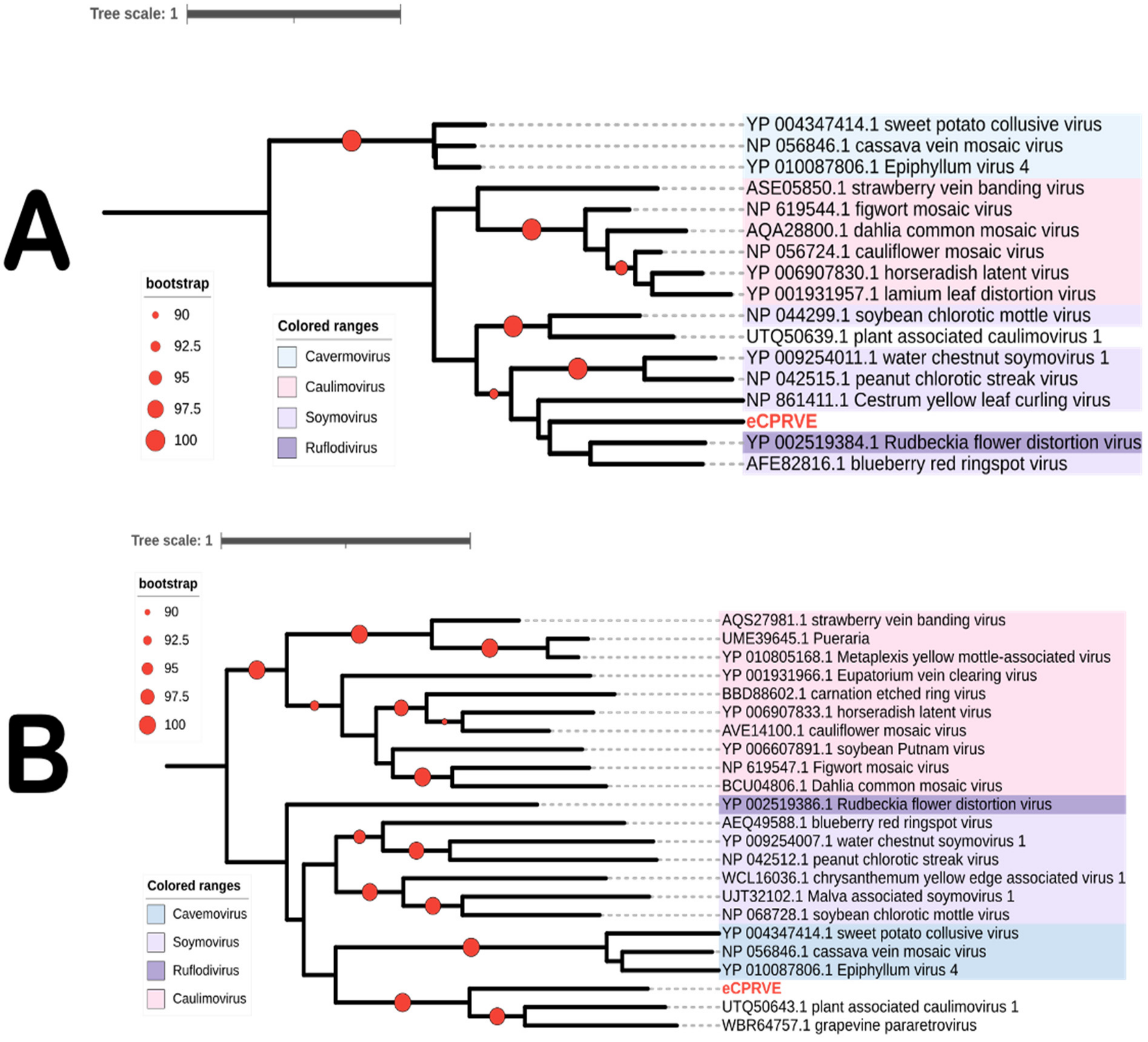


**Figure S3.** Maximum likelihood tree reconstructed on amino acid alignments of movement (**A**) and coat (**B**) proteins. The tree was reconstructed by IQtree (and visualized with iTOL v6 (Letunic and Bork, 2021). Currently recognized genera in the family *Caulimoviridae* are shaded and color-coded. Clades with statistical support of >90% are indicated by the red circles.

**Table S1.** List of cotton germplasm tested in PCR with eCPrV-specific primers.

| Species/hybrid | Code | PCR | Note |
|---|---|---|---|
| *Gossypium barbadense* | GB 10 | - | |
| *Gossypium barbadense* | GB 646 | - | |
| *Gossypium barbadense* | GB 713 | - | |
| *Gossypium barbadense* | GB 679 | - | |
| *Gossypium barbadense* | GB 379 | - | |
| *Gossypium barbadense* | GB 220 | - | |
| *Gossypium barbadense* | GB 222 | - | |
| *Gossypium barbadense* | GB 224 | - | |
| *Gossypium barbadense* | GB 245 | - | |
| *Gossypium barbadense* | GB 227 | - | |
| *Gossypium barbadense* | GB 1023 (Pima S7 CGC) | - | |
| *Gossypium barbadense* | GB 1030 (Pima S6 CGC) | - | |
| *Gossypium barbadense* | SA 1092 (Pima S1 CGC) | - | |
| *Gossypium barbadense* | Sea Island version 1 | + | Old cultivar (USA, pre-1890) |
| *Gossypium hirsutum* | Armor 9371 B3XF | + | Modern cultivar |
| *Gossypium hirsutum* | Deltapine DP 1646 B2XF | + | Modern cultivar |
| *Gossypium hirsutum* | Deltapine DP 1845 B2XF | + | Modern cultivar |
| *Gossypium hirsutum* | Deltapine DP 2115 B3XF | + | Modern cultivar |
| *Gossypium hirsutum* | Deltapine DP 2020 B3XF | + | Modern cultivar |
| *Gossypium hirsutum* | PhytoGen PHY 443 W3FE | + | Modern cultivar |
| *Gossypium hirsutum* | PhytoGen PHY 490 W3FE | + | Modern cultivar |
| *Gossypium hirsutum* | Stoneville ST 5091 B3XF | + | Modern cultivar |
| *Gossypium hirsutum* | BX 2296 B3XF | + | Modern cultivar |
| *Gossypium hirsutum* | Hopi | + | Old Hopi Indian cotton |
| *Gossypium hirsutum* | Maxxa | + | Modern Acala cultivar |
| *Gossypium hirsutum* | SG 747 | + | Modern cultivar |
| *Gossypium hirsutum* | DES 119 | + | Modern cultivar |
| *Gossypium hirsutum* | FiberMax FM 832 B2R | + | Modern cultivar |
| *Gossypium hirsutum* | Stoneville 2B | + | Old cultivar |
| *Gossypium hirsutum* | Stoneville 2 | + | Old cultivar (USA 1929) |
| *Gossypium hirsutum* | Deltapine 16 | + | Old cultivar |
| *Gossypium hirsutum* | FiberMax FM 958 B2R | + | Modern cultivar |
| *Gossypium hirsutum* | PD 09046 | + | Pee Dee ARS line |
| *Gossypium hirsutum* | Acala 1517 | + | Modern Acala cultivar |
| *Gossypium hirsutum* | Alamo | + | Old cultivar |
| *Gossypium hirsutum* | Kekchi | + | Old cultivar (USA 1904) |
| *Gossypium hirsutum* | Lone Star | + | Old cultivar (USA 1905) |
| *Gossypium hirsutum* | Dixie Triumph | + | Old cultivar (USA 1915) |
| *Gossypium hirsutum* | Macha | + | Old cultivar (USA 1935) |
| *Gossypium hirsutum* | Marie Galante | + | Moco cotton – wild (Brazil) |
| *Gossypium hirsutum* | Uzbek S9 UEL | + | Uzbekistan |
| *Gossypium hirsutum* | CIM-632 | + | Pakistan |
| *Gossypium hirsutum* | Nankeen spot | + | China (mutant, wild) |
| *Gossypium hirsutum* | CSX 215 | + | Old cultivar (Australia) |
| *Gossypium hirsutum* | UKA J2 (72) 017 | + | Old cultivar (Swaziland) |
| *Gossypium hirsutum* | ALA 70-1 | + | Old cultivar (Tanzania) |
| *Gossypium hirsutum* | Reba B50 | + | Old cultivar (Chad) |
| *Gossypium hirsutum* | CEDIX | + | Old cultivar (El Salvador or Africa) |
| *Gossypium hirsutum* | Latifolium | + | Cotton landrace |
| *Gossypium tomentosum* | AD3-5 | - | Tetraploid, native to Hawaii |
| *Gossypium tomentosum* | AD3 CMD panel 11 | - | Tetraploid, native to Hawaii |
| *Gossypium mustelinum* | AD4-1 | - | Tetraploid, native to Brazil |
| *Gossypium mustelinum* | AD4 CMD panel 12 | - | Tetraploid, native to Brazil |
| *Gossypium darwinii* | AD5-20 | - | Tetraploid, native to Galapagos |
| *Gossypium darwinii* | AD5-17 | - | Tetraploid, native to Galapagos |
| *Gossypium herbaceum* | A1 CGC | - | Diploid, similar to cotton A genome |
| *Gossypium arboreum* | AKA 8401 | - | Diploid, similar to cotton A genome |
| *Gossypium raimondii* | D5-3 CMD panel 10 | - | Diploid, similar to cotton D genome |
| *Gossypium sturtianum* | C1-1 | - | Diploid, native to Australia |
| *Gossypium thuberi* | D1-11 | - | Diploid, native to Mexico and SW USA |

**Table S2**. List of *Gossypium* and *Gossypoides* spp. screened for the presence of eCPRVE sequences.

| Cotton species (genotype) | Genome assembly |
|---|---|
| **Tetraploids** | |
| *Gossypium hirsutum* (AD1) | 'TM-1' UTX_v2.1 |
| *Gossypium hirsutum* (AD1) | Bar32 NSF v.10 |
| *Gossypium hirsutum* (AD1) | 'NDM8' HEAU_v1 |
| *Gossypium hirsutum* (AD1) | B713 NSF v1.0 |
| *Gossypium hirsutum* (AD1) | 'TM-1' WHU_updated v1 |
| *Gossypium hirsutum* (AD1) | 'TM-1' CRI_v1 |
| *Gossypium hirsutum* (AD1) | 'ZM24' CRI_v1 |
| *Gossypium hirsutum* (AD1) | 'TM-1' ZJU-improved_v2.1_a1 |
| *Gossypium hirsutum* (AD1) | 'TM-1' HAU_v1 / v1.1 |
| *Gossypium hirsutum* (AD1) | JGI-Interim-release _v1.1 |
| *Gossypium hirsutum* (AD1) | 'TM-1' NBI_v1.1_a1.1 |
| *Gossypium hirsutum* (AD1) | BGI_v1_a1 |
| *Gossypium barbadense* (AD2) | 'Pima90' HEAU_v1 |
| *Gossypium barbadense* (AD2) | '3-79' HGS_v1.1 |
| *Gossypium barbadense* (AD2) | 'H7124' ZJU_v1.1_a1 |
| *Gossypium barbadense* (AD2) | '3-79' HAU_v1 |
| *Gossypium tomentosum* (AD3) | HGS_v1.1 |
| *Gossypium tomentosum* (AD3) | HAU v1.0 |
| *Gossypium tomentosum* (AD3) | HAU v2.0 |
| *Gossypium mustelinum* (AD4) | JGI_v1.1 |
| *Gossypium darwinii* (AD5) | HGS_v1.1 |
| *Gossypium ekmanianum* (AD6) | 'AD602' CRI v1 |
| **Diploids** | |
| *Gossypioides kirkii* | ISU-v1.0 |
| *Gossypium herbaceum* (A1) | 'Mutema' WHU_v1 |
| *Gossypium herbaceum* (A1) | A1-Wagad |
| *Gossypium arboreum* (A2) | 'SXY1' HAU v1 |
| *Gossypium arboreum* (A2) | NCGR |
| *Gossypium arboreum* (A2) | 'SXY1' WHU-updated v1 |
| *Gossypium arboreum* (A2) | 'SXY1' CRI-updated_v1 |
| *Gossypium arboreum* (A2) | 'SXY1' CGP-BGI_v2_a1 |
| *Gossypium anomalum* (B1) | B1 NSF_v1 |
| *Gossypium thurberi* (D1-5) | CRI_v1 |
| *Gossypium thurberi* (D1-35) | ISU_v1 |
| *Gossypium armourianum* (D2-1) | D21 ISU_v1 |
| *Gossypium harknessii* (D2-2) | ISU_v1 |
| *Gossypium davidsonii* (D3d-8) | CRI_v1 |
| *Gossypium davidsonii* (D3d-27) | ISU_v1 |
| *Gossypium klotzschianum* (D3k) | ISU_v1 |
| *Gossypium aridum* (D4) | ISU_v1 |
| *Gossypium raimondii* (D5) | 'Grai D502' HAU v1 |
| *Gossypium raimondii* (D5) | 'D5-8' e ISU_v1 |
| *Gossypium raimondii* (D5) | 'D5-4' NSF_v1 |
| *Gossypium raimondii* (D5) | JGI_v2_a2.1 |
| *Gossypium raimondii* (D5) | BGI-CGP-draft_v1 |
| *Gossypium gossypioides* (D6) | ISU_v1 |
| *Gossypium lobatum* (D7) | ISU_v1 |
| *Gossypium trilobum* (D8) | ISU_v1 |
| *Gossypium laxum* (D9) | ISU_v1 |
| *Gossypium turneri* (D10) | NSF_v1_a2 |
| *Gossypium schwendimanii* (D11) | ISU_v1 |
| *Gossypium stocksii* (E1) | NSF_v1 |
| *Gossypium stocksii* (E1) | NSF_v1 |
| *Gossypium longicalyx* (F1) | ZSTU v1.2 a.1.1 |
| *Gossypium australe* (G2) | CRI_v1.1 |
| *Gossypium rotundifolium* (K12) | 'Grot K201' HAU v1 |

**Table S3.** Percentage (%) nt identity among 21 eCPRVE contigs generated in this work by HTS

| | | | | | | | | | | | | | | | | | | | | | |
|---|---|---|---|---|---|---|---|---|---|---|---|---|---|---|---|---|---|---|---|---|---|
| 1: DEC02-76 | 100.00 | 100.00 | 100.00 | 100.00 | 100.00 | 100.00 | 100.00 | 99.97 | 99.97 | 99.96 | 100.00 | 99.98 | 100.00 | 100.00 | 100.00 | 99.84 | 100.00 | 99.98 | 100.00 | 100.00 | 100.00 |
| 2: CLR03-38395 | 100.00 | 100.00 | 100.00 | 100.00 | 100.00 | 100.00 | 100.00 | 99.98 | 99.98 | 100.00 | 100.00 | 100.00 | 100.00 | 100.00 | 100.00 | 99.87 | 100.00 | 100.00 | 100.00 | 100.00 | 100.00 |
| 3: CLR01-43699 | 100.00 | 100.00 | 100.00 | 100.00 | 100.00 | 100.00 | 100.00 | 99.98 | 99.98 | 99.97 | 100.00 | 99.99 | 100.00 | 100.00 | 100.00 | 99.87 | 100.00 | 99.98 | 100.00 | 100.00 | 100.00 |
| 4: APR16-68 | 100.00 | 100.00 | 100.00 | 100.00 | 100.00 | 100.00 | 100.00 | 99.98 | 99.98 | 100.00 | 100.00 | 99.98 | 100.00 | 100.00 | 100.00 | 99.87 | 100.00 | 100.00 | 100.00 | 100.00 | 100.00 |
| 5: APR18-62 | 100.00 | 100.00 | 100.00 | 100.00 | 100.00 | 100.00 | 100.00 | 99.98 | 99.98 | 99.98 | 100.00 | 99.98 | 100.00 | 100.00 | 100.00 | 99.87 | 100.00 | 99.98 | 100.00 | 100.00 | 100.00 |
| 6: APR19-43 | 100.00 | 100.00 | 100.00 | 100.00 | 100.00 | 100.00 | 100.00 | 99.98 | 99.98 | 99.97 | 100.00 | 99.98 | 100.00 | 100.00 | 100.00 | 99.87 | 100.00 | 99.98 | 100.00 | 100.00 | 100.00 |
| 7: APR20-70 | 100.00 | 100.00 | 100.00 | 100.00 | 100.00 | 100.00 | 100.00 | 99.98 | 99.98 | 100.00 | 100.00 | 100.00 | 100.00 | 100.00 | 100.00 | 99.92 | 100.00 | 100.00 | 100.00 | 100.00 | 100.00 |
| 8: DEC10-249 | 99.97 | 99.98 | 99.98 | 99.98 | 99.98 | 99.98 | 99.98 | 100.00 | 99.98 | 99.98 | 99.98 | 99.98 | 99.98 | 99.98 | 99.98 | 99.98 | 99.98 | 99.98 | 99.98 | 99.98 | 100.00 |
| 9: DEC08-241 | 99.97 | 99.98 | 99.98 | 99.98 | 99.98 | 99.98 | 99.98 | 99.98 | 100.00 | 99.98 | 99.98 | 99.98 | 99.98 | 99.98 | 99.98 | 99.98 | 99.98 | 99.98 | 100.00 | 99.98 | 100.00 |
| 10: DEC06-81 | 99.96 | 100.00 | 99.97 | 100.00 | 99.98 | 99.97 | 100.00 | 99.98 | 99.98 | 100.00 | 99.97 | 99.95 | 100.00 | 100.00 | 100.00 | 99.87 | 100.00 | 99.98 | 100.00 | 100.00 | 100.00 |
| 11: DEC04-13db | 100.00 | 100.00 | 100.00 | 100.00 | 100.00 | 100.00 | 100.00 | 99.98 | 99.98 | 99.97 | 100.00 | 99.99 | 100.00 | 100.00 | 100.00 | 99.87 | 100.00 | 99.98 | 100.00 | 100.00 | 100.00 |
| 12: MAY17-11 | 99.98 | 100.00 | 99.99 | 99.98 | 99.98 | 99.98 | 100.00 | 99.98 | 99.98 | 99.95 | 99.99 | 100.00 | 100.00 | 100.00 | 100.00 | 99.87 | 100.00 | 99.97 | 100.00 | 100.00 | 100.00 |
| 13: MAY15-78 | 100.00 | 100.00 | 100.00 | 100.00 | 100.00 | 100.00 | 100.00 | 99.98 | 99.98 | 100.00 | 100.00 | 100.00 | 100.00 | 100.00 | 100.00 | 99.97 | 100.00 | 100.00 | 100.00 | 100.00 | 100.00 |
| 14: JUL05-102 | 100.00 | 100.00 | 100.00 | 100.00 | 100.00 | 100.00 | 100.00 | 99.98 | 99.98 | 100.00 | 100.00 | 100.00 | 100.00 | 100.00 | 100.00 | 100.00 | 100.00 | 100.00 | 100.00 | 100.00 | 100.00 |
| 15: JUL04-107 | 100.00 | 100.00 | 100.00 | 100.00 | 100.00 | 100.00 | 100.00 | 99.98 | 99.98 | 100.00 | 100.00 | 100.00 | 100.00 | 100.00 | 100.00 | 99.97 | 100.00 | 100.00 | 100.00 | 100.00 | 100.00 |
| 16: JUL03-55 | 99.84 | 99.87 | 99.87 | 99.87 | 99.87 | 99.87 | 99.92 | 99.98 | 99.98 | 99.87 | 99.87 | 99.87 | 99.97 | 100.00 | 99.97 | 100.00 | 100.00 | 99.87 | 100.00 | 100.00 | 100.00 |
| 17: JUL02-106 | 100.00 | 100.00 | 100.00 | 100.00 | 100.00 | 100.00 | 100.00 | 99.98 | 99.98 | 100.00 | 100.00 | 100.00 | 100.00 | 100.00 | 100.00 | 100.00 | 100.00 | 100.00 | 100.00 | 100.00 | 100.00 |
| 18: JUL01-85 | 99.98 | 100.00 | 99.98 | 100.00 | 99.98 | 99.98 | 100.00 | 99.98 | 99.98 | 99.98 | 99.98 | 99.97 | 100.00 | 100.00 | 100.00 | 99.87 | 100.00 | 100.00 | 100.00 | 100.00 | 100.00 |
| 19: DEC03-186 | 100.00 | 100.00 | 100.00 | 100.00 | 100.00 | 100.00 | 100.00 | 99.98 | 100.00 | 100.00 | 100.00 | 100.00 | 100.00 | 100.00 | 100.00 | 100.00 | 100.00 | 100.00 | 100.00 | 100.00 | 100.00 |
| 20: DEC07-98 | 100.00 | 100.00 | 100.00 | 100.00 | 100.00 | 100.00 | 100.00 | 99.98 | 99.98 | 100.00 | 100.00 | 100.00 | 100.00 | 100.00 | 100.00 | 100.00 | 100.00 | 100.00 | 100.00 | 100.00 | 100.00 |
| 21: APR17-291 | 100.00 | 100.00 | 100.00 | 100.00 | 100.00 | 100.00 | 100.00 | 100.00 | 100.00 | 100.00 | 100.00 | 100.00 | 100.00 | 100.00 | 100.00 | 100.00 | 100.00 | 100.00 | 100.00 | 100.00 | 100.00 |

**Figure S4.** Clustal Omega alignment of 21 caulimovirid-like contigs generated with HTS.

```
DEC02-76        ------------------------------------------------------------   0
CLR03-38395     ------------------------------------------------------------   0
CLR01-43699     ------------------------------------------------------------   0
APR16-68        ------------------------------------------------------------   0
APR18-62        ------------------------------------------------------------   0
APR19-43        ------------------------------------------------------------   0
APR20-70        ------------------------------------------------------------   0
DEC10-249       ------------------------------------------------------------   0
DEC08-241       ------------------------------------------------------------   0
DEC06-81        ------------------------------------------------------------   0
DEC04-13db      AATTGAGCTATACATGCTGCATTATCTTCGTATAAGATAGTTGGCATATTTTCCTGTAAA   60
MAY17-11        ------------------------------------------------------------   0
MAY15-78        ------------------------------------------------------------   0
JUL05-102       ------------------------------------------------------------   0
JUL04-107       ------------------------------------------------------------   0
JUL03-55        ------------------------------------------------------------   0
JUL02-106       ------------------------------------------------------------   0
JUL01-85        ------------------------------------------------------------   0
DEC03-186       ------------------------------------------------------------   0
DEC07-98        ------------------------------------------------------------   0
APR17-291       ------------------------------------------------------------   0


DEC02-76        ------------------------------------------------------------   0
CLR03-38395     ------------------------------------------------------------   0
CLR01-43699     ------------------------------------------------------------   0
APR16-68        ------------------------------------------------------------   0
APR18-62        ------------------------------------------------------------   0
APR19-43        ------------------------------------------------------------   0
APR20-70        ------------------------------------------------------------   0
DEC10-249       ------------------------------------------------------------   0
DEC08-241       ------------------------------------------------------------   0
DEC06-81        ------------------------------------------------------------   0
DEC04-13db      GGCAAATTACAAGATAGTTGGCATATTTTCCTATAAAGGCAAATTACATATCTTCTGAAT   120
MAY17-11        ------------------------------------------------------------   0
MAY15-78        ------------------------------------------------------------   0
JUL05-102       ------------------------------------------------------------   0
JUL04-107       ------------------------------------------------------------   0
JUL03-55        ------------------------------------------------------------   0
JUL02-106       ------------------------------------------------------------   0
JUL01-85        ------------------------------------------------------------   0
DEC03-186       ------------------------------------------------------------   0
DEC07-98        ------------------------------------------------------------   0
APR17-291       ------------------------------------------------------------   0


DEC02-76        ------------------------------------------------------------   0
CLR03-38395     ------------------------------------------------------------   0
CLR01-43699     ------------------------------------------------------------   0
APR16-68        ------------------------------------------------------------   0
APR18-62        ------------------------------------------------------------   0
APR19-43        ------------------------------------------------------------   0
APR20-70        ------------------------------------------------------------   0
DEC10-249       ------------------------------------------------------------   0
DEC08-241       ------------------------------------------------------------   0
DEC06-81        ------------------------------------------------------------   0
DEC04-13db      ATGTTGGGTTAATAACCTTAGCCAAACACACTCTCGGCTTGCCTCATGCATTGCAATTAT   180
MAY17-11        ------------------------------------------------------------   0
```

Figure S4

```
MAY15-78        ------------------------------------------------------------   0
JUL05-102       ------------------------------------------------------------   0
JUL04-107       ------------------------------------------------------------   0
JUL03-55        ------------------------------------------------------------   0
JUL02-106       ------------------------------------------------------------   0
JUL01-85        ------------------------------------------------------------   0
DEC03-186       ------------------------------------------------------------   0
DEC07-98        ------------------------------------------------------------   0
APR17-291       ------------------------------------------------------------   0


DEC02-76        ------------------------------------------------------------   0
CLR03-38395     ------------------------------------------------------------   0
CLR01-43699     ------------------------------------------------------------   0
APR16-68        ------------------------------------------------------------   0
APR18-62        ------------------------------------------------------------   0
APR19-43        ------------------------------------------------------------   0
APR20-70        ------------------------------------------------------------   0
DEC10-249       ------------------------------------------------------------   0
DEC08-241       ------------------------------------------------------------   0
DEC06-81        ------------------------------------------------------------   0
DEC04-13db      TTCAGCATGATTTGAAGAGGCAGCAGCTAATGTTTGCTTTGTCGAACGCCATGATATGTC   240
MAY17-11        ------------------------------------------------------------   0
MAY15-78        ------------------------------------------------------------   0
JUL05-102       ------------------------------------------------------------   0
JUL04-107       ------------------------------------------------------------   0
JUL03-55        ------------------------------------------------------------   0
JUL02-106       ------------------------------------------------------------   0
JUL01-85        ------------------------------------------------------------   0
DEC03-186       ------------------------------------------------------------   0
DEC07-98        ------------------------------------------------------------   0
APR17-291       ------------------------------------------------------------   0


DEC02-76        ------------------------------------------------------------   0
CLR03-38395     ------------------------------------------------------------   0
CLR01-43699     ------------------------------------------------------------   0
APR16-68        ------------------------------------------------------------   0
APR18-62        ------------------------------------------------------------   0
APR19-43        ------------------------------------------------------------   0
APR20-70        ------------------------------------------------------------   0
DEC10-249       ------------------------------------------------------------   0
DEC08-241       ------------------------------------------------------------   0
DEC06-81        ------------------------------------------------------------   0
DEC04-13db      TGTACCTCCACATGTAAATAAATATCCCATTTGAGATTGACCTTTATATGGATCCGACAA   300
MAY17-11        ------------------------------------------------------------   0
MAY15-78        ------------------------------------------------------------   0
JUL05-102       ------------------------------------------------------------   0
JUL04-107       ------------------------------------------------------------   0
JUL03-55        ------------------------------------------------------------   0
JUL02-106       ------------------------------------------------------------   0
JUL01-85        ------------------------------------------------------------   0
DEC03-186       ------------------------------------------------------------   0
DEC07-98        ------------------------------------------------------------   0
APR17-291       ------------------------------------------------------------   0


DEC02-76        ------------------------------------------------------------   0
CLR03-38395     ------------------------------------------------------------   0
CLR01-43699     ------------------------------------------------------------   0
APR16-68        ------------------------------------------------------------   0
APR18-62        ------------------------------------------------------------   0
APR19-43        ------------------------------------------------------------   0
```

Figure S4

```
APR20-70          ------------------------------------------------------------   0
DEC10-249         ------------------------------------------------------------   0
DEC08-241         ------------------------------------------------------------   0
DEC06-81          ------------------------------------------------------------   0
DEC04-13db        ATATCCAGCATCAACATAACCAATTAATAGGGATTTTGAATCATTTGAATAAAATAACCC   360
MAY17-11          ------------------------------------------------------------   0
MAY15-78          ------------------------------------------------------------   0
JUL05-102         ------------------------------------------------------------   0
JUL04-107         ------------------------------------------------------------   0
JUL03-55          ------------------------------------------------------------   0
JUL02-106         ------------------------------------------------------------   0
JUL01-85          ------------------------------------------------------------   0
DEC03-186         ------------------------------------------------------------   0
DEC07-98          ------------------------------------------------------------   0
APR17-291         ------------------------------------------------------------   0


DEC02-76          ------------------------------------------------------------   0
CLR03-38395       ------------------------------------------------------------   0
CLR01-43699       ------------------------------------------------------------   0
APR16-68          ------------------------------------------------------------   0
APR18-62          ------------------------------------------------------------   0
APR19-43          ------------------------------------------------------------   0
APR20-70          ------------------------------------------------------------   0
DEC10-249         ------------------------------------------------------------   0
DEC08-241         ------------------------------------------------------------   0
DEC06-81          ------------------------------------------------------------   0
DEC04-13db        CATATTAATGGTCCCTCTAAGATATCTAAATACATGTTTAATTCTATTCCAATGTTTACG   420
MAY17-11          ------------------------------------------------------------   0
MAY15-78          ------------------------------------------------------------   0
JUL05-102         ------------------------------------------------------------   0
JUL04-107         ------------------------------------------------------------   0
JUL03-55          ------------------------------------------------------------   0
JUL02-106         ------------------------------------------------------------   0
JUL01-85          ------------------------------------------------------------   0
DEC03-186         ------------------------------------------------------------   0
DEC07-98          ------------------------------------------------------------   0
APR17-291         ------------------------------------------------------------   0


DEC02-76          ------------------------------------------------------------   0
CLR03-38395       ------------------------------------------------------------   0
CLR01-43699       ------------------------------------------------------------   0
APR16-68          ------------------------------------------------------------   0
APR18-62          ------------------------------------------------------------   0
APR19-43          ------------------------------------------------------------   0
APR20-70          ------------------------------------------------------------   0
DEC10-249         ------------------------------------------------------------   0
DEC08-241         ------------------------------------------------------------   0
DEC06-81          ------------------------------------------------------------   0
DEC04-13db        TGTTAAAGAAGAACTAAATCTTGCTAACAAGTTTACACAAAAAGCTATATCAGGTCTTGT   480
MAY17-11          ------------------------------------------------------------   0
MAY15-78          ------------------------------------------------------------   0
JUL05-102         ------------------------------------------------------------   0
JUL04-107         ------------------------------------------------------------   0
JUL03-55          ------------------------------------------------------------   0
JUL02-106         ------------------------------------------------------------   0
JUL01-85          ------------------------------------------------------------   0
DEC03-186         ------------------------------------------------------------   0
DEC07-98          ------------------------------------------------------------   0
APR17-291         ------------------------------------------------------------   0
```

```
DEC02-76        ------------------------------------------------------------   0
CLR03-38395     ------------------------------------------------------------   0
CLR01-43699     ------------------------------------------------------------   0
APR16-68        ------------------------------------------------------------   0
APR18-62        ------------------------------------------------------------   0
APR19-43        ------------------------------------------------------------   0
APR20-70        ------------------------------------------------------------   0
DEC10-249       ------------------------------------------------------------   0
DEC08-241       ------------------------------------------------------------   0
DEC06-81        ------------------------------------------------------------   0
DEC04-13db      GTTGTTTGTAAGATACATCAATGCTCCTATGGCACTTAGATATGGTACTTCAGGACTAAG   540
MAY17-11        ------------------------------------------------------------   0
MAY15-78        ------------------------------------------------------------   0
JUL05-102       ------------------------------------------------------------   0
JUL04-107       ------------------------------------------------------------   0
JUL03-55        ------------------------------------------------------------   0
JUL02-106       ------------------------------------------------------------   0
JUL01-85        ------------------------------------------------------------   0
DEC03-186       ------------------------------------------------------------   0
DEC07-98        ------------------------------------------------------------   0
APR17-291       ------------------------------------------------------------   0


DEC02-76        ------------------------------------------------------------   0
CLR03-38395     ------------------------------------------------------------   0
CLR01-43699     ------------------------------------------------------------   0
APR16-68        ------------------------------------------------------------   0
APR18-62        ------------------------------------------------------------   0
APR19-43        ------------------------------------------------------------   0
APR20-70        ------------------------------------------------------------   0
DEC10-249       ------------------------------------------------------------   0
DEC08-241       ------------------------------------------------------------   0
DEC06-81        ------------------------------------------------------------   0
DEC04-13db      AACTCTACATCATTCTCGCAATGACGAAATTGATCTTTATTCACATCTAACGATCATACA   600
MAY17-11        ------------------------------------------------------------   0
MAY15-78        ------------------------------------------------------------   0
JUL05-102       ------------------------------------------------------------   0
JUL04-107       ------------------------------------------------------------   0
JUL03-55        ------------------------------------------------------------   0
JUL02-106       ------------------------------------------------------------   0
JUL01-85        ------------------------------------------------------------   0
DEC03-186       ------------------------------------------------------------   0
DEC07-98        ------------------------------------------------------------   0
APR17-291       ------------------------------------------------------------   0


DEC02-76        ------------------------------------------------------------   0
CLR03-38395     ------------------------------------------------------------   0
CLR01-43699     ------------------------------------------------------------   0
APR16-68        ------------------------------------------------------------   0
APR18-62        ------------------------------------------------------------   0
APR19-43        ------------------------------------------------------------   0
APR20-70        ------------------------------------------------------------   0
DEC10-249       ------------------------------------------------------------   0
DEC08-241       ------------------------------------------------------------   0
DEC06-81        ------------------------------------------------------------   0
DEC04-13db      ACCATCGGGGTACTCAATGGATTTTCTTTATCCATATAAATTTCTTTAAGATCTTTTTC   660
MAY17-11        ------------------------------------------------------------   0
MAY15-78        ------------------------------------------------------------   0
JUL05-102       ------------------------------------------------------------   0
JUL04-107       ------------------------------------------------------------   0
JUL03-55        ------------------------------------------------------------   0
JUL02-106       ------------------------------------------------------------   0
```

```
JUL01-85          ------------------------------------------------------------   0
DEC03-186         ------------------------------------------------------------   0
DEC07-98          ------------------------------------------------------------   0
APR17-291         ------------------------------------------------------------   0


DEC02-76          ------------------------------------------------------------   0
CLR03-38395       ------------------------------------------------------------   0
CLR01-43699       ------------------------------------------------------------   0
APR16-68          ------------------------------------------------------------   0
APR18-62          ------------------------------------------------------------   0
APR19-43          ------------------------------------------------------------   0
APR20-70          ------------------------------------------------------------   0
DEC10-249         ------------------------------------------------------------   0
DEC08-241         ------------------------------------------------------------   0
DEC06-81          ------------------------------------------------------------   0
DEC04-13db        GTATAAGTTGACTGATGGACTTGAATTTTATCTTTTAAATGCTCGAACTGCAGGCTAAGA   720
MAY17-11          ------------------------------------------------------------   0
MAY15-78          ------------------------------------------------------------   0
JUL05-102         ------------------------------------------------------------   0
JUL04-107         ------------------------------------------------------------   0
JUL03-55          ------------------------------------------------------------   0
JUL02-106         ------------------------------------------------------------   0
JUL01-85          ------------------------------------------------------------   0
DEC03-186         ------------------------------------------------------------   0
DEC07-98          ------------------------------------------------------------   0
APR17-291         ------------------------------------------------------------   0


DEC02-76          ------------------------------------------------------------   0
CLR03-38395       ------------------------------------------------------------   0
CLR01-43699       ------------------------------------------------------------   0
APR16-68          ------------------------------------------------------------   0
APR18-62          ------------------------------------------------------------   0
APR19-43          ------------------------------------------------------------   0
APR20-70          ------------------------------------------------------------   0
DEC10-249         ------------------------------------------------------------   0
DEC08-241         ------------------------------------------------------------   0
DEC06-81          ------------------------------------------------------------   0
DEC04-13db        AAAAACTTTGTTTTTCCAAGATCTTTCATCTCAAATTCTTTCTTTAAATAATTTACTGCA   780
MAY17-11          ------------------------------------------------------------   0
MAY15-78          ------------------------------------------------------------   0
JUL05-102         ------------------------------------------------------------   0
JUL04-107         ------------------------------------------------------------   0
JUL03-55          ------------------------------------------------------------   0
JUL02-106         ------------------------------------------------------------   0
JUL01-85          ------------------------------------------------------------   0
DEC03-186         ------------------------------------------------------------   0
DEC07-98          ------------------------------------------------------------   0
APR17-291         ------------------------------------------------------------   0


DEC02-76          ------------------------------------------------------------   0
CLR03-38395       ------------------------------------------------------------   0
CLR01-43699       ------------------------------------------------------------   0
APR16-68          ------------------------------------------------------------   0
APR18-62          ------------------------------------------------------------   0
APR19-43          ------------------------------------------------------------   0
APR20-70          ------------------------------------------------------------   0
DEC10-249         ------------------------------------------------------------   0
DEC08-241         ------------------------------------------------------------   0
DEC06-81          ------------------------------------------------------------   0
DEC04-13db        TTTTGAGGCTTTTCAAGTGTTCTAATAATATTTAGATCATCAACATAAATAGAAATTATC   840
```

```
MAY17-11        ------------------------------------------------------------   0
MAY15-78        ------------------------------------------------------------   0
JUL05-102       ------------------------------------------------------------   0
JUL04-107       ------------------------------------------------------------   0
JUL03-55        ------------------------------------------------------------   0
JUL02-106       ------------------------------------------------------------   0
JUL01-85        ------------------------------------------------------------   0
DEC03-186       ------------------------------------------------------------   0
DEC07-98        ------------------------------------------------------------   0
APR17-291       ------------------------------------------------------------   0


DEC02-76        ------------------------------------------------------------   0
CLR03-38395     ------------------------------------------------------------   0
CLR01-43699     ------------------------------------------------------------   0
APR16-68        ------------------------------------------------------------   0
APR18-62        ------------------------------------------------------------   0
APR19-43        ------------------------------------------------------------   0
APR20-70        ------------------------------------------------------------   0
DEC10-249       ------------------------------------------------------------   0
DEC08-241       ------------------------------------------------------------   0
DEC06-81        ------------------------------------------------------------   0
DEC04-13db      ACAAATTTTGATTTAGACCTTTTTATAAAAACACATGGGCAGATTGGATCGTTTTTATAA   900
MAY17-11        ------------------------------------------------------------   0
MAY15-78        ------------------------------------------------------------   0
JUL05-102       ------------------------------------------------------------   0
JUL04-107       ------------------------------------------------------------   0
JUL03-55        ------------------------------------------------------------   0
JUL02-106       ------------------------------------------------------------   0
JUL01-85        ------------------------------------------------------------   0
DEC03-186       ------------------------------------------------------------   0
DEC07-98        ------------------------------------------------------------   0
APR17-291       ------------------------------------------------------------   0


DEC02-76        ------------------------------------------------------------   0
CLR03-38395     ------------------------------------------------------------   0
CLR01-43699     ------------------------------------------------------------   0
APR16-68        ------------------------------------------------------------   0
APR18-62        ------------------------------------------------------------   0
APR19-43        ------------------------------------------------------------   0
APR20-70        ------------------------------------------------------------   0
DEC10-249       ------------------------------------------------------------   0
DEC08-241       ------------------------------------------------------------   0
DEC06-81        ------------------------------------------------------------   0
DEC04-13db      CATTCATTTAACAAATATTCACTAAGACAATTGTACCACATAAGTCCAGATTGTTTTAAT   960
MAY17-11        ------------------------------------------------------------   0
MAY15-78        ------------------------------------------------------------   0
JUL05-102       ------------------------------------------------------------   0
JUL04-107       ------------------------------------------------------------   0
JUL03-55        ------------------------------------------------------------   0
JUL02-106       ------------------------------------------------------------   0
JUL01-85        ------------------------------------------------------------   0
DEC03-186       ------------------------------------------------------------   0
DEC07-98        ------------------------------------------------------------   0
APR17-291       ------------------------------------------------------------   0


DEC02-76        ------------------------------------------------------------   0
CLR03-38395     ------------------------------------------------------------   0
CLR01-43699     ------------------------------------------------------------   0
APR16-68        ------------------------------------------------------------   0
APR18-62        ------------------------------------------------------------   0
```

Figure S4

```
APR19-43        ------------------------------------------------------------   0
APR20-70        ------------------------------------------------------------   0
DEC10-249       ------------------------------------------------------------   0
DEC08-241       ------------------------------------------------------------   0
DEC06-81        ------------------------------------------------------------   0
DEC04-13db      CCATATAAACTTTTCTTTAATCTGATTGAGCAATTTTCCCGGGAAACTCTGTATCCTTCA   1020
MAY17-11        ------------------------------------------------------------   0
MAY15-78        ------------------------------------------------------------   0
JUL05-102       ------------------------------------------------------------   0
JUL04-107       ------------------------------------------------------------   0
JUL03-55        ------------------------------------------------------------   0
JUL02-106       ------------------------------------------------------------   0
JUL01-85        ------------------------------------------------------------   0
DEC03-186       ------------------------------------------------------------   0
DEC07-98        ------------------------------------------------------------   0
APR17-291       ------------------------------------------------------------   0


DEC02-76        ------------------------------------------------------------   0
CLR03-38395     ------------------------------------------------------------   0
CLR01-43699     ------------------------------------------------------------   0
APR16-68        ------------------------------------------------------------   0
APR18-62        ------------------------------------------------------------   0
APR19-43        ------------------------------------------------------------   0
APR20-70        ------------------------------------------------------------   0
DEC10-249       ------------------------------------------------------------   0
DEC08-241       ------------------------------------------------------------   0
DEC06-81        ------------------------------------------------------------   0
DEC04-13db      GGGATTTTAAATCCTTCTAGGATTTTCATATAAATTTCATTATCAAGTGTACCATACAAA   1080
MAY17-11        ------------------------------------------------------------   0
MAY15-78        ------------------------------------------------------------   0
JUL05-102       ------------------------------------------------------------   0
JUL04-107       ------------------------------------------------------------   0
JUL03-55        ------------------------------------------------------------   0
JUL02-106       ------------------------------------------------------------   0
JUL01-85        ------------------------------------------------------------   0
DEC03-186       ------------------------------------------------------------   0
DEC07-98        ------------------------------------------------------------   0
APR17-291       ------------------------------------------------------------   0


DEC02-76        ------------------------------------------------------------   0
CLR03-38395     ------------------------------------------------------------   0
CLR01-43699     ------------------------------------------------------------   0
APR16-68        ------------------------------------------------------------   0
APR18-62        ------------------------------------------------------------   0
APR19-43        ------------------------------------------------------------   0
APR20-70        ------------------------------------------------------------   0
DEC10-249       ------------------------------------------------------------   0
DEC08-241       ------------------------------------------------------------   0
DEC06-81        ------------------------------------------------------------   0
DEC04-13db      TAGGCTATAACAACATCCATTAGACATGTCAAGTTTTTCATGTACTGCTAAACTAATAAG   1140
MAY17-11        ------------------------------------------------------------   0
MAY15-78        ------------------------------------------------------------   0
JUL05-102       ------------------------------------------------------------   0
JUL04-107       ------------------------------------------------------------   0
JUL03-55        ------------------------------------------------------------   0
JUL02-106       ------------------------------------------------------------   0
JUL01-85        ------------------------------------------------------------   0
DEC03-186       ------------------------------------------------------------   0
DEC07-98        ------------------------------------------------------------   0
APR17-291       ------------------------------------------------------------   0
```

```
DEC02-76        ------------------------------------------------------------   0
CLR03-38395     ------------------------------------------------------------   0
CLR01-43699     ------------------------------------------------------------   0
APR16-68        ------------------------------------------------------------   0
APR18-62        ------------------------------------------------------------   0
APR19-43        ------------------------------------------------------------   0
APR20-70        ------------------------------------------------------------   0
DEC10-249       ------------------------------------------------------------   0
DEC08-241       ------------------------------------------------------------   0
DEC06-81        ------------------------------------------------------------   0
DEC04-13db      GTATCTAAATGTGATTACATCCACCAAAGGAGAATATGTCTCTTCATAATCAATGTCAGG   1200
MAY17-11        ------------------------------------------------------------   0
MAY15-78        ------------------------------------------------------------   0
JUL05-102       ------------------------------------------------------------   0
JUL04-107       ------------------------------------------------------------   0
JUL03-55        ------------------------------------------------------------   0
JUL02-106       ------------------------------------------------------------   0
JUL01-85        ------------------------------------------------------------   0
DEC03-186       ------------------------------------------------------------   0
DEC07-98        ------------------------------------------------------------   0
APR17-291       ------------------------------------------------------------   0


DEC02-76        ------------------------------------------------------------   0
CLR03-38395     ------------------------------------------------------------   0
CLR01-43699     ------------------------------------------------------------   0
APR16-68        ------------------------------------------------------------   0
APR18-62        ------------------------------------------------------------   0
APR19-43        ------------------------------------------------------------   0
APR20-70        ------------------------------------------------------------   0
DEC10-249       ------------------------------------------------------------   0
DEC08-241       ------------------------------------------------------------   0
DEC06-81        ------------------------------------------------------------   0
DEC04-13db      CCTTTATGAAAATCCTTGTGTTAAAAGTCATGCTTTATATGTTATGGCTTCATTTTTCTC   1260
MAY17-11        ------------------------------------------------------------   0
MAY15-78        ------------------------------------------------------------   0
JUL05-102       ------------------------------------------------------------   0
JUL04-107       ------------------------------------------------------------   0
JUL03-55        ------------------------------------------------------------   0
JUL02-106       ------------------------------------------------------------   0
JUL01-85        ------------------------------------------------------------   0
DEC03-186       ------------------------------------------------------------   0
DEC07-98        ------------------------------------------------------------   0
APR17-291       ------------------------------------------------------------   0


DEC02-76        ------------------------------------------------------------   0
CLR03-38395     ------------------------------------------------------------   0
CLR01-43699     ------------------------------------------------------------   0
APR16-68        ------------------------------------------------------------   0
APR18-62        ------------------------------------------------------------   0
APR19-43        ------------------------------------------------------------   0
APR20-70        ------------------------------------------------------------   0
DEC10-249       ------------------------------------------------------------   0
DEC08-241       ------------------------------------------------------------   0
DEC06-81        ------------------------------------------------------------   0
DEC04-13db      ATTTTGTTTTCACAAAAATATCCATTTATATCCTACCGGCTTTACATATTTAGATGTTTG   1320
MAY17-11        ------------------------------------------------------------   0
MAY15-78        ------------------------------------------------------------   0
JUL05-102       ------------------------------------------------------------   0
JUL04-107       ------------------------------------------------------------   0
JUL03-55        ------------------------------------------------------------   0
```

Figure S4

```
JUL02-106         ------------------------------------------------------------   0
JUL01-85          ------------------------------------------------------------   0
DEC03-186         ------------------------------------------------------------   0
DEC07-98          ------------------------------------------------------------   0
APR17-291         ------------------------------------------------------------   0


DEC02-76          ------------------------------------------------------------   0
CLR03-38395       ------------------------------------------------------------   0
CLR01-43699       ------------------------------------------------------------   0
APR16-68          ------------------------------------------------------------   0
APR18-62          ------------------------------------------------------------   0
APR19-43          ------------------------------------------------------------   0
APR20-70          ------------------------------------------------------------   0
DEC10-249         ------------------------------------------------------------   0
DEC08-241         ------------------------------------------------------------   0
DEC06-81          ------------------------------------------------------------   0
DEC04-13db        GACTATAGGTCCAAAAACCTCATGTTTAGAAAGTGAATTTAATTCTGCTTGAATTGCGTC   1380
MAY17-11          ------------------------------------------------------------   0
MAY15-78          ------------------------------------------------------------   0
JUL05-102         ------------------------------------------------------------   0
JUL04-107         ------------------------------------------------------------   0
JUL03-55          ------------------------------------------------------------   0
JUL02-106         ------------------------------------------------------------   0
JUL01-85          ------------------------------------------------------------   0
DEC03-186         ------------------------------------------------------------   0
DEC07-98          ------------------------------------------------------------   0
APR17-291         ------------------------------------------------------------   0


DEC02-76          ------------------------------------------------------------   0
CLR03-38395       ------------------------------------------------------------   0
CLR01-43699       ------------------------------------------------------------   0
APR16-68          ------------------------------------------------------------   0
APR18-62          ------------------------------------------------------------   0
APR19-43          ------------------------------------------------------------   0
APR20-70          ------------------------------------------------------------   0
DEC10-249         ------------------------------------------------------------   0
DEC08-241         ------------------------------------------------------------   0
DEC06-81          ------------------------------------------------------------   0
DEC04-13db        TTTCCATTTTGGCCAATCTTTTTTATTTCTACATTCCTCAGTAAATTTAGGCTCAGGATC   1440
MAY17-11          ------------------------------------------------------------   0
MAY15-78          ------------------------------------------------------------   0
JUL05-102         ------------------------------------------------------------   0
JUL04-107         ------------------------------------------------------------   0
JUL03-55          ------------------------------------------------------------   0
JUL02-106         ------------------------------------------------------------   0
JUL01-85          ------------------------------------------------------------   0
DEC03-186         ------------------------------------------------------------   0
DEC07-98          ------------------------------------------------------------   0
APR17-291         ------------------------------------------------------------   0


DEC02-76          ------------------------------------------------------------   0
CLR03-38395       ------------------------------------------------------------   0
CLR01-43699       ------------------------------------------------------------   0
APR16-68          ------------------------------------------------------------   0
APR18-62          ------------------------------------------------------------   0
APR19-43          ------------------------------------------------------------   0
APR20-70          ------------------------------------------------------------   0
DEC10-249         ------------------------------------------------------------   0
DEC08-241         ------------------------------------------------------------   0
DEC06-81          ------------------------------------------------------------   0
```

```
DEC04-13db      CTCATTTTCTTTTGCTATTTCGATAGCAACATTATAAGTAAAATTGTTGTCGACAACTAT   1500
MAY17-11        ------------------------------------------------------------   0
MAY15-78        ------------------------------------------------------------   0
JUL05-102       ------------------------------------------------------------   0
JUL04-107       ------------------------------------------------------------   0
JUL03-55        ------------------------------------------------------------   0
JUL02-106       ------------------------------------------------------------   0
JUL01-85        ------------------------------------------------------------   0
DEC03-186       ------------------------------------------------------------   0
DEC07-98        ------------------------------------------------------------   0
APR17-291       ------------------------------------------------------------   0


DEC02-76        ------------------------------------------------------------   0
CLR03-38395     ------------------------------------------------------------   0
CLR01-43699     ------------------------------------------------------------   0
APR16-68        ------------------------------------------------------------   0
APR18-62        ------------------------------------------------------------   0
APR19-43        ------------------------------------------------------------   0
APR20-70        ------------------------------------------------------------   0
DEC10-249       ------------------------------------------------------------   0
DEC08-241       ------------------------------------------------------------   0
DEC06-81        ------------------------------------------------------------   0
DEC04-13db      ATTTTTTCGGTTTCATTTTTTTCCTGAAGTAACATAACTTATTCAGATTTCTTCGTTATC   1560
MAY17-11        ------------------------------------------------------------   0
MAY15-78        ------------------------------------------------------------   0
JUL05-102       ------------------------------------------------------------   0
JUL04-107       ------------------------------------------------------------   0
JUL03-55        ------------------------------------------------------------   0
JUL02-106       ------------------------------------------------------------   0
JUL01-85        ------------------------------------------------------------   0
DEC03-186       ------------------------------------------------------------   0
DEC07-98        ------------------------------------------------------------   0
APR17-291       ------------------------------------------------------------   0


DEC02-76        ------------------------------------------------------------   0
CLR03-38395     ------------------------------------------------------------   0
CLR01-43699     ------------------------------------------------------------   0
APR16-68        ------------------------------------------------------------   0
APR18-62        ------------------------------------------------------------   0
APR19-43        ------------------------------------------------------------   0
APR20-70        ------------------------------------------------------------   0
DEC10-249       ------------------------------------------------------------   0
DEC08-241       ------------------------------------------------------------   0
DEC06-81        ------------------------------------------------------------   0
DEC04-13db      ACCATTTTCAGGCACATGAACCTCTTTTGGGGTTTTTTGATTAGTTATATGTTTAGCCTT   1620
MAY17-11        ------------------------------------------------------------   0
MAY15-78        ------------------------------------------------------------   0
JUL05-102       ------------------------------------------------------------   0
JUL04-107       ------------------------------------------------------------   0
JUL03-55        ------------------------------------------------------------   0
JUL02-106       ------------------------------------------------------------   0
JUL01-85        ------------------------------------------------------------   0
DEC03-186       ------------------------------------------------------------   0
DEC07-98        ------------------------------------------------------------   0
APR17-291       ------------------------------------------------------------   0


DEC02-76        ------------------------------------------------------------   0
CLR03-38395     ------------------------------------------------------------   0
CLR01-43699     ------------------------------------------------------------   0
APR16-68        ------------------------------------------------------------   0
```

```
APR18-62        ------------------------------------------------------------   0
APR19-43        ------------------------------------------------------------   0
APR20-70        ------------------------------------------------------------   0
DEC10-249       ------------------------------------------------------------   0
DEC08-241       ------------------------------------------------------------   0
DEC06-81        ------------------------------------------------------------   0
DEC04-13db      TTCTTGGGCACTTGCCTCCACAATATTATCATCTTGAATAATTGTTCCTTTCCTTTTACG   1680
MAY17-11        ------------------------------------------------------------   0
MAY15-78        ------------------------------------------------------------   0
JUL05-102       ------------------------------------------------------------   0
JUL04-107       ------------------------------------------------------------   0
JUL03-55        ------------------------------------------------------------   0
JUL02-106       ------------------------------------------------------------   0
JUL01-85        ------------------------------------------------------------   0
DEC03-186       ------------------------------------------------------------   0
DEC07-98        ------------------------------------------------------------   0
APR17-291       ------------------------------------------------------------   0


DEC02-76        ------------------------------------------------------------   0
CLR03-38395     ------------------------------------------------------------   0
CLR01-43699     ------------------------------------------------------------   0
APR16-68        ------------------------------------------------------------   0
APR18-62        ------------------------------------------------------------   0
APR19-43        ------------------------------------------------------------   0
APR20-70        ------------------------------------------------------------   0
DEC10-249       ------------------------------------------------------------   0
DEC08-241       ------------------------------------------------------------   0
DEC06-81        ------------------------------------------------------------   0
DEC04-13db      AGTATTTTTATCTTTGGAACTGATTGGCCTTCCACGCTTCAGGCATGGATTACTTTCTTT   1740
MAY17-11        ------------------------------------------------------------   0
MAY15-78        ------------------------------------------------------------   0
JUL05-102       ------------------------------------------------------------   0
JUL04-107       ------------------------------------------------------------   0
JUL03-55        ------------------------------------------------------------   0
JUL02-106       ------------------------------------------------------------   0
JUL01-85        ------------------------------------------------------------   0
DEC03-186       ------------------------------------------------------------   0
DEC07-98        ------------------------------------------------------------   0
APR17-291       ------------------------------------------------------------   0


DEC02-76        ------------------------------------------------------------   0
CLR03-38395     ------------------------------------------------------------   0
CLR01-43699     ------------------------------------------------------------   0
APR16-68        ------------------------------------------------------------   0
APR18-62        ------------------------------------------------------------   0
APR19-43        ------------------------------------------------------------   0
APR20-70        ------------------------------------------------------------   0
DEC10-249       ------------------------------------------------------------   0
DEC08-241       ------------------------------------------------------------   0
DEC06-81        ------------------------------------------------------------   0
DEC04-13db      TACACAAACAATTTGCCCTATTAGGATATCAATCCGTATTGGAGCATTTTCAGCTGGTAT   1800
MAY17-11        ------------------------------------------------------------   0
MAY15-78        ------------------------------------------------------------   0
JUL05-102       ------------------------------------------------------------   0
JUL04-107       ------------------------------------------------------------   0
JUL03-55        ------------------------------------------------------------   0
JUL02-106       ------------------------------------------------------------   0
JUL01-85        ------------------------------------------------------------   0
DEC03-186       ------------------------------------------------------------   0
DEC07-98        ------------------------------------------------------------   0
APR17-291       ------------------------------------------------------------   0
```

```
DEC02-76        ------------------------------------------------------------   0
CLR03-38395     ------------------------------------------------------------   0
CLR01-43699     ------------------------------------------------------------   0
APR16-68        ------------------------------------------------------------   0
APR18-62        ------------------------------------------------------------   0
APR19-43        ------------------------------------------------------------   0
APR20-70        ------------------------------------------------------------   0
DEC10-249       ------------------------------------------------------------   0
DEC08-241       ------------------------------------------------------------   0
DEC06-81        ------------------------------------------------------------   0
DEC04-13db      GTGAGATTTTGTAATTCTTTTTAGGTCAGTAAATGAATCTGGCAATTGATTGATGATGTT   1860
MAY17-11        ------------------------------------------------------------   0
MAY15-78        ------------------------------------------------------------   0
JUL05-102       ------------------------------------------------------------   0
JUL04-107       ------------------------------------------------------------   0
JUL03-55        ------------------------------------------------------------   0
JUL02-106       ------------------------------------------------------------   0
JUL01-85        ------------------------------------------------------------   0
DEC03-186       ------------------------------------------------------------   0
DEC07-98        ------------------------------------------------------------   0
APR17-291       ------------------------------------------------------------   0


DEC02-76        ------------------------------------------------------------   0
CLR03-38395     ------------------------------------------------------------   0
CLR01-43699     ------------------------------------------------------------   0
APR16-68        ------------------------------------------------------------   0
APR18-62        ------------------------------------------------------------   0
APR19-43        ------------------------------------------------------------   0
APR20-70        ------------------------------------------------------------   0
DEC10-249       ------------------------------------------------------------   0
DEC08-241       ------------------------------------------------------------   0
DEC06-81        ------------------------------------------------------------   0
DEC04-13db      TTGCAAGTGTATGATCCTTTGAACTTCTTGTTCACATTGACTTGTGCGATGATCAAATTG   1920
MAY17-11        ------------------------------------------------------------   0
MAY15-78        ------------------------------------------------------------   0
JUL05-102       ------------------------------------------------------------   0
JUL04-107       ------------------------------------------------------------   0
JUL03-55        ------------------------------------------------------------   0
JUL02-106       ------------------------------------------------------------   0
JUL01-85        ------------------------------------------------------------   0
DEC03-186       ------------------------------------------------------------   0
DEC07-98        ------------------------------------------------------------   0
APR17-291       ------------------------------------------------------------   0


DEC02-76        ------------------------------------------------------------   0
CLR03-38395     ------------------------------------------------------------   0
CLR01-43699     ------------------------------------------------------------   0
APR16-68        ------------------------------------------------------------   0
APR18-62        ------------------------------------------------------------   0
APR19-43        ------------------------------------------------------------   0
APR20-70        ------------------------------------------------------------   0
DEC10-249       ------------------------------------------------------------   0
DEC08-241       ------------------------------------------------------------   0
DEC06-81        ------------------------------------------------------------   0
DEC04-13db      AGATAATGATGATCCATTCCATGTAATTTTTTTACCAATTGTATTTTCTCTCCCCCTAA   1980
MAY17-11        ------------------------------------------------------------   0
MAY15-78        ------------------------------------------------------------   0
JUL05-102       ------------------------------------------------------------   0
JUL04-107       ------------------------------------------------------------   0
```

```
JUL03-55         ------------------------------------------------------------   0
JUL02-106        ------------------------------------------------------------   0
JUL01-85         ------------------------------------------------------------   0
DEC03-186        ------------------------------------------------------------   0
DEC07-98         ------------------------------------------------------------   0
APR17-291        ------------------------------------------------------------   0


DEC02-76         ------------------------------------------------------------   0
CLR03-38395      ------------------------------------------------------------   0
CLR01-43699      ------------------------------------------------------------   0
APR16-68         ------------------------------------------------------------   0
APR18-62         ------------------------------------------------------------   0
APR19-43         ------------------------------------------------------------   0
APR20-70         ------------------------------------------------------------   0
DEC10-249        ------------------------------------------------------------   0
DEC08-241        ------------------------------------------------------------   0
DEC06-81         ------------------------------------------------------------   0
DEC04-13db       TATTGTAAATGTTGTTTCATCAAAATGACAATCAATAAATTGTGCAGTAAATAAACCTCC   2040
MAY17-11         ------------------------------------------------------------   0
MAY15-78         ------------------------------------------------------------   0
JUL05-102        ------------------------------------------------------------   0
JUL04-107        ------------------------------------------------------------   0
JUL03-55         ------------------------------------------------------------   0
JUL02-106        ------------------------------------------------------------   0
JUL01-85         ------------------------------------------------------------   0
DEC03-186        ------------------------------------------------------------   0
DEC07-98         ------------------------------------------------------------   0
APR17-291        ------------------------------------------------------------   0


DEC02-76         ------------------------------------------------------------   0
CLR03-38395      ------------------------------------------------------------   0
CLR01-43699      ------------------------------------------------------------   0
APR16-68         ------------------------------------------------------------   0
APR18-62         ------------------------------------------------------------   0
APR19-43         ------------------------------------------------------------   0
APR20-70         ------------------------------------------------------------   0
DEC10-249        ------------------------------------------------------------   0
DEC08-241        ------------------------------------------------------------   0
DEC06-81         ------------------------------------------------------------   0
DEC04-13db       AGTTAATGGTTCAACATATTTAATAATAAAAGTGGATGAAAATACATGAGAATTTTTCAT   2100
MAY17-11         ------------------------------------------------------------   0
MAY15-78         ------------------------------------------------------------   0
JUL05-102        ------------------------------------------------------------   0
JUL04-107        ------------------------------------------------------------   0
JUL03-55         ------------------------------------------------------------   0
JUL02-106        ------------------------------------------------------------   0
JUL01-85         ------------------------------------------------------------   0
DEC03-186        ------------------------------------------------------------   0
DEC07-98         ------------------------------------------------------------   0
APR17-291        ------------------------------------------------------------   0


DEC02-76         ------------------------------------------------------------   0
CLR03-38395      ------------------------------------------------------------   0
CLR01-43699      ------------------------------------------------------------   0
APR16-68         ------------------------------------------------------------   0
APR18-62         ------------------------------------------------------------   0
APR19-43         ------------------------------------------------------------   0
APR20-70         ------------------------------------------------------------   0
DEC10-249        ------------------------------------------------------------   0
DEC08-241        ------------------------------------------------------------   0
```

```
DEC06-81        ------------------------------------------------------------   0
DEC04-13db      TTTCTTATTTTTACTTATAATGTGCTATTTATATGCTCCTTTACATTCAATTAATAAATG   2160
MAY17-11        ------------------------------------------------------------   0
MAY15-78        ------------------------------------------------------------   0
JUL05-102       ------------------------------------------------------------   0
JUL04-107       ------------------------------------------------------------   0
JUL03-55        ------------------------------------------------------------   0
JUL02-106       ------------------------------------------------------------   0
JUL01-85        ------------------------------------------------------------   0
DEC03-186       ------------------------------------------------------------   0
DEC07-98        ------------------------------------------------------------   0
APR17-291       ------------------------------------------------------------   0


DEC02-76        ------------------------------------------------------------   0
CLR03-38395     ------------------------------------------------------------   0
CLR01-43699     ------------------------------------------------------------   0
APR16-68        ------------------------------------------------------------   0
APR18-62        ------------------------------------------------------------   0
APR19-43        ------------------------------------------------------------   0
APR20-70        ------------------------------------------------------------   0
DEC10-249       ------------------------------------------------------------   0
DEC08-241       ------------------------------------------------------------   0
DEC06-81        ------------------------------------------------------------   0
DEC04-13db      TATTACATTTAATGAACTAAGTTTTCTAATTAATCCATTTAATGATCTCCTAATTATAAA   2220
MAY17-11        ------------------------------------------------------------   0
MAY15-78        ------------------------------------------------------------   0
JUL05-102       ------------------------------------------------------------   0
JUL04-107       ------------------------------------------------------------   0
JUL03-55        ------------------------------------------------------------   0
JUL02-106       ------------------------------------------------------------   0
JUL01-85        ------------------------------------------------------------   0
DEC03-186       ------------------------------------------------------------   0
DEC07-98        ------------------------------------------------------------   0
APR17-291       ------------------------------------------------------------   0


DEC02-76        ------------------------------------------------------------   0
CLR03-38395     ------------------------------------------------------------   0
CLR01-43699     ------------------------------------------------------------   0
APR16-68        ------------------------------------------------------------   0
APR18-62        ------------------------------------------------------------   0
APR19-43        ------------------------------------------------------------   0
APR20-70        ------------------------------------------------------------   0
DEC10-249       ------------------------------------------------------------   0
DEC08-241       ------------------------------------------------------------   0
DEC06-81        ------------------------------------------------------------   0
DEC04-13db      TGAAGTAAAGAGTTTTATCTCATTACTTTAATATGCTTAAGGGGGTTATAGACATCCATT   2280
MAY17-11        ------------------------------------------------------------   0
MAY15-78        ------------------------------------------------------------   0
JUL05-102       ------------------------------------------------------------   0
JUL04-107       ------------------------------------------------------------   0
JUL03-55        ------------------------------------------------------------   0
JUL02-106       ------------------------------------------------------------   0
JUL01-85        ------------------------------------------------------------   0
DEC03-186       ------------------------------------------------------------   0
DEC07-98        ------------------------------------------------------------   0
APR17-291       ------------------------------------------------------------   0


DEC02-76        ------------------------------------------------------------   0
CLR03-38395     ------------------------------------------------------------   0
CLR01-43699     ------------------------------------------------------------   0
```

| | | |
|---|---|---|
| APR16-68 | ------------------------------------------------------------ | 0 |
| APR18-62 | ------------------------------------------------------------ | 0 |
| APR19-43 | ------------------------------------------------------------ | 0 |
| APR20-70 | ------------------------------------------------------------ | 0 |
| DEC10-249 | ------------------------------------------------------------ | 0 |
| DEC08-241 | ------------------------------------------------------------ | 0 |
| DEC06-81 | ------------------------------------------------------------ | 0 |
| DEC04-13db | TAATTATAAAAGGAGATTTATAACCAACATATATTCCCAACTGTGGTGGAGCAATTGGAA | 2340 |
| MAY17-11 | ------------------------------------------------------------ | 0 |
| MAY15-78 | ------------------------------------------------------------ | 0 |
| JUL05-102 | ------------------------------------------------------------ | 0 |
| JUL04-107 | ------------------------------------------------------------ | 0 |
| JUL03-55 | ------------------------------------------------------------ | 0 |
| JUL02-106 | ------------------------------------------------------------ | 0 |
| JUL01-85 | ------------------------------------------------------------ | 0 |
| DEC03-186 | ------------------------------------------------------------ | 0 |
| DEC07-98 | ------------------------------------------------------------ | 0 |
| APR17-291 | ------------------------------------------------------------ | 0 |

| | | |
|---|---|---|
| DEC02-76 | ------------------------------------------------------------ | 0 |
| CLR03-38395 | ------------------------------------------------------------ | 0 |
| CLR01-43699 | ------------------------------------------------------------ | 0 |
| APR16-68 | ------------------------------------------------------------ | 0 |
| APR18-62 | ------------------------------------------------------------ | 0 |
| APR19-43 | ------------------------------------------------------------ | 0 |
| APR20-70 | ------------------------------------------------------------ | 0 |
| DEC10-249 | ------------------------------------------------------------ | 0 |
| DEC08-241 | ------------------------------------------------------------ | 0 |
| DEC06-81 | ------------------------------------------------------------ | 0 |
| DEC04-13db | CATATACTGCGCATCCAAAAATTCTATGATGGGAAATATTAGCTCTTGACCAAAAGCCAA | 2400 |
| MAY17-11 | ------------------------------------------------------------ | 0 |
| MAY15-78 | ------------------------------------------------------------ | 0 |
| JUL05-102 | ------------------------------------------------------------ | 0 |
| JUL04-107 | ------------------------------------------------------------ | 0 |
| JUL03-55 | ------------------------------------------------------------ | 0 |
| JUL02-106 | ------------------------------------------------------------ | 0 |
| JUL01-85 | ------------------------------------------------------------ | 0 |
| DEC03-186 | ------------------------------------------------------------ | 0 |
| DEC07-98 | ------------------------------------------------------------ | 0 |
| APR17-291 | ------------------------------------------------------------ | 0 |

| | | |
|---|---|---|
| DEC02-76 | ------------------------------------------------------------ | 0 |
| CLR03-38395 | ------------------------------------------------------------ | 0 |
| CLR01-43699 | ------------------------------------------------------------ | 0 |
| APR16-68 | ------------------------------------------------------------ | 0 |
| APR18-62 | ------------------------------------------------------------ | 0 |
| APR19-43 | ------------------------------------------------------------ | 0 |
| APR20-70 | ------------------------------------------------------------ | 0 |
| DEC10-249 | ------------------------------------------------------------ | 0 |
| DEC08-241 | ------------------------------------------------------------ | 0 |
| DEC06-81 | ------------------------------------------------------------ | 0 |
| DEC04-13db | TTGTAATGGGGAGTACTTATTATAACTTGTTGACCTTAAGCATACAAGTACTGCTGCATG | 2460 |
| MAY17-11 | ------------------------------------------------------------ | 0 |
| MAY15-78 | ------------------------------------------------------------ | 0 |
| JUL05-102 | ------------------------------------------------------------ | 0 |
| JUL04-107 | ------------------------------------------------------------ | 0 |
| JUL03-55 | ------------------------------------------------------------ | 0 |
| JUL02-106 | ------------------------------------------------------------ | 0 |
| JUL01-85 | ------------------------------------------------------------ | 0 |
| DEC03-186 | ------------------------------------------------------------ | 0 |
| DEC07-98 | ------------------------------------------------------------ | 0 |

```
APR17-291      ------------------------------------------------------------   0


DEC02-76       ------------------------------------------------------------   0
CLR03-38395    ------------------------------------------------------------   0
CLR01-43699    ------------------------------------------------------------   0
APR16-68       ------------------------------------------------------------   0
APR18-62       ------------------------------------------------------------   0
APR19-43       ------------------------------------------------------------   0
APR20-70       ------------------------------------------------------------   0
DEC10-249      ------------------------------------------------------------   0
DEC08-241      ------------------------------------------------------------   0
DEC06-81       ------------------------------------------------------------   0
DEC04-13db     CAAAATAGTATATCCCCAAGCTGTAACAGGGAGTTTTGTTCTCATAAGCAATGGTCGAGC   2520
MAY17-11       ------------------------------------------------------------   0
MAY15-78       ------------------------------------------------------------   0
JUL05-102      ------------------------------------------------------------   0
JUL04-107      ------------------------------------------------------------   0
JUL03-55       ------------------------------------------------------------   0
JUL02-106      ------------------------------------------------------------   0
JUL01-85       ------------------------------------------------------------   0
DEC03-186      ------------------------------------------------------------   0
DEC07-98       ------------------------------------------------------------   0
APR17-291      ------------------------------------------------------------   0


DEC02-76       ------------------------------------------------------------   0
CLR03-38395    ------------------------------------------------------------   0
CLR01-43699    ------------------------------------------------------------   0
APR16-68       ------------------------------------------------------------   0
APR18-62       ------------------------------------------------------------   0
APR19-43       ------------------------------------------------------------   0
APR20-70       ------------------------------------------------------------   0
DEC10-249      ------------------------------------------------------------   0
DEC08-241      ------------------------------------------------------------   0
DEC06-81       ------------------------------------------------------------   0
DEC04-13db     AATTAGTAGGAGGCGTTTGATAAACGATTCAGCTAAACCATTTTGTGTGTAAATATGAGC   2580
MAY17-11       ------------------------------------------------------------   0
MAY15-78       ------------------------------------------------------------   0
JUL05-102      ------------------------------------------------------------   0
JUL04-107      ------------------------------------------------------------   0
JUL03-55       ------------------------------------------------------------   0
JUL02-106      ------------------------------------------------------------   0
JUL01-85       ------------------------------------------------------------   0
DEC03-186      ------------------------------------------------------------   0
DEC07-98       ------------------------------------------------------------   0
APR17-291      ------------------------------------------------------------   0


DEC02-76       ------------------------------------------------------------   0
CLR03-38395    ------------------------------------------------------------   0
CLR01-43699    ------------------------------------------------------------   0
APR16-68       ------------------------------------------------------------   0
APR18-62       ------------------------------------------------------------   0
APR19-43       ------------------------------------------------------------   0
APR20-70       ------------------------------------------------------------   0
DEC10-249      ------------------------------------------------------------   0
DEC08-241      ------------------------------------------------------------   0
DEC06-81       ------------------------------------------------------------   0
DEC04-13db     TATAGGATGTTCAACTTTTATCCCAATTAACATACAATAATCATTAAAAGCTTGGGATGT   2640
MAY17-11       ------------------------------------------------------------   0
MAY15-78       ------------------------------------------------------------   0
JUL05-102      ------------------------------------------------------------   0
```

```
JUL04-107     ------------------------------------------------------------  0
JUL03-55      ------------------------------------------------------------  0
JUL02-106     ------------------------------------------------------------  0
JUL01-85      ------------------------------------------------------------  0
DEC03-186     ------------------------------------------------------------  0
DEC07-98      ------------------------------------------------------------  0
APR17-291     ------------------------------------------------------------  0


DEC02-76      ------------------------------------------------------------  0
CLR03-38395   ------------------------------------------------------------  0
CLR01-43699   ------------------------------------------------------------  0
APR16-68      ------------------------------------------------------------  0
APR18-62      ------------------------------------------------------------  0
APR19-43      ------------------------------------------------------------  0
APR20-70      ------------------------------------------------------------  0
DEC10-249     ------------------------------------------------------------  0
DEC08-241     ------------------------------------------------------------  0
DEC06-81      ------------------------------------------------------------  0
DEC04-13db    AAACTCACCAGCATTATCAAGATGAATAGTTTTGATTGCATAATCTGGAAATTATGCTCT  2700
MAY17-11      ------------------------------------------------------------  0
MAY15-78      ------------------------------------------------------------  0
JUL05-102     ------------------------------------------------------------  0
JUL04-107     ------------------------------------------------------------  0
JUL03-55      ------------------------------------------------------------  0
JUL02-106     ------------------------------------------------------------  0
JUL01-85      ------------------------------------------------------------  0
DEC03-186     ------------------------------------------------------------  0
DEC07-98      ------------------------------------------------------------  0
APR17-291     ------------------------------------------------------------  0


DEC02-76      ------------------------------------------------------------  0
CLR03-38395   ------------------------------------------------------------  0
CLR01-43699   ------------------------------------------------------------  0
APR16-68      ------------------------------------------------------------  0
APR18-62      ------------------------------------------------------------  0
APR19-43      ------------------------------------------------------------  0
APR20-70      ------------------------------------------------------------  0
DEC10-249     ------------------------------------------------------------  0
DEC08-241     ------------------------------------------------------------  0
DEC06-81      ------------------------------------------------------------  0
DEC04-13db    TAATCGAATTATTTGAGTGAGTAGTCTCGCAAATGTCAGGTTGCGAGTCGATAATAAAGA  2760
MAY17-11      ------------------------------------------------------------  0
MAY15-78      ------------------------------------------------------------  0
JUL05-102     ------------------------------------------------------------  0
JUL04-107     ------------------------------------------------------------  0
JUL03-55      ------------------------------------------------------------  0
JUL02-106     ------------------------------------------------------------  0
JUL01-85      ------------------------------------------------------------  0
DEC03-186     ------------------------------------------------------------  0
DEC07-98      ------------------------------------------------------------  0
APR17-291     ------------------------------------------------------------  0


DEC02-76      ------------------------------------------------------------  0
CLR03-38395   ------------------------------------------------------------  0
CLR01-43699   ------------------------------------------------------------  0
APR16-68      ------------------------------------------------------------  0
APR18-62      ------------------------------------------------------------  0
APR19-43      ------------------------------------------------------------  0
APR20-70      ------------------------------------------------------------  0
DEC10-249     ------------------------------------------------------------  0
```

```
DEC08-241       ------------------------------------------------------------   0
DEC06-81        ------------------------------------------------------------   0
DEC04-13db      CACACGTGACCACCTGCTAGATGCATCTATTAATACTATAAAATATTTGAATGGTCCATA   2820
MAY17-11        ------------------------------------------------------------   0
MAY15-78        ------------------------------------------------------------   0
JUL05-102       ------------------------------------------------------------   0
JUL04-107       ------------------------------------------------------------   0
JUL03-55        ------------------------------------------------------------   0
JUL02-106       ------------------------------------------------------------   0
JUL01-85        ------------------------------------------------------------   0
DEC03-186       ------------------------------------------------------------   0
DEC07-98        ------------------------------------------------------------   0
APR17-291       ------------------------------------------------------------   0


DEC02-76        ------------------------------------------------------------   0
CLR03-38395     ------------------------------------------------------------   0
CLR01-43699     ------------------------------------------------------------   0
APR16-68        ------------------------------------------------------------   0
APR18-62        ------------------------------------------------------------   0
APR19-43        ------------------------------------------------------------   0
APR20-70        ------------------------------------------------------------   0
DEC10-249       ------------------------------------------------------------   0
DEC08-241       ------------------------------------------------------------   0
DEC06-81        ------------------------------------------------------------   0
DEC04-13db      TGGTGGATGAATGGGCCCACATATATCACCTTGAATACGTTCCAAAAATGCAGGAGATTC   2880
MAY17-11        ------------------------------------------------------------   0
MAY15-78        ------------------------------------------------------------   0
JUL05-102       ------------------------------------------------------------   0
JUL04-107       ------------------------------------------------------------   0
JUL03-55        ------------------------------------------------------------   0
JUL02-106       ------------------------------------------------------------   0
JUL01-85        ------------------------------------------------------------   0
DEC03-186       ------------------------------------------------------------   0
DEC07-98        ------------------------------------------------------------   0
APR17-291       ------------------------------------------------------------   0


DEC02-76        ------------------------------------------------------------   0
CLR03-38395     ------------------------------------------------------------   0
CLR01-43699     ------------------------------------------------------------   0
APR16-68        ------------------------------------------------------------   0
APR18-62        ------------------------------------------------------------   0
APR19-43        ------------------------------------------------------------   0
APR20-70        ------------------------------------------------------------   0
DEC10-249       ------------------------------------------------------------   0
DEC08-241       ------------------------------------------------------------   0
DEC06-81        ------------------------------------------------------------   0
DEC04-13db      AGTCCCAACTTTTACTGGTGATGGTCTAATAATCAATTTTCCTTGAGAATAAGCGACATA   2940
MAY17-11        ------------------------------------------------------------   0
MAY15-78        ------------------------------------------------------------   0
JUL05-102       ------------------------------------------------------------   0
JUL04-107       ------------------------------------------------------------   0
JUL03-55        ------------------------------------------------------------   0
JUL02-106       ------------------------------------------------------------   0
JUL01-85        ------------------------------------------------------------   0
DEC03-186       ------------------------------------------------------------   0
DEC07-98        ------------------------------------------------------------   0
APR17-291       ------------------------------------------------------------   0


DEC02-76        ------------------------------------------------------------   0
CLR03-38395     ------------------------------------------------------------   0
```

```
CLR01-43699      ------------------------------------------------------------   0
APR16-68         ------------------------------------------------------------   0
APR18-62         ------------------------------------------------------------   0
APR19-43         ------------------------------------------------------------   0
APR20-70         ------------------------------------------------------------   0
DEC10-249        ------------------------------------------------------------   0
DEC08-241        ------------------------------------------------------------   0
DEC06-81         ------------------------------------------------------------   0
DEC04-13db       AGATAAATCTTTGAATTCAAGAATCTTTTGGTTCTTTAATGAGTGTCCAATTGAATTCTT   3000
MAY17-11         ------------------------------------------------------------   0
MAY15-78         ------------------------------------------------------------   0
JUL05-102        ------------------------------------------------------------   0
JUL04-107        ------------------------------------------------------------   0
JUL03-55         ------------------------------------------------------------   0
JUL02-106        ------------------------------------------------------------   0
JUL01-85         ------------------------------------------------------------   0
DEC03-186        ------------------------------------------------------------   0
DEC07-98         ------------------------------------------------------------   0
APR17-291        ------------------------------------------------------------   0


DEC02-76         ------------------------------------------------------------   0
CLR03-38395      ------------------------------------------------------------   0
CLR01-43699      ------------------------------------------------------------   0
APR16-68         ------------------------------------------------------------   0
APR18-62         ------------------------------------------------------------   0
APR19-43         ------------------------------------------------------------   0
APR20-70         ------------------------------------------------------------   0
DEC10-249        ------------------------------------------------------------   0
DEC08-241        ------------------------------------------------------------   0
DEC06-81         ------------------------------------------------------------   0
DEC04-13db       GATGATTCTTCGCATTATAATAAATCCGGGATGGCCTAATCGGTTATGCCAAATAGTAAA   3060
MAY17-11         ------------------------------------------------------------   0
MAY15-78         ------------------------------------------------------------   0
JUL05-102        ------------------------------------------------------------   0
JUL04-107        ------------------------------------------------------------   0
JUL03-55         ------------------------------------------------------------   0
JUL02-106        ------------------------------------------------------------   0
JUL01-85         ------------------------------------------------------------   0
DEC03-186        ------------------------------------------------------------   0
DEC07-98         ------------------------------------------------------------   0
APR17-291        ------------------------------------------------------------   0


DEC02-76         ------------------------------------------------------------   0
CLR03-38395      ------------------------------------------------------------   0
CLR01-43699      ------------------------------------------------------------   0
APR16-68         ------------------------------------------------------------   0
APR18-62         ------------------------------------------------------------   0
APR19-43         ------------------------------------------------------------   0
APR20-70         ------------------------------------------------------------   0
DEC10-249        ------------------------------------------------------------   0
DEC08-241        ------------------------------------------------------------   0
DEC06-81         ------------------------------------------------------------   0
DEC04-13db       AGTATGTGGTTATACAAACTTCTAGTTTGTAGTAACATGTGACTCAATTGCACTAATATG   3120
MAY17-11         ------------------------------------------------------------   0
MAY15-78         ------------------------------------------------------------   0
JUL05-102        ------------------------------------------------------------   0
JUL04-107        ------------------------------------------------------------   0
JUL03-55         ------------------------------------------------------------   0
JUL02-106        ------------------------------------------------------------   0
JUL01-85         ------------------------------------------------------------   0
DEC03-186        ------------------------------------------------------------   0
```

```
DEC07-98          ------------------------------------------------------------   0
APR17-291         ------------------------------------------------------------   0


DEC02-76          ------------------------------------------------------------   0
CLR03-38395       ------------------------------------------------------------   0
CLR01-43699       ------------------------------------------------------------   0
APR16-68          ------------------------------------------------------------   0
APR18-62          ------------------------------------------------------------   0
APR19-43          ------------------------------------------------------------   0
APR20-70          ------------------------------------------------------------   0
DEC10-249         ------------------------------------------------------------   0
DEC08-241         ------------------------------------------------------------   0
DEC06-81          ------------------------------------------------------------   0
DEC04-13db        TGTATAATATAAACCTGATGAAAAAGCAGCTAACCTTTCCAAAACATATTTCTTTCCACG   3180
MAY17-11          ------------------------------------------------------------   0
MAY15-78          ------------------------------------------------------------   0
JUL05-102         ------------------------------------------------------------   0
JUL04-107         ------------------------------------------------------------   0
JUL03-55          ------------------------------------------------------------   0
JUL02-106         ------------------------------------------------------------   0
JUL01-85          ------------------------------------------------------------   0
DEC03-186         ------------------------------------------------------------   0
DEC07-98          ------------------------------------------------------------   0
APR17-291         ------------------------------------------------------------   0


DEC02-76          ------------------------------------------------------------   0
CLR03-38395       ------------------------------------------------------------   0
CLR01-43699       ------------------------------------------------------------   0
APR16-68          ------------------------------------------------------------   0
APR18-62          ------------------------------------------------------------   0
APR19-43          ------------------------------------------------------------   0
APR20-70          ------------------------------------------------------------   0
DEC10-249         ------------------------------------------------------------   0
DEC08-241         ------------------------------------------------------------   0
DEC06-81          ------------------------------------------------------------   0
DEC04-13db        TTCAACATTTGTAATATATAGATATTCAATATTTTTCTCATTCATAGTCTCAATATGATG   3240
MAY17-11          ------------------------------------------------------------   0
MAY15-78          ------------------------------------------------------------   0
JUL05-102         ------------------------------------------------------------   0
JUL04-107         ------------------------------------------------------------   0
JUL03-55          ------------------------------------------------------------   0
JUL02-106         ------------------------------------------------------------   0
JUL01-85          ------------------------------------------------------------   0
DEC03-186         ------------------------------------------------------------   0
DEC07-98          ------------------------------------------------------------   0
APR17-291         ------------------------------------------------------------   0


DEC02-76          ------------------------------------------------------------   0
CLR03-38395       ------------------------------------------------------------   0
CLR01-43699       ------------------------------------------------------------   0
APR16-68          ------------------------------------------------------------   0
APR18-62          ------------------------------------------------------------   0
APR19-43          ------------------------------------------------------------   0
APR20-70          ------------------------------------------------------------   0
DEC10-249         ------------------------------------------------------------   0
DEC08-241         ------------------------------------------------------------   0
DEC06-81          ------------------------------------------------------------   0
DEC04-13db        TCCATTAAGATGAATATCTTTAAAACTCAATAAATTTCTTTGAAACTTAATGGAATATAA   3300
MAY17-11          ------------------------------------------------------------   0
MAY15-78          ------------------------------------------------------------   0
```

```
JUL05-102       ------------------------------------------------------------   0
JUL04-107       ------------------------------------------------------------   0
JUL03-55        ------------------------------------------------------------   0
JUL02-106       ------------------------------------------------------------   0
JUL01-85        ------------------------------------------------------------   0
DEC03-186       ------------------------------------------------------------   0
DEC07-98        ------------------------------------------------------------   0
APR17-291       ------------------------------------------------------------   0


DEC02-76        ------------------------------------------------------------   0
CLR03-38395     ------------------------------------------------------------   0
CLR01-43699     ------------------------------------------------------------   0
APR16-68        ------------------------------------------------------------   0
APR18-62        ------------------------------------------------------------   0
APR19-43        ------------------------------------------------------------   0
APR20-70        ------------------------------------------------------------   0
DEC10-249       ------------------------------------------------------------   0
DEC08-241       ------------------------------------------------------------   0
DEC06-81        ------------------------------------------------------------   0
DEC04-13db      AGCATCATCATGATAAATTTTGTACCTTTAGGTAATAATATAATAGCTCTTCCAGAGCCT   3360
MAY17-11        ------------------------------------------------------------   0
MAY15-78        ------------------------------------------------------------   0
JUL05-102       ------------------------------------------------------------   0
JUL04-107       ------------------------------------------------------------   0
JUL03-55        ------------------------------------------------------------   0
JUL02-106       ------------------------------------------------------------   0
JUL01-85        ------------------------------------------------------------   0
DEC03-186       ------------------------------------------------------------   0
DEC07-98        ------------------------------------------------------------   0
APR17-291       ------------------------------------------------------------   0


DEC02-76        ------------------------------------------------------------   0
CLR03-38395     ------------------------------------------------------------   0
CLR01-43699     ------------------------------------------------------------   0
APR16-68        ------------------------------------------------------------   0
APR18-62        ------------------------------------------------------------   0
APR19-43        ------------------------------------------------------------   0
APR20-70        ------------------------------------------------------------   0
DEC10-249       ------------------------------------------------------------   0
DEC08-241       ------------------------------------------------------------   0
DEC06-81        ------------------------------------------------------------   0
DEC04-13db      CCAATAAGTTTTGAACTTCCCGATATTATATTAACATGGGCATTACTTGTTGTCAAATGA   3420
MAY17-11        ------------------------------------------------------------   0
MAY15-78        ------------------------------------------------------------   0
JUL05-102       ------------------------------------------------------------   0
JUL04-107       ------------------------------------------------------------   0
JUL03-55        ------------------------------------------------------------   0
JUL02-106       ------------------------------------------------------------   0
JUL01-85        ------------------------------------------------------------   0
DEC03-186       ------------------------------------------------------------   0
DEC07-98        ------------------------------------------------------------   0
APR17-291       ------------------------------------------------------------   0


DEC02-76        ------------------------------------------------------------   0
CLR03-38395     ------------------------------------------------------------   0
CLR01-43699     ------------------------------------------------------------   0
APR16-68        ------------------------------------------------------------   0
APR18-62        ------------------------------------------------------------   0
APR19-43        ------------------------------------------------------------   0
APR20-70        ------------------------------------------------------------   0
```

```
DEC10-249       ------------------------------------------------------------   0
DEC08-241       ------------------------------------------------------------   0
DEC06-81        ------------------------------------------------------------   0
DEC04-13db      GAAAAATATTTTTTATCTTTGAGTATCGTATGTGTTGTAGCACTATCTGCAACACACATG   3480
MAY17-11        ------------------------------------------------------------   0
MAY15-78        ------------------------------------------------------------   0
JUL05-102       ------------------------------------------------------------   0
JUL04-107       ------------------------------------------------------------   0
JUL03-55        ------------------------------------------------------------   0
JUL02-106       ------------------------------------------------------------   0
JUL01-85        ------------------------------------------------------------   0
DEC03-186       ------------------------------------------------------------   0
DEC07-98        ------------------------------------------------------------   0
APR17-291       ------------------------------------------------------------   0


DEC02-76        ------------------------------------------------------------   0
CLR03-38395     ------------------------------------------------------------   0
CLR01-43699     ------------------------------------------------------------   0
APR16-68        ------------------------------------------------------------   0
APR18-62        ------------------------------------------------------------   0
APR19-43        ------------------------------------------------------------   0
APR20-70        ------------------------------------------------------------   0
DEC10-249       ------------------------------------------------------------   0
DEC08-241       ------------------------------------------------------------   0
DEC06-81        ------------------------------------------------------------   0
DEC04-13db      TCTCCATTGATTTTGGGTCCATCGAAATTTTGTTGAATATTCATATTATTCATAAAAACA   3540
MAY17-11        ------------------------------------------------------------   0
MAY15-78        ------------------------------------------------------------   0
JUL05-102       ------------------------------------------------------------   0
JUL04-107       ------------------------------------------------------------   0
JUL03-55        ------------------------------------------------------------   0
JUL02-106       ------------------------------------------------------------   0
JUL01-85        ------------------------------------------------------------   0
DEC03-186       ------------------------------------------------------------   0
DEC07-98        ------------------------------------------------------------   0
APR17-291       ------------------------------------------------------------   0


DEC02-76        ------------------------------------------------------------   0
CLR03-38395     ------------------------------------------------------------   0
CLR01-43699     ------------------------------------------------------------   0
APR16-68        ------------------------------------------------------------   0
APR18-62        ------------------------------------------------------------   0
APR19-43        ------------------------------------------------------------   0
APR20-70        ------------------------------------------------------------   0
DEC10-249       ------------------------------------------------------------   0
DEC08-241       ------------------------------------------------------------   0
DEC06-81        ------------------------------------------------------------   0
DEC04-13db      AACAAAATATAATATGAGAAACATTGCAAATATTTATTAAATATATAAATTATTCTTTAT   3600
MAY17-11        ------------------------------------------------------------   0
MAY15-78        ------------------------------------------------------------   0
JUL05-102       ------------------------------------------------------------   0
JUL04-107       ------------------------------------------------------------   0
JUL03-55        ------------------------------------------------------------   0
JUL02-106       ------------------------------------------------------------   0
JUL01-85        ------------------------------------------------------------   0
DEC03-186       ------------------------------------------------------------   0
DEC07-98        ------------------------------------------------------------   0
APR17-291       ------------------------------------------------------------   0


DEC02-76        ------------------------------------------------------------   0
```

```
CLR03-38395      ------------------------------------------------------------   0
CLR01-43699      ------------------------------------------------------------   0
APR16-68         ------------------------------------------------------------   0
APR18-62         ------------------------------------------------------------   0
APR19-43         ------------------------------------------------------------   0
APR20-70         ------------------------------------------------------------   0
DEC10-249        ------------------------------------------------------------   0
DEC08-241        ------------------------------------------------------------   0
DEC06-81         ------------------------------------------------------------   0
DEC04-13db       GCAAGAAAGTAAAACATACTTAAAACAACATAAAAATGTCAAAATAAAATCAATTTTTCT   3660
MAY17-11         ------------------------------------------------------------   0
MAY15-78         ------------------------------------------------------------   0
JUL05-102        ------------------------------------------------------------   0
JUL04-107        ------------------------------------------------------------   0
JUL03-55         ------------------------------------------------------------   0
JUL02-106        ------------------------------------------------------------   0
JUL01-85         ------------------------------------------------------------   0
DEC03-186        ------------------------------------------------------------   0
DEC07-98         ------------------------------------------------------------   0
APR17-291        ------------------------------------------------------------   0


DEC02-76         ------------------------------------------------------------   0
CLR03-38395      ------------------------------------------------------------   0
CLR01-43699      ------------------------------------------------------------   0
APR16-68         ------------------------------------------------------------   0
APR18-62         ------------------------------------------------------------   0
APR19-43         ------------------------------------------------------------   0
APR20-70         ------------------------------------------------------------   0
DEC10-249        ------------------------------------------------------------   0
DEC08-241        ------------------------------------------------------------   0
DEC06-81         ------------------------------------------------------------   0
DEC04-13db       AATGATTCTCAAAGAAATCTGCCACATCTAGGTGAGTTATATTATTAAGGTCATTAAATT   3720
MAY17-11         ------------------------------------------------------------   0
MAY15-78         ------------------------------------------------------------   0
JUL05-102        ------------------------------------------------------------   0
JUL04-107        ------------------------------------------------------------   0
JUL03-55         ------------------------------------------------------------   0
JUL02-106        ------------------------------------------------------------   0
JUL01-85         ------------------------------------------------------------   0
DEC03-186        ------------------------------------------------------------   0
DEC07-98         ------------------------------------------------------------   0
APR17-291        ------------------------------------------------------------   0


DEC02-76         ------------------------------------------------------------   0
CLR03-38395      ------------------------------------------------------------   0
CLR01-43699      ------------------------------------------------------------   0
APR16-68         ------------------------------------------------------------   0
APR18-62         ------------------------------------------------------------   0
APR19-43         ------------------------------------------------------------   0
APR20-70         ------------------------------------------------------------   0
DEC10-249        ------------------------------------------------------------   0
DEC08-241        ------------------------------------------------------------   0
DEC06-81         ------------------------------------------------------------   0
DEC04-13db       TATCACCCTCGTAGGCATGGTTAGTTTTAGTATTATAGTGAATATCTTCATCTTTTGCCT   3780
MAY17-11         ------------------------------------------------------------   0
MAY15-78         ------------------------------------------------------------   0
JUL05-102        ------------------------------------------------------------   0
JUL04-107        ------------------------------------------------------------   0
JUL03-55         ------------------------------------------------------------   0
JUL02-106        ------------------------------------------------------------   0
JUL01-85         ------------------------------------------------------------   0
```

```
DEC03-186         ------------------------------------------------------------   0
DEC07-98          ------------------------------------------------------------   0
APR17-291         ------------------------------------------------------------   0


DEC02-76          ------------------------------------------------------------   0
CLR03-38395       ------------------------------------------------------------   0
CLR01-43699       ------------------------------------------------------------   0
APR16-68          ------------------------------------------------------------   0
APR18-62          ------------------------------------------------------------   0
APR19-43          ------------------------------------------------------------   0
APR20-70          ------------------------------------------------------------   0
DEC10-249         ------------------------------------------------------------   0
DEC08-241         ------------------------------------------------------------   0
DEC06-81          ------------------------------------------------------------   0
DEC04-13db        CCATTTTATCATTTTGAGATATAAAATTTGTCTCCATATACATTTCCTTCTTTTTAATGG   3840
MAY17-11          ------------------------------------------------------------   0
MAY15-78          ------------------------------------------------------------   0
JUL05-102         ------------------------------------------------------------   0
JUL04-107         ------------------------------------------------------------   0
JUL03-55          ------------------------------------------------------------   0
JUL02-106         ------------------------------------------------------------   0
JUL01-85          ------------------------------------------------------------   0
DEC03-186         ------------------------------------------------------------   0
DEC07-98          ------------------------------------------------------------   0
APR17-291         ------------------------------------------------------------   0


DEC02-76          ------------------------------------------------------------   0
CLR03-38395       ------------------------------------------------------------   0
CLR01-43699       ------------------------------------------------------------   0
APR16-68          ------------------------------------------------------------   0
APR18-62          ------------------------------------------------------------   0
APR19-43          ------------------------------------------------------------   0
APR20-70          ------------------------------------------------------------   0
DEC10-249         ------------------------------------------------------------   0
DEC08-241         ------------------------------------------------------------   0
DEC06-81          ------------------------------------------------------------   0
DEC04-13db        ATGCTTGATAAAGTTTCACTAAATACTCAGACGTACGATAGGTATATGACCAATGTCCCT   3900
MAY17-11          ------------------------------------------------------------   0
MAY15-78          ------------------------------------------------------------   0
JUL05-102         ------------------------------------------------------------   0
JUL04-107         ------------------------------------------------------------   0
JUL03-55          ------------------------------------------------------------   0
JUL02-106         ------------------------------------------------------------   0
JUL01-85          ------------------------------------------------------------   0
DEC03-186         ------------------------------------------------------------   0
DEC07-98          ------------------------------------------------------------   0
APR17-291         ------------------------------------------------------------   0


DEC02-76          ------------------------------------------------------------   0
CLR03-38395       ------------------------------------------------------------   0
CLR01-43699       ------------------------------------------------------------   0
APR16-68          ------------------------------------------------------------   0
APR18-62          ------------------------------------------------------------   0
APR19-43          ------------------------------------------------------------   0
APR20-70          ------------------------------------------------------------   0
DEC10-249         ------------------------------------------------------------   0
DEC08-241         ------------------------------------------------------------   0
DEC06-81          ------------------------------------------------------------   0
DEC04-13db        TCATACCACATCGGTAGCATATATTCTCAATAATCTTTGAAGGATTATTTTGACCATTTT   3960
MAY17-11          ------------------------------------------------------------   0
```

```
MAY15-78          ------------------------------------------------------------    0
JUL05-102         ------------------------------------------------------------    0
JUL04-107         ------------------------------------------------------------    0
JUL03-55          ------------------------------------------------------------    0
JUL02-106         ------------------------------------------------------------    0
JUL01-85          ------------------------------------------------------------    0
DEC03-186         ------------------------------------------------------------    0
DEC07-98          ------------------------------------------------------------    0
APR17-291         ------------------------------------------------------------    0


DEC02-76          ------------------------------------------------------------    0
CLR03-38395       ------------------------------------------------------------    0
CLR01-43699       ------------------------------------------------------------    0
APR16-68          ------------------------------------------------------------    0
APR18-62          ------------------------------------------------------------    0
APR19-43          ------------------------------------------------------------    0
APR20-70          ------------------------------------------------------------    0
DEC10-249         ------------------------------------------------------------    0
DEC08-241         ------------------------------------------------------------    0
DEC06-81          ------------------------------------------------------------    0
DEC04-13db        TTTCTTGTATTTCATTTTTATTCTTTTTCTGGTGGTTAGAAGTATCAGTCTTATGGCCAC    4020
MAY17-11          ------------------------------------------------------------    0
MAY15-78          ------------------------------------------------------------    0
JUL05-102         ------------------------------------------------------------    0
JUL04-107         ------------------------------------------------------------    0
JUL03-55          ------------------------------------------------------------    0
JUL02-106         ------------------------------------------------------------    0
JUL01-85          ------------------------------------------------------------    0
DEC03-186         ------------------------------------------------------------    0
DEC07-98          ------------------------------------------------------------    0
APR17-291         ------------------------------------------------------------    0


DEC02-76          ------------------------------------------------------------    0
CLR03-38395       ------------------------------------------------------------    0
CLR01-43699       ------------------------------------------------------------    0
APR16-68          ------------------------------------------------------------    0
APR18-62          ------------------------------------------------------------    0
APR19-43          ------------------------------------------------------------    0
APR20-70          ------------------------------------------------------------    0
DEC10-249         ------------------------------------------------------------    0
DEC08-241         ------------------------------------------------------------    0
DEC06-81          ------------------------------------------------------------    0
DEC04-13db        CATGATAACGATTACTAATGCGTCCTTGACCACATCCCCCACTACGTCCACGACCACAGT    4080
MAY17-11          ------------------------------------------------------------    0
MAY15-78          ------------------------------------------------------------    0
JUL05-102         ------------------------------------------------------------    0
JUL04-107         ------------------------------------------------------------    0
JUL03-55          ------------------------------------------------------------    0
JUL02-106         ------------------------------------------------------------    0
JUL01-85          ------------------------------------------------------------    0
DEC03-186         ------------------------------------------------------------    0
DEC07-98          ------------------------------------------------------------    0
APR17-291         ------------------------------------------------------------    0


DEC02-76          ------------------------------------------------------------    0
CLR03-38395       ------------------------------------------------------------    0
CLR01-43699       ------------------------------------------------------------    0
APR16-68          ------------------------------------------------------------    0
APR18-62          ------------------------------------------------------------    0
APR19-43          ------------------------------------------------------------    0
```

```
APR20-70        ------------------------------------------------------------   0
DEC10-249       ------------------------------------------------------------   0
DEC08-241       ------------------------------------------------------------   0
DEC06-81        ------------------------------------------------------------   0
DEC04-13db      CGCGACATCTATATTTTCTATTTTCACAATTATTGTGTATTGCTACATTCACTTCAGGGA   4140
MAY17-11        ------------------------------------------------------------   0
MAY15-78        ------------------------------------------------------------   0
JUL05-102       ------------------------------------------------------------   0
JUL04-107       ------------------------------------------------------------   0
JUL03-55        ------------------------------------------------------------   0
JUL02-106       ------------------------------------------------------------   0
JUL01-85        ------------------------------------------------------------   0
DEC03-186       ------------------------------------------------------------   0
DEC07-98        ------------------------------------------------------------   0
APR17-291       ------------------------------------------------------------   0


DEC02-76        ------------------------------------------------------------   0
CLR03-38395     ------------------------------------------------------------   0
CLR01-43699     ------------------------------------------------------------   0
APR16-68        ------------------------------------------------------------   0
APR18-62        ------------------------------------------------------------   0
APR19-43        ------------------------------------------------------------   0
APR20-70        ------------------------------------------------------------   0
DEC10-249       ------------------------------------------------------------   0
DEC08-241       ------------------------------------------------------------   0
DEC06-81        ------------------------------------------------------------   0
DEC04-13db      ATGGTACAGAACTAGTGGGATGAATTCCATGATTTTTCATCAATAGCTCATTATTTTGTT   4200
MAY17-11        ------------------------------------------------------------   0
MAY15-78        ------------------------------------------------------------   0
JUL05-102       ------------------------------------------------------------   0
JUL04-107       ------------------------------------------------------------   0
JUL03-55        ------------------------------------------------------------   0
JUL02-106       ------------------------------------------------------------   0
JUL01-85        ------------------------------------------------------------   0
DEC03-186       ------------------------------------------------------------   0
DEC07-98        ------------------------------------------------------------   0
APR17-291       ------------------------------------------------------------   0


DEC02-76        ------------------------------------------------------------   0
CLR03-38395     ------------------------------------------------------------   0
CLR01-43699     ------------------------------------------------------------   0
APR16-68        ------------------------------------------------------------   0
APR18-62        ------------------------------------------------------------   0
APR19-43        ------------------------------------------------------------   0
APR20-70        ------------------------------------------------------------   0
DEC10-249       ------------------------------------------------------------   0
DEC08-241       ------------------------------------------------------------   0
DEC06-81        ------------------------------------------------------------   0
DEC04-13db      CAGCCACCAAAAAGCATGAAATCAATTCAGAATACCTTTTAAAACCTTTTTCACGGTATT   4260
MAY17-11        ------------------------------------------------------------   0
MAY15-78        ------------------------------------------------------------   0
JUL05-102       ------------------------------------------------------------   0
JUL04-107       ------------------------------------------------------------   0
JUL03-55        ------------------------------------------------------------   0
JUL02-106       ------------------------------------------------------------   0
JUL01-85        ------------------------------------------------------------   0
DEC03-186       ------------------------------------------------------------   0
DEC07-98        ------------------------------------------------------------   0
APR17-291       ------------------------------------------------------------   0
```

```
DEC02-76        ------------------------------------------------------------   0
CLR03-38395     ------------------------------------------------------------   0
CLR01-43699     ------------------------------------------------------------   0
APR16-68        ------------------------------------------------------------   0
APR18-62        ------------------------------------------------------------   0
APR19-43        ------------------------------------------------------------   0
APR20-70        ------------------------------------------------------------   0
DEC10-249       ------------------------------------------------------------   0
DEC08-241       ------------------------------------------------------------   0
DEC06-81        ------------------------------------------------------------   0
DEC04-13db      ACTACTGCAGAAGCACATTAGTAGCATGAAAGGTTGAAAATGTTTTCTCTAACAAGTCCT   4320
MAY17-11        ------------------------------------------------------------   0
MAY15-78        ------------------------------------------------------------   0
JUL05-102       ------------------------------------------------------------   0
JUL04-107       ------------------------------------------------------------   0
JUL03-55        ------------------------------------------------------------   0
JUL02-106       ------------------------------------------------------------   0
JUL01-85        ------------------------------------------------------------   0
DEC03-186       ------------------------------------------------------------   0
DEC07-98        ------------------------------------------------------------   0
APR17-291       ------------------------------------------------------------   0


DEC02-76        ------------------------------------------------------------   0
CLR03-38395     ------------------------------------------------------------   0
CLR01-43699     ------------------------------------------------------------   0
APR16-68        ------------------------------------------------------------   0
APR18-62        ------------------------------------------------------------   0
APR19-43        ------------------------------------------------------------   0
APR20-70        ------------------------------------------------------------   0
DEC10-249       ------------------------------------------------------------   0
DEC08-241       ------------------------------------------------------------   0
DEC06-81        ------------------------------------------------------------   0
DEC04-13db      CATCAATTATGTTTTCTCCCGATAATTTTATTTGAGAACTAATTTTGAAAATTTCTAAAT   4380
MAY17-11        ------------------------------------------------------------   0
MAY15-78        ------------------------------------------------------------   0
JUL05-102       ------------------------------------------------------------   0
JUL04-107       ------------------------------------------------------------   0
JUL03-55        ------------------------------------------------------------   0
JUL02-106       ------------------------------------------------------------   0
JUL01-85        ------------------------------------------------------------   0
DEC03-186       ------------------------------------------------------------   0
DEC07-98        ------------------------------------------------------------   0
APR17-291       ------------------------------------------------------------   0


DEC02-76        ------------------------------------------------------------   0
CLR03-38395     ------------------------------------------------------------   0
CLR01-43699     ------------------------------------------------------------   0
APR16-68        ------------------------------------------------------------   0
APR18-62        ------------------------------------------------------------   0
APR19-43        ------------------------------------------------------------   0
APR20-70        ------------------------------------------------------------   0
DEC10-249       ------------------------------------------------------------   0
DEC08-241       ------------------------------------------------------------   0
DEC06-81        ------------------------------------------------------------   0
DEC04-13db      TGTATTCACTTACAGTTTTAAAATCTTGCTACTGTAAGTGCATCCAATCATAACGAACTT   4440
MAY17-11        ------------------------------------------------------------   0
MAY15-78        ------------------------------------------------------------   0
JUL05-102       ------------------------------------------------------------   0
JUL04-107       ------------------------------------------------------------   0
JUL03-55        ------------------------------------------------------------   0
JUL02-106       ------------------------------------------------------------   0
```

```
JUL01-85         ------------------------------------------------------------   0
DEC03-186        ------------------------------------------------------------   0
DEC07-98         ------------------------------------------------------------   0
APR17-291        ------------------------------------------------------------   0


DEC02-76         ------------------------------------------------------------   0
CLR03-38395      ------------------------------------------------------------   0
CLR01-43699      ------------------------------------------------------------   0
APR16-68         ------------------------------------------------------------   0
APR18-62         ------------------------------------------------------------   0
APR19-43         ------------------------------------------------------------   0
APR20-70         ------------------------------------------------------------   0
DEC10-249        ------------------------------------------------------------   0
DEC08-241        ------------------------------------------------------------   0
DEC06-81         ------------------------------------------------------------   0
DEC04-13db       TAGGGAGTATCACAGTTTTCTGATGGTCAAATCGTTCTTTCAAATTTTTCCACAACTCAA   4500
MAY17-11         ------------------------------------------------------------   0
MAY15-78         ------------------------------------------------------------   0
JUL05-102        ------------------------------------------------------------   0
JUL04-107        ------------------------------------------------------------   0
JUL03-55         ------------------------------------------------------------   0
JUL02-106        ------------------------------------------------------------   0
JUL01-85         ------------------------------------------------------------   0
DEC03-186        ------------------------------------------------------------   0
DEC07-98         ------------------------------------------------------------   0
APR17-291        ------------------------------------------------------------   0


DEC02-76         ------------------------------------------------------------   0
CLR03-38395      ------------------------------------------------------------   0
CLR01-43699      ------------------------------------------------------------   0
APR16-68         ------------------------------------------------------------   0
APR18-62         ------------------------------------------------------------   0
APR19-43         ------------------------------------------------------------   0
APR20-70         ------------------------------------------------------------   0
DEC10-249        ------------------------------------------------------------   0
DEC08-241        ------------------------------------------------------------   0
DEC06-81         ------------------------------------------------------------   0
DEC04-13db       GAGGGTCTTTCACAGTGAGATATTCCACTTTTAACCCTTCATGTAAATGATGACTAATGA   4560
MAY17-11         ------------------------------------------------------------   0
MAY15-78         ------------------------------------------------------------   0
JUL05-102        ------------------------------------------------------------   0
JUL04-107        ------------------------------------------------------------   0
JUL03-55         ------------------------------------------------------------   0
JUL02-106        ------------------------------------------------------------   0
JUL01-85         ------------------------------------------------------------   0
DEC03-186        ------------------------------------------------------------   0
DEC07-98         ------------------------------------------------------------   0
APR17-291        ------------------------------------------------------------   0


DEC02-76         ------------------------------------------------------------   0
CLR03-38395      ------------------------------------------------------------   0
CLR01-43699      ------------------------------------------------------------   0
APR16-68         ------------------------------------------------------------   0
APR18-62         ------------------------------------------------------------   0
APR19-43         ------------------------------------------------------------   0
APR20-70         ------------------------------------------------------------   0
DEC10-249        ------------------------------------------------------------   0
DEC08-241        ------------------------------------------------------------   0
DEC06-81         ------------------------------------------------------------   0
DEC04-13db       AAATCATTGCTTTTGTCTTGTATTGATTAGATGCTTCTTTATCAACTAATATAGTATTTC   4620
```

```
MAY17-11        ------------------------------------------------------------   0
MAY15-78        ------------------------------------------------------------   0
JUL05-102       ------------------------------------------------------------   0
JUL04-107       ------------------------------------------------------------   0
JUL03-55        ------------------------------------------------------------   0
JUL02-106       ------------------------------------------------------------   0
JUL01-85        ------------------------------------------------------------   0
DEC03-186       ------------------------------------------------------------   0
DEC07-98        ------------------------------------------------------------   0
APR17-291       ------------------------------------------------------------   0


DEC02-76        ------------------------------------------------------------   0
CLR03-38395     ------------------------------------------------------------   0
CLR01-43699     ------------------------------------------------------------   0
APR16-68        ------------------------------------------------------------   0
APR18-62        ------------------------------------------------------------   0
APR19-43        ------------------------------------------------------------   0
APR20-70        ------------------------------------------------------------   0
DEC10-249       ------------------------------------------------------------   0
DEC08-241       ------------------------------------------------------------   0
DEC06-81        ------------------------------------------------------------   0
DEC04-13db      CTAGACCTTTAGTATCTAGGTGAATTTCAGCATCTAGCACCCATGACAAATAATTCTTTC   4680
MAY17-11        ------------------------------------------------------------   0
MAY15-78        ------------------------------------------------------------   0
JUL05-102       ------------------------------------------------------------   0
JUL04-107       ------------------------------------------------------------   0
JUL03-55        ------------------------------------------------------------   0
JUL02-106       ------------------------------------------------------------   0
JUL01-85        ------------------------------------------------------------   0
DEC03-186       ------------------------------------------------------------   0
DEC07-98        ------------------------------------------------------------   0
APR17-291       ------------------------------------------------------------   0


DEC02-76        ------------------------------------------------------------   0
CLR03-38395     ------------------------------------------------------------   0
CLR01-43699     ------------------------------------------------------------   0
APR16-68        ------------------------------------------------------------   0
APR18-62        ------------------------------------------------------------   0
APR19-43        ------------------------------------------------------------   0
APR20-70        ------------------------------------------------------------   0
DEC10-249       ------------------------------------------------------------   0
DEC08-241       ------------------------------------------------------------   0
DEC06-81        ------------------------------------------------------------   0
DEC04-13db      CTGAGATGTCTAAGGCCGTAGATTCAAGTTTAGCAAGATTTGACATTATAATAACTTGAA   4740
MAY17-11        ------------------------------------------------------------   0
MAY15-78        ------------------------------------------------------------   0
JUL05-102       ------------------------------------------------------------   0
JUL04-107       ------------------------------------------------------------   0
JUL03-55        ------------------------------------------------------------   0
JUL02-106       ------------------------------------------------------------   0
JUL01-85        ------------------------------------------------------------   0
DEC03-186       ------------------------------------------------------------   0
DEC07-98        ------------------------------------------------------------   0
APR17-291       ------------------------------------------------------------   0


DEC02-76        ------------------------------------------------------------   0
CLR03-38395     ------------------------------------------------------------   0
CLR01-43699     ------------------------------------------------------------   0
APR16-68        ------------------------------------------------------------   0
APR18-62        ------------------------------------------------------------   0
```

```
APR19-43         ------------------------------------------------------------   0
APR20-70         ------------------------------------------------------------   0
DEC10-249        ------------------------------------------------------------   0
DEC08-241        ------------------------------------------------------------   0
DEC06-81         ------------------------------------------------------------   0
DEC04-13db       TCAAAATAAAAAAAATTAGTAAAATAATAAGCATTCTCAATCATAAAATAATTCAATTAT   4800
MAY17-11         ------------------------------------------------------------   0
MAY15-78         ------------------------------------------------------------   0
JUL05-102        ------------------------------------------------------------   0
JUL04-107        ------------------------------------------------------------   0
JUL03-55         ------------------------------------------------------------   0
JUL02-106        ------------------------------------------------------------   0
JUL01-85         ------------------------------------------------------------   0
DEC03-186        ------------------------------------------------------------   0
DEC07-98         ------------------------------------------------------------   0
APR17-291        ------------------------------------------------------------   0


DEC02-76         ------------------------------------------------------------   0
CLR03-38395      ------------------------------------------------------------   0
CLR01-43699      ------------------------------------------------------------   0
APR16-68         ------------------------------------------------------------   0
APR18-62         ------------------------------------------------------------   0
APR19-43         ------------------------------------------------------------   0
APR20-70         ------------------------------------------------------------   0
DEC10-249        ------------------------------------------------------------   0
DEC08-241        ------------------------------------------------------------   0
DEC06-81         ------------------------------------------------------------   0
DEC04-13db       ATATACCAAATTTGCTAAATAATAATATGTATTTCAAATATTGGCATAGAGAGGAATAGC   4860
MAY17-11         ------------------------------------------------------------   0
MAY15-78         ------------------------------------------------------------   0
JUL05-102        ------------------------------------------------------------   0
JUL04-107        ------------------------------------------------------------   0
JUL03-55         ------------------------------------------------------------   0
JUL02-106        ------------------------------------------------------------   0
JUL01-85         ------------------------------------------------------------   0
DEC03-186        ------------------------------------------------------------   0
DEC07-98         ------------------------------------------------------------   0
APR17-291        ------------------------------------------------------------   0


DEC02-76         ------------------------------------------------------------   0
CLR03-38395      ------------------------------------------------------------   0
CLR01-43699      ------------------------------------------------------------   0
APR16-68         ------------------------------------------------------------   0
APR18-62         ------------------------------------------------------------   0
APR19-43         ------------------------------------------------------------   0
APR20-70         ------------------------------------------------------------   0
DEC10-249        ------------------------------------------------------------   0
DEC08-241        ------------------------------------------------------------   0
DEC06-81         ------------------------------------------------------------   0
DEC04-13db       CATAATAATAACTTAACACAATTACATTAATTGAAATTTCTTTTAACAAAGAAAAACATG   4920
MAY17-11         ------------------------------------------------------------   0
MAY15-78         ------------------------------------------------------------   0
JUL05-102        ------------------------------------------------------------   0
JUL04-107        ------------------------------------------------------------   0
JUL03-55         ------------------------------------------------------------   0
JUL02-106        ------------------------------------------------------------   0
JUL01-85         ------------------------------------------------------------   0
DEC03-186        ------------------------------------------------------------   0
DEC07-98         ------------------------------------------------------------   0
APR17-291        ------------------------------------------------------------   0
```

Figure S4

```
DEC02-76        ------------------------------------------------------------   0
CLR03-38395     ------------------------------------------------------------   0
CLR01-43699     -------------CATTATTAGATTTTAGTTGAAAAAATAAATGTGAAAATATTACCCAA   47
APR16-68        ------------------------------------------------------------   0
APR18-62        ------------------------------------------------------------   0
APR19-43        ------------------------------------------------------------   0
APR20-70        ------------------------------------------------------------   0
DEC10-249       ------------------------------------------------------------   0
DEC08-241       ------------------------------------------------------------   0
DEC06-81        ------------------------------------------------------------   0
DEC04-13db      TATATATAATAATCATTATTAGATTTTAGTTGAAAAAATAAATGTGAAAATATTACCCAA   4980
MAY17-11        ------------------------------------------------------------   0
MAY15-78        ------------------------------------------------------------   0
JUL05-102       ------------------------------------------------------------   0
JUL04-107       ------------------------------------------------------------   0
JUL03-55        ------------------------------------------------------------   0
JUL02-106       ------------------------------------------------------------   0
JUL01-85        ------------------------------------------------------------   0
DEC03-186       ------------------------------------------------------------   0
DEC07-98        ------------------------------------------------------------   0
APR17-291       ------------------------------------------------------------   0


DEC02-76        ------------------------------------------------------------   0
CLR03-38395     ------------------------------------------------------------   0
CLR01-43699     AGCAAATATTTTATATAGAAAATAAAATAAAAGAATTATGAAAATACGTATACCTTATAA   107
APR16-68        ------------------------------------------------------------   0
APR18-62        ------------------------------------------------------------   0
APR19-43        ------------------------------------------------------------   0
APR20-70        ------------------------------------------------------------   0
DEC10-249       ------------------------------------------------------------   0
DEC08-241       ------------------------------------------------------------   0
DEC06-81        ------------------------------------------------------------   0
DEC04-13db      AGCAAATATTTTATATAGAAAATAAAATAAAAGAATTATGAAAATACGTATACCTTATAA   5040
MAY17-11        ------------------------------------------------------------   0
MAY15-78        ------------------------------------------------------------   0
JUL05-102       ------------------------------------------------------------   0
JUL04-107       ------------------------------------------------------------   0
JUL03-55        ------------------------------------------------------------   0
JUL02-106       ------------------------------------------------------------   0
JUL01-85        ------------------------------------------------------------   0
DEC03-186       ------------------------------------------------------------   0
DEC07-98        ------------------------------------------------------------   0
APR17-291       ------------------------------------------------------------   0


DEC02-76        ------------------------------------------------------------   0
CLR03-38395     ------------------------------------------------------------   0
CLR01-43699     AGTTTAACGAGAGTTATACGGACAAATTATATAAAGTCGAAATGATGTAAACTTCACTAT   167
APR16-68        ------------------------------------------------------------   0
APR18-62        ------------------------------------------------------------   0
APR19-43        ------------------------------------------------------------   0
APR20-70        ------------------------------------------------------------   0
DEC10-249       ------------------------------------------------------------   0
DEC08-241       ------------------------------------------------------------   0
DEC06-81        ------------------------------------------------------------   0
DEC04-13db      AGTTTAACGAGAGTTATACGGACAAATTATATAAAGTCGAAATGATGTAAACTTCACTAT   5100
MAY17-11        ------------------------------------------------------------   0
MAY15-78        ------------------------------------------------------------   0
JUL05-102       ------------------------------------------------------------   0
JUL04-107       ------------------------------------------------------------   0
JUL03-55        ------------------------------------------------------------   0
```

Figure S4

```
JUL02-106        ------------------------------------------------------------   0
JUL01-85         ------------------------------------------------------------   0
DEC03-186        ------------------------------------------------------------   0
DEC07-98         ------------------------------------------------------------   0
APR17-291        ------------------------------------------------------------   0


DEC02-76         ------------------------------------------------------------   0
CLR03-38395      ------------------------------------------------------------   0
CLR01-43699      GAACTCGTAGAAGCTCGTGTTGAGAACGTATTATAAAATAATCAAATAAAAGAATACTTA   227
APR16-68         ------------------------------------------------------------   0
APR18-62         ------------------------------------------------------------   0
APR19-43         ------------------------------------------------------------   0
APR20-70         ------------------------------------------------------------   0
DEC10-249        ------------------------------------------------------------   0
DEC08-241        ------------------------------------------------------------   0
DEC06-81         ------------------------------------------------------------   0
DEC04-13db       GAACTCGTAGAAGCTCGTGTTGAGAACGTATTATAAAATAATCAAATAAAAGAATACTTA   5160
MAY17-11         ------------------------------------------------------------   0
MAY15-78         ------------------------------------------------------------   0
JUL05-102        ------------------------------------------------------------   0
JUL04-107        ------------------------------------------------------------   0
JUL03-55         ------------------------------------------------------------   0
JUL02-106        ------------------------------------------------------------   0
JUL01-85         ------------------------------------------------------------   0
DEC03-186        ------------------------------------------------------------   0
DEC07-98         ------------------------------------------------------------   0
APR17-291        ------------------------------------------------------------   0


DEC02-76         ------------------------------------------------------------   0
CLR03-38395      ------------------------------------------------------------   0
CLR01-43699      AAGTAAACAGATAATTTTCTCGTATCTTATTTCACTTAGGTAAGGATTCTATTTAGGGAT   287
APR16-68         ------------------------------------------------------------   0
APR18-62         ------------------------------------------------------------   0
APR19-43         ------------------------------------------------------------   0
APR20-70         ------------------------------------------------------------   0
DEC10-249        ------------------------------------------------------------   0
DEC08-241        ------------------------------------------------------------   0
DEC06-81         ------------------------------------------------------------   0
DEC04-13db       AAGTAAACAGATAATTTTCTCGTATCTTATTTCACTTAGGTAAGGATTCTATTTAGGGAT   5220
MAY17-11         ------------------------------------------------------------   0
MAY15-78         ------------------------------------------------------------   0
JUL05-102        ------------------------------------------------------------   0
JUL04-107        ------------------------------------------------------------   0
JUL03-55         ------------------------------------------------------------   0
JUL02-106        ------------------------------------------------------------   0
JUL01-85         ------------------------------------------------------------   0
DEC03-186        ------------------------------------------------------------   0
DEC07-98         ------------------------------------------------------------   0
APR17-291        ------------------------------------------------------------   0


DEC02-76         ------------------------------------------------------------   0
CLR03-38395      ------------------------------------------------------------   0
CLR01-43699      AATCAAAGAAATAAAATAAAAAATATAGTCTGCTACCCTAGCAAGCTATTGTCTACCGTC   347
APR16-68         ------------------------------------------------------------   0
APR18-62         ------------------------------------------------------------   0
APR19-43         ------------------------------------------------------------   0
APR20-70         ------------------------------------------------------------   0
DEC10-249        ------------------------------------------------------------   0
DEC08-241        ------------------------------------------------------------   0
DEC06-81         ------------------------------------------------------------   0
```

```
DEC04-13db      AATCAAAGAAATAAAATAAAAAATATAGTCTGCTACCCTAGCAAGCTATTGTCTACCGTC   5280
MAY17-11        ------------------------------------------------------------   0
MAY15-78        ------------------------------------------------------------   0
JUL05-102       ------------------------------------------------------------   0
JUL04-107       ------------------------------------------------------------   0
JUL03-55        ------------------------------------------------------------   0
JUL02-106       ------------------------------------------------------------   0
JUL01-85        ------------------------------------------------------------   0
DEC03-186       ------------------------------------------------------------   0
DEC07-98        ------------------------------------------------------------   0
APR17-291       ------------------------------------------------------------   0


DEC02-76        ------------------------------------------------------------   0
CLR03-38395     ------------------------------------------------------------   0
CLR01-43699     TCTGGCAATAAGTTTTAGGTTTTGCTATATTTTTTTATGTTAGTTTGTCGTGCTTGTTTG   407
APR16-68        ------------------------------------------------------------   0
APR18-62        ------------------------------------------------------------   0
APR19-43        ------------------------------------------------------------   0
APR20-70        ------------------------------------------------------------   0
DEC10-249       ------------------------------------------------------------   0
DEC08-241       ------------------------------------------------------------   0
DEC06-81        ------------------------------------------------------------   0
DEC04-13db      TCTGGCAATAAGTTTTAGGTTTTGCTATATTTTTTTATGTTAGTTTGTCGTGCTTGTTTG   5340
MAY17-11        ------------------------------------------------------------   0
MAY15-78        ------------------------------------------------------------   0
JUL05-102       ------------------------------------------------------------   0
JUL04-107       ------------------------------------------------------------   0
JUL03-55        ------------------------------------------------------------   0
JUL02-106       ------------------------------------------------------------   0
JUL01-85        ------------------------------------------------------------   0
DEC03-186       ------------------------------------------------------------   0
DEC07-98        ------------------------------------------------------------   0
APR17-291       ------------------------------------------------------------   0


DEC02-76        ------------------------------------------------------------   0
CLR03-38395     ------------------------------------------------------------   0
CLR01-43699     ATTTTGGTCTCTGTGAGGCGATGCCCTTGAGTGCTTAGCGACTATTATCCGTAGGAAGAT   467
APR16-68        ------------------------------------------------------------   0
APR18-62        ------------------------------------------------------------   0
APR19-43        ------------------------------------------------------------   0
APR20-70        ------------------------------------------------------------   0
DEC10-249       ------------------------------------------------------------   0
DEC08-241       ------------------------------------------------------------   0
DEC06-81        ------------------------------------------------------------   0
DEC04-13db      ATTTTGGTCTCTGTGAGGCGATGCCCTTGAGTGCTTAGCGACTATTATCCGTAGGAAGAT   5400
MAY17-11        ------GTCTCTGTGAGGCGATGCCCTTGAGTGCTTAGCGACTATTATCCGTAGGAAGAT   54
MAY15-78        ------------------------------------------------------------   0
JUL05-102       ------------------------------------------------------------   0
JUL04-107       ------------------------------------------------------------   0
JUL03-55        ------------------------------------------------------------   0
JUL02-106       ------------------------------------------------------------   0
JUL01-85        ------------------------------------------------------------   0
DEC03-186       ------------------------------------------------------------   0
DEC07-98        ------------------------------------------------------------   0
APR17-291       ------------------------------------------------------------   0


DEC02-76        ------------------------------------------------------------   0
CLR03-38395     ------------------------------------------------------------   0
CLR01-43699     AGAAAAATTTGCAATCTACACCTGCAAGCATGGAGAATGAATTGGCTCAACTTTCAATTA   527
APR16-68        ------------------------------------------------------------   0
```

Figure S4

```
APR18-62        ------------------------------------------------------------   0
APR19-43        ------------------------------------------------------------   0
APR20-70        ------------------------------------------------------------   0
DEC10-249       ------------------------------------------------------------   0
DEC08-241       ------------------------------------------------------------   0
DEC06-81        ------------------------------------------------------------   0
DEC04-13db      AGAAAAATTTGCAATCTACACCTGCAAGCATGGAGAATGAATTGGCTCAACTTTCAATTA   5460
MAY17-11        AGAAAAATTTGCAATCTACACCTGCAAGCATGGAGAATGAATTGGCTCAACTTTCAATTA   114
MAY15-78        ------------------------------------------------------------   0
JUL05-102       ------------------------------------------------------------   0
JUL04-107       ------------------------------------------------------------   0
JUL03-55        ------------------------------------------------------------   0
JUL02-106       ------------------------------------------------------------   0
JUL01-85        ------------------------------------------------------------   0
DEC03-186       ------------------------------------------------------------   0
DEC07-98        ------------------------------------------------------------   0
APR17-291       ------------------------------------------------------------   0


DEC02-76        ------------------------------------------------------------   0
CLR03-38395     ------------------------------------------------------------   0
CLR01-43699     ATGAAGAAAAATACGATATTATTCAAATTCAACCAGATCTGAACAAGGAAGAAGAGGGGG   587
APR16-68        ------------------------------------------------------------   0
APR18-62        ------------------------------------------------------------   0
APR19-43        ------------------------------------------------------------   0
APR20-70        ------------------------------------------------------------   0
DEC10-249       ------------------------------------------------------------   0
DEC08-241       ------------------------------------------------------------   0
DEC06-81        ------------------------------------------------------------   0
DEC04-13db      ATGAAGAAAAATACGATATTATTCAAATTCAACCAGATCTGAACAAGGAAGAAGAGGGGG   5520
MAY17-11        ATGAAGAAAAATACGATATTATTCAAATTCAACCAGATCTGAACAAGGAAGAAGAGGGGG   174
MAY15-78        ------------------------------------------------------------   0
JUL05-102       ------------------------------------------------------------   0
JUL04-107       ------------------------------------------------------------   0
JUL03-55        ------------------------------------------------------------   0
JUL02-106       ------------------------------------------------------------   0
JUL01-85        ------------------------------------------------------------   0
DEC03-186       ------------------------------------------------------------   0
DEC07-98        ------------------------------------------------------------   0
APR17-291       ------------------------------------------------------------   0


DEC02-76        ------------------------------------------------------------   0
CLR03-38395     ------------------------------------------------------------   0
CLR01-43699     AGAGTTTTCTATTAGCGGGTTATTTCTTGACAACTAGTATCATTTACTTTCCTGCAATGA   647
APR16-68        ------------------------------------------------------------   0
APR18-62        ------------------------------------------------------------   0
APR19-43        ------------------------------------------------------------   0
APR20-70        ------------------------------------------------------------   0
DEC10-249       ------------------------------------------------------------   0
DEC08-241       ------------------------------------------------------------   0
DEC06-81        ------------------------------------------------------------   0
DEC04-13db      AGAGTTTTCTATTAGCGGGTTATTTCTTGACAACTAGTATCATTTACTTTCCTGCAATGA   5580
MAY17-11        AGAGTTTTCTATTAGCGGGTTATTTCTTGACAACTAGTATCATTTACTTTCCTGCAATGA   234
MAY15-78        ------------------------------------------------------------   0
JUL05-102       ------------------------------------------------------------   0
JUL04-107       ------------------------------------------------------------   0
JUL03-55        ------------------------------------------------------------   0
JUL02-106       ------------------------------------------------------------   0
JUL01-85        ------------------------------------------------------------   0
DEC03-186       ------------------------------------------------------------   0
DEC07-98        ------------------------------------------------------------   0
APR17-291       ------------------------------------------------------------   0
```

Figure S4

```
DEC02-76        ------------------------------------------------------------   0
CLR03-38395     ------------------------------------------------------------   0
CLR01-43699     AGAGTACTTCGGCAAAATTATGGCATCCGGTGAGGGGAGTTTAGATTCGAGATCTGGGGG   707
APR16-68        ------------------------------------------------------------   0
APR18-62        ------------------------------------------------------------   0
APR19-43        ------------------------------------------------------------   0
APR20-70        ------------------------------------------------------------   0
DEC10-249       ------------------------------------------------------------   0
DEC08-241       ------------------------------------------------------------   0
DEC06-81        ------------------------------------------------------------   0
DEC04-13db      AGAGTACTTCGGCAAAATTATGGCATCCGGTGAGGGGAGTTTAGATTCGAGATCTGGGGG   5640
MAY17-11        AGAGTACTTCGGCAAAATTATGGCATCCGGTGAGGGGAGTTTAGATTCGAGATCTGGGGG   294
MAY15-78        ------------------------------------------------------------   0
JUL05-102       ------------------------------------------------------------   0
JUL04-107       ------------------------------------------------------------   0
JUL03-55        ------------------------------------------------------------   0
JUL02-106       ------------------------------------------------------------   0
JUL01-85        ------------------------------------------------------------   0
DEC03-186       ------------------------------------------------------------   0
DEC07-98        ------------------------------------------------------------   0
APR17-291       ------------------------------------------------------------   0


DEC02-76        ------------------------------------------------------------   0
CLR03-38395     ------------------------------------------------------------   0
CLR01-43699     AGAAAAGGTATTTGTTCCAATTTTTTCATATGATGGACTTGGAAAGGGTTCTTAAAGGCT   767
APR16-68        ------------------------------------------------------------   0
APR18-62        ------------------------------------------------------------   0
APR19-43        ------------------------------------------------------------   0
APR20-70        ------------------------------------------------------------   0
DEC10-249       ------------------------------------------------------------   0
DEC08-241       ------------------------------------------------------------   0
DEC06-81        ------------------------------------------------------------   0
DEC04-13db      AGAAAAGGTATTTGTTCCAATTTTTTCATATGATGGACTTGGAAAGGGTTCTTAAAGGCT   5700
MAY17-11        AGAAAAGGTATTTGTTCCAATTTTTTCATATGATGGACTTGGAAAGGGTTCTTAAAGGCT   354
MAY15-78        ------------------------------------------------------------   0
JUL05-102       ------------------------------------------------------------   0
JUL04-107       ------------------------------------------------------------   0
JUL03-55        ------------------------------------------------------------   0
JUL02-106       ------------------------------------------------------------   0
JUL01-85        ------------------------------------------------------------   0
DEC03-186       ------------------------------------------------------------   0
DEC07-98        ------------------------------------------------------------   0
APR17-291       ------------------------------------------------------------   0


DEC02-76        ------------------------------------------------------------   0
CLR03-38395     ------------------------------------------------------------   0
CLR01-43699     CCCCTTAGACTTTTAATAATCATTTGCTGCTTCTTTATAAACAGCAATAGGGGGAGGATC   827
APR16-68        ------------------------------------------------------------   0
APR18-62        ------------------------------------------------------------   0
APR19-43        ------------------------------------------------------------   0
APR20-70        ------------------------------------------------------------   0
DEC10-249       ------------------------------------------------------------   0
DEC08-241       ------------------------------------------------------------   0
DEC06-81        ------------------------------------------------------------   0
DEC04-13db      CCCCTTAGACTTTTAATAATCATTTGCTGCTTCTTTATAAACAGCAATAGGGGGAGGATC   5760
MAY17-11        CCCCTTAGACTTTTAATAATCATTTGCTGCTTCTTTATAAACAGCAATAGGGGGAGGATC   414
MAY15-78        ------------------------------------------------------------   0
JUL05-102       ------------------------------------------------------------   0
JUL04-107       ------------------------------------------------------------   0
```

Figure S4

```
JUL03-55       ------------------------------------------------------------   0
JUL02-106      ------------------------------------------------------------   0
JUL01-85       ------------------------------------------------------------   0
DEC03-186      ------------------------------------------------------------   0
DEC07-98       ------------------------------------------------------------   0
APR17-291      ------------------------------------------------------------   0


DEC02-76       ------------------------------------------------------------   0
CLR03-38395    ------------------------------------------------------------   0
CLR01-43699    CATTACAAGTACCTTTAGTTATGGCACCTTTTTGGGTGCAGATTCATAATGTACCTATTG   887
APR16-68       ------------------------------------------------------------   0
APR18-62       ------------------------------------------------------------   0
APR19-43       ------------------------------------------------------------   0
APR20-70       ------------------------------------------------------------   0
DEC10-249      ------------------------------------------------------------   0
DEC08-241      ------------------------------------------------------------   0
DEC06-81       ------------------------------------------------------------   0
DEC04-13db     CATTACAAGTACCTTTAGTTATGGCACCTTTTTGGGTGCAGATTCATAATGTACCTATTG   5820
MAY17-11       CATTACAAGTACCTTTAGTTATGGCACCTTTTTGGGTGCAGATTCATAATGTACCTATTG   474
MAY15-78       ------------------------------------------------------------   0
JUL05-102      ------------------------------------------------------------   0
JUL04-107      ------------------------------------------------------------   0
JUL03-55       ------------------------------------------------------------   0
JUL02-106      ------------------------------------------------------------   0
JUL01-85       ------------------------------------------------------------   0
DEC03-186      ------------------------------------------------------------   0
DEC07-98       ------------------------------------------------------------   0
APR17-291      ------------------------------------------------------------   0


DEC02-76       ------------------------------------------------------------   0
CLR03-38395    ------------------------------------------------------------   0
CLR01-43699    GTTTATTCTCTGAAAATTTGGCGATGTAAATAAGAAATTTTATAGGTGTTTTTCTGGAAT   947
APR16-68       ------------------------------------------------------------   0
APR18-62       ------------------------------------------------------------   0
APR19-43       ------------------------------------------------------------   0
APR20-70       ------------------------------------------------------------   0
DEC10-249      ------------------------------------------------------------   0
DEC08-241      ------------------------------------------------------------   0
DEC06-81       ------------------------------------------------------------   0
DEC04-13db     GTTTATTCTCTGAAAATTTGGCGATGTAAATAAGAAATTTTATAGGTGTTTTTCTGGAAT   5880
MAY17-11       GTTTATTCTCTGAAAATTTGGCGATGTAAATAAGAAATTTTATAGGTGTTTTTCTGGAAT   534
MAY15-78       ------------------------------------------------------------   0
JUL05-102      ------------------------------------------------------------   0
JUL04-107      ------------------------------------------------------------   0
JUL03-55       ------------------------------------------------------------   0
JUL02-106      ------------------------------------------------------------   0
JUL01-85       ------------------------------------------------------------   0
DEC03-186      ------------------------------------------------------------   0
DEC07-98       ------------------------------------------------------------   0
APR17-291      ------------------------------------------------------------   0


DEC02-76       ------------------------------------------------------------   0
CLR03-38395    ------------------------------------------------------------   0
CLR01-43699    ATGATGCATCAAATCTGGAGAAGGAGAATAGGACTTTCATGAGAATCAGAGTTTAAATTG   1007
APR16-68       ------------------------------------------------------------   0
APR18-62       ------------------------------------------------------------   0
APR19-43       ------------------------------------------------------------   0
APR20-70       ------------------------------------------------------------   0
DEC10-249      ------------------------------------------------------------   0
DEC08-241      ------------------------------------------------------------   0
```

Figure S4

```
DEC06-81         ------------------------------------------------------------   0
DEC04-13db       ATGATGCATCAAATCTGGAGAAGGAGAATAGGACTTTCATGAGAATCAGAGTTTAAATTG   5940
MAY17-11         ATGATGCATCAAATCTGGAGAAGGAGAATAGGACTTTCATGAGAATCAGAGTTTAAATTG   594
MAY15-78         ------------------------------------------------------------   0
JUL05-102        ------------------------------------------------------------   0
JUL04-107        ------------------------------------------------------------   0
JUL03-55         ------------------------------------------------------------   0
JUL02-106        ------------------------------------------------------------   0
JUL01-85         ------------------------------------------------------------   0
DEC03-186        ------------------------------------------------------------   0
DEC07-98         ------------------------------------------------------------   0
APR17-291        ------------------------------------------------------------   0


DEC02-76         ------------------------------------------------------------   0
CLR03-38395      ------------------------------------------------------------   0
CLR01-43699      ATGTTAGGCTCCCTTTGAAAAGGAAAAAAATAGATTCTATTTTGTGGGAGATGTTCATAT   1067
APR16-68         ------------------------------------------------------------   0
APR18-62         ------------------------------------------------------------   0
APR19-43         ------------------------------------------------------------   0
APR20-70         ------------------------------------------------------------   0
DEC10-249        ------------------------------------------------------------   0
DEC08-241        ------------------------------------------------------------   0
DEC06-81         ------------------------------------------------------------   0
DEC04-13db       ATGTTAGGCTCCCTTTGAAAAGGAAAAAAATAGATTCTATTTTGTGGGAGATGTTCATAT   6000
MAY17-11         ATGTTAGGCTCCCTTTGAAAAGGAAAAAAATAGATTCTATTTTGTGGGAGATGTTCATAT   654
MAY15-78         ------------------------------------------------------------   0
JUL05-102        ------------------------------------------------------------   0
JUL04-107        ------------------------------------------------------------   0
JUL03-55         ------------------------------------------------------------   0
JUL02-106        ------------------------------------------------------------   0
JUL01-85         ------------------------------------------------------------   0
DEC03-186        ------------------------------------------------------------   0
DEC07-98         ------------------------------------------------------------   0
APR17-291        ------------------------------------------------------------   0


DEC02-76         ------------------------------------------------------------   0
CLR03-38395      ------------------------------------------------------------   0
CLR01-43699      GTCAATTTTAAATATGAGCGATTACCATTATTTTGCTTTTATTGTGGTCGACTAGGTCAT   1127
APR16-68         ------------------------------------------------------------   0
APR18-62         ------------------------------------------------------------   0
APR19-43         ------------------------------------------------------------   0
APR20-70         ------------------------------------------------------------   0
DEC10-249        ------------------------------------------------------------   0
DEC08-241        ------------------------------------------------------------   0
DEC06-81         ------------------------------------------------------------   0
DEC04-13db       GTCAATTTTAAATATGAGCGATTACCATTATTTTGCTTTTATTGTGGTCGACTAGGTCAT   6060
MAY17-11         GTCAATTTTAAATATGAGCGATTACCATTATTTTGCTTTTATTGTGGTCGACTAGGTCAT   714
MAY15-78         ------------------------------------------------------------   0
JUL05-102        ------------------------------------------------------------   0
JUL04-107        ------------------------------------------------------------   0
JUL03-55         ------------------------------------------------------------   0
JUL02-106        ------------------------------------------------------------   0
JUL01-85         ------------------------------------------------------------   0
DEC03-186        ------------------------------------------------------------   0
DEC07-98         ------------------------------------------------------------   0
APR17-291        ------------------------------------------------------------   0


DEC02-76         ------------------------------------------------------------   0
CLR03-38395      ------------------------------------------------------------   0
CLR01-43699      AATGACTAATTTTGTGAAGCTAAGATGATACTTGGTGTGGAAGCCGCTGATATGGGTTGG   1187
```

Figure S4

```
APR16-68        ------------------------------------------------------------   0
APR18-62        ------------------------------------------------------------   0
APR19-43        ------------------------------------------------------------   0
APR20-70        ------------------------------------------------------------   0
DEC10-249       ------------------------------------------------------------   0
DEC08-241       ------------------------------------------------------------   0
DEC06-81        ------------------------------------------------------------   0
DEC04-13db      AATGACTAATTTTGTGAAGCTAAGATGATACTTGGTGTGGAAGCCGCTGATATGGGTTGG   6120
MAY17-11        AATGACTAATTTTGTGAAGCTAAGATGATACTTGGTGTGGAAGCCGCTGATATGGGTTGG   774
MAY15-78        ------------------------------------------------------------   0
JUL05-102       ------------------------------------------------------------   0
JUL04-107       ------------------------------------------------------------   0
JUL03-55        ------------------------------------------------------------   0
JUL02-106       ------------------------------------------------------------   0
JUL01-85        ------------------------------------------------------------   0
DEC03-186       ------------------------------------------------------------   0
DEC07-98        ------------------------------------------------------------   0
APR17-291       ------------------------------------------------------------   0


DEC02-76        ------------------------------------------------------------   0
CLR03-38395     ------------------------------------------------------------   0
CLR01-43699     GATCTATCTTTATGAACTCAATCCCGAAGAGCCTTAACTATGAATAGTATCTGGCTACGT   1247
APR16-68        ------------------------------------------------------------   0
APR18-62        ------------------------------------------------------------   0
APR19-43        ------------------------------------------------------------   0
APR20-70        ------------------------------------------------------------   0
DEC10-249       ------------------------------------------------------------   0
DEC08-241       ------------------------------------------------------------   0
DEC06-81        ------------------------------------------------------------   0
DEC04-13db      GATCTATCTTTATGAACTCAATCCCGAAGAGCCTTAACTATGAATAGTATCTGGCTACGT   6180
MAY17-11        GATCTATCTTTATGAACTCAATCCCGAAGAGCCTTAACTATGAATAGTATCTGGCTACGT   834
MAY15-78        ------------------------------------------------------------   0
JUL05-102       ------------------------------------------------------------   0
JUL04-107       ------------------------------------------------------------   0
JUL03-55        ------------------------------------------------------------   0
JUL02-106       ------------------------------------------------------------   0
JUL01-85        ------------------------------------------------------------   0
DEC03-186       ------------------------------------------------------------   0
DEC07-98        ------------------------------------------------------------   0
APR17-291       ------------------------------------------------------------   0


DEC02-76        ------------------------------------------------------------   0
CLR03-38395     ------------------------------------------------------------   0
CLR01-43699     GAGGTTGGAGATGGTAAAGTGGATGGTAGTAACTAAGGTAATGGACTCATAAAGAATGGG   1307
APR16-68        ------------------------------------------------------------   0
APR18-62        ------------------------------------------------------------   0
APR19-43        ------------------------------------------------------------   0
APR20-70        ------------------------------------------------------------   0
DEC10-249       ------------------------------------------------------------   0
DEC08-241       ------------------------------------------------------------   0
DEC06-81        ------------------------------------------------------------   0
DEC04-13db      GAGGTTGGAGATGGTAAAGTGGATGGTAGTAACTAAGGTAATGGACTCATAAAGAATGGG   6240
MAY17-11        GAGGTTGGAGATGGTAAAGTGGATGGTAGTAACTAAGGTAATGGACTCATAAAGAATGGG   894
MAY15-78        ------------------------------------------------------------   0
JUL05-102       ------------------------------------------------------------   0
JUL04-107       ------------------------------------------------------------   0
JUL03-55        ------------------------------------------------------------   0
JUL02-106       ------------------------------------------------------------   0
JUL01-85        ------------------------------------------------------------   0
DEC03-186       ------------------------------------------------------------   0
DEC07-98        ------------------------------------------------------------   0
```

Figure S4

```
APR17-291       ------------------------------------------------------------   0

DEC02-76        ------------------------------------------------------------   0
CLR03-38395     ------------------------------------------------------------   0
CLR01-43699     CAATGGCAAGCGGAAGATAAAAAGAGTTGCAGAAAGAGGGTAGACCCAATTCTTGAATTC   1367
APR16-68        ------------------------------------------------------------   0
APR18-62        ------------------------------------------------------------   0
APR19-43        ------------------------------------------------------------   0
APR20-70        ------------------------------------------------------------   0
DEC10-249       ------------------------------------------------------------   0
DEC08-241       ------------------------------------------------------------   0
DEC06-81        ------------------------------------------------------------   0
DEC04-13db      CAATGGCAAGCGGAAGATAAAAAGAGTTGCAGAAAGAGGGTAGACCCAATTCTTGAATTC   6300
MAY17-11        CAATGGCAAGCGGAAGATAAAAAGAGTTGCAGAAAGAGGGTAGACCCAATTCTTGAATTC   954
MAY15-78        ------------------------------------------------------------   0
JUL05-102       ------------------------------------------------------------   0
JUL04-107       ------------------------------------------------------------   0
JUL03-55        ------------------------------------------------------------   0
JUL02-106       ------------------------------------------------------------   0
JUL01-85        ------------------------------------------------------------   0
DEC03-186       ------------------------------------------------------------   0
DEC07-98        ------------------------------------------------------------   0
APR17-291       ------------------------------------------------------------   0

DEC02-76        ------------------------------------------------------------   0
CLR03-38395     ------------------------------------------------------------   0
CLR01-43699     AACATAGAAGGGGTATCATAAGGTTTTGGCGAATGGATGAAAGACGTTTTGTTAGGTCAA   1427
APR16-68        ------------------------------------------------------------   0
APR18-62        ------------------------------------------------------------   0
APR19-43        ------------------------------------------------------------   0
APR20-70        ------------------------------------------------------------   0
DEC10-249       ------------------------------------------------------------   0
DEC08-241       ------------------------------------------------------------   0
DEC06-81        ------------------------------------------------------------   0
DEC04-13db      AACATAGAAGGGGTATCATAAGGTTTTGGCGAATGGATGAAAGACGTTTTGTTAGGTCAA   6360
MAY17-11        AACATAGAAGGGGTATCATAAGGTTTTGGCGAATGGATGAAAGACGTTTTGTTAGGTCAA   1014
MAY15-78        ------------------------------------------------------------   0
JUL05-102       ------------------------------------------------------------   0
JUL04-107       ------------------------------------------------------------   0
JUL03-55        ------------------------------------------------------------   0
JUL02-106       ------------------------------------------------------------   0
JUL01-85        ------------------------------------------------------------   0
DEC03-186       ------------------------------------------------------------   0
DEC07-98        ------------------------------------------------------------   0
APR17-291       ------------------------------------------------------------   0

DEC02-76        ------------------------------------------------------------   0
CLR03-38395     ------------------------------------------------------------   0
CLR01-43699     ATCTATAGTACGATGGAACATGACATGGATGATACAACATTAATAGGAGAGGAGGGCAAG   1487
APR16-68        ------------------------------------------------------------   0
APR18-62        ------------------------------------------------------------   0
APR19-43        ------------------------------------------------------------   0
APR20-70        ------------------------------------------------------------   0
DEC10-249       ------------------------------------------------------------   0
DEC08-241       ------------------------------------------------------------   0
DEC06-81        ------------------------------------------------------------   0
DEC04-13db      ATCTATAGTACGATGGAACATGACATGGATGATACAACATTAATAGGAGAGGAGGGCAAG   6420
MAY17-11        ATCTATAGTACGATGGAACATGACATGGATGATACAACATTAATAGGAGAGGAGGGCAAG   1074
MAY15-78        ------------------------------------------------------------   0
JUL05-102       ------------------------------------------------------------   0
```

Figure S4

```
JUL04-107       ------------------------------------------------------------   0
JUL03-55        ------------------------------------------------------------   0
JUL02-106       ------------------------------------------------------------   0
JUL01-85        ------------------------------------------------------------   0
DEC03-186       ------------------------------------------------------------   0
DEC07-98        ------------------------------------------------------------   0
APR17-291       ------------------------------------------------------------   0


DEC02-76        ------------------------------------------------------------   0
CLR03-38395     ------------------------------------------------------------   0
CLR01-43699     AAAAGGTCTAGAAGGGAAGTTGATGAGTCATCAGGAAAAAATGAGGTGAACAAATTAATG   1547
APR16-68        ------------------------------------------------------------   0
APR18-62        ------------------------------------------------------------   0
APR19-43        ------------------------------------------------------------   0
APR20-70        ------------------------------------------------------------   0
DEC10-249       ------------------------------------------------------------   0
DEC08-241       ------------------------------------------------------------   0
DEC06-81        ------------------------------------------------------------   0
DEC04-13db      AAAAGGTCTAGAAGGGAAGTTGATGAGTCATCAGGAAAAAATGAGGTGAACAAATTAATG   6480
MAY17-11        AAAAGGTCTAGAAGGGAAGTTGATGAGTCATCAGGAAAAAATGAGGTGAACAAATTAATG   1134
MAY15-78        ------------------------------------------------------------   0
JUL05-102       ------------------------------------------------------------   0
JUL04-107       ------------------------------------------------------------   0
JUL03-55        ------------------------------------------------------------   0
JUL02-106       ------------------------------------------------------------   0
JUL01-85        ------------------------------------------------------------   0
DEC03-186       ------------------------------------------------------------   0
DEC07-98        ------------------------------------------------------------   0
APR17-291       ------------------------------------------------------------   0


DEC02-76        ------------------------------------------------------------   0
CLR03-38395     ------------------------------------------------------------   0
CLR01-43699     TCAAGAAGCGGGAGAATGGGGGATCAAAATCATTTATTATGACTGTATACACAAGAAAAT   1607
APR16-68        ------------------------------------------------------------   0
APR18-62        ------------------------------------------------------------   0
APR19-43        ------------------------------------------------------------   0
APR20-70        ------------------------------------------------------------   0
DEC10-249       ------------------------------------------------------------   0
DEC08-241       ------------------------------------------------------------   0
DEC06-81        ------------------------------------------------------------   0
DEC04-13db      TCAAGAAGCGGGAGAATGGGGGATCAAAATCATTTATTATGACTGTATACACAAGAAAAT   6540
MAY17-11        TCAAGAAGCGGGAGAATGGGGGATCAAAATCATTTATTATGACTGTATACACAAGAAAAT   1194
MAY15-78        ------------------------------------------------------------   0
JUL05-102       ------------------------------------------------------------   0
JUL04-107       ------------------------------------------------------------   0
JUL03-55        ------------------------------------------------------------   0
JUL02-106       ------------------------------------------------------------   0
JUL01-85        ------------------------------------------------------------   0
DEC03-186       ------------------------------------------------------------   0
DEC07-98        ------------------------------------------------------------   0
APR17-291       ------------------------------------------------------------   0


DEC02-76        ------------------------------------------------------------   0
CLR03-38395     ------------------------------------------------------------   0
CLR01-43699     TAAGAGGATCAAAGATAAACAGGAGAAAATGATAAATGAAATTATAAATTATGACTTTAA   1667
APR16-68        ------------------------------------------------------------   0
APR18-62        ------------------------------------------------------------   0
APR19-43        ------------------------------------------------------------   0
APR20-70        ------------------------------------------------------------   0
DEC10-249       ------------------------------------------------------------   0
```

Figure S4

```
DEC08-241       ------------------------------------------------------------   0
DEC06-81        ------------------------------------------------------------   0
DEC04-13db      TAAGAGGATCAAAGATAAACAGGAGAAAATGATAAATGAAATTATAAATTATGACTTTAA   6600
MAY17-11        TAAGAGGATCAAAGATAAACAGGAGAAAATGATAAATGAAATTATAAATTATGACTTTAA   1254
MAY15-78        ------------------------------------------------------------   0
JUL05-102       ------------------------------------------------------------   0
JUL04-107       ------------------------------------------------------------   0
JUL03-55        ------------------------------------------------------------   0
JUL02-106       ------------------------------------------------------------   0
JUL01-85        ------------------------------------------------------------   0
DEC03-186       ------------------------------------------------------------   0
DEC07-98        ------------------------------------------------------------   0
APR17-291       ------------------------------------------------------------   0


DEC02-76        ------------------------------------------------------------   0
CLR03-38395     ------------------------------------------------------------   0
CLR01-43699     TCCGCAGGAAATTTATCAAATTCTCGAGGAAGGTGACTCCCTAACCAAGGGTGCATCGCA   1727
APR16-68        ------------------------------------------------------------   0
APR18-62        ------------------------------------------------------------   0
APR19-43        ------------------------------------------------------------   0
APR20-70        ------------------------------------------------------------   0
DEC10-249       ------------------------------------------------------------   0
DEC08-241       ------------------------------------------------------------   0
DEC06-81        ------------------------------------------------------------   0
DEC04-13db      TCCGCAGGAAATTTATCAAATTCTCGAGGAAGGTGACTCCCTAACCAAGGGTGCATCGCA   6660
MAY17-11        TCCGCAGGAAATTTATCAAATTCTCGAGGAAGGTGACTCCCTAACCAAGGGTGCATCGCA   1314
MAY15-78        ------------------------------------------------------------   0
JUL05-102       ------------------------------------------------------------   0
JUL04-107       ------------------------------------------------------------   0
JUL03-55        ------------------------------------------------------------   0
JUL02-106       ------------------------------------------------------------   0
JUL01-85        ------------------------------------------------------------   0
DEC03-186       ------------------------------------------------------------   0
DEC07-98        ------------------------------------------------------------   0
APR17-291       ------------------------------------------------------------   0


DEC02-76        ----------TGGTATCAGAGTAAATCACGATCTCAGGACAAATAAAATGGATGAAAATC   50
CLR03-38395     ------------------------------------------------------------   0
CLR01-43699     ATAAAAAATCTGGTATCAGAGTAAATCACGATCTCAGGACAAATAAAATGGATGAAAATC   1787
APR16-68        ------------------------------------------------------------   0
APR18-62        ------------------------------------------------------------   0
APR19-43        ------------------------------------------------------------   0
APR20-70        ------------------------------------------------------------   0
DEC10-249       ------------------------------------------------------------   0
DEC08-241       ------------------------------------------------------------   0
DEC06-81        ------------------------------------------------------------   0
DEC04-13db      ATAAAAAATCTGGTATCAGAGTAAATCACGATCTCAGGACAAATAAAATGGATGAAAATC   6720
MAY17-11        ATAAAAAATCTGGTATCAGAGTAAATCACGATCTCAGGACAAATAAAATGGATGAAAATC   1374
MAY15-78        ------------------------------------------------------------   0
JUL05-102       ------------------------------------------------------------   0
JUL04-107       ------------------------------------------------------------   0
JUL03-55        ------------------------------------------------------------   0
JUL02-106       ------------------------------------------------------------   0
JUL01-85        ------------------------------------------------------------   0
DEC03-186       ------------------------------------------------------------   0
DEC07-98        ------------------------------------------------------------   0
APR17-291       ------------------------------------------------------------   0


DEC02-76        CTGAATATAAAGGATATAAGGATGTAAAAGAAATCAAAGAACAATTAAGCCTAATAATGA   110
CLR03-38395     ------------------------------------------------------------   0
```

Figure S4

```
CLR01-43699     CTGAATATAAAGGATATAAGGATGTAAAAGAAATCAAAGAACAATTAAGCCTAATAATGA   1847
APR16-68        ------------------------------------------------------------   0
APR18-62        ------------------------------------------------------------   0
APR19-43        ------------------------------------------------------------   0
APR20-70        ------------------------------------------------------------   0
DEC10-249       ------------------------------------------------------------   0
DEC08-241       ------------------------------------------------------------   0
DEC06-81        ------------------------------------------------------------   0
DEC04-13db      CTGAATATAAAGGATATAAGGATGTAAAAGAAATCAAAGAACAATTAAGCCTAATAATGA   6780
MAY17-11        CTGAATATAAAGGATATAAGGATGTAAAAGAAATCAAAGAACAATTAAGCCTAATAATGA   1434
MAY15-78        ------------------------------------------------------------   0
JUL05-102       ------------------------------------------------------------   0
JUL04-107       ------------------------------------------------------------   0
JUL03-55        ------------------------------------------------------------   0
JUL02-106       ------------------------------------------------------------   0
JUL01-85        ------------------------------------------------------------   0
DEC03-186       ------------------------------------------------------------   0
DEC07-98        ------------------------------------------------------------   0
APR17-291       ------------------------------------------------------------   0


DEC02-76        TTCAAATAAACAGGACTCAAGAGCAAATAATAAAGCTCCAAGAGACTGTTAATCTGATAG   170
CLR03-38395     ------------------------------------------------------------   0
CLR01-43699     TTCAAATAAACAGGACTCAAGAGCAAATAATAAAGCTCCAAGAGACTGTTAATCTGATAG   1907
APR16-68        ------------------------------------------------------------   0
APR18-62        ------------------------------------------------------------   0
APR19-43        ------------------------------------------------------------   0
APR20-70        ------------------------------------------------------------   0
DEC10-249       ------------------------------------------------------------   0
DEC08-241       ------------------------------------------------------------   0
DEC06-81        ------------------------------------------------------------   0
DEC04-13db      TTCAAATAAACAGGACTCAAGAGCAAATAATAAAGCTCCAAGAGACTGTTAATCTGATAG   6840
MAY17-11        TTCAAATAAACAGGACTCAAGAGCAAATAATAAAGCTCCAAGAGACTGTTAATCTGATAG   1494
MAY15-78        ------------------------------------------------------------   0
JUL05-102       ------------------------------------------------------------   0
JUL04-107       ------------------------------------------------------------   0
JUL03-55        ------------------------------------------------------------   0
JUL02-106       ------------------------------------------------------------   0
JUL01-85        ------------------------------------------------------------   0
DEC03-186       ------------------------------------------------------------   0
DEC07-98        ------------------------------------------------------------   0
APR17-291       ------------------------------------------------------------   0


DEC02-76        CAACAGGAACACTGATGCTTTCAGAAGAACAATCTCAAAAAACTGAGGAAAACTTTAAAC   230
CLR03-38395     ------------------------------------------------------------   0
CLR01-43699     CAACAGGAACACTGATGCTTTCAGAAGAACAATCTCAAAAAACTGAGGAAAACTTTAAAC   1967
APR16-68        ------------------------------------------------------------   0
APR18-62        ------------------------------------------------------------   0
APR19-43        ------------------------------------------------------------   0
APR20-70        ------------------------------------------------------------   0
DEC10-249       ------------------------------------------------------------   0
DEC08-241       ------------------------------------------------------------   0
DEC06-81        ------------------------------------------------------------   0
DEC04-13db      CAACAGGAACACTGATGCTTTCAGAAGAACAATCTCAAAAAACTGAGGAAAACTTTAAAC   6900
MAY17-11        CAACAGGAACACTGATGCTTTCAGAAGAACAATCTCAAAAAACTGAGGAAAACTTTAAAC   1554
MAY15-78        ------------------------------------------------------------   0
JUL05-102       ------------------------------------------------------------   0
JUL04-107       ------------------------------------------------------------   0
JUL03-55        ------------------------------------------------------------   0
JUL02-106       ------------------------------------------------------------   0
JUL01-85        ------------------------------------------------------------   0
DEC03-186       ------------------------------------------------------------   0
```

Figure S4

```
DEC07-98        ------------------------------------------------------------   0
APR17-291       ------------------------------------------------------------   0


DEC02-76        AATTAAAAGATTTAATTGAACCTATTAAATCAGGATATTTATTAATTGAAAAATTAATAA   290
CLR03-38395     ------------------------------------------------------------   0
CLR01-43699     AATTAAAAGATTTAATTGAACCTATTAAATCAGGATATTTATTAATTGAAAAATTAATAA   2027
APR16-68        ------------------------------------------------------------   0
APR18-62        ------------------------------------------------------------   0
APR19-43        ------------------------------------------------------------   0
APR20-70        ------------------------------------------------------------   0
DEC10-249       ------------------------------------------------------------   0
DEC08-241       ------------------------------------------------------------   0
DEC06-81        ------------------------------------------------------------   0
DEC04-13db      AATTAAAAGATTTAATTGAACCTATTAAATCAGGATATTTATTAATTGAAAAATTAATAA   6960
MAY17-11        AATTAAAAGATTTAATTGAACCTATTAAATCAGGATATTTATTAATTGAAAAATTAATAA   1614
MAY15-78        ------------------------------------------------------------   0
JUL05-102       ------------------------------------------------------------   0
JUL04-107       ------------------------------------------------------------   0
JUL03-55        ------------------------------------------------------------   0
JUL02-106       ------------------------------------------------------------   0
JUL01-85        ------------------------------------------------------------   0
DEC03-186       ------------------------------------------------------------   0
DEC07-98        ------------------------------------------------------------   0
APR17-291       ------------------------------------------------------------   0


DEC02-76        AAGACTCAAGACCACAAATACAGAGGAGGTATGAGCAATTGAAACAATTTACAAAATGGT   350
CLR03-38395     ------------------------------------------------------------   0
CLR01-43699     AAGACTCAAGACCACAAATACAGAGGAGGTATGAGCAATTGAAACAATTTACAAAATGGT   2087
APR16-68        ------------------------------------------------------------   0
APR18-62        ------------------------------------------------------------   0
APR19-43        ------------------------------------------------------------   0
APR20-70        ------------------------------------------------------------   0
DEC10-249       ------------------------------------------------------------   0
DEC08-241       ------------------------------------------------------------   0
DEC06-81        ------------------------------------------------------------   0
DEC04-13db      AAGACTCAAGACCACAAATACAGAGGAGGTATGAGCAATTGAAACAATTTACAAAATGGT   7020
MAY17-11        AAGACTCAAGACCACAAATACAGAGGAGGTATGAGCAATTGAAACAATTTACAAAATGGT   1674
MAY15-78        ------------------------------------------------------------   0
JUL05-102       ------------------------------------------------------------   0
JUL04-107       ------------------------------------------------------------   0
JUL03-55        ------------------------------------------------------------   0
JUL02-106       ------------------------------------------------------------   0
JUL01-85        ------------------------------------------------------------   0
DEC03-186       ------------------------------------------------------------   0
DEC07-98        ------------------------------------------------------------   0
APR17-291       ------------------------------------------------------------   0


DEC02-76        TCTTAATTGGGCTAGGTTGTATATTGATCTTTGCCATCCAAAGAAGCTTGAATTGAGTAT   410
CLR03-38395     ------------------------------------------------------------   0
CLR01-43699     TCTTAATTGGGCTAGGTTGTATATTGATCTTTGCCATCCAAAGAAGCTTGAATTGAGTAT   2147
APR16-68        ------------------------------------------------------------   0
APR18-62        ------------------------------------------------------------   0
APR19-43        ------------------------------------------------------------   0
APR20-70        ------------------------------------------------------------   0
DEC10-249       ------------------------------------------------------------   0
DEC08-241       ------------------------------------------------------------   0
DEC06-81        ------------------------------------------------------------   0
DEC04-13db      TCTTAATTGGGCTAGGTTGTATATTGATCTTTGCCATCCAAAGAAGCTTGAATTGAGTAT   7080
MAY17-11        TCTTAATTGGGCTAGGTTGTATATTGATCTTTGCCATCCAAAGAAGCTTGAATTGAGTAT   1734
MAY15-78        ------------------------------------------------------------   0
```

Figure S4

```
JUL05-102       ------------------------------------------------------------   0
JUL04-107       ------------------------------------------------------------   0
JUL03-55        ------------------------------------------------------------   0
JUL02-106       ------------------------------------------------------------   0
JUL01-85        ------------------------------------------------------------   0
DEC03-186       ------------------------------------------------------------   0
DEC07-98        ------------------------------------------------------------   0
APR17-291       ------------------------------------------------------------   0


DEC02-76        TCAGTTTAAGCCAAAAAATGAATACAGTAAATGCTTTAACAAGTATCTACAAGGATTACA   470
CLR03-38395     ------------------------------------------------------------   0
CLR01-43699     TCAGTTTAAGCCAAAAAATGAATACAGTAAATGCTTTAACAAGTATCTACAAGGATTACA   2207
APR16-68        ------------------------------------------------------------   0
APR18-62        ------------------------------------------------------------   0
APR19-43        ------------------------------------------------------------   0
APR20-70        ------------------------------------------------------------   0
DEC10-249       ------------------------------------------------------------   0
DEC08-241       ------------------------------------------------------------   0
DEC06-81        ------------------------------------------------------------   0
DEC04-13db      TCAGTTTAAGCCAAAAAATGAATACAGTAAATGCTTTAACAAGTATCTACAAGGATTACA   7140
MAY17-11        TCAGTTTAAGCCAAAAAATGAATACAGTAAATGCTTTAACAAGTATCTACAAGGATTACA   1794
MAY15-78        ------------------------------------------------------------   0
JUL05-102       ------------------------------------------------------------   0
JUL04-107       ------------------------------------------------------------   0
JUL03-55        ------------------------------------------------------------   0
JUL02-106       ------------------------------------------------------------   0
JUL01-85        ------------------------------------------------------------   0
DEC03-186       ------------------------------------------------------------   0
DEC07-98        ------------------------------------------------------------   0
APR17-291       ------------------------------------------------------------   0


DEC02-76        TTACTGGTTTAATAAAAACCAGAAAATACCATTAATAATTCCAGGATTCAGCTATAGTTA   530
CLR03-38395     ------------------------------------------------------------   0
CLR01-43699     TTACTGGTTTAATAAAAACCAGAAAATACCATTAATAATTCCAGGATTCAGCTATAGTTA   2267
APR16-68        ------------------------------------------------------------   0
APR18-62        ------------------------------------------------------------   0
APR19-43        ------------------------------------------------------------   0
APR20-70        ------------------------------------------------------------   0
DEC10-249       ------------------------------------------------------------   0
DEC08-241       ------------------------------------------------------------   0
DEC06-81        ------------------------------------------------------------   0
DEC04-13db      TTACTGGTTTAATAAAAACCAGAAAATACCATTAATAATTCCAGGATTCAGCTATAGTTA   7200
MAY17-11        TTACTGGTTTAATAAAAACCAGAAAATACCATTAATAATTCCAGGATTCAGCTATAGTTA   1854
MAY15-78        ------------------------------------------------------------   0
JUL05-102       ------------------------------------------------------------   0
JUL04-107       ------------------------------------------------------------   0
JUL03-55        ------------------------------------------------------------   0
JUL02-106       ------------------------------------------------------------   0
JUL01-85        ------------------------------------------------------------   0
DEC03-186       ------------------------------------------------------------   0
DEC07-98        ------------------------------------------------------------   0
APR17-291       ------------------------------------------------------------   0


DEC02-76        TAATTTCCAACATCATGGTGGAAATCTTAACTACAAATATAATTTCAATTATAAAATGAA   590
CLR03-38395     ------------------------------------------------------------   0
CLR01-43699     TAATTTCCAACATCATGGTGGAAATCTTAACTACAAATATAATTTCAATTATAAAATGAA   2327
APR16-68        ------------------------------------------------------------   0
APR18-62        ------------------------------------------------------------   0
APR19-43        ------------------------------------------------------------   0
APR20-70        ------------------------------------------------------------   0
```

```
DEC10-249      ------------------------------------------------------------   0
DEC08-241      ------------------------------------------------------------   0
DEC06-81       ------------------------------------------------------------   0
DEC04-13db     TAATTTCCAACATCATGGTGGAAATCTTAACTACAAATATAATTTCAATTATAAAATGAA   7260
MAY17-11       TAATTTCCAACATCATGGTGGAAATCTTAACTACAAATATAATTTCAATTATAAAATGAA   1914
MAY15-78       ------------------------------------------------------------   0
JUL05-102      ------------------------------------------------------------   0
JUL04-107      ------------------------------------------------------------   0
JUL03-55       ------------------------------------------------------------   0
JUL02-106      ------------------------------------------------------------   0
JUL01-85       ------------------------------------------------------------   0
DEC03-186      ------------------------------------------------------------   0
DEC07-98       ------------------------------------------------------------   0
APR17-291      ------------------------------------------------------------   0


DEC02-76       ACAACAGGAAATGGATTTCAAACAATTGAATCCACTCGCTAATGAAATTCAGGAACTATT   650
CLR03-38395    ------------------------------------------------------------   0
CLR01-43699    ACAACAGGAAATGGATTTCAAACAATTGAATCCACTCGCTAATGAAATTCAGGAACTATT   2387
APR16-68       ------------------------------------------------------------   0
APR18-62       ------------------------------------------------------------   0
APR19-43       ------------------------------------------------------------   0
APR20-70       ------------------------------------------------------------   0
DEC10-249      ------------------------------------------------------------   0
DEC08-241      ------------------------------------------------------------   0
DEC06-81       ------------------------------------------------------------   0
DEC04-13db     ACAACAGGAAATGGATTTCAAACAATTGAATCCACTCGCTAATGAAATTCAGGAACTATT   7320
MAY17-11       ACAACAGGAAATGGATTTCAAACAATTGAATCCACTCGCTAATGAAATTCAGGAACTATT   1974
MAY15-78       ------------------------------------------------------------   0
JUL05-102      ------------------------------------------------------------   0
JUL04-107      ------------------------------------------------------------   0
JUL03-55       ------------------------------------------------------------   0
JUL02-106      ------------------------------------------------------------   0
JUL01-85       ------------------------------------------------------------   0
DEC03-186      ------------------------------------------------------------   0
DEC07-98       ------------------------------------------------------------   0
APR17-291      ------------------------------------------------------------   0


DEC02-76       AAGACAAGGAATTATTAATAATGAACAATTAACAGAAGCTTTAAGATATTTAAAATATCT   710
CLR03-38395    ------------------------------------------------------------   0
CLR01-43699    AAGACAAGGAATTATTAATAATGAACAATTAACAGAAGCTTTAAGATATTTAAAATATCT   2447
APR16-68       ------------------------------------------------------------   0
APR18-62       ------------------------------------------------------------   0
APR19-43       ------------------------------------------------------------   0
APR20-70       ------------------------------------------------------------   0
DEC10-249      ------------------------------------------------------------   0
DEC08-241      ------------------------------------------------------------   0
DEC06-81       ------------------------------------------------------------   0
DEC04-13db     AAGACAAGGAATTATTAATAATGAACAATTAACAGAAGCTTTAAGATATTTAAAATATCT   7380
MAY17-11       AAGACAAGGAATTATTAATAATGAACAATTAACAGAAGCTTTAAGATATTTAAAATATCT   2034
MAY15-78       ------------------------------------------------------------   0
JUL05-102      ------------------------------------------------------------   0
JUL04-107      ------------------------------------------------------------   0
JUL03-55       ------------------------------------------------------------   0
JUL02-106      ------------------------------------------------------------   0
JUL01-85       ------------------------------------------------------------   0
DEC03-186      ------------------------------------------------------------   0
DEC07-98       ------------------------------------------------------------   0
APR17-291      ------------------------------------------------------------   0


DEC02-76       TTCAGAGCACACTAATGAAAGACAAAAATTAAGCTATGATGAATTGAATAAACTATTCAA   770
```

Figure S4

```
CLR03-38395     ------------------------------------------------------------   0
CLR01-43699     TTCAGAGCACACTAATGAAAGACAAAAATTAAGCTATGATGAATTGAATAAACTATTCAA   2507
APR16-68        ------------------------------------------------------------   0
APR18-62        ------------------------------------------------------------   0
APR19-43        -----------------------------TAAGCTATGATGAATTGAATAAACTATTCAA   31
APR20-70        ------------------------------------------------------------   0
DEC10-249       ------------------------------------------------------------   0
DEC08-241       ------------------------------------------------------------   0
DEC06-81        ------------------------------------------------------------   0
DEC04-13db      TTCAGAGCACACTAATGAAAGACAAAAATTAAGCTATGATGAATTGAATAAACTATTCAA   7440
MAY17-11        TTCAGAGCACACTAATGAAAGACAAAAATTAAGCTATGATGAATTGAATAAACTATTCAA   2094
MAY15-78        ------------------------------------------------------------   0
JUL05-102       ------------------------------------------------------------   0
JUL04-107       ------------------------------------------------------------   0
JUL03-55        ------------------------------------------------------------   0
JUL02-106       ------------------------------------------------------------   0
JUL01-85        ------------------------------------------------------------   0
DEC03-186       ------------------------------------------------------------   0
DEC07-98        ------------------------------------------------------------   0
APR17-291       ------------------------------------------------------------   0


DEC02-76        CATATTTGGAGAAAGAATTGATAAAAGTATCAAAGATTTCAAGGAAGAAGTTGAAAGACA   830
CLR03-38395     ------------------------------------------------------------   0
CLR01-43699     CATATTTGGAGAAAGAATTGATAAAAGTATCAAAGATTTCAAGGAAGAAGTTGAAAGACA   2567
APR16-68        ------------------------------------------------------------   0
APR18-62        ------------------------------------------------------------   0
APR19-43        CATATTTGGAGAAAGAATTGATAAAAGTATCAAAGATTTCAAGGAAGAAGTTGAAAGACA   91
APR20-70        ------------------------------------------------------------   0
DEC10-249       ------------------------------------------------------------   0
DEC08-241       ------------------------------------------------------------   0
DEC06-81        ------------------------------------------------------------   0
DEC04-13db      CATATTTGGAGAAAGAATTGATAAAAGTATCAAAGATTTCAAGGAAGAAGTTGAAAGACA   7500
MAY17-11        CATATTTGGAGAAAGAATTGATAAAAGTATCAAAGATTTCAAGGAAGAAGTTGAAAGACA   2154
MAY15-78        ------------------------------------------------------------   0
JUL05-102       ------------------------------------------------------------   0
JUL04-107       ------------------------------------------------------------   0
JUL03-55        ------------------------------------------------------------   0
JUL02-106       ------------------------------------------------------------   0
JUL01-85        ------------------------------------------------------------   0
DEC03-186       ------------------------------------------------------------   0
DEC07-98        ------------------------------------------------------------   0
APR17-291       ------------------------------------------------------------   0


DEC02-76        GTTTAAAAATTTAACTCAAGATAAAAAAGATATTCAAGAAATAAAAGAATTATTAAAAAA   890
CLR03-38395     ------------------------------------------------------------   0
CLR01-43699     GTTTAAAAATTTAACTCAAGATAAAAAAGATATTCAAGAAATAAAAGAATTATTAAAAAA   2627
APR16-68        ------------------------------------------------------------   0
APR18-62        ----------------------------------CAAGAAATAAAAGAATTATTAAAAAA   26
APR19-43        GTTTAAAAATTTAACTCAAGATAAAAAAGATATTCAAGAAATAAAAGAATTATTAAAAAA   151
APR20-70        ------------------------------------------------------------   0
DEC10-249       ------------------------------------------------------------   0
DEC08-241       ------------------------------------------------------------   0
DEC06-81        ------------------------AAAACATATTCAAGAAATAACAGAATTATTAAAAAA   36
DEC04-13db      GTTTAAAAATTTAACTCAAGATAAAAAAGATATTCAAGAAATAAAAGAATTATTAAAAAA   7560
MAY17-11        GTTTAAAAATTTAACTCAAGATAAAAAAGATATTCAAGAAATAAAAGAATTATTAAAAAA   2214
MAY15-78        ------------------------------------------------------------   0
JUL05-102       ------------------------------------------------------------   0
JUL04-107       ------------------------------------------------------------   0
JUL03-55        ------------------------------------------------------------   0
JUL02-106       ------------------------------------------------------------   0
JUL01-85        ------------------------------------------------------------   0
```

```
DEC03-186       ------------------------------------------------------------   0
DEC07-98        ------------------------------------------------------------   0
APR17-291       ------------------------------------------------------------   0


DEC02-76        TAATAATCAGGATCTTGAAGAAATAAAGAAAAGACTTTCAGCAGTAGAAAAGAAATTATG   950
CLR03-38395     --------------------------------------------TAGAAAAGAAATTATG   16
CLR01-43699     TAATAATCAGGATCTTGAAGAAATAAAGAAAAGACTTTCAGCAGTAGAAAAGAAATTATG   2687
APR16-68        ----------------GAAGAAATAAAGAAAAGACTTTCAGCAGTAGAAAAGAAATTATG   44
APR18-62        TAATAATCAGGATCTTGAAGAAATAAAGAAAAGACTTTCAGCAGTAGAAAAGAAATTATG   86
APR19-43        TAATAATCAGGATCTTGAAGAAATAAAGAAAAGACTTTCAGCAGTAGAAAAGAAATTATG   211
APR20-70        ------------------------------------------------------------   0
DEC10-249       ------------------------------------------------------------   0
DEC08-241       ------------------------------------------------------------   0
DEC06-81        TAATAATCAGGATCTTGAAGAAATAAAGAAAAGACTTTCAGCAGTAGAAAAGAAATTATG   96
DEC04-13db      TAATAATCAGGATCTTGAAGAAATAAAGAAAAGACTTTCAGCAGTAGAAAAGAAATTATG   7620
MAY17-11        TAATAATCAGGATCTTGAAGAAATAAAGAAACGACTTTCAGCAGTAGAAAAGAAATTATG   2274
MAY15-78        ------------------------------------------------------------   0
JUL05-102       ------------------------------------------------------------   0
JUL04-107       ------------------------------------------------------------   0
JUL03-55        ------------------------------------------------------------   0
JUL02-106       ------------------------------------------------------------   0
JUL01-85        -----TTCAGGATCTTGAAGAAATAAAGAAAAGACTTTCAGCAGTAGAAAAGAAATTATG   55
DEC03-186       ------------------------------------------------------------   0
DEC07-98        ------------------------------------------------------------   0
APR17-291       ------------------------------------------------------------   0


DEC02-76        AGTACTAATTCAGAAATTAGTACAAACATAGGAAATAATCTCGATAACGAGGTTATAATC   1010
CLR03-38395     AGTACTAATTCAGAAATTAGTACAAACATAGGAAATAATCTCGATAACGAGGTTATAATC   76
CLR01-43699     AGTACTAATTCAGAAATTAGTACAAACATAGGAAATAATCTCGATAACGAGGTTATAATC   2747
APR16-68        AGTACTAATTCAGAAATTAGTACAAACATAGGAAATAATCTCGATAACGAGGTTATAATC   104
APR18-62        AGTACTAATTCAGAAATTAGTACAAACATAGGAAATAATCTCGATAACGAGGTTATAATC   146
APR19-43        AGTACTAATTCAGAAATTAGTACAAACATAGGAAATAATCTCGATAACGAGGTTATAATC   271
APR20-70        ------------------------------------------------------------   0
DEC10-249       ------------------------------------------------------------   0
DEC08-241       ------------------------------------------------------------   0
DEC06-81        AGTACTAATTCAGAAATTAGTACAAACATAGGAAATAATCTCGATAACGAGGTTATAATC   156
DEC04-13db      AGTACTAATTCAGAAATTAGTACAAACATAGGAAATAATCTCGATAACGAGGTTATAATC   7680
MAY17-11        AGTACTAATTCAGAAATTAGTACAAACATAGGAAATAATCTCGATAACGAGGTTATAATC   2334
MAY15-78        ------------------------------------------------------------   0
JUL05-102       ------------------------------------------------------------   0
JUL04-107       ------------------------------------------------------------   0
JUL03-55        --------------------------------------------------GGTTATTATC   10
JUL02-106       ------------------------------------------------------------   0
JUL01-85        AGTACTAATTCAGAAATTAGTACAAACATAGGAAATAATCTCGATAACGAGGTTATAATC   115
DEC03-186       ------------------------------------------------------------   0
DEC07-98        ------------------------------------------------------------   0
APR17-291       ------------------------------------------------------------   0


DEC02-76        AGGAATATTGATGACAATGAAACCTATCAAAGAGAAATATTAGTTAACGCTAATAAATTA   1070
CLR03-38395     AGGAATATTGATGACAATGAAACCTATCAAAGAGAAATATTAGTTAACGCTAATAAATTA   136
CLR01-43699     AGGAATATTGATGACAATGAAACCTATCAAAGAGAAATATTAGTTAACGCTAATAAATTA   2807
APR16-68        AGGAATATTGATGACAATGAAACCTATCAAAGAGAAATATTAGTTAACGCTAATAAATTA   164
APR18-62        AGGAATATTGATGACAATGAAACCTATCAAAGAGAAATATTAGTTAACGCTAATAAATTA   206
APR19-43        AGGAATATTGATGACAATGAAACCTATCAAAGAGAAATATTAGTTAACGCTAATAAATTA   331
APR20-70        ---------GATGACAATGAAACCTATCAAAGAGAAATATTAGTTAACGCTAATAAATTA   51
DEC10-249       ------------------------------------------------------------   0
DEC08-241       ------------------------------------------------------------   0
DEC06-81        AGGAATATTGATGACAATGAAACCTATCAAAGAGAAATATTAGTTAACGCTAATAAATTA   216
DEC04-13db      AGGAATATTGATGACAATGAAACCTATCAAAGAGAAATATTAGTTAACGCTAATAAATTA   7740
MAY17-11        AGGAATATTGATGACAATGAAACCTATCAAAGAGAAATATTAGTTAACGCTAATAAATTA   2394
```

Figure S4

```
MAY15-78        ------------------------------------------------------------   0
JUL05-102       ------------------------------------------------------------   0
JUL04-107       ------------------------------------------------------------   0
JUL03-55        AGGAACATAGATGATAATGAAACCTATCAAAGAGAAATATTAGTTAATGCTAATAAATTA   70
JUL02-106       ------------------------------------------------------------   0
JUL01-85        AGGAATATTGATGACAATGAAACCTATCAAAGAGAAATATTAGTTAACGCTAATAAATTA   175
DEC03-186       ------------------------------------------------------------   0
DEC07-98        ------------------------------------------------------------   0
APR17-291       ------------------------------------------------------------   0


DEC02-76        TCTAAGATAGAGAAAAAAGGAAAGATAACTCTTGAAGATGGTCAGGATATTAAGGATAAT   1130
CLR03-38395     TCTAAGATAGAGAAAAAAGGAAAGATAACTCTTGAAGATGGTCAGGATATTAAGGATAAT   196
CLR01-43699     TCTAAGATAGAGAAAAAAGGAAAGATAACTCTTGAAGATGGTCAGGATATTAAGGATAAT   2867
APR16-68        TCTAAGATAGAGAAAAAAGGAAAGATAACTCTTGAAGATGGTCAGGATATTAAGGATAAT   224
APR18-62        TCTAAGATAGAGAAAAAAGGAAAGATAACTCTTGAAGATGGTCAGGATATTAAGGATAAT   266
APR19-43        TCTAAGATAGAGAAAAAAGGAAAGATAACTCTTGAAGATGGTCAGGATATTAAGGATAAT   391
APR20-70        TCTAAGATAGAGAAAAAAGGAAAGATAACTCTTGAAGATGGTCAGGATATTAAGGATAAT   111
DEC10-249       ------------------------------------------------------------   0
DEC08-241       ------------------------------------------------------------   0
DEC06-81        TCTAAGATAGAGAAAAAAGGAAAGATAACTCTTGAAGATGGTCAGGATATTAAGGATAAT   276
DEC04-13db      TCTAAGATAGAGAAAAAAGGAAAGATAACTCTTGAAGATGGTCAGGATATTAAGGATAAT   7800
MAY17-11        TCTAAGATAGAGAAAAAAGGAAAGATAACTCTTGAAGATGGTCAGGATATTAAGGATAAT   2454
MAY15-78        ----------------------------CTCTTGAAGATGGTCAGGATATTAAGGATAAT   32
JUL05-102       ---------------------------------------GGTCAGGATATTAAGGATAAT   21
JUL04-107       ----------------------------CTCTTGAAGATGGTCAGGATATTAAGGATAAT   32
JUL03-55        TCTAAGATAGAGAAAAAAGGAAAAATAACTCTAGAAGACGGTCAGGATATTAAGGATAAT   130
JUL02-106       ---------------------------------------GGTCAGGATATTAAGGATAAT   21
JUL01-85        TCTAAGATAGAGAAAAAAGGAAAGATAACTCTTGAAGATGGTCAGGATATTAAGGATAAT   235
DEC03-186       ------------------------------------------------------------   0
DEC07-98        ------------------------------------------------------------   0
APR17-291       ------------------------------------------------------------   0


DEC02-76        CTAAGTCAACAATTCAAAAGATTGTTTGACAGAGAAAACATACTATTTTTAGGAAAATTT   1190
CLR03-38395     CTAAGTCAACAATTCAAAAGATTGTTTGACAGAGAAAACATACTATTTTTAGGAAAATTT   256
CLR01-43699     CTAAGTCAACAATTCAAAAGATTGTTTGACAGAGAAAACATACTATTTTTAGGAAAATTT   2927
APR16-68        CTAAGTCAACAATTCAAAAGATTGTTTGACAGAGAAAACATACTATTTTTAGGAAAATTT   284
APR18-62        CTAAGTCAACAATTCAAAAGATTGTTTGACAGAGAAAACATACTATTTTTAGGAAAATTT   326
APR19-43        CTAAGTCAACAATTCAAAAGATTGTTTGACAGAGAAAACATACTATTTTTAGGAAAATTT   451
APR20-70        CTAAGTCAACAATTCAAAAGATTGTTTGACAGAGAAAACATACTATTTTTAGGAAAATTT   171
DEC10-249       ------------------------------------------------------------   0
DEC08-241       ------------------------------------------------------------   0
DEC06-81        CTAAGTCAACAATTCAAAAGATTGTTTGACAGAGAAAACATACTATTTTTAGGAAAATTT   336
DEC04-13db      CTAAGTCAACAATTCAAAAGATTGTTTGACAGAGAAAACATACTATTTTTAGGAAAATTT   7860
MAY17-11        CTAAGTCAACAATTCAAAAGATTGTTTGACAGAGAAAACATACTATTTTTAGGAAAATTT   2514
MAY15-78        CTAAGTCAACAATTCAAAAGATTGTTTGACAGAGAAAACATACTATTTTTAGGAAAATTT   92
JUL05-102       CTAAGTCAACAATTCAAAAGATTGTTTGACAGAGAAAACATACTATTTTTAGGAAAATTT   81
JUL04-107       CTAAGTCAACAATTCAAAAGATTGTTTGACAGAGAAAACATACTATTTTTAGGAAAATTT   92
JUL03-55        CTAAGTCAACAATTCAAAAGATTGTTTGACAGAGAAAACATACTATTTTTAGGAAAATTT   190
JUL02-106       CTAAGTCAACAATTCAAAAGATTGTTTGACAGAGAAAACATACTATTTTTAGGAAAATTT   81
JUL01-85        CTAAGTCAACAATTCAAAAGATTGTTTGACAGAGAAAACATACTATTTTTAGGAAAATTT   295
DEC03-186       ------------------------------------------------------------   0
DEC07-98        ------------------------------------------------------------   0
APR17-291       ------------------------------------------------------------   0


DEC02-76        GTAGAGGAACTTCCTATACAAATTAAGCAAGCTACAGGAAACACTGTGTTACCTTTCATA   1250
CLR03-38395     GTAGAGGAACTTCCTATACAAATTAAGCAAGCTACAGGAAACACTGTGTTACCTTTCATA   316
CLR01-43699     GTAGAGGAACTTCCTATACAAATTAAGCAAGCTACAGGAAACACTGTGTTACCTTTCATA   2987
APR16-68        GTAGAGGAACTTCCTATACAAATTAAGCAAGCTACAGGAAACACTGTGTTACCTTTCATA   344
APR18-62        GTAGAGGAACTTCCTATACAAATTAAGCAAGCTACAGGAAACACTGTGTTACCTTTCATA   386
APR19-43        GTAGAGGAACTTCCTATACAAATTAAGCAAGCTACAGGAAACACTGTGTTACCTTTCATA   511
```

Figure S4

| | | |
|---|---|---|
| APR20-70 | GTAGAGGAACTTCCTATACAAATTAAGCAAGCTACAGGAAACACTGTGTTACCTTTCATA | 231 |
| DEC10-249 | ------------------------------------------------------------ | 0 |
| DEC08-241 | ------------------------------------------------------------ | 0 |
| DEC06-81 | GTAGAGGAACTTCCTATACAAATTAAGCAAGCTACAGGAAACACTGTGTTACCTTTCATA | 396 |
| DEC04-13db | GTAGAGGAACTTCCTATACAAATTAAGCAAGCTACAGGAAACACTGTGTTACCTTTCATA | 7920 |
| MAY17-11 | GTAGAGGAACTTCCTATACAAATTAAGCAAGCTACAGGAAACACTGTGTTACCTTTCATA | 2574 |
| MAY15-78 | GTAGAGGAACTTCCTATACAAATTAAGCAAGCTACAGGAAACACTGTGTTACCTTTCATA | 152 |
| JUL05-102 | GTAGAGGAACTTCCTATACAAATTAAGCAAGCTACAGGAAACACTGTGTTACCTTTCATA | 141 |
| JUL04-107 | GTAGAGGAACTTCCTATACAAATTAAGCAAGCTACAGGAAACACTGTGTTACCTTTCATA | 152 |
| JUL03-55 | GTAGAGGAACTTCCTATACAAATTAAGCAAGCTACAGGAAACACTGTGTTACCTTTCATA | 250 |
| JUL02-106 | GTAGAGGAACTTCCTATACAAATTAAGCAAGCTACAGGAAACACTGTGTTACCTTTCATA | 141 |
| JUL01-85 | GTAGAGGAACTTCCTATACAAATTAAGCAAGCTACAGGAAACACTGTGTTACCTTTCATA | 355 |
| DEC03-186 | ------------------------------------------------------------ | 0 |
| DEC07-98 | ------------------------------------------------------------ | 0 |
| APR17-291 | ------------------------------------------------------------ | 0 |

| | | |
|---|---|---|
| DEC02-76 | ACACAGCAAAGTTTAGAGGAAAAATTATCAAAGATACCTAAAAAGGATAGGACCAAAATA | 1310 |
| CLR03-38395 | ACACAGCAAAGTTTAGAGGAAAAATTATCAAAGATACCTAAAAAGGATAGGACCAAAATA | 376 |
| CLR01-43699 | ACACAGCAAAGTTTAGAGGAAAAATTATCAAAGATACCTAAAAAGGATAGGACCAAAATA | 3047 |
| APR16-68 | ACACAGCAAAGTTTAGAGGAAAAATTATCAAAGATACCTAAAAAGGATAGGACCAAAATA | 404 |
| APR18-62 | ACACAGCAAAGTTTAGAGGAAAAATTATCAAAGATACCTAAAAAGGATAGGACCAAAATA | 446 |
| APR19-43 | ACACAGCAAAGTTTAGAGGAAAAATTATCAAAGATACCTAAAAAGGATAGGACCAAAATA | 571 |
| APR20-70 | ACACAGCAAAGTTTAGAGGAAAAATTATCAAAGATACCTAAAAAGGATAGGACCAAAATA | 291 |
| DEC10-249 | ------------------------------------------------------------ | 0 |
| DEC08-241 | ------------------------------------------------------------ | 0 |
| DEC06-81 | ACACAGCAAAGTTTAGAGGAAAAATTATCAAAGATACCTAAAAAGGATAGGACCAAAATA | 456 |
| DEC04-13db | ACACAGCAAAGTTTAGAGGAAAAATTATCAAAGATACCTAAAAAGGATAGGACCAAAATA | 7980 |
| MAY17-11 | ACACAGCAAAGTTTAGAGGAAAAATTATCAAAGATACCTAAAAAGGATAGGACCAAAATA | 2634 |
| MAY15-78 | ACACAGCAAAGTTTAGAGGAAAAATTATCAAAGATACCTAAAAAGGATAGGACCAAAATA | 212 |
| JUL05-102 | ACACAGCAAAGTTTAGAGGAAAAATTATCAAAGATACCTAAAAAGGATAGGACCAAAATA | 201 |
| JUL04-107 | ACACAGCAAAGTTTAGAGGAAAAATTATCAAAGATACCTAAAAAGGATAGGACCAAAATA | 212 |
| JUL03-55 | ACACAGCAAAGTTTAGAGGAAAAATTATCAAAGATACCTAAAAAGGATAGGACCAAAATA | 310 |
| JUL02-106 | ACACAGCAAAGTTTAGAGGAAAAATTATCAAAGATACCTAAAAAGGATAGGACCAAAATA | 201 |
| JUL01-85 | ACACAGCAAAGTTTAGAGGAAAAATTATCAAAGATACCTAAAAAGGATAGGACCAAAATA | 415 |
| DEC03-186 | ------------------------------------------------------------ | 0 |
| DEC07-98 | ------------------------------------------------------------ | 0 |
| APR17-291 | ------------------------------------------------------------ | 0 |

| | | |
|---|---|---|
| DEC02-76 | AAATATATACATTTTGGAAAAGCACAAATAATAATAAAACCAACGATAAAATCAGGAATA | 1370 |
| CLR03-38395 | AAATATATACATTTTGGAAAAGCACAAATAATAATAAAACCAACGATAAAATCAGGAATA | 436 |
| CLR01-43699 | AAATATATACATTTTGGAAAAGCACAAATAATAATAAAACCAACGATAAAATCAGGAATA | 3107 |
| APR16-68 | AAATATATACATTTTGGAAAAGCACAAATAATAATAAAACCAACGATAAAATCAGGAATA | 464 |
| APR18-62 | AAATATATACATTTTGGAAAAGCACAAATAATAATAAAACCAACGATAAAATCAGGAATA | 506 |
| APR19-43 | AAATATATACATTTTGGAAAAGCACAAATAATAATAAAACCAACGATAAAATCAGGAATA | 631 |
| APR20-70 | AAATATATACATTTTGGAAAAGCACAAATAATAATAAAACCAACGATAAAATCAGGAATA | 351 |
| DEC10-249 | ------------------------------------------------------------ | 0 |
| DEC08-241 | ------------------------------------------------------------ | 0 |
| DEC06-81 | AAATATATACATTTTGGAAAAGCACAAATAATAATAAAACCAACGATAAAATCAGGAATA | 516 |
| DEC04-13db | AAATATATACATTTTGGAAAAGCACAAATAATAATAAAACCAACGATAAAATCAGGAATA | 8040 |
| MAY17-11 | AAATATATACATTTTGGAAAAGCACAAATAATAATAAAACCAACGATAAAATCAGGAATA | 2694 |
| MAY15-78 | AAATATATACATTTTGGAAAAGCACAAATAATAATAAAACCAACGATAAAATCAGGAATA | 272 |
| JUL05-102 | AAATATATACATTTTGGAAAAGCACAAATAATAATAAAACCAACGATAAAATCAGGAATA | 261 |
| JUL04-107 | AAATATATACATTTTGGAAAAGCACAAATAATAATAAAACCAACGATAAAATCAGGAATA | 272 |
| JUL03-55 | AAATATATACATTTTGGAAAAGCACAAATAATAATAAAACCAACGATAAAATCAGGAATA | 370 |
| JUL02-106 | AAATATATACATTTTGGAAAAGCACAAATAATAATAAAACCAACGATAAAATCAGGAATA | 261 |
| JUL01-85 | AAATATATACATTTTGGAAAAGCACAAATAATAATAAAACCAACGATAAAATCAGGAATA | 475 |
| DEC03-186 | ------------------------------------------------------------ | 0 |
| DEC07-98 | ------------------------------------------------------------ | 0 |
| APR17-291 | ------------------------------------------------------------ | 0 |

Figure S4

```
DEC02-76        GACACACCCATTGAAATAATAGTGTATGACAGGAGAATAACGAGTAATAATATTAATGAA   1430
CLR03-38395     GACACACCCATTGAAATAATAGTGTATGACAGGAGAATAACGAGTAATAATATTAATGAA   496
CLR01-43699     GACACACCCATTGAAATAATAGTGTATGACAGGAGAATAACGAGTAATAATATTAATGAA   3167
APR16-68        GACACACCCATTGAAATAATAGTGTATGACAGGAGAATAACGAGTAATAATATTAATGAA   524
APR18-62        GACACACCCATTGAAATAATAGTGTATGACAGGAGAATAACGAGTAATAATATTAATGAA   566
APR19-43        GACACACCCATTGAAATAATAGTGTATGACAGGAGAATAACGAGTAATAATATTAATGAA   691
APR20-70        GACACACCCATTGAAATAATAGTGTATGACAGGAGAATAACGAGTAATAATATTAATGAA   411
DEC10-249       ------------------------------------------------------------   0
DEC08-241       ------------------------------------------------------------   0
DEC06-81        GACACACCCATTGAAATAATAGTGTATGACAGGAGAATAACGAGTAATAATATTAATGAA   576
DEC04-13db      GACACACCCATTGAAATAATAGTGTATGACAGGAGAATAACGAGTAATAATATTAATGAA   8100
MAY17-11        GACACACCCATTGAAATAATAGTGTATGACAGGAGAATAACGAGTAATAATATTAATGAA   2754
MAY15-78        GACACACCCATTGAAATAATAGTGTATGACAGGAGAATAACGAGTAATAATATTAATGAA   332
JUL05-102       GACACACCCATTGAAATAATAGTGTATGACAGGAGAATAACGAGTAATAATATTAATGAA   321
JUL04-107       GACACACCCATTGAAATAATAGTGTATGACAGGAGAATAACGAGTAATAATATTAATGAA   332
JUL03-55        GACACACCCATTGAAATAATAGTGTATGACAGGAGAATAACGAGTAATAATATTAATGAA   430
JUL02-106       GACACACCCATTGAAATAATAGTGTATGACAGGAGAATAACGAGTAATAATATTAATGAA   321
JUL01-85        GACACACCCATTGAAATAATAGTGTATGACAGGAGAATAACGAGTAATAATATTAATGAA   535
DEC03-186       ------------------------------------------------------------   0
DEC07-98        ------------------------------------------------------------   0
APR17-291       ------------------------------------------------------------   0


DEC02-76        ATAATAATAGGACGACTCGAAGGAAACTTAGGATATCCAGCAGTAAAATTTGATGTTAGC   1490
CLR03-38395     ATAATAATAGGACGACTCGAAGGAAACTTAGGATATCCAGCAGTAAAATTTGATGTTAGC   556
CLR01-43699     ATAATAATAGGACGACTCGAAGGAAACTTAGGATATCCAGCAGTAAAATTTGATGTTAGC   3227
APR16-68        ATAATAATAGGACGACTCGAAGGAAACTTAGGATATCCAGCAGTAAAATTTGATGTTAGC   584
APR18-62        ATAATAATAGGACGACTCGAAGGAAACTTAGGATATCCAGCAGTAAAATTTGATGTTAGC   626
APR19-43        ATAATAATAGGACGACTCGAAGGAAACTTAGGATATCCAGCAGTAAAATTTGATGTTAGC   751
APR20-70        ATAATAATAGGACGACTCGAAGGAAACTTAGGATATCCAGCAGTAAAATTTGATGTTAGC   471
DEC10-249       ------------------------------------------------------------   0
DEC08-241       ------------------------------------------------------------   0
DEC06-81        ATAATAATAGGACGACTCGAAGGAAACTTAGGATATCCAGCAGTAAAATTTGATGTTAGC   636
DEC04-13db      ATAATAATAGGACGACTCGAAGGAAACTTAGGATATCCAGCAGTAAAATTTGATGTTAGC   8160
MAY17-11        ATAATAATAGGACGACTCGAAGGAAACTTAGGATATCCAGCAGTAAAATTTGATGTTAGC   2814
MAY15-78        ATAATAATAGGACGACTCGAAGGAAACTTAGGATATCCAGCAGTAAAATTTGATGTTAGC   392
JUL05-102       ATAATAATAGGACGACTCGAAGGAAACTTAGGATATCCAGCAGTAAAATTTGATGTTAGC   381
JUL04-107       ATAATAATAGGACGACTCGAAGGAAACTTAGGATATCCAGCAGTAAAATTTGATGTTAGC   392
JUL03-55        ATAATAATAGGACGACTCGAAGGAAACTTAGGATATCCAGCAGTAAAATTTGATGTTAGC   490
JUL02-106       ATAATAATAGGACGACTCGAAGGAAACTTAGGATATCCAGCAGTAAAATTTGATGTTAGC   381
JUL01-85        ATAATAATAGGACGACTCGAAGGAAACTTAGGATATCCAGCAGTAAAATTTGATGTTAGC   595
DEC03-186       ------------------------------------------------------------   0
DEC07-98        ------------------------------------------------------------   0
APR17-291       ------------------------------------------------------------   0


DEC02-76        CTACAAATAGGAATTCCAATAATATCAAATTATCTAGGAAAGTCTATAGGATTAAGTTTC   1550
CLR03-38395     CTACAAATAGGAATTCCAATAATATCAAATTATCTAGGAAAGTCTATAGGATTAAGTTTC   616
CLR01-43699     CTACAAATAGGAATTCCAATAATATCAAATTATCTAGGAAAGTCTATAGGATTAAGTTTC   3287
APR16-68        CTACAAATAGGAATTCCAATAATATCAAATTATCTAGGAAAGTCTATAGGATTAAGTTTC   644
APR18-62        CTACAAATAGGAATTCCAATAATATCAAATTATCTAGGAAAGTCTATAGGATTAAGTTTC   686
APR19-43        CTACAAATAGGAATTCCAATAATATCAAATTATCTAGGAAAGTCTATAGGATTAAGTTTC   811
APR20-70        CTACAAATAGGAATTCCAATAATATCAAATTATCTAGGAAAGTCTATAGGATTAAGTTTC   531
DEC10-249       ------------------------------------------------------------   0
DEC08-241       ------------------------------------------------------------   0
DEC06-81        CTACAAATAGGAATTCCAATAATATCAAATTATCTAGGAAAGTCTATAGGATTAAGTTTC   696
DEC04-13db      CTACAAATAGGAATTCCAATAATATCAAATTATCTAGGAAAGTCTATAGGATTAAGTTTC   8220
MAY17-11        CTACAAATAGGAATTCCAATAATATCAAATTATCTAGGAAAGTCTATAGGATTAAGTTTC   2874
MAY15-78        CTACAAATAGGAATTCCAATAATATCAAATTATCTAGGAAAGTCTATAGGATTAAGTTTC   452
JUL05-102       CTACAAATAGGAATTCCAATAATATCAAATTATCTAGGAAAGTCTATAGGATTAAGTTTC   441
JUL04-107       CTACAAATAGGAATTCCAATAATATCAAATTATCTAGGAAAGTCTATAGGATTAAGTTTC   452
JUL03-55        CTACAAATAGGAATTCCAATAATATCAAATTATCTAGGAAAGTCTATAGGATTAAGTTTC   550
JUL02-106       CTACAAATAGGAATTCCAATAATATCAAATTATCTAGGAAAGTCTATAGGATTAAGTTTC   441
```

Figure S4

| | | |
|---|---|---|
| JUL01-85 | CTACAAATAGGAATTCCAATAATATCAAATTATCTAGGAAAGTCTATAGGATTAAGTTTC | 655 |
| DEC03-186 | ------------------------------------------------------------ | 0 |
| DEC07-98 | ------------------------------------------------------------ | 0 |
| APR17-291 | ------------------------------------------------------------ | 0 |

| | | |
|---|---|---|
| DEC02-76 | AGATACTTAAGACAAGACTTAATGAATCAGGATGATTACCCATTCAGCATTTTATATGCT | 1610 |
| CLR03-38395 | AGATACTTAAGACAAGACTTAATGAATCAGGATGATTACCCATTCAGCATTTTATATGCT | 676 |
| CLR01-43699 | AGATACTTAAGACAAGACTTAATGAATCAGGATGATTACCCATTCAGCATTTTATATGCT | 3347 |
| APR16-68 | AGATACTTAAGACAAGACTTAATGAATCAGGATGATTACCCATTCAGCATTTTATATGCT | 704 |
| APR18-62 | AGATACTTAAGACAAGACTTAATGAATCAGGATGATTACCCATTCAGCATTTTATATGCT | 746 |
| APR19-43 | AGATACTTAAGACAAGACTTAATGAATCAGGATGATTACCCATTCAGCATTTTATATGCT | 871 |
| APR20-70 | AGATACTTAAGACAAGACTTAATGAATCAGGATGATTACCCATTCAGCATTTTATATGCT | 591 |
| DEC10-249 | ------------------------------------------------------------ | 0 |
| DEC08-241 | ------------------------------------------------------------ | 0 |
| DEC06-81 | AGATACTTAAGACAAGACTTAATGAATCAGGATGATTACCCATTCAGCATTTTATATGCT | 756 |
| DEC04-13db | AGATACTTAAGACAAGACTTAATGAATCAGGATGATTACCCATTCAGCATTTTATATGCT | 8280 |
| MAY17-11 | AGATACTTAAGACAAGACTTAATGAATCAGGATGATTACCCATTCAGCATTTTATATGCT | 2934 |
| MAY15-78 | AGATACTTAAGACAAGACTTAATGAATCAGGATGATTACCCATTCAGCATTTTATATGCT | 512 |
| JUL05-102 | AGATACTTAAGACAAGACTTAATGAATCAGGATGATTACCCATTCAGCATTTTATATGCT | 501 |
| JUL04-107 | AGATACTTAAGACAAGACTTAATGAATCAGGATGATTACCCATTCAGCATTTTATATGCT | 512 |
| JUL03-55 | AGATACTTAAGACAAGACTTAATGAATCAGGATGATTACCCATTCAGCATTTTATATGCT | 610 |
| JUL02-106 | AGATACTTAAGACAAGACTTAATGAATCAGGATGATTACCCATTCAGCATTTTATATGCT | 501 |
| JUL01-85 | AGATACTTAAGACAAGACTTAATGAATCAGGATGATTACCCATTCAGCATTTTATATGCT | 715 |
| DEC03-186 | ------------------------------------------------------------ | 0 |
| DEC07-98 | ------------------------------------------------------------ | 0 |
| APR17-291 | ------------------------------------------------------------ | 0 |

| | | |
|---|---|---|
| DEC02-76 | GTAGGATATGGATTGAGTAATTCGCATTACTCAGTTAATTTCAAGACCAGCAACAAGATA | 1670 |
| CLR03-38395 | GTAGGATATGGATTGAGTAATTCGCATTACTCAGTTAATTTCAAGACCAGCAACAAGATA | 736 |
| CLR01-43699 | GTAGGATATGGATTGAGTAATTCGCATTACTCAGTTAATTTCAAGACCAGCAACAAGATA | 3407 |
| APR16-68 | GTAGGATATGGATTGAGTAATTCGCATTACTCAGTTAATTTCAAGACCAGCAACAAGATA | 764 |
| APR18-62 | GTAGGATATGGATTGAGTAATTCGCATTACTCAGTTAATTTCAAGACCAGCAACAAGATA | 806 |
| APR19-43 | GTAGGATATGGATTGAGTAATTCGCATTACTCAGTTAATTTCAAGACCAGCAACAAGATA | 931 |
| APR20-70 | GTAGGATATGGATTGAGTAATTCGCATTACTCAGTTAATTTCAAGACCAGCAACAAGATA | 651 |
| DEC10-249 | ------------------------------------------------------------ | 0 |
| DEC08-241 | ------------------------------------------------------------ | 0 |
| DEC06-81 | GTAGGATATGGATTGAGTAATTCGCATTACTCAGTTAATTTCAAGACCAGCAACAAGATA | 816 |
| DEC04-13db | GTAGGATATGGATTGAGTAATTCGCATTACTCAGTTAATTTCAAGACCAGCAACAAGATA | 8340 |
| MAY17-11 | GTAGGATATGGATTGAGTAATTCGCATTACTCAGTTAATTTCAAGACCAGCAACAAGATA | 2994 |
| MAY15-78 | GTAGGATATGGATTGAGTAATTCGCATTACTCAGTTAATTTCAAGACCAGCAACAAGATA | 572 |
| JUL05-102 | GTAGGATATGGATTGAGTAATTCGCATTACTCAGTTAATTTCAAGACCAGCAACAAGATA | 561 |
| JUL04-107 | GTAGGATATGGATTGAGTAATTCGCATTACTCAGTTAATTTCAAGACCAGCAACAAGATA | 572 |
| JUL03-55 | GTAGGATATGGATTGAGTAATTCGCATTACTCAGTTAATTTCAAGACCAGCAACAAGATA | 670 |
| JUL02-106 | GTAGGATATGGATTGAGTAATTCGCATTACTCAGTTAATTTCAAGACCAGCAACAAGATA | 561 |
| JUL01-85 | GTAGGATATGGATTGAGTAATTCGCATTACTCAGTTAATTTCAAGACCAGCAACAAGATA | 775 |
| DEC03-186 | ------------------------------------------------------------ | 0 |
| DEC07-98 | ------------------------------------------------------------ | 0 |
| APR17-291 | ------------------------------------------------------------ | 0 |

| | | |
|---|---|---|
| DEC02-76 | GAAATTGAACAAATATTTGCAGAAACAAGTACTAAGCTAATAGAAATCCCCAGAAGTCAT | 1730 |
| CLR03-38395 | GAAATTGAACAAATATTTGCAGAAACAAGTACTAAGCTAATAGAAATCCCCAGAAGTCAT | 796 |
| CLR01-43699 | GAAATTGAACAAATATTTGCAGAAACAAGTACTAAGCTAATAGAAATCCCCAGAAGTCAT | 3467 |
| APR16-68 | GAAATTGAACAAATATTTGCAGAAACAAGTACTAAGCTAATAGAAATCCCCAGAAGTCAT | 824 |
| APR18-62 | GAAATTGAACAAATATTTGCAGAAACAAGTACTAAGCTAATAGAAATCCCCAGAAGTCAT | 866 |
| APR19-43 | GAAATTGAACAAATATTTGCAGAAACAAGTACTAAGCTAATAGAAATCCCCAGAAGTCAT | 991 |
| APR20-70 | GAAATTGAACAAATATTTGCAGAAACAAGTACTAAGCTAATAGAAATCCCCAGAAGTCAT | 711 |
| DEC10-249 | ------------------------------------------------------------ | 0 |
| DEC08-241 | ------------------------------------------------------------ | 0 |
| DEC06-81 | GAAATTGAACAAATATTTGCAGAAACAAGTACTAAGCTAATAGAAATCCCCAGAAGTCAT | 876 |
| DEC04-13db | GAAATTGAACAAATATTTGCAGAAACAAGTACTAAGCTAATAGAAATCCCCAGAAGTCAT | 8400 |

```
MAY17-11       GAAATTGAACAAATATTTGCAGAAACAAGTACTAAGCTAATAGAAATCCCCAGAAGTCAT   3054
MAY15-78       GAAATTGAACAAATATTTGCAGAAACAAGTACTAAGCTAATAGAAATCCCCAGAAGTCAT   632
JUL05-102      GAAATTGAACAAATATTTGCAGAAACAAGTACTAAGCTAATAGAAATCCCCAGAAGTCAT   621
JUL04-107      GAAATTGAACAAATATTTGCAGAAACAAGTACTAAGCTAATAGAAATCCCCAGAAGTCAT   632
JUL03-55       GAAATTGAACAAATATTTGCAGAAACAAGTACTAAGCTAATAGAAATCCCCAGAAGTCAT   730
JUL02-106      GAAATTGAACAAATATTTGCAGAAACAAGTACTAAGCTAATAGAAATCCCCAGAAGTCAT   621
JUL01-85       GAAATTGAACAAATATTTGCAGAAACAAGTACTAAGCTAATAGAAATCCCCAGAAGTCAT   835
DEC03-186      ------------------------------------------------------------   0
DEC07-98       ------------------------------------------------------------   0
APR17-291      ------------------------------------------------------------   0


DEC02-76       TTTAGTTTACCTAAATTAACGAATGGAGACAGGATTGAGGAAACTTCTGAAAATATTTTC   1790
CLR03-38395    TTTAGTTTACCTAAATTAACGAATGGAGACAGGATTGAGGAAACTTCTGAAAATATTTTC   856
CLR01-43699    TTTAGTTTACCTAAATTAACGAATGGAGACAGGATTGAGGAAACTTCTGAAAATATTTTC   3527
APR16-68       TTTAGTTTACCTAAATTAACGAATGGAGACAGGATTGAGGAAACTTCTGAAAATATTTTC   884
APR18-62       TTTAGTTTACCTAAATTAACGAATGGAGACAGGATTGAGGAAACTTCTGAAAATATTTTC   926
APR19-43       TTTAGTTTACCTAAATTAACGAATGGAGACAGGATTGAGGAAACTTCTGAAAATATTTTC   1051
APR20-70       TTTAGTTTACCTAAATTAACGAATGGAGACAGGATTGAGGAAACTTCTGAAAATATTTTC   771
DEC10-249      ------------------------------------------------------------   0
DEC08-241      ------------------------------------------------------------   0
DEC06-81       TTTAGTTTACCTAAATTAACGAATGGAGACAGGATTGAGGAAACTTCTGAAAATATTTTC   936
DEC04-13db     TTTAGTTTACCTAAATTAACGAATGGAGACAGGATTGAGGAAACTTCTGAAAATATTTTC   8460
MAY17-11       TTTAGTTTACCTAAATTAACGAATGGAGACAGGATTGAGGAAACTTCTGAAAATATTTTC   3114
MAY15-78       TTTAGTTTACCTAAATTAACGAATGGAGACAGGATTGAGGAAACTTCTGAAAATATTTTC   692
JUL05-102      TTTAGTTTACCTAAATTAACGAATGGAGACAGGATTGAGGAAACTTCTGAAAATATTTTC   681
JUL04-107      TTTAGTTTACCTAAATTAACGAATGGAGACAGGATTGAGGAAACTTCTGAAAATATTTTC   692
JUL03-55       TTTAGTTTACCTAAATTAACGAATGGAGACAGGATTGAGGAAACTTCTGAAAATATTTTC   790
JUL02-106      TTTAGTTTACCTAAATTAACGAATGGAGACAGGATTGAGGAAACTTCTGAAAATATTTTC   681
JUL01-85       TTTAGTTTACCTAAATTAACGAATGGAGACAGGATTGAGGAAACTTCTGAAAATATTTTC   895
DEC03-186      ------------------------------------------------------------   0
DEC07-98       ------------------------------------------------------------   0
APR17-291      ------------------------------------------------------------   0


DEC02-76       AGAAATTATAATGATAGCACTGCACTTGTGCCAAAACAAGGATTTAGCTTAAATAGGAGT   1850
CLR03-38395    AGAAATTATAATGATAGCACTGCACTTGTGCCAAAACAAGGATTTAGCTTAAATAGGAGT   916
CLR01-43699    AGAAATTATAATGATAGCACTGCACTTGTGCCAAAACAAGGATTTAGCTTAAATAGGAGT   3587
APR16-68       AGAAATTATAATGATAGCACTGCACTTGTGCCAAAACAAGGATTTAGCTTAAATAGGAGT   944
APR18-62       AGAAATTATAATGATAGCACTGCACTTGTGCCAAAACAAGGATTTAGCTTAAATAGGAGT   986
APR19-43       AGAAATTATAATGATAGCACTGCACTTGTGCCAAAACAAGGATTTAGCTTAAATAGGAGT   1111
APR20-70       AGAAATTATAATGATAGCACTGCACTTGTGCCAAAACAAGGATTTAGCTTAAATAGGAGT   831
DEC10-249      ------------------------------------------------------------   0
DEC08-241      ------------------------------------------------------------   0
DEC06-81       AGAAATTATAATGATAGCACTGCACTTGTGCCAAAACAAGGATTTAGCTTAAATAGGAGT   996
DEC04-13db     AGAAATTATAATGATAGCACTGCACTTGTGCCAAAACAAGGATTTAGCTTAAATAGGAGT   8520
MAY17-11       AGAAATTATAATGATAGCACTGCACTTGTGCCAAAACAAGGATTTAGCTTAAATAGGAGT   3174
MAY15-78       AGAAATTATAATGATAGCACTGCACTTGTGCCAAAACAAGGATTTAGCTTAAATAGGAGT   752
JUL05-102      AGAAATTATAATGATAGCACTGCACTTGTGCCAAAACAAGGATTTAGCTTAAATAGGAGT   741
JUL04-107      AGAAATTATAATGATAGCACTGCACTTGTGCCAAAACAAGGATTTAGCTTAAATAGGAGT   752
JUL03-55       AGAAATTATAATGATAGCACTGCACTTGTGCCAAAACAAGGATTTAGCTTAAATAGGAGT   850
JUL02-106      AGAAATTATAATGATAGCACTGCACTTGTGCCAAAACAAGGATTTAGCTTAAATAGGAGT   741
JUL01-85       AGAAATTATAATGATAGCACTGCACTTGTGCCAAAACAAGGATTTAGCTTAAATAGGAGT   955
DEC03-186      ------------------------------------------------------------   0
DEC07-98       ------------------------------------------------------------   0
APR17-291      ------------------------------------------------------------   0


DEC02-76       CAAAGTCAAAGCTTTAGACTACCTAGACAGGAGTATGAAATACCAGTAGAGCACCCTTCT   1910
CLR03-38395    CAAAGTCAAAGCTTTAGACTACCTAGACAGGAGTATGAAATACCAGTAGAGCACCCTTCT   976
CLR01-43699    CAAAGTCAAAGCTTTAGACTACCTAGACAGGAGTATGAAATACCAGTAGAGCACCCTTCT   3647
APR16-68       CAAAGTCAAAGCTTTAGACTACCTAGACAGGAGTATGAAATACCAGTAGAGCACCCTTCT   1004
APR18-62       CAAAGTCAAAGCTTTAGACTACCTAGACAGGAGTATGAAATACCAGTAGAGCACCCTTCT   1046
```

Figure S4

| | | |
|---|---|---|
| APR19-43 | CAAAGTCAAAGCTTTAGACTACCTAGACAGGAGTATGAAATACCAGTAGAGCACCCTTCT | 1171 |
| APR20-70 | CAAAGTCAAAGCTTTAGACTACCTAGACAGGAGTATGAAATACCAGTAGAGCACCCTTCT | 891 |
| DEC10-249 | ------------------------------------------------------------ | 0 |
| DEC08-241 | ------------------------------------------------------------ | 0 |
| DEC06-81 | CAAAGTCAAAGCTTTAGACTACCTAGACAGGAGTATGAAATACCAGTAGAGCACCCTTCT | 1056 |
| DEC04-13db | CAAAGTCAAAGCTTTAGACTACCTAGACAGGAGTATGAAATACCAGTAGAGCACCCTTCT | 8580 |
| MAY17-11 | CAAAGTCAAAGCTTTAGACTACCTAGACAGGAGTATGAAATACCAGTAGAGCACCCTTCT | 3234 |
| MAY15-78 | CAAAGTCAAAGCTTTAGACTACCTAGACAGGAGTATGAAATACCAGTAGAGCACCCTTCT | 812 |
| JUL05-102 | CAAAGTCAAAGCTTTAGACTACCTAGACAGGAGTATGAAATACCAGTAGAGCACCCTTCT | 801 |
| JUL04-107 | CAAAGTCAAAGCTTTAGACTACCTAGACAGGAGTATGAAATACCAGTAGAGCACCCTTCT | 812 |
| JUL03-55 | CAAAGTCAAAGCTTTAGACTACCTAGACAGGAGTATGAAATACCAGTAGAGCACCCTTCT | 910 |
| JUL02-106 | CAAAGTCAAAGCTTTAGACTACCTAGACAGGAGTATGAAATACCAGTAGAGCACCCTTCT | 801 |
| JUL01-85 | CAAAGTCAAAGCTTTAGACTACCTAGACAGGAGTATGAAATACCAGTAGAGCACCCTTCT | 1015 |
| DEC03-186 | ------------------------------------------------------------ | 0 |
| DEC07-98 | ------------------------------------------------------------ | 0 |
| APR17-291 | ------------------------------------------------------------ | 0 |

| | | |
|---|---|---|
| DEC02-76 | AGTATTCCTATAATATTAGAAGAACCTACTAATATTCATACAGTAATAAACACACAAAAG | 1970 |
| CLR03-38395 | AGTATTCCTATAATATTAGAAGAACCTACTAATATTCATACAGTAATAAACACACAAAAG | 1036 |
| CLR01-43699 | AGTATTCCTATAATATTAGAAGAACCTACTAATATTCATACAGTAATAAACACACAAAAG | 3707 |
| APR16-68 | AGTATTCCTATAATATTAGAAGAACCTACTAATATTCATACAGTAATAAACACACAAAAG | 1064 |
| APR18-62 | AGTATTCCTATAATATTAGAAGAACCTACTAATATTCATACAGTAATAAACACACAAAAG | 1106 |
| APR19-43 | AGTATTCCTATAATATTAGAAGAACCTACTAATATTCATACAGTAATAAACACACAAAAG | 1231 |
| APR20-70 | AGTATTCCTATAATATTAGAAGAACCTACTAATATTCATACAGTAATAAACACACAAAAG | 951 |
| DEC10-249 | ------------------------------------------------------------ | 0 |
| DEC08-241 | ------------------------------------------------------------ | 0 |
| DEC06-81 | AGTATTCCTATAATATTAGAAGAACCTACTAATATTCATACAGTAATAAACACACAAAAG | 1116 |
| DEC04-13db | AGTATTCCTATAATATTAGAAGAACCTACTAATATTCATACAGTAATAAACACACAAAAG | 8640 |
| MAY17-11 | AGTATTCCTATAATATTAGAAGAACCTACTAATATTCATACAGTAATAAACACACAAAAG | 3294 |
| MAY15-78 | AGTATTCCTATAATATTAGAAGAACCTACTAATATTCATACAGTAATAAACACACAAAAG | 872 |
| JUL05-102 | AGTATTCCTATAATATTAGAAGAACCTACTAATATTCATACAGTAATAAACACACAAAAG | 861 |
| JUL04-107 | AGTATTCCTATAATATTAGAAGAACCTACTAATATTCATACAGTAATAAACACACAAAAG | 872 |
| JUL03-55 | AGTATTCCTATAATATTAGAAGAACCTACTAATATTCATACAGTAATAAACACACAAAAG | 970 |
| JUL02-106 | AGTATTCCTATAATATTAGAAGAACCTACTAATATTCATACAGTAATAAACACACAAAAG | 861 |
| JUL01-85 | AGTATTCCTATAATATTAGAAGAACCTACTAATATTCATACAGTAATAAACACACAAAAG | 1075 |
| DEC03-186 | ------------------------------------------------------------ | 0 |
| DEC07-98 | ------------------------------------------------------------ | 0 |
| APR17-291 | ------------------------------------------------------------ | 0 |

| | | |
|---|---|---|
| DEC02-76 | GATTTAAATGAAGTTAAAGAAATGATAAGAAAAATTGATAATAAATTATGAGCAGGTCAG | 2030 |
| CLR03-38395 | GATTTAAATGAAGTTAAAGAAATGATAAGAAAAATTGATAATAAATTATGAGCAGGTCAG | 1096 |
| CLR01-43699 | GATTTAAATGAAGTTAAAGAAATGATAAGAAAAATTGATAATAAATTATGAGCAGGTCAG | 3767 |
| APR16-68 | GATTTAAATGAAGTTAAAGAAATGATAAGAAAAATTGATAATAAATTATGAGCAGGTCAG | 1124 |
| APR18-62 | GATTTAAATGAAGTTAAAGAAATGATAAGAAAAATTGATAATAAATTATGAGCAGGTCAG | 1166 |
| APR19-43 | GATTTAAATGAAGTTAAAGAAATGATAAGAAAAATTGATAATAAATTATGAGCAGGTCAG | 1291 |
| APR20-70 | GATTTAAATGAAGTTAAAGAAATGATAAGAAAAATTGATAATAAATTATGAGCAGGTCAG | 1011 |
| DEC10-249 | ------------------------------------------------------------ | 0 |
| DEC08-241 | ------------------------------------------------------------ | 0 |
| DEC06-81 | GATTTAAATGAAGTTAAAGAAATGATAAGAAAAATTGATAATAAATTATGAGCAGGTCAG | 1176 |
| DEC04-13db | GATTTAAATGAAGTTAAAGAAATGATAAGAAAAATTGATAATAAATTATGAGCAGGTCAG | 8700 |
| MAY17-11 | GATTTAAATGAAGTTAAAGAAATGATAAGAAAAATTGATAATAAATTATGAGCAGGTCAG | 3354 |
| MAY15-78 | GATTTAAATGAAGTTAAAGAAATGATAAGAAAAATTGATAATAAATTATGAGCAGGTCAG | 932 |
| JUL05-102 | GATTTAAATGAAGTTAAAGAAATGATAAGAAAAATTGATAATAAATTATGAGCAGGTCAG | 921 |
| JUL04-107 | GATTTAAATGAAGTTAAAGAAATGATAAGAAAAATTGATAATAAATTATGAGCAGGTCAG | 932 |
| JUL03-55 | GATTTAAATGAAGTTAAAGAAATGATAAGAAAAATTGATAATAAATTATGAGCAGGTCAG | 1030 |
| JUL02-106 | GATTTAAATGAAGTTAAAGAAATGATAAGAAAAATTGATAATAAATTATGAGCAGGTCAG | 921 |
| JUL01-85 | GATTTAAATGAAGTTAAAGAAATGATAAGAAAAATTGATAATAAATTATGAGCAGGTCAG | 1135 |
| DEC03-186 | ------------------------------------------------------------ | 0 |
| DEC07-98 | ------------------------------------------------------------ | 0 |
| APR17-291 | ------------------------------------------------------------ | 0 |

```
DEC02-76        AATTTGAATTCAGACCAGAGGAAAGCATTTCACAATATCTCTCTAGGAATTTACCAAAAC   2090
CLR03-38395     AATTTGAATTCAGACCAGAGGAAAGCATTTCACAATATCTCTCTAGGAATTTACCAAAAC   1156
CLR01-43699     AATTTGAATTCAGACCAGAGGAAAGCATTTCACAATATCTCTCTAGGAATTTACCAAAAC   3827
APR16-68        AATTTGAATTCAGACCAGAGGAAAGCATTTCACAATATCTCTCTAGGAATTTACCAAAAC   1184
APR18-62        AATTTGAATTCAGACCAGAGGAAAGCATTTCACAATATCTCTCTAGGAATTTACCAAAAC   1226
APR19-43        AATTTGAATTCAGACCAGAGGAAAGCATTTCACAATATCTCTCTAGGAATTTACCAAAAC   1351
APR20-70        AATTTGAATTCAGACCAGAGGAAAGCATTTCACAATATCTCTCTAGGAATTTACCAAAAC   1071
DEC10-249       ------------------------------------------------------------   0
DEC08-241       ------------------------------------------------------------   0
DEC06-81        AATTTGAATTCAGACCAGAGGAAAGCATTTCACAATATCTCTCTAGGAATTTACCAAAAC   1236
DEC04-13db      AATTTGAATTCAGACCAGAGGAAAGCATTTCACAATATCTCTCTAGGAATTTACCAAAAC   8760
MAY17-11        AATTTGAATTCAGACCAGAGGAAAGCATTTCACAATATCTCTCTAGGAATTTACCAAAAC   3414
MAY15-78        AATTTGAATTCAGACCAGAGGAAAGCATTTCACAATATCTCTCTAGGAATTTACCAAAAC   992
JUL05-102       AATTTGAATTCAGACCAGAGGAAAGCATTTCACAATATCTCTCTAGGAATTTACCAAAAC   981
JUL04-107       AATTTGAATTCAGACCAGAGGAAAGCATTTCACAATATCTCTCTAGGAATTTACCAAAAC   992
JUL03-55        AATTTGAATTCAGACCAGAGGAAAGCATTTCACAATATCTCTCTAGGAATTTACCAAAAC   1090
JUL02-106       AATTTGAATTCAGACCAGAGGAAAGCATTTCACAATATCTCTCTAGGAATTTACCAAAAC   981
JUL01-85        AATTTGAATTCAGACCAGAGGAAAGCATTTCACAATATCTCTCTAGGAATTTACCAAAAC   1195
DEC03-186       ------------------------------------------------------------   0
DEC07-98        ------------------------------------------------------------   0
APR17-291       ------------------------------------------------------------   0


DEC02-76        TCCTTCAAGATAATAGTGAAATAGCAGAAAATCTAAAAGGATTAACAATAATAATAAATA   2150
CLR03-38395     TCCTTCAAGATAATAGTGAAATAGCAGAAAATCTAAAAGGATTAACAATAATAATAAATA   1216
CLR01-43699     TCCTTCAAGATAATAGTGAAATAGCAGAAAATCTAAAAGGATTAACAATAATAATAAATA   3887
APR16-68        TCCTTCAAGATAATAGTGAAATAGCAGAAAATCTAAAAGGATTAACAATAATAATAAATA   1244
APR18-62        TCCTTCAAGATAATAGTGAAATAGCAGAAAATCTAAAAGGATTAACAATAATAATAAATA   1286
APR19-43        TCCTTCAAGATAATAGTGAAATAGCAGAAAATCTAAAAGGATTAACAATAATAATAAATA   1411
APR20-70        TCCTTCAAGATAATAGTGAAATAGCAGAAAATCTAAAAGGATTAACAATAATAATAAATA   1131
DEC10-249       ------------------------------------------------------------   0
DEC08-241       ------------------------------------------------------------   0
DEC06-81        TCCTTCAAGATAATAGTGAAATAGCAGAAAATCTAAAAGGATTAACAATAATAATAAATA   1296
DEC04-13db      TCCTTCAAGATAATAGTGAAATAGCAGAAAATCTAAAAGGATTAACAATAATAATAAATA   8820
MAY17-11        TCCTTCAAGATAATAGTGAAATAGCAGAAAATCTAAAAGGATTAACAATAATAATAAATA   3474
MAY15-78        TCCTTCAAGATAATAGTGAAATAGCAGAAAATCTAAAAGGATTAACAATAATAATAAATA   1052
JUL05-102       TCCTTCAAGATAATAGTGAAATAGCAGAAAATCTAAAAGGATTAACAATAATAATAAATA   1041
JUL04-107       TCCTTCAAGATAATAGTGAAATAGCAGAAAATCTAAAAGGATTAACAATAATAATAAATA   1052
JUL03-55        TCCTTCAAGATAATAGTGAAATAGCAGAAAATCTAAAAGGATTAACAATAATAATAAATA   1150
JUL02-106       TCCTTCAAGATAATAGTGAAATAGCAGAAAATCTAAAAGGATTAACAATAATAATAAATA   1041
JUL01-85        TCCTTCAAGATAATAGTGAAATAGCAGAAAATCTAAAAGGATTAACAATAATAATAAATA   1255
DEC03-186       ------------------------------------------------------------   0
DEC07-98        ------------------------------------------TAACAATAATAATAAATA   18
APR17-291       ------------------------------------------------------------   0


DEC02-76        AACTTACAGAAAATCAAGAAAAATATGATGAAAATATGAAAAGATTTGAGGACTTATTAA   2210
CLR03-38395     AACTTACAGAAAATCAAGAAAAATATGATGAAAATATGAAAAGATTTGAGGACTTATTAA   1276
CLR01-43699     AACTTACAGAAAATCAAGAAAAATATGATGAAAATATGAAAAGATTTGAGGACTTATTAA   3947
APR16-68        AACTTACAGAAAATCAAGAAAAATATGATGAAAATATGAAAAGATTTGAGGACTTATTAA   1304
APR18-62        AACTTACAGAAAATCAAGAAAAATATGATGAAAATATGAAAAGATTTGAGGACTTATTAA   1346
APR19-43        AACTTACAGAAAATCAAGAAAAATATGATGAAAATATGAAAAGATTTGAGGACTTATTAA   1471
APR20-70        AACTTACAGAAAATCAAGAAAAATATGATGAAAATATGAAAAGATTTGAGGACTTATTAA   1191
DEC10-249       ------------------------------------------------------------   0
DEC08-241       ------------------------------------------------------------   0
DEC06-81        AACTTACAGAAAATCAAGAAAAATATGATGAAAATATGAAAAGATTTGAGGACTTATTAA   1356
DEC04-13db      AACTTACAGAAAATCAAGAAAAATATGATGAAAATATGAAAAGATTTGAGGACTTATTAA   8880
MAY17-11        AACTTACAGAAAATCAAGAAAAATATGATGAAAATATGAAAAGATTTGAGGACTTATTAA   3534
MAY15-78        AACTTACAGAAAATCAAGAAAAATATGATGAAAATATGAAAAGATTTGAGGACTTATTAA   1112
JUL05-102       AACTTACAGAAAATCAAGAAAAATATGATGAAAATATGAAAAGATTTGAGGACTTATTAA   1101
JUL04-107       AACTTACAGAAAATCAAGAAAAATATGATGAAAATATGAAAAGATTTGAGGACTTATTAA   1112
JUL03-55        AACTTACAGAAAATCAAGAAAAATATGATGAAAATATGAAAAGATTTGAGGACTTATTAA   1210
```

```
JUL02-106       AACTTACAGAAAATCAAGAAAAATATGATGAAAATATGAAAagattTGAGGACTTATTAA   1101
JUL01-85        AACTTACAGAAAATCAAGAAAAATATGATGAAAATATGAAAAGATTTGAGGACTTATTAA   1315
DEC03-186       ------------------------------------------------------------   0
DEC07-98        AACTTACAGAAAATCAAGAAAAATATGATGAAAATATGAAAAGATTTGAGGACTTATTAA   78
APR17-291       ------------------------------------------------------------   0

DEC02-76        GAGAAATAGATAGGAAGATTACTAATCTACCATCTAGTTCATCACCTAAGAAGAAGACAA   2270
CLR03-38395     GAGAAATAGATAGGAAGATTACTAATCTACCATCTAGTTCATCACCTAAGAAGAAGACAA   1336
CLR01-43699     GAGAAATAGATAGGAAGATTACTAATCTACCATCTAGTTCATCACCTAAGAAGAAGACAA   4007
APR16-68        GAGAAATAGATAGGAAGATTACTAATCTACCATCTAGTTCATCACCTAAGAAGAAGACAA   1364
APR18-62        GAGAAATAGATAGGAAGATTACTAATCTACCATCTAGTTCATCACCTAAGAAGAAGACAA   1406
APR19-43        GAGAAATAGATAGGAAGATTACTAATCTACCATCTAGTTCATCACCTAAGAAGAAGACAA   1531
APR20-70        GAGAAATAGATAGGAAGATTACTAATCTACCATCTAGTTCATCACCTAAGAAGAAGACAA   1251
DEC10-249       ------------------------------------------------------------   0
DEC08-241       ------------------------------------------------------------   0
DEC06-81        GAGAAATAGATAGGAAGATTACTAATCTACCATCTAGTTCATCACCTAAGAAGAAGACAA   1416
DEC04-13db      GAGAAATAGATAGGAAGATTACTAATCTACCATCTAGTTCATCACCTAAGAAGAAGACAA   8940
MAY17-11        GAGAAATAGATAGGAAGATTACTAATCTACCATCTAGTTCATCACCTAAGAAGAAGACAA   3594
MAY15-78        GAGAAATAGATAGGAAGATTACTAATCTACCATCTAGTTCATCACCTAAGAAGAAGACAA   1172
JUL05-102       GAGAAATAGATAGGAAGATTACTAATCTACCATCTAGTTCATCACCTAAGAAGAAGACAA   1161
JUL04-107       GAGAAATAGATAGGAAGATTACTAATCTACCATCTAGTTCATCACCTAAGAAGAAGACAA   1172
JUL03-55        GAGAAATAGATAGGAAGATTACTAATCTACCATCTAGTTCATCACCTAAGAAGAAGACAA   1270
JUL02-106       GAGAAATAGATAGGAAGATTACTAATCTACCATCTAGTTCATCACCTAAGAAGAAGACAA   1161
JUL01-85        GAGAAATAGATAGGAAGATTACTAATCTACCATCTAGTTCATCACCTAAGAAGAAGACAA   1375
DEC03-186       ------------------------------------------------------------   0
DEC07-98        GAGAAATAGATAGGAAGATTACTAATCTACCATCTAGTTCATCACCTAAGAAGAAGACAA   138
APR17-291       ------------------------------------------------------------   0

DEC02-76        TTAAGTGGACCAAACCCGATCCACCATTCCCAATACCTACTAAGATACCACCTCTACCAG   2330
CLR03-38395     TTAAGTGGACCAAACCCGATCCACCATTCCCAATACCTACTAAGATACCACCTCTACCAG   1396
CLR01-43699     TTAAGTGGACCAAACCCGATCCACCATTCCCAATACCTACTAAGATACCACCTCTACCAG   4067
APR16-68        TTAAGTGGACCAAACCCGATCCACCATTCCCAATACCTACTAAGATACCACCTCTACCAG   1424
APR18-62        TTAAGTGGACCAAACCCGATCCACCATTCCCAATACCTACTAAGATACCACCTCTACCAG   1466
APR19-43        TTAAGTGGACCAAACCCGATCCACCATTCCCAATACCTACTAAGATACCACCTCTACCAG   1591
APR20-70        TTAAGTGGACCAAACCCGATCCACCATTCCCAATACCTACTAAGATACCACCTCTACCAG   1311
DEC10-249       ------------------------------------------------------------   0
DEC08-241       ------------------------------------------------------------   0
DEC06-81        TTAAGTGGACCAAACCCGATCCACCATTCCCAATACCTACTAAGATACCACCTCTACCAG   1476
DEC04-13db      TTAAGTGGACCAAACCCGATCCACCATTCCCAATACCTACTAAGATACCACCTCTACCAG   9000
MAY17-11        TTAAGTGGACCAAACCCGATCCACCATTCCCAATACCTACTAAGATACCACCTCTACCAG   3654
MAY15-78        TTAAGTGGACCAAACCCGATCCACCATTCCCAATACCTACTAAGATACCACCTCTACCAG   1232
JUL05-102       TTAAGTGGACCAAACCCGATCCACCATTCCCAATACCTACTAAGATACCACCTCTACCAG   1221
JUL04-107       TTAAGTGGACCAAACCCGATCCACCATTCCCAATACCTACTAAGATACCACCTCTACCAG   1232
JUL03-55        TTAAGTGGACCAAACCCGATCCACCATTCCCAATACCTACTAAGATACCACCTCTACCAG   1330
JUL02-106       TTAAGTGGACCAAACCCGATCCACCATTCCCAATACCTACTAAGATACCACCTCTACCAG   1221
JUL01-85        TTAAGTGGACCAAACCCGATCCACCATTCCCAATACCTACTAAGATACCACCTCTACCAG   1435
DEC03-186       ------------------------------------------------------------   0
DEC07-98        TTAAGTGGACCAAACCCGATCCACCATTCCCAATACCTACTAAGATACCACCTCTACCAG   198
APR17-291       ------------------------------------------------------------   0

DEC02-76        ATAAAAAAACCACAATAGAATCTGTTAGAAGACAAGAAGAATTAGGAGAAAGGATTAGGAA   2390
CLR03-38395     ATAAAAAAACCACAATAGAATCTGTTAGAAGACAAGAAGAATTAGGAGAAAGGATTAGGAA   1456
CLR01-43699     ATAAAAAAACCACAATAGAATCTGTTAGAAGACAAGAAGAATTAGGAGAAAGGATTAGGAA   4127
APR16-68        ATAAAAAAACCACAATAGAATCTGTTAGAAGACAAGAAGAATTAGGAGAAAGGATTAGGAA   1484
APR18-62        ATAAAAAAACCACAATAGAATCTGTTAGAAGACAAGAAGAATTAGGAGAAAGGATTAGGAA   1526
APR19-43        ATAAAAAAACCACAATAGAATCTGTTAGAAGACAAGAAGAATTAGGAGAAAGGATTAGGAA   1651
APR20-70        ATAAAAAAACCACAATAGAATCTGTTAGAAGACAAGAAGAATTAGGAGAAAGGATTAGGAA   1371
DEC10-249       ------------------------------------------------------------   0
DEC08-241       ------------------------------------------------------------   0
DEC06-81        ATAAAAAAACCACAATAGAATCTGTTAGAAGACAAGAAGAATTAGGAGAAAGGATTAGGAA   1536
```

Figure S4

```
DEC04-13db      ATAAAAAACCACAATAGAATCTGTTAGAAGACAAGAAGAATTAGGAGAAAGGATTAGGAA   9060
MAY17-11        ATAAAAAACCACAATAGAATCTGTTAGAAGACAAGAAGAATTAGGAGAAAGGATTAGGAA   3714
MAY15-78        ATAAAAAACCACAATAGAATCTGTTAGAAGACAAGAAGAATTAGGAGAAAGGATTAGGAA   1292
JUL05-102       ATAAAAAACCACAATAGAATCTGTTAGAAGACAAGAAGAATTAGGAGAAAGGATTAGGAA   1281
JUL04-107       ATAAAAAACCACAATAGAATCTGTTAGAAGACAAGAAGAATTAGGAGAAAGGATTAGGAA   1292
JUL03-55        ATAAAAAACCACAATAGAATCTGTTAGAAGACAAGAAGAATTAGGAGAAAGGATTAGGAA   1390
JUL02-106       ATAAAAAACCACAATAGAATCTGTTAGAAGACAAGAAGAATTAGGAGAAAGGATTAGGAA   1281
JUL01-85        ATAAAAAACCACAATAGAATCTGTTAGAAGACAAGAAGAATTAGGAGAAAGGATTAGGAA   1495
DEC03-186       ------------------------------------------------------------   0
DEC07-98        ATAAAAAACCACAATAGAATCTGTTAGAAGACAAGAAGAATTAGGAGAAAGGATTAGGAA   258
APR17-291       ------------------------------------------------------------   0


DEC02-76        CTGTAAAAATCAAAGAGAATTAGATTCTTTAATAGAGGAAACCTGTAATATGGAATTTGG   2450
CLR03-38395     CTGTAAAAATCAAAGAGAATTAGATTCTTTAATAGAGGAAACCTGTAATATGGAATTTGG   1516
CLR01-43699     CTGTAAAAATCAAAGAGAATTAGATTCTTTAATAGAGGAAACCTGTAATATGGAATTTGG   4187
APR16-68        CTGTAAAAATCAAAGAGAATTAGATTCTTTAATAGAGGAAACCTGTAATATGGAATTTGG   1544
APR18-62        CTGTAAAAATCAAAGAGAATTAGATTCTTTAATAGAGGAAACCTGTAATATGGAATTTGG   1586
APR19-43        CTGTAAAAATCAAAGAGAATTAGATTCTTTAATAGAGGAAACCTGTAATATGGAATTTGG   1711
APR20-70        CTGTAAAAATCAAAGAGAATTAGATTCTTTAATAGAGGAAACCTGTAATATGGAATTTGG   1431
DEC10-249       ------------------------------------------------------------   0
DEC08-241       ------------------------------------------------------------   0
DEC06-81        CTGTAAAAATCAAAGAGAATTAGATTCTTTAATAGAGGAAACCTGTAATATGGAATTTGG   1596
DEC04-13db      CTGTAAAAATCAAAGAGAATTAGATTCTTTAATAGAGGAAACCTGTAATATGGAATTTGG   9120
MAY17-11        CTGTAAAAATCAAAGAGAATTAGATTCTTTAATAGAGGAAACCTGTAATATGGAATTTGG   3774
MAY15-78        CTGTAAAAATCAAAGAGAATTAGATTCTTTAATAGAGGAAACCTGTAATATGGAATTTGG   1352
JUL05-102       CTGTAAAAATCAAAGAGAATTAGATTCTTTAATAGAGGAAACCTGTAATATGGAATTTGG   1341
JUL04-107       CTGTAAAAATCAAAGAGAATTAGATTCTTTAATAGAGGAAACCTGTAATATGGAATTTGG   1352
JUL03-55        CTGTAAAAATCAAAGAGAATTAGATTCTTTAATAGAGGAAACCTGTAATATGGAATTTGG   1450
JUL02-106       CTGTAAAAATCAAAGAGAATTAGATTCTTTAATAGAGGAAACCTGTAATATGGAATTTGG   1341
JUL01-85        CTGTAAAAATCAAAGAGAATTAGATTCTTTAATAGAGGAAACCTGTAATATGGAATTTGG   1555
DEC03-186       ------------------------------------------------------------   0
DEC07-98        CTGTAAAAATCAAAGAGAATTAGATTCTTTAATAGAGGAAACCTGTAATATGGAATTTGG   318
APR17-291       ------------------------------------------------------------   0


DEC02-76        TTTTCAAATCCAGGATGACATCGAAGAATATATATTCGACGATGAAGATTATGAGGATGA   2510
CLR03-38395     TTTTCAAATCCAGGATGACATCGAAGAATATATATTCGACGATGAAGATTATGAGGATGA   1576
CLR01-43699     TTTTCAAATCCAGGATGACATCGAAGAATATATATTCGACGATGAAGATTATGAGGATGA   4247
APR16-68        TTTTCAAATCCAGGATGACATCGAAGAATATATATTCGACGATGAAGATTATGAGGATGA   1604
APR18-62        TTTTCAAATCCAGGATGACATCGAAGAATATATATTCGACGATGAAGATTATGAGGATGA   1646
APR19-43        TTTTCAAATCCAGGATGACATCGAAGAATATATATTCGACGATGAAGATTATGAGGATGA   1771
APR20-70        TTTTCAAATCCAGGATGACATCGAAGAATATATATTCGACGATGAAGATTATGAGGATGA   1491
DEC10-249       ------------------------------------------------------------   0
DEC08-241       ------------------------------------------------------------   0
DEC06-81        TTTTCAAATCCAGGATGACATCGAAGAATATATATTCGACGATGAAGATTATGAGGATGA   1656
DEC04-13db      TTTTCAAATCCAGGATGACATCGAAGAATATATATTCGACGATGAAGATTATGAGGATGA   9180
MAY17-11        TTTTCAAATCCAGGATGACATCGAAGAATATATATTCGACGATGAAGATTATGAGGATGA   3834
MAY15-78        TTTTCAAATCCAGGATGACATCGAAGAATATATATTCGACGATGAAGATTATGAGGATGA   1412
JUL05-102       TTTTCAAATCCAGGATGACATCGAAGAATATATATTCGACGATGAAGATTATGAGGATGA   1401
JUL04-107       TTTTCAAATCCAGGATGACATCGAAGAATATATATTCGACGATGAAGATTATGAGGATGA   1412
JUL03-55        TTTTCAAATCCAGGATGACATCGAAGAATATATATTCGACGATGAAGATTATGAGGATGA   1510
JUL02-106       TTTTCAAATCCAGGATGACATCGAAGAATATATATTCGACGATGAAGATTATGAGGATGA   1401
JUL01-85        TTTTCAAATCCAGGATGACATCGAAGAATATATATTCGACGATGAAGATTATGAGGATGA   1615
DEC03-186       ------------------------------------------------------------   0
DEC07-98        TTTTCAAATCCAGGATGACATCGAAGAATATATATTCGACGATGAAGATTATGAGGATGA   378
APR17-291       ------------------------------------------------------------   0


DEC02-76        ATATGATCATGATGAATTAATAAAAATGGAAAAAGATAAAATAAAGATTGAAGAAATATC   2570
CLR03-38395     ATATGATCATGATGAATTAATAAAAATGGAAAAAGATAAAATAAAGATTGAAGAAATATC   1636
CLR01-43699     ATATGATCATGATGAATTAATAAAAATGGAAAAAGATAAAATAAAGATTGAAGAAATATC   4307
APR16-68        ATATGATCATGATGAATTAATAAAAATGGAAAAAGATAAAATAAAGATTGAAGAAATATC   1664
```

Figure S4

```
APR18-62        ATATGATCATGATGAATTAATAAAAATGGAAAAAGATAAAATAAAGATTGAAGAAATATC   1706
APR19-43        ATATGATCATGATGAATTAATAAAAATGGAAAAAGATAAAATAAAGATTGAAGAAATATC   1831
APR20-70        ATATGATCATGATGAATTAATAAAAATGGAAAAAGATAAAATAAAGATTGAAGAAATATC   1551
DEC10-249       ------------------------------------------------------------   0
DEC08-241       --------------------------------------------------------CATC   4
DEC06-81        ATATGATCATGATGAATTAATAAAAATGGAAAAAGATAAAATAAAGATTGAAGAAATATC   1716
DEC04-13db      ATATGATCATGATGAATTAATAAAAATGGAAAAAGATAAAATAAAGATTGAAGAAATATC   9240
MAY17-11        ATATGATCATGATGAATTAATAAAAATGGAAAAAGATAAAATAAAGATTGAAGAAATATC   3894
MAY15-78        ATATGATCATGATGAATTAATAAAAATGGAAAAAGATAAAATAAAGATTGAAGAAATATC   1472
JUL05-102       ATATGATCATGATGAATTAATAAAAATGGAAAAAGATAAAATAAAGATTGAAGAAATATC   1461
JUL04-107       ATATGATCATGATGAATTAATAAAAATGGAAAAAGATAAAATAAAGATTGAAGAAATATC   1472
JUL03-55        ATATGATCATGATGAATTAATAAAAATGGAAAAAGATAAAATAAAGATTGAAGAAATATC   1570
JUL02-106       ATATGATCATGATGAATTAATAAAAATGGAAAAAGATAAAATAAAGATTGAAGAAATATC   1461
JUL01-85        ATATGATCATGATGAATTAATAAAAATGGAAAAAGATAAAATAAAGATTGAAGAAATATC   1675
DEC03-186       ------------------------------------------------------------   0
DEC07-98        ATATGATCATGATGAATTAATAAAAATGGAAAAAGATAAAATAAAGATTGAAGAAATATC   438
APR17-291       ------------------------------------------------------------   0


DEC02-76        AGATGATGAAAAATACTTAGATGCCCCTCTAAGTAAAACTGAACAAGGAGAATCTAGTAA   2630
CLR03-38395     AGATGATGAAAAATACTTAGATGCCCCTCTAAGTAAAACTGAACAAGGAGAATCTAGTAA   1696
CLR01-43699     AGATGATGAAAAATACTTAGATGCCCCTCTAAGTAAAACTGAACAAGGAGAATCTAGTAA   4367
APR16-68        AGATGATGAAAAATACTTAGATGCCCCTCTAAGTAAAACTGAACAAGGAGAATCTAGTAA   1724
APR18-62        AGATGATGAAAAATACTTAGATGCCCCTCTAAGTAAAACTGAACAAGGAGAATCTAGTAA   1766
APR19-43        AGATGATGAAAAATACTTAGATGCCCCTCTAAGTAAAACTGAACAAGGAGAATCTAGTAA   1891
APR20-70        AGATGATGAAAAATACTTAGATGCCCCTCTAAGTAAAACTGAACAAGGAGAATCTAGTAA   1611
DEC10-249       ------------------------------------------------------------   0
DEC08-241       AGATGATGAAAAATACTTAGATGCCCCTCTAAGTAAAACTGAACAAGGAGAATCTAGTAA   64
DEC06-81        AGATGATGAAAAATACTTAGATGCCCCTCTAAGTAAAACTGAACAAGGAGAATCTAGTAA   1776
DEC04-13db      AGATGATGAAAAATACTTAGATGCCCCTCTAAGTAAAACTGAACAAGGAGAATCTAGTAA   9300
MAY17-11        AGATGATGAAAAATACTTAGATGCCCCTCTAAGTAAAACTGAACAAGGAGAATCTAGTAA   3954
MAY15-78        AGATGATGAAAAATACTTAGATGCCCCTCTAAGTAAAACTGAACAAGGAGAATCTAGTAA   1532
JUL05-102       AGATGATGAAAAATACTTAGATGCCCCTCTAAGTAAAACTGAACAAGGAGAATCTAGTAA   1521
JUL04-107       AGATGATGAAAAATACTTAGATGCCCCTCTAAGTAAAACTGAACAAGGAGAATCTAGTAA   1532
JUL03-55        AGATGATGAAAAATACTTAGATGCCCCTCTAAGTAAAACTGAACAAGGAGAATCTAGTAA   1630
JUL02-106       AGATGATGAAAAATACTTAGATGCCCCTCTAAGTAAAACTGAACAAGGAGAATCTAGTAA   1521
JUL01-85        AGATGATGAAAAATACTTAGATGCCCCTCTAAGTAAAACTGAACAAGGAGAATCTAGTAA   1735
DEC03-186       ------------------------------------------------------------   0
DEC07-98        AGATGATGAAAAATACTTAGATGCCCCTCTAAGTAAAACTGAACAAGGAGAATCTAGTAA   498
APR17-291       ------------------------------------------------------------   0


DEC02-76        CAGTAAAGTTAAATATGAAGATGAAGAAGGAATTAACTTAAAAGATGAGGAATTTAGTGA   2690
CLR03-38395     CAGTAAAGTTAAATATGAAGATGAAGAAGGAATTAACTTAAAAGATGAGGAATTTAGTGA   1756
CLR01-43699     CAGTAAAGTTAAATATGAAGATGAAGAAGGAATTAACTTAAAAGATGAGGAATTTAGTGA   4427
APR16-68        CAGTAAAGTTAAATATGAAGATGAAGAAGGAATTAACTTAAAAGATGAGGAATTTAGTGA   1784
APR18-62        CAGTAAAGTTAAATATGAAGATGAAGAAGGAATTAACTTAAAAGATGAGGAATTTAGTGA   1826
APR19-43        CAGTAAAGTTAAATATGAAGATGAAGAAGGAATTAACTTAAAAGATGAGGAATTTAGTGA   1951
APR20-70        CAGTAAAGTTAAATATGAAGATGAAGAAGGAATTAACTTAAAAGATGAGGAATTTAGTGA   1671
DEC10-249       ------------------------------------------------------------   0
DEC08-241       CAGTAAAGTTAAATATGAAGATGAAGAAGGAATTAACTTAAAAGATGAGGAATTTAGTGA   124
DEC06-81        CAGTAAAGTTAAATATGAAGATGAAGAAGGAATTAACTTAAAAGATGAGGAATTTAGTGA   1836
DEC04-13db      CAGTAAAGTTAAATATGAAGATGAAGAAGGAATTAACTTAAAAGATGAGGAATTTAGTGA   9360
MAY17-11        CAGTAAAGTTAAATATGAAGATGAAGAAGGAATTAACTTAAAAGATGAGGAATTTAGTGA   4014
MAY15-78        CAGTAAAGTTAAATATGAAGATGAAGAAGGAATTAACTTAAAAGATGAGGAATTTAGTGA   1592
JUL05-102       CAGTAAAGTTAAATATGAAGATGAAGAAGGAATTAACTTAAAAGATGAGGAATTTAGTGA   1581
JUL04-107       CAGTAAAGTTAAATATGAAGATGAAGAAGGAATTAACTTAAAAGATGAGGAATTTAGTGA   1592
JUL03-55        CAGTAAAGTTAAATATGAAGATGAAGAAGGAATTAACTTAAAAGATGAGGAATTTAGTGA   1690
JUL02-106       CAGTAAAGTTAAATATGAAGATGAAGAAGGAATTAACTTAAAAGATGAGGAATTTAGTGA   1581
JUL01-85        CAGTAAAGTTAAATATGAAGATGAAGAAGGAATTAACTTAAAAGATGAGGAATTTAGTGA   1795
DEC03-186       ---------------------TGAAGAAGGAATTAACTTAAAAGATGAGGAATTTAGTGA   39
DEC07-98        CAGTAAAGTTAAATATGAAGATGAAGAAGGAATTAACTTAAAAGATGAGGAATTTAGTGA   558
APR17-291       ------------------------------------------------------------   0
```

Figure S4

```
DEC02-76        TGATGAAGTAAATGAATATTTCGACCCTTCATTAGATCCACTAAAAGATGGAATAGAAAA   2750
CLR03-38395     TGATGAAGTAAATGAATATTTCGACCCTTCATTAGATCCACTAAAAGATGGAATAGAAAA   1816
CLR01-43699     TGATGAAGTAAATGAATATTTCGACCCTTCATTAGATCCACTAAAAGATGGAATAGAAAA   4487
APR16-68        TGATGAAGTAAATGAATATTTCGACCCTTCATTAGATCCACTAAAAGATGGAATAGAAAA   1844
APR18-62        TGATGAAGTAAATGAATATTTCGACCCTTCATTAGATCCACTAAAAGATGGAATAGAAAA   1886
APR19-43        TGATGAAGTAAATGAATATTTCGACCCTTCATTAGATCCACTAAAAGATGGAATAGAAAA   2011
APR20-70        TGATGAAGTAAATGAATATTTCGACCCTTCATTAGATCCACTAAAAGATGGAATAGAAAA   1731
DEC10-249       ------------------------------------------------------------   0
DEC08-241       TGATGAAGTAAATGAATATTTCGACCCTTCATTAGATCCACTAAAAGATGGAATAGAAAA   184
DEC06-81        TGATGAAGTAAATGAATATTTCGACCCTTCATTAGATCCACTAAAAGATGGAATAGAAAA   1896
DEC04-13db      TGATGAAGTAAATGAATATTTCGACCCTTCATTAGATCCACTAAAAGATGGAATAGAAAA   9420
MAY17-11        TGATGAAGTAAATGAATATTTCGACCCTTCATTAGATCCACTAAAAGATGGAATAGAAAA   4074
MAY15-78        TGATGAAGTAAATGAATATTTCGACCCTTCATTAGATCCACTAAAAGATGGAATAGAAAA   1652
JUL05-102       TGATGAAGTAAATGAATATTTCGACCCTTCATTAGATCCACTAAAAGATGGAATAGAAAA   1641
JUL04-107       TGATGAAGTAAATGAATATTTCGACCCTTCATTAGATCCACTAAAAGATGGAATAGAAAA   1652
JUL03-55        TGATGAAGTAAATGAATATTTCGACCCTTCATTAGATCCACTAAAAGATGGAATAGAAAA   1750
JUL02-106       TGATGAAGTAAATGAATATTTCGACCCTTCATTAGATCCACTAAAAGATGGAATAGAAAA   1641
JUL01-85        TGATGAAGTAAATGAATATTTCGACCCTTCATTAGATCCACTAAAAGATGGAATAGAAAA   1855
DEC03-186       TGATGAAGTAAATGAATATTTCGACCCTTCATTAGATCCACTAAAAGATGGAATAGAAAA   99
DEC07-98        TGATGAAGTAAATGAATATTTCGACCCTTCATTAGATCCACTAAAAGATGGAATAGAAAA   618
APR17-291       ------------------------------------------------------------   0


DEC02-76        CATTGATAATGATGGATTAAGCAGATTAGAAACTAGGAAATCTAGTAAAAGAAGAGGAAC   2810
CLR03-38395     CATTGATAATGATGGATTAAGCAGATTAGAAACTAGGAAATCTAGTAAAAGAAGAGGAAC   1876
CLR01-43699     CATTGATAATGATGGATTAAGCAGATTAGAAACTAGGAAATCTAGTAAAAGAAGAGGAAC   4547
APR16-68        CATTGATAATGATGGATTAAGCAGATTAGAAACTAGGAAATCTAGTAAAAGAAGAGGAAC   1904
APR18-62        CATTGATAATGATGGATTAAGCAGATTAGAAACTAGGAAATCTAGTAAAAGAAGAGGAAC   1946
APR19-43        CATTGATAATGATGGATTAAGCAGATTAGAAACTAGGAAATCTAGTAAAAGAAGAGGAAC   2071
APR20-70        CATTGATAATGATGGATTAAGCAGATTAGAAACTAGGAAATCTAGTAAAAGAAGAGGAAC   1791
DEC10-249       ------------------------------------------------------------   0
DEC08-241       CATTGATAATGATGGATTAAGCAGATTAGAAACTAGGAAATCTAGTAAAAGAAGAGGAAC   244
DEC06-81        CATTGATAATGATGGATTAAGCAGATTAGAAACTAGGAAATCTAGTAAAAGAAGAGGAAC   1956
DEC04-13db      CATTGATAATGATGGATTAAGCAGATTAGAAACTAGGAAATCTAGTAAAAGAAGAGGAAC   9480
MAY17-11        CATTGATAATGATGGATTAAGCAGATTAGAAACTAGGAAATCTAGTAAAAGAAGAGGAAC   4134
MAY15-78        CATTGATAATGATGGATTAAGCAGATTAGAAACTAGGAAATCTAGTAAAAGAAGAGGAAC   1712
JUL05-102       CATTGATAATGATGGATTAAGCAGATTAGAAACTAGGAAATCTAGTAAAAGAAGAGGAAC   1701
JUL04-107       CATTGATAATGATGGATTAAGCAGATTAGAAACTAGGAAATCTAGTAAAAGAAGAGGAAC   1712
JUL03-55        CATTGATAATGATGGATTAAGCAGATTAGAAACTAGGAAATCTAGTAAAAGAAGAGGAAC   1810
JUL02-106       CATTGATAATGATGGATTAAGCAGATTAGAAACTAGGAAATCTAGTAAAAGAAGAGGAAC   1701
JUL01-85        CATTGATAATGATGGATTAAGCAGATTAGAAACTAGGAAATCTAGTAAAAGAAGAGGAAC   1915
DEC03-186       CATTGATAATGATGGATTAAGCAGATTAGAAACTAGGAAATCTAGTAAAAGAAGAGGAAC   159
DEC07-98        CATTGATAATGATGGATTAAGCAGATTAGAAACTAGGAAATCTAGTAAAAGAAGAGGAAC   678
APR17-291       ------------------------------------------------------------   0


DEC02-76        TTCAGGATACTATCATTCTTATGATAGTAAATACAAGACTAAAATACCCCCACAATACCA   2870
CLR03-38395     TTCAGGATACTATCATTCTTATGATAGTAAATACAAGACTAAAATACCCCCACAATACCA   1936
CLR01-43699     TTCAGGATACTATCATTCTTATGATAGTAAATACAAGACTAAAATACCCCCACAATACCA   4607
APR16-68        TTCAGGATACTATCATTCTTATGATAGTAAATACAAGACTAAAATACCCCCACAATACCA   1964
APR18-62        TTCAGGATACTATCATTCTTATGATAGTAAATACAAGACTAAAATACCCCCACAATACCA   2006
APR19-43        TTCAGGATACTATCATTCTTATGATAGTAAATACAAGACTAAAATACCCCCACAATACCA   2131
APR20-70        TTCAGGATACTATCATTCTTATGATAGTAAATACAAGACTAAAATACCCCCACAATACCA   1851
DEC10-249       ---------------------------------------------GCCCCCACAATACCA   15
DEC08-241       TTCAGGATACTATCATTCTTATGATAGTAAATACAAGACTAAAATACCCCCACAATACCA   304
DEC06-81        TTCAGGATACTATCATTCTTATGATAGTAAATACAAGACTAAAATACCCCCACAATACCA   2016
DEC04-13db      TTCAGGATACTATCATTCTTATGATAGTAAATACAAGACTAAAATACCCCCACAATACCA   9540
MAY17-11        TTCAGGATACTATCATTCTTATGATAGTAAATACAAGACTAAAATACCCCCACAATACCA   4194
MAY15-78        TTCAGGATACTATCATTCTTATGATAGTAAATACAAGACTAAAATACCCCCACAATACCA   1772
JUL05-102       TTCAGGATACTATCATTCTTATGATAGTAAATACAAGACTAAAATACCCCCACAATACCA   1761
JUL04-107       TTCAGGATACTATCATTCTTATGATAGTAAATACAAGACTAAAATACCCCCACAATACCA   1772
```

Figure S4

| | | |
|---|---|---|
| JUL03-55 | TTCAGGATACTATCATTCTTATGATAGTAAATACAAGACTAAAATACCCCCACAATACCA | 1870 |
| JUL02-106 | TTCAGGATACTATCATTCTTATGATAGTAAATACAAGACTAAAATACCCCCACAATACCA | 1761 |
| JUL01-85 | TTCAGGATACTATCATTCTTATGATAGTAAATACAAGACTAAAATACCCCCACAATACCA | 1975 |
| DEC03-186 | TTCAGGATACTATCATTCTTATGATAGTAAATACAAGACTAAAATACCCCCACAATACCA | 219 |
| DEC07-98 | TTCAGGATACTATCATTCTTATGATAGTAAATACAAGACTAAAATACCCCCACAATACCA | 738 |
| APR17-291 | ------------------------------------------------------------ | 0 |

| | | |
|---|---|---|
| DEC02-76 | GCTAAGCCATAGCGAAAATGCGTATAATAATAGGTGGTTAAACCTAGATTGTACGTTAGA | 2930 |
| CLR03-38395 | GCTAAGCCATAGCGAAAATGCGTATAATAATAGGTGGTTAAACCTAGATTGTACGTTAGA | 1996 |
| CLR01-43699 | GCTAAGCCATAGCGAAAATGCGTATAATAATAGGTGGTTAAACCTAGATTGTACGTTAGA | 4667 |
| APR16-68 | GCTAAGCCATAGCGAAAATGCGTATAATAATAGGTGGTTAAACCTAGATTGTACGTTAGA | 2024 |
| APR18-62 | GCTAAGCCATAGCGAAAATGCGTATAATAATAGGTGGTTAAACCTAGATTGTACGTTAGA | 2066 |
| APR19-43 | GCTAAGCCATAGCGAAAATGCGTATAATAATAGGTGGTTAAACCTAGATTGTACGTTAGA | 2191 |
| APR20-70 | GCTAAGCCATAGCGAAAATGCGTATAATAATAGGTGGTTAAACCTAGATTGTACGTTAGA | 1911 |
| DEC10-249 | GCTAAGCCATAGCGAAAATGCGTATAATAATAGGTGGTTAAACCTAGATTGTACGTTAGA | 75 |
| DEC08-241 | GCTAAGCCATAGCGAAAATGCGTATAATAATAGGTGGTTAAACCTAGATTGTACGTTAGA | 364 |
| DEC06-81 | GCTAAGCCATAGCGAAAATGCGTATAATAATAGGTGGTTAAACCTAGATTGTACGTTAGA | 2076 |
| DEC04-13db | GCTAAGCCATAGCGAAAATGCGTATAATAATAGGTGGTTAAACCTAGATTGTACGTTAGA | 9600 |
| MAY17-11 | GCTAAGCCATAGCGAAAATGCGTATAATAATAGGTGGTTAAACCTAGATTGTACGTTAGA | 4254 |
| MAY15-78 | GCTAAGCCATAGCGAAAATGCGTATAATAATAGGTGGTTAAACCTAGATTGTACGTTAGA | 1832 |
| JUL05-102 | GCTAAGCCATAGCGAAAATGCGTATAATAATAGGTGGTTAAACCTAGATTGTACGTTAGA | 1821 |
| JUL04-107 | GCTAAGCCATAGCGAAAATGCGTATAATAATAGGTGGTTAAACCTAGATTGTACGTTAGA | 1832 |
| JUL03-55 | GCTAAGCCATAGCGAAAATGCGTATAATAATAGGTGGTTAAACCTAGATTGTACGTTAGA | 1930 |
| JUL02-106 | GCTAAGCCATAGCGAAAATGCGTATAATAATAGGTGGTTAAACCTAGATTGTACGTTAGA | 1821 |
| JUL01-85 | GCTAAGCCATAGCGAAAATGCGTATAATAATAGGTGGTTAAACCTAGATTGTACGTTAGA | 2035 |
| DEC03-186 | GCTAAGCCATAGCGAAAATGCGTATAATAATAGGTGGTTAAACCTAGATTGTACGTTAGA | 279 |
| DEC07-98 | GCTAAGCCATAGCGAAAATGCGTATAATAATAGGTGGTTAAACCTAGATTGTACGTTAGA | 798 |
| APR17-291 | ------------------------------------------------------------ | 0 |

| | | |
|---|---|---|
| DEC02-76 | TAAAACCAATGAATTAGACGGTTGGTACCGTCAAATGAGCTTTTTAAGCTTAAGGAACAT | 2990 |
| CLR03-38395 | TAAAACCAATGAATTAGACGGTTGGTACCGTCAAATGAGCTTTTTAAGCTTAAGGAACAT | 2056 |
| CLR01-43699 | TAAAACCAATGAATTAGACGGTTGGTACCGTCAAATGAGCTTTTTAAGCTTAAGGAACAT | 4727 |
| APR16-68 | TAAAACCAATGAATTAGACGGTTGGTACCGTCAAATGAGCTTTTTAAGCTTAAGGAACAT | 2084 |
| APR18-62 | TAAAACCAATGAATTAGACGGTTGGTACCGTCAAATGAGCTTTTTAAGCTTAAGGAACAT | 2126 |
| APR19-43 | TAAAACCAATGAATTAGACGGTTGGTACCGTCAAATGAGCTTTTTAAGCTTAAGGAACAT | 2251 |
| APR20-70 | TAAAACCAATGAATTAGACGGTTGGTACCGTCAAATGAGCTTTTTAAGCTTAAGGAACAT | 1971 |
| DEC10-249 | TAAAACCAATGAATTAGACGGTTGGTACCGTCAAATGAGCTTTTTAAGCTTAAGGAACAT | 135 |
| DEC08-241 | TAAAACCAATGAATTAGACGGTTGGTACCGTCAAATGAGCTTTTTAAGCTTAAGGAACAT | 424 |
| DEC06-81 | TAAAACCAATGAATTAGACGGTTGGTACCGTCAAATGAGCTTTTTAAGCTTAAGGAACAT | 2136 |
| DEC04-13db | TAAAACCAATGAATTAGACGGTTGGTACCGTCAAATGAGCTTTTTAAGCTTAAGGAACAT | 9660 |
| MAY17-11 | TAAAACCAATGAATTAGACGGTTGGTACCGTCAAATGAGCTTTTTAAGCTTAAGGAACAT | 4314 |
| MAY15-78 | TAAAACCAATGAATTAGACGGTTGGTACCGTCAAATGAGCTTTTTAAGCTTAAGGAACAT | 1892 |
| JUL05-102 | TAAAACCAATGAATTAGACGGTTGGTACCGTCAAATGAGCTTTTTAAGCTTAAGGAACAT | 1881 |
| JUL04-107 | TAAAACCAATGAATTAGACGGTTGGTACCGTCAAATGAGCTTTTTAAGCTTAAGGAACAT | 1892 |
| JUL03-55 | TAAAACCAATGAATTAGACGGTTGGTACCGTCAAATGAGCTTTTTAAGCTTAAGGAACAT | 1990 |
| JUL02-106 | TAAAACCAATGAATTAGACGGTTGGTACCGTCAAATGAGCTTTTTAAGCTTAAGGAACAT | 1881 |
| JUL01-85 | TAAAACCAATGAATTAGACGGTTGGTACCGTCAAATGAGCTTTTTAAGCTTAAGGAACAT | 2095 |
| DEC03-186 | TAAAACCAATGAATTAGACGGTTGGTACCGTCAAATGAGCTTTTTAAGCTTAAGGAACAT | 339 |
| DEC07-98 | TAAAACCAATGAATTAGACGGTTGGTACCGTCAAATGAGCTTTTTAAGCTTAAGGAACAT | 858 |
| APR17-291 | ------------------------------------------------------------ | 0 |

| | | |
|---|---|---|
| DEC02-76 | AGAAACAGTAGCAGACCTAGAACCGTTCTTGGAACATTTCATGACTGGAAATGTACGAGC | 3050 |
| CLR03-38395 | AGAAACAGTAGCAGACCTAGAACCGTTCTTGGAACATTTCATGACTGGAAATGTACGAGC | 2116 |
| CLR01-43699 | AGAAACAGTAGCAGACCTAGAACCGTTCTTGGAACATTTCATGACTGGAAATGTACGAGC | 4787 |
| APR16-68 | AGAAACAGTAGCAGACCTAGAACCGTTCTTGGAACATTTCATGACTGGAAATGTACGAGC | 2144 |
| APR18-62 | AGAAACAGTAGCAGACCTAGAACCGTTCTTGGAACATTTCATGACTGGAAATGTACGAGC | 2186 |
| APR19-43 | AGAAACAGTAGCAGACCTAGAACCGTTCTTGGAACATTTCATGACTGGAAATGTACGAGC | 2311 |
| APR20-70 | AGAAACAGTAGCAGACCTAGAACCGTTCTTGGAACATTTCATGACTGGAAATGTACGAGC | 2031 |
| DEC10-249 | AGAAACAGTAGCAGACCTAGAACCGTTCTTGGAACATTTCATGACTGGAAATGTACGAGC | 195 |
| DEC08-241 | AGAAACAGTAGCAGACCTAGAACCGTTCTTGGAACATTTCATGACTGGAAATGTACGAGC | 484 |

Figure S4

```
DEC06-81        AGAAACAGTAGCAGACCTAGAACCGTTCTTGGAACATTTCATGACTGGAAATGTACGAGC   2196
DEC04-13db      AGAAACAGTAGCAGACCTAGAACCGTTCTTGGAACATTTCATGACTGGAAATGTACGAGC   9720
MAY17-11        AGAAACAGTAGCAGACCTAGAACCGTTCTTGGAACATTTCATGACTGGAAATGTACGAGC   4374
MAY15-78        AGAAACAGTAGCAGACCTAGAACCGTTCTTGGAACATTTCATGACTGGAAATGTACGAGC   1952
JUL05-102       AGAAACAGTAGCAGACCTAGAACCGTTCTTGGAACATTTCATGACTGGAAATGTACGAGC   1941
JUL04-107       AGAAACAGTAGCAGACCTAGAACCGTTCTTGGAACATTTCATGACTGGAAATGTACGAGC   1952
JUL03-55        AGAAACAGTAGCAGACCTAGAACCGTTCTTGGAACATTTCATGACTGGAAATGTACGAGC   2050
JUL02-106       AGAAACAGTAGCAGACCTAGAACCGTTCTTGGAACATTTCATGACTGGAAATGTACGAGC   1941
JUL01-85        AGAAACAGTAGCAGACCTAGAACCGTTCTTGGAACATTTCATGACTGGAAATGTACGAGC   2155
DEC03-186       AGAAACAGTAGCAGACCTAGAACCGTTCTTGGAACATTTCATGACTGGAAATGTACGAGC   399
DEC07-98        AGAAACAGTAGCAGACCTAGAACCGTTCTTGGAACATTTCATGACTGGAAATGTACGAGC   918
APR17-291       ------------------------------------------------------------   0


DEC02-76        ATGGTGGAATTCTGAAAGAGGAGAAGCATTAAAATCTACTTATCTAACTTCAGATGACAC   3110
CLR03-38395     ATGGTGGAATTCTGAAAGAGGAGAAGCATTAAAATCTACTTATCTAACTTCAGATGACAC   2176
CLR01-43699     ATGGTGGAATTCTGAAAGAGGAGAAGCATTAAAATCTACTTATCTAACTTCAGATGACAC   4847
APR16-68        ATGGTGGAATTCTGAAAGAGGAGAAGCATTAAAATCTACTTATCTAACTTCAGATGACAC   2204
APR18-62        ATGGTGGAATTCTGAAAGAGGAGAAGCATTAAAATCTACTTATCTAACTTCAGATGACAC   2246
APR19-43        ATGGTGGAATTCTGAAAGAGGAGAAGCATTAAAATCTACTTATCTAACTTCAGATGACAC   2371
APR20-70        ATGGTGGAATTCTGAAAGAGGAGAAGCATTAAAATCTACTTATCTAACTTCAGATGACAC   2091
DEC10-249       ATGGTGGAATTCTGAAAGAGGAGAAGCATTAAAATCTACTTATCTAACTTCAGATGACAC   255
DEC08-241       ATGGTGGAATTCTGAAAGAGGAGAAGCATTAAAATCTACTTATCTAACTTCAGATGACAC   544
DEC06-81        ATGGTGGAATTCTGAAAGAGGAGAAGCATTAAAATCTACTTATCTAACTTCAGATGACAC   2256
DEC04-13db      ATGGTGGAATTCTGAAAGAGGAGAAGCATTAAAATCTACTTATCTAACTTCAGATGACAC   9780
MAY17-11        ATGGTGGAATTCTGAAAGAGGAGAAGCATTAAAATCTACTTATCTAACTTCAGATGACAC   4434
MAY15-78        ATGGTGGAATTCTGAAAGAGGAGAAGCATTAAAATCTACTTATCTAACTTCAGATGACAC   2012
JUL05-102       ATGGTGGAATTCTGAAAGAGGAGAAGCATTAAAATCTACTTATCTAACTTCAGATGACAC   2001
JUL04-107       ATGGTGGAATTCTGAAAGAGGAGAAGCATTAAAATCTACTTATCTAACTTCAGATGACAC   2012
JUL03-55        ATGGTGGAATTCTGAAAGAGGAGAAGCATTAAAATCTACTTATCTAACTTCAGATGACAC   2110
JUL02-106       ATGGTGGAATTCTGAAAGAGGAGAAGCATTAAAATCTACTTATCTAACTTCAGATGACAC   2001
JUL01-85        ATGGTGGAATTCTGAAAGAGGAGAAGCATTAAAATCTACTTATCTAACTTCAGATGACAC   2215
DEC03-186       ATGGTGGAATTCTGAAAGAGGAGAAGCATTAAAATCTACTTATCTAACTTCAGATGACAC   459
DEC07-98        ATGGTGGAATTCTGAAAGAGGAGAAGCATTAAAATCTACTTATCTAACTTCAGATGACAC   978
APR17-291       ------------------------------------------------------------   0


DEC02-76        AATACAAACTAGATTAACGAAGATTAAAGCCTTAATTTCTAACGAATTTATAGGTTTAAA   3170
CLR03-38395     AATACAAACTAGATTAACGAAGATTAAAGCCTTAATTTCTAACGAATTTATAGGTTTAAA   2236
CLR01-43699     AATACAAACTAGATTAACGAAGATTAAAGCCTTAATTTCTAACGAATTTATAGGTTTAAA   4907
APR16-68        AATACAAACTAGATTAACGAAGATTAAAGCCTTAATTTCTAACGAATTTATAGGTTTAAA   2264
APR18-62        AATACAAACTAGATTAACGAAGATTAAAGCCTTAATTTCTAACGAATTTATAGGTTTAAA   2306
APR19-43        AATACAAACTAGATTAACGAAGATTAAAGCCTTAATTTCTAACGAATTTATAGGTTTAAA   2431
APR20-70        AATACAAACTAGATTAACGAAGATTAAAGCCTTAATTTCTAACGAATTTATAGGTTTAAA   2151
DEC10-249       AATACAAACTAGATTAACGAAGATTAAAGCCTTAATTTCTAACGAATTTATAGGTTTAAA   315
DEC08-241       AATACAAACTAGATTAACGAAGATTAAAGCCTTAATTTCTAACGAATTTATAGGTTTAAA   604
DEC06-81        AATACAAACTAGATTAACGAAGATTAAAGCCTTAATTTCTAACGAATTTATAGGTTTAAA   2316
DEC04-13db      AATACAAACTAGATTAACGAAGATTAAAGCCTTAATTTCTAACGAATTTATAGGTTTAAA   9840
MAY17-11        AATACAAACTAGATTAACGAAGATTAAAGCCTTAATTTCTAACGAATTTATAGGTTTAAA   4494
MAY15-78        AATACAAACTAGATTAACGAAGATTAAAGCCTTAATTTCTAACGAATTTATAGGTTTAAA   2072
JUL05-102       AATACAAACTAGATTAACGAAGATTAAAGCCTTAATTTCTAACGAATTTATAGGTTTAAA   2061
JUL04-107       AATACAAACTAGATTAACGAAGATTAAAGCCTTAATTTCTAACGAATTTATAGGTTTAAA   2072
JUL03-55        AATACAAACTAGATTAACGAAGATTAAAGCCTTAATTTCTAACGAATTTATAGGTTTAAA   2170
JUL02-106       AATACAAACTAGATTAACGAAGATTAAAGCCTTAATTTCTAACGAATTTATAGGTTTAAA   2061
JUL01-85        AATACAAACTAGATTAACGAAGATTAAAGCCTTAATTTCTAACGAATTTATAGGTTTAAA   2275
DEC03-186       AATACAAACTAGATTAACGAAGATTAAAGCCTTAATTTCTAACGAATTTATAGGTTTAAA   519
DEC07-98        AATACAAACTAGATTAACGAAGATTAAAGCCTTAATTTCTAACGAATTTATAGGTTTAAA   1038
APR17-291       ------------------------------------------------------------   0


DEC02-76        TCAAATTAATCTAAAAGCAGTTTAGGAAAAAGAATCAGAGAAAGCAGAATATATTCTCAA   3230
CLR03-38395     TCAAATTAATCTAAAAGCAGTTTAGGAAAAAGAATCAGAGAAAGCAGAATATATTCTCAA   2296
CLR01-43699     TCAAATTAATCTAAAAGCAGTTTAGGAAAAAGAATCAGAGAAAGCAGAATATATTCTCAA   4967
```

Figure S4

```
APR16-68        TCAAATTAATCTAAAAGCAGTTTAGGAAAAAGAATCAGAGAAAGCAGAATATATTCTCAA   2324
APR18-62        TCAAATTAATCTAAAAGCAGTTTAGGAAAAAGAATCAGAGAAAGCAGAATATATTCTCAA   2366
APR19-43        TCAAATTAATCTAAAAGCAGTTTAGGAAAAAGAATCAGAGAAAGCAGAATATATTCTCAA   2491
APR20-70        TCAAATTAATCTAAAAGCAGTTTAGGAAAAAGAATCAGAGAAAGCAGAATATATTCTCAA   2211
DEC10-249       TCAAATTAATCTAAAAGCAGTTTAGGAAAAAGAATCAGAGAAAGCAGAATATATTCTCAA   375
DEC08-241       TCAAATTAATCTAAAAGCAGTTTAGGAAAAAGAATCAGAGAAAGCAGAATATATTCTCAA   664
DEC06-81        TCAAATTAATCTAAAAGCAGTTTAGGAAAAAGAATCAGAGAAAGCAGAATATATTCTCAA   2376
DEC04-13db      TCAAATTAATCTAAAAGCAGTTTAGGAAAAAGAATCAGAGAAAGCAGAATATATTCTCAA   9900
MAY17-11        TCAAATTAATCTAAAAGCAGTTTAGGAAAAAGAATCAGAGAAAGCAGAATATATTCTCAA   4554
MAY15-78        TCAAATTAATCTAAAAGCAGTTTAGGAAAAAGAATCAGAGAAAGCAGAATATATTCTCAA   2132
JUL05-102       TCAAATTAATCTAAAAGCAGTTTAGGAAAAAGAATCAGAGAAAGCAGAATATATTCTCAA   2121
JUL04-107       TCAAATTAATCTAAAAGCAGTTTAGGAAAAAGAATCAGAGAAAGCAGAATATATTCTCAA   2132
JUL03-55        TCAAATTAATCTAAAAGCAGTTTAGGAAAAAGAATCAGAGAAAGCAGAATATATTCTCAA   2230
JUL02-106       TCAAATTAATCTAAAAGCAGTTTAGGAAAAAGAATCAGAGAAAGCAGAATATATTCTCAA   2121
JUL01-85        TCAAATTAATCTAAAAGCAGTTTAGGAAAAAGAATCAGAGAAAGCAGAATATATTCTCAA   2335
DEC03-186       TCAAATTAATCTAAAAGCAGTTTAGGAAAAAGAATCAGAGAAAGCAGAATATATTCTCAA   579
DEC07-98        TCAAATTAATCTAAAAGCAGTTTAGGAAAAAGAATCAGAGAAAGCAGAATATATTCTCAA   1098
APR17-291       ------------------------------------------------------------   0


DEC02-76        CAATATCAAGATCTGTGATCTATGTTATTGGAAGAATTTTGTCTGTGAGTATGAAAAATG   3290
CLR03-38395     CAATATCAAGATCTGTGATCTATGTTATTGGAAGAATTTTGTCTGTGAGTATGAAAAATG   2356
CLR01-43699     CAATATCAAGATCTGTGATCTATGTTATTGGAAGAATTTTGTCTGTGAGTATGAAAAATG   5027
APR16-68        CAATATCAAGATCTGTGATCTATGTTATTGGAAGAATTTTGTCTGTGAGTATGAAAAATG   2384
APR18-62        CAATATCAAGATCTGTGATCTATGTTATTGGAAGAATTTTGTCTGTGAGTATGAAAAATG   2426
APR19-43        CAATATCAAGATCTGTGATCTATGTTATTGGAAGAATTTTGTCTGTGAGTATGAAAAATG   2551
APR20-70        CAATATCAAGATCTGTGATCTATGTTATTGGAAGAATTTTGTCTGTGAGTATGAAAAATG   2271
DEC10-249       CAATATCAAGATCTGTGATCTATGTTATTGGAAGAATTTTGTCTGTGAGTATGAAAAATG   435
DEC08-241       CAATATCAAGATCTGTGATCTATGTTATTGGAAGAATTTTGTCTGTGAGTATGAAAAATG   724
DEC06-81        CAATATCAAGATCTGTGATCTATGTTATTGGAAGAATTTTGTCTGTGAGTATGAAAAATG   2436
DEC04-13db      CAATATCAAGATCTGTGATCTATGTTATTGGAAGAATTTTGTCTGTGAGTATGAAAAATG   9960
MAY17-11        CAATATCAAGATCTGTGATCTATGTTATTGGAAGAATTTTGTCTGTGAGTATGAAAAATG   4614
MAY15-78        CAATATCAAGATCTGTGATCTATGTTATTGGAAGAATTTTGTCTGTGAGTATGAAAAATG   2192
JUL05-102       CAATATCAAGATCTGTGATCTATGTTATTGGAAGAATTTTGTCTGTGAGTATGAAAAATG   2181
JUL04-107       CAATATCAAGATCTGTGATCTATGTTATTGGAAGAATTTTGTCTGTGAGTATGAAAAATG   2192
JUL03-55        CAATATCAAGATCTGTGATCTATGTTATTGGAAGAATTTTGTCTGTGAGTATGAAAAATG   2290
JUL02-106       CAATATCAAGATCTGTGATCTATGTTATTGGAAGAATTTTGTCTGTGAGTATGAAAAATG   2181
JUL01-85        CAATATCAAGATCTGTGATCTATGTTATTGGAAGAATTTTGTCTGTGAGTATGAAAAATG   2395
DEC03-186       CAATATCAAGATCTGTGATCTATGTTATTGGAAGAATTTTGTCTGTGAGTATGAAAAATG   639
DEC07-98        CAATATCAAGATCTGTGATCTATGTTATTGGAAGAATTTTGTCTGTGAGTATGAAAAATG   1158
APR17-291       ----------------------------------------GTCTGTGAGTATGAAAAATG   20
                                                        ********************

DEC02-76        GTTCTACAAATTAGGAAAACGAGAAAAGATGGATCAATATGAAAAGCAATTCTATCTCAA   3350
CLR03-38395     GTTCTACAAATTAGGAAAACGAGAAAAGATGGATCAATATGAAAAGCAATTCTATCTCAA   2416
CLR01-43699     GTTCTACAAATTAGGAAAACGAGAAAAGATGGATCAATATGAAAAGCAATTCTATCTCAA   5087
APR16-68        GTTCTACAAATTAGGAAAACGAGAAAAGATGGATCAATATGAAAAGCAATTCTATCTCAA   2444
APR18-62        GTTCTACAAATTAGGAAAACGAGAAAAGATGGATCAATATGAAAAGCAATTCTATCTCAA   2486
APR19-43        GTTCTACAAATTAGGAAAACGAGAAAAGATGGATCAATATGAAAAGCAATTCTATCTCAA   2611
APR20-70        GTTCTACAAATTAGGAAAACGAGAAAAGATGGATCAATATGAAAAGCAATTCTATCTCAA   2331
DEC10-249       GTTCTACAAATTAGGAAAACGAGAAAAGATGGATCAATATGAAAAGCAATTCTATCTCAA   495
DEC08-241       GTTCTACAAATTAGGAAAACGAGAAAAGATGGATCAATATGAAAAGCAATTCTATCTCAA   784
DEC06-81        GTTCTACAAATTAGGAAAACGAGAAAAGATGGATCAATATGAAAAGCAATTCTATCTCAA   2496
DEC04-13db      GTTCTACAAATTAGGAAAACGAGAAAAGATGGATCAATATGAAAAGCAATTCTATCTCAA   10020
MAY17-11        GTTCTACAAATTAGGAAAACGAGAAAAGATGGATCAATATGAAAAGCAATTCTATCTCAA   4674
MAY15-78        GTTCTACAAATTAGGAAAACGAGAAAAGATGGATCAATATGAAAAGCAATTCTATCTCAA   2252
JUL05-102       GTTCTACAAATTAGGAAAACGAGAAAAGATGGATCAATATGAAAAGCAATTCTATCTCAA   2241
JUL04-107       GTTCTACAAATTAGGAAAACGAGAAAAGATGGATCAATATGAAAAGCAATTCTATCTCAA   2252
JUL03-55        GTTCTACAAATTAGGAAAACGAGAAAAGATGGATCAATATGAAAAGCAATTCTATCTCAA   2350
JUL02-106       GTTCTACAAATTAGGAAAACGAGAAAAGATGGATCAATATGAAAAGCAATTCTATCTCAA   2241
JUL01-85        GTTCTACAAATTAGGAAAACGAGAAAAGATGGATCAATATGAAAAGCAATTCTATCTCAA   2455
DEC03-186       GTTCTACAAATTAGGAAAACGAGAAAAGATGGATCAATATGAAAAGCAATTCTATCTCAA   699
DEC07-98        GTTCTACAAATTAGGAAAACGAGAAAAGATGGATCAATATGAAAAGCAATTCTATCTCAA   1218
```

```
APR17-291        GTTCTACAAATTAGGAAAACGAGAAAAGATGGATCAATATGAAAAGCAATTCTATCTCAA   80
                 ************************************************************

DEC02-76         GTTTCCACCAACATGGAAAGATGAGTTAGAAAAAATATTTGAAACAGAAAAAGATGCAAG   3410
CLR03-38395      GTTTCCACCAACATGGAAAGATGAGTTAGAAAAAATATTTGAAACAGAAAAAGATGCAAG   2476
CLR01-43699      GTTTCCACCAACATGGAAAGATGAGTTAGAAAAAATATTTGAAACAGAAAAAGATGCAAG   5147
APR16-68         GTTTCCACCAACATGGAAAGATGAGTTAGAAAAAATATTTGAAACAGAAAAAGATGCAAG   2504
APR18-62         GTTTCCACCAACATGGAAAGATGAGTTAGAAAAAATATTTGAAACAGAAAAAGATGCAAG   2546
APR19-43         GTTTCCACCAACATGGAAAGATGAGTTAGAAAAAATATTTGAAACAGAAAAAGATGCAAG   2671
APR20-70         GTTTCCACCAACATGGAAAGATGAGTTAGAAAAAATATTTGAAACAGAAAAAGATGCAAG   2391
DEC10-249        GTTTCCACCAACATGGAAAGATGAGTTAGAAAAAATATTTGAAACAGAAAAAGATGCAAG   555
DEC08-241        GTTTCCACCAACATGGAAAGATGAGTTAGAAAAAATATTTGAAACAGAAAAAGATGCAAG   844
DEC06-81         GTTTCCACCAACATGGAAAGATGAGTTAGAAAAAATATTTGAAACAGAAAAAGATGCAAG   2556
DEC04-13db       GTTTCCACCAACATGGAAAGATGAGTTAGAAAAAATATTTGAAACAGAAAAAGATGCAAG   10080
MAY17-11         GTTTCCACCAACATGGAAAGATGAGTTAGAAAAAATATTTGAAACAGAAAAAGATGCAAG   4734
MAY15-78         GTTTCCACCAACATGGAAAGATGAGTTAGAAAAAATATTTGAAACAGAAAAAGATGCAAG   2312
JUL05-102        GTTTCCACCAACATGGAAAGATGAGTTAGAAAAAATATTTGAAACAGAAAAAGATGCAAG   2301
JUL04-107        GTTTCCACCAACATGGAAAGATGAGTTAGAAAAAATATTTGAAACAGAAAAAGATGCAAG   2312
JUL03-55         GTTTCCACCAACATGGAAAGATGAGTTAGAAAAAATATTTGAAACAGAAAAAGATGCAAG   2410
JUL02-106        GTTTCCACCAACATGGAAAGATGAGTTAGAAAAAATATTTGAAACAGAAAAAGATGCAAG   2301
JUL01-85         GTTTCCACCAACATGGAAAGATGAGTTAGAAAAAATATTTGAAACAGAAAAAGATGCAAG   2515
DEC03-186        GTTTCCACCAACATGGAAAGATGAGTTAGAAAAAATATTTGAAACAGAAAAAGATGCAAG   759
DEC07-98         GTTTCCACCAACATGGAAAGATGAGTTAGAAAAAATATTTGAAACAGAAAAAGATGCAAG   1278
APR17-291        GTTTCCACCAACATGGAAAGATGAGTTAGAAAAAATATTTGAAACAGAAAAAGATGCAAG   140
                 ************************************************************

DEC02-76         ATTAACCAATAATTTGGCAGGAAGAGTACAATGTTTACAGGAATTAATGGAACAAAAATG   3470
CLR03-38395      ATTAACCAATAATTTGGCAGGAAGAGTACAATGTTTACAGGAATTAATGGAACAAAAATG   2536
CLR01-43699      ATTAACCAATAATTTGGCAGGAAGAGTACAATGTTTACAGGAATTAATGGAACAAAAATG   5207
APR16-68         ATTAACCAATAATTTGGCAGGAAGAGTACAATGTTTACAGGAATTAATGGAACAAAAATG   2564
APR18-62         ATTAACCAATAATTTGGCAGGAAGAGTACAATGTTTACAGGAATTAATGGAACAAAAATG   2606
APR19-43         ATTAACCAATAATTTGGCAGGAAGAGTACAATGTTTACAGGAATTAATGGAACAAAAATG   2731
APR20-70         ATTAACCAATAATTTGGCAGGAAGAGTACAATGTTTACAGGAATTAATGGAACAAAAATG   2451
DEC10-249        ATTAACCAATAATTTGGCAGGAAGAGTACAATGTTTACAGGAATTAATGGAACAAAAATG   615
DEC08-241        ATTAACCAATAATTTGGCAGGAAGAGTACAATGTTTACAGGAATTAATGGAACAAAAATG   904
DEC06-81         ATTAACCAATAATTTGGCAGGAAGAGTACAATGTTTACAGGAATTAATGGAACAAAAATG   2616
DEC04-13db       ATTAACCAATAATTTGGCAGGAAGAGTACAATGTTTACAGGAATTAATGGAACAAAAATG   10140
MAY17-11         ATTAACCAATAATTTGGCAGGAAGAGTACAATGTTTACAGGAATTAATGGAACAAAAATG   4794
MAY15-78         ATTAACCAATAATTTGGCAGGAAGAGTACAATGTTTACAGGAATTAATGGAACAAAAATG   2372
JUL05-102        ATTAACCAATAATTTGGCAGGAAGAGTACAATGTTTACAGGAATTAATGGAACAAAAATG   2361
JUL04-107        ATTAACCAATAATTTGGCAGGAAGAGTACAATGTTTACAGGAATTAATGGAACAAAAATG   2372
JUL03-55         ATTAACCAATAATTTGGCAGGAAGAGTACAATGTTTACAGGAATTAATGGAACAAAAATG   2470
JUL02-106        ATTAACCAATAATTTGGCAGGAAGAGTACAATGTTTACAGGAATTAATGGAACAAAAATG   2361
JUL01-85         ATTAACCAATAATTTGGCAGGAAGAGTACAATGTTTACAGGAATTAATGGAACAAAAATG   2575
DEC03-186        ATTAACCAATAATTTGGCAGGAAGAGTACAATGTTTACAGGAATTAATGGAACAAAAATG   819
DEC07-98         ATTAACCAATAATTTGGCAGGAAGAGTACAATGTTTACAGGAATTAATGGAACAAAAATG   1338
APR17-291        ATTAACCAATAATTTGGCAGGAAGAGTACAATGTTTACAGGAATTAATGGAACAAAAATG   200
                 ************************************************************

DEC02-76         TAAAGAAAAATATTTTAATGAGGATGTAACAAGATTCACTAATAAGAAACCCTGTTGTGA   3530
CLR03-38395      TAAAGAAAAATATTTTAATGAGGATGTAACAAGATTCACTAATAAGAAACCCTGTTGTGA   2596
CLR01-43699      TAAAGAAAAATATTTTAATGAGGATGTAACAAGATTCACTAATAAGAAACCCTGTTGTGA   5267
APR16-68         TAAAGAAAAATATTTTAATGAGGATGTAACAAGATTCACTAATAAGAAACCCTGTTGTGA   2624
APR18-62         TAAAGAAAAATATTTTAATGAGGATGTAACAAGATTCACTAATAAGAAACCCTGTTGTGA   2666
APR19-43         TAAAGAAAAATATTTTAATGAGGATGTAACAAGATTCACTAATAAGAAACCCTGTTGTGA   2791
APR20-70         TAAAGAAAAATATTTTAATGAGGATGTAACAAGATTCACTAATAAGAAACCCTGTTGTGA   2511
DEC10-249        TAAAGAAAAATATTTTAATGAGGATGTAACAAGATTCACTAATAAGAAACCCTGTTGTGA   675
DEC08-241        TAAAGAAAAATATTTTAATGAGGATGTAACAAGATTCACTAATAAGAAACCCTGTTGTGA   964
DEC06-81         TAAAGAAAAATATTTTAATGAGGATGTAACAAGATTCACTAATAAGAAACCCTGTTGTGA   2676
DEC04-13db       TAAAGAAAAATATTTTAATGAGGATGTAACAAGATTCACTAATAAGAAACCCTGTTGTGA   10200
MAY17-11         TAAAGAAAAATATTTTAATGAGGATGTAACAAGATTCACTAATAAGAAACCCTGTTGTGA   4854
MAY15-78         TAAAGAAAAATATTTTAATGAGGATGTAACAAGATTCACTAATAAGAAACCCTGTTGTGA   2432
JUL05-102        TAAAGAAAAATATTTTAATGAGGATGTAACAAGATTCACTAATAAGAAACCCTGTTGTGA   2421
```

Figure S4

```
JUL04-107       TAAAGAAAAATATTTTAATGAGGATGTAACAAGATTCACTAATAAGAAACCCTGTTGTGA   2432
JUL03-55        TAAAGAAAAATATTTTAATGAGGATGTAACAAGATTCACTAATAAGAAACCCTGTTGTGA   2530
JUL02-106       TAAAGAAAAATATTTTAATGAGGATGTAACAAGATTCACTAATAAGAAACCCTGTTGTGA   2421
JUL01-85        TAAAGAAAAATATTTTAATGAGGATGTAACAAGATTCACTAATAAGAAACCCTGTTGTGA   2635
DEC03-186       TAAAGAAAAATATTTTAATGAGGATGTAACAAGATTCACTAATAAGAAACCCTGTTGTGA   879
DEC07-98        TAAAGAAAAATATTTTAATGAGGATGTAACAAGATTCACTAATAAGAAACCCTGTTGTGA   1398
APR17-291       TAAAGAAAAATATTTTAATGAGGATGTAACAAGATTCACTAATAAGAAACCCTGTTGTGA   260
                ************************************************************

DEC02-76        TCCTTCGCTAATCGACATTCCTACTAGATGGGGATGTAGACCGCAAAAGGATTATAGGAA   3590
CLR03-38395     TCCTTCGCTAATCGACATTCCTACTAGATGGGGATGTAGACCGCAAAAGGATTATAGGAA   2656
CLR01-43699     TCCTTCGCTAATCGACATTCCTACTAGATGGGGATGTAGACCGCAAAAGGATTATAGGAA   5327
APR16-68        TCCTTCGCTAATCGACATTCCTACTAGATGGGGATGTAGACCGCAAAAGGATTATAGGAA   2684
APR18-62        TCCTTCGCTAATCGACATTCCTACTAGATGGGGATGTAGACCGCAAAAGGATTATAGGAA   2726
APR19-43        TCCTTCGCTAATCGACATTCCTACTAGATGGGGATGTAGACCGCAAAAGGATTATAGGAA   2851
APR20-70        TCCTTCGCTAATCGACATTCCTACTAGATGGGGATGTAGACCGCAAAAGGATTATAGGAA   2571
DEC10-249       TCCTTCGCTAATCGACATTCCTACTAGATGGGGATGTAGACCGCAAAAGGATTATAGGAA   735
DEC08-241       TCCTTCGCTAATCGACATTCCTACTAGATGGGGATGTAGACCGCAAAAGGATTATAGGAA   1024
DEC06-81        TCCTTCGCTAATCGACATTCCTACTAGATGGGGATGTAGACCGCAAAAGGATTATAGGAA   2736
DEC04-13db      TCCTTCGCTAATCGACATTCCTACTAGATGGGGATGTAGACCGCAAAAGGATTATAGGAA   10260
MAY17-11        TCCTTCGCTAATCGACATTCCTACTAGATGGGGATGTAGACCGCAAAAGGATTATAGGAA   4914
MAY15-78        TCCTTCGCTAATCGACATTCCTACTAGATGGGGATGTAGACCGCAAAAGGATTATAGGAA   2492
JUL05-102       TCCTTCGCTAATCGACATTCCTACTAGATGGGGATGTAGACCGCAAAAGGATTATAGGAA   2481
JUL04-107       TCCTTCGCTAATCGACATTCCTACTAGATGGGGATGTAGACCGCAAAAGGATTATAGGAA   2492
JUL03-55        TCCTTCGCTAATCGACATTCCTACTAGATGGGGATGTAGACCGCAAAAGGATTATAGGAA   2590
JUL02-106       TCCTTCGCTAATCGACATTCCTACTAGATGGGGATGTAGACCGCAAAAGGATTATAGGAA   2481
JUL01-85        TCCTTCGCTAATCGACATTCCTACTAGATGGGGATGTAGACCGCAAAAGGATTATAGGAA   2695
DEC03-186       TCCTTCGCTAATCGACATTCCTACTAGATGGGGATGTAGACCGCAAAAGGATTATAGGAA   939
DEC07-98        TCCTTCGCTAATCGACATTCCTACTAGATGGGGATGTAGACCGCAAAAGGATTATAGGAA   1458
APR17-291       TCCTTCGCTAATCGACATTCCTACTAGATGGGGATGTAGACCGCAAAAGGATTATAGGAA   320
                ************************************************************

DEC02-76        ATCTTATAAGACTTATAAGAAAAAAATACAGGAGATATAGGAAGAGGTTTTCACCTCGAAA   3650
CLR03-38395     ATCTTATAAGACTTATAAGAAAAAAATACAGGAGATATAGGAAGAGGTTTTCACCTCGAAA   2716
CLR01-43699     ATCTTATAAGACTTATAAGAAAAAAATACAGGAGATATAGGAAGAGGTTTTCACCTCGAAA   5387
APR16-68        ATCTTATAAGACTTATAAGAAAAAAATACAGGAGATATAGGAAGAGGTTTTCACCTCGAAA   2744
APR18-62        ATCTTATAAGACTTATAAGAAAAAAATACAGGAGATATAGGAAGAGGTTTTCACCTCGAAA   2786
APR19-43        ATCTTATAAGACTTATAAGAAAAAAATACAGGAGATATAGGAAGAGGTTTTCACCTCGAAA   2911
APR20-70        ATCTTATAAGACTTATAAGAAAAAAATACAGGAGATATAGGAAGAGGTTTTCACCTCGAAA   2631
DEC10-249       ATCTTATAAGACTTATAAGAAAAAAATACAGGAGATATAGGAAGAGGTTTTCACCTCGAAA   795
DEC08-241       ATCTTATAAGACTTATAAGAAAAAAATACAGGAGATATAGGAAGAGGTTTTCACCTCGAAA   1084
DEC06-81        ATCTTATAAGACTTATAAGAAAAAAATACAGGAGATATAGGAAGAGGTTTTCACCTCGAAA   2796
DEC04-13db      ATCTTATAAGACTTATAAGAAAAAAATACAGGAGATATAGGAAGAGGTTTTCACCTCGAAA   10320
MAY17-11        ATCTTATAAGACTTATAAGAAAAAAATACAGGAGATATAGGAAGAGGTTTTCACCTCGAAA   4974
MAY15-78        ATCTTATAAGACTTATAAGAAAAAAATACAGGAGATATAGGAAGAGGTTTTCACCTCGAAA   2552
JUL05-102       ATCTTATAAGACTTATAAGAAAAAAATACAGGAGATATAGGAAGAGGTTTTCACCTCGAAA   2541
JUL04-107       ATCTTATAAGACTTATAAGAAAAAAATACAGGAGATATAGGAAGAGGTTTTCACCTCGAAA   2552
JUL03-55        ATCTTATAAGACTTATAAGAAAAAAATACAGGAGATATAGGAAGAGGTTTTCACCTCGAAA   2650
JUL02-106       ATCTTATAAGACTTATAAGAAAAAAATACAGGAGATATAGGAAGAGGTTTTCACCTCGAAA   2541
JUL01-85        ATCTTATAAGACTTATAAGAAAAAAATACAGGAGATATAGGAAGAGGTTTTCACCTCGAAA   2755
DEC03-186       ATCTTATAAGACTTATAAGAAAAAAATACAGGAGATATAGGAAGAGGTTTTCACCTCGAAA   999
DEC07-98        ATCTTATAAGACTTATAAGAAAAAAATACAGGAGATATAGGAAGAGGTTTTCACCTCGAAA   1518
APR17-291       ATCTTATAAGACTTATAAGAAAAAAATACAGGAGATATAGGAAGAGGTTTTCACCTCGAAA   380
                ************************************************************

DEC02-76        ATATAAAAAATATAAAAGGCAATACAGGAAGAAAAAAGGATTTAGAAAGAAAGGACGCCA   3710
CLR03-38395     ATATAAAAAATATAAAAGGCAATACAGGAAGAAAAAAGGATTTAGAAAGAAAGGACGCCA   2776
CLR01-43699     ATATAAAAAATATAAAAGGCAATACAGGAAGAAAAAAGGATTTAGAAAGAAAGGACGCCA   5447
APR16-68        ATATAAAAAATATAAAAGGCAATACAGGAAGAAAAAAGGATTTAGAAAGAAAGGACGCCA   2804
APR18-62        ATATAAAAAATATAAAAGGCAATACAGGAAGAAAAAAGGATTTAGAAAGAAAGGACGCCA   2846
APR19-43        ATATAAAAAATATAAAAGGCAATACAGGAAGAAAAAAGGATTTAGAAAGAAAGGACGCCA   2971
APR20-70        ATATAAAAAATATAAAAGGCAATACAGGAAGAAAAAAGGATTTAGAAAGAAAGGACGCCA   2691
DEC10-249       ATATAAAAAATATAAAAGGCAATACAGGAAGAAAAAAGGATTTAGAAAGAAAGGACGCCA   855
```

```
DEC08-241       ATATAAAAAATATAAAAGGCAATACAGGAAGAAAAAAGGATTTAGAAAGAAAGGACGCCA   1144
DEC06-81        ATATAAAAAATATAAAAGGCAATACAGGAAGAAAAAAGGATTTAGAAAGAAAGGACGCCA   2856
DEC04-13db      ATATAAAAAATATAAAAGGCAATACAGGAAGAAAAAAGGATTTAGAAAGAAAGGACGCCA   10380
MAY17-11        ATATAAAAAATATAAAAGGCAATACAGGAAGAAAAAAGGATTTAGAAAGAAAGGACGCCA   5034
MAY15-78        ATATAAAAAATATAAAAGGCAATACAGGAAGAAAAAAGGATTTAGAAAGAAAGGACGCCA   2612
JUL05-102       ATATAAAAAATATAAAAGGCAATACAGGAAGAAAAAAGGATTTAGAAAGAAAGGACGCCA   2601
JUL04-107       ATATAAAAAATATAAAAGGCAATACAGGAAGAAAAAAGGATTTAGAAAGAAAGGACGCCA   2612
JUL03-55        ATATAAAAAATATAAAAGGCAATACAGGAAGAAAAAAGGATTTAGAAAGAAAGGACGCCA   2710
JUL02-106       ATATAAAAAATATAAAAGGCAATACAGGAAGAAAAAAGGATTTAGAAAGAAAGGACGCCA   2601
JUL01-85        ATATAAAAAATATAAAAGGCAATACAGGAAGAAAAAAGGATTTAGAAAGAAAGGACGCCA   2815
DEC03-186       ATATAAAAAATATAAAAGGCAATACAGGAAGAAAAAAGGATTTAGAAAGAAAGGACGCCA   1059
DEC07-98        ATATAAAAAATATAAAAGGCAATACAGGAAGAAAAAAGGATTTAGAAAGAAAGGACGCCA   1578
APR17-291       ATATAAAAAATATAAAAGGCAATACAGGAAGAAAAAAGGATTTAGAAAGAAAGGACGCCA   440
                ************************************************************

DEC02-76        TAAAAGGAAAAATAGTTATGATGAGCTTCGCGATAACAATTATCGTGATAGTAATAAAAA   3770
CLR03-38395     TAAAAGGAAAAATAGTTATGATGAGCTTCGCGATAACAATTATCGTGATAGTAATAAAAA   2836
CLR01-43699     TAAAAGGAAAAATAGTTATGATGAGCTTCGCGATAACAATTATCGTGATAGTAATAAAAA   5507
APR16-68        TAAAAGGAAAAATAGTTATGATGAGCTTCGCGATAACAATTATCGTGATAGTAATAAAAA   2864
APR18-62        TAAAAGGAAAAATAGTTATGATGAGCTTCGCGATAACAATTATCGTGATAGTAATAAAAA   2906
APR19-43        TAAAAGGAAAAATAGTTATGATGAGCTTCGCGATAACAATTATCGTGATAGTAATAAAAA   3031
APR20-70        TAAAAGGAAAAATAGTTATGATGAGCTTCGCGATAACAATTATCGTGATAGTAATAAAAA   2751
DEC10-249       TAAAAGGAAAAATAGTTATGATGAGCTTCGCGATAACAATTATCGTGATAGTAATAAAAA   915
DEC08-241       TAAAAGGAAAAATAGTTATGATGAGCTTCGCGATAACAATTATCGTGATAGTAATAAAAA   1204
DEC06-81        TAAAAGGAAAAATAGTTATGATGAGCTTCGCGATAACAATTATCGTGATAGTAATAAAAA   2916
DEC04-13db      TAAAAGGAAAAATAGTTATGATGAGCTTCGCGATAACAATTATCGTGATAGTAATAAAAA   10440
MAY17-11        TAAAAGGAAAAATAGTTATGATGAGCTTCGCGATAACAATTATCGTGATAGTAATAAAAA   5094
MAY15-78        TAAAAGGAAAAATAGTTATGATGAGCTTCGCGATAACAATTATCGTGATAGTAATAAAAA   2672
JUL05-102       TAAAAGGAAAAATAGTTATGATGAGCTTCGCGATAACAATTATCGTGATAGTAATAAAAA   2661
JUL04-107       TAAAAGGAAAAATAGTTATGATGAGCTTCGCGATAACAATTATCGTGATAGTAATAAAAA   2672
JUL03-55        TAAAAGGAAAAATAGTTATGATGAGCTTCGCGATAACAATTATCGTGATAGTAATAAAAA   2770
JUL02-106       TAAAAGGAAAAATAGTTATGATGAGCTTCGCGATAACAATTATCGTGATAGTAATAAAAA   2661
JUL01-85        TAAAAGGAAAAATAGTTATGATGAGCTTCGCGATAACAATTATCGTGATAGTAATAAAAA   2875
DEC03-186       TAAAAGGAAAAATAGTTATGATGAGCTTCGCGATAACAATTATCGTGATAGTAATAAAAA   1119
DEC07-98        TAAAAGGAAAAATAGTTATGATGAGCTTCGCGATAACAATTATCGTGATAGTAATAAAAA   1638
APR17-291       TAAAAGGAAAAATAGTTATGATGAGCTTCGCGATAACAATTATCGTGATAGTAATAAAAA   500
                ************************************************************

DEC02-76        ACAGCAGGATTGTCCCAGAAAGAAAAAGGATTGCAGATGTTGGCTCTGCAAAGAGGAAGG   3830
CLR03-38395     ACAGCAGGATTGTCCCAGAAAGAAAAAGGATTGCAGATGTTGGCTCTGCAAAGAGGAAGG   2896
CLR01-43699     ACAGCAGGATTGTCCCAGAAAGAAAAAGGATTGCAGATGTTGGCTCTGCAAAGAGGAAGG   5567
APR16-68        ACAGCAGGATTGTCCCAGAAAGAAAAAGGATTGCAGATGTTGGCTCTGCAAAGAGGAAGG   2924
APR18-62        ACAGCAGGATTGTCCCAGAAAGAAAAAGGATTGCAGATGTTGGCTCTGCAAAGAGGAAGG   2966
APR19-43        ACAGCAGGATTGTCCCAGAAAGAAAAAGGATTGCAGATGTTGGCTCTGCAAAGAGGAAGG   3091
APR20-70        ACAGCAGGATTGTCCCAGAAAGAAAAAGGATTGCAGATGTTGGCTCTGCAAAGAGGAAGG   2811
DEC10-249       ACAGCAGGATTGTCCCAGAAAGAAAAAGGATTGCAGATGTTGGCTCTGCAAAGAGGAAGG   975
DEC08-241       ACAGCAGGATTGTCCCAGAAAGAAAAAGGATTGCAGATGTTGGCTCTGCAAAGAGGAAGG   1264
DEC06-81        ACAGCAGGATTGTCCCAGAAAGAAAAAGGATTGCAGATGTTGGCTCTGCAAAGAGGAAGG   2976
DEC04-13db      ACAGCAGGATTGTCCCAGAAAGAAAAAGGATTGCAGATGTTGGCTCTGCAAAGAGGAAGG   10500
MAY17-11        ACAGCAGGATTGTCCCAGAAAGAAAAAGGATTGCAGATGTTGGCTCTGCAAAGAGGAAGG   5154
MAY15-78        ACAGCAGGATTGTCCCAGAAAGAAAAAGGATTGCAGATGTTGGCTCTGCAAAGAGGAAGG   2732
JUL05-102       ACAGCAGGATTGTCCCAGAAAGAAAAAGGATTGCAGATGTTGGCTCTGCAAAGAGGAAGG   2721
JUL04-107       ACAGCAGGATTGTCCCAGAAAGAAAAAGGATTGCAGATGTTGGCTCTGCAAAGAGGAAGG   2732
JUL03-55        ACAGCAGGATTGTCCCAGAAAGAAAAAGGATTGCAGATGTTGGCTCTGCAAAGAGGAAGG   2830
JUL02-106       ACAGCAGGATTGTCCCAGAAAGAAAAAGGATTGCAGATGTTGGCTCTGCAAAGAGGAAGG   2721
JUL01-85        ACAGCAGGATTGTCCCAGAAAGAAAAAGGATTGCAGATGTTGGCTCTGCAAAGAGGAAGG   2935
DEC03-186       ACAGCAGGATTGTCCCAGAAAGAAAAAGGATTGCAGATGTTGGCTCTGCAAAGAGGAAGG   1179
DEC07-98        ACAGCAGGATTGTCCCAGAAAGAAAAAGGATTGCAGATGTTGGCTCTGCAAAGAGGAAGG   1698
APR17-291       ACAGCAGGATTGTCCCAGAAAGAAAAAGGATTGCAGATGTTGGCTCTGCAAAGAGGAAGG   560
                ************************************************************

DEC02-76        ACATTATGCGAATGAATGTCCACAAAGATATAAAAAAGAGAATAATAAAATAATAAGACA   3890
CLR03-38395     ACATTATGCGAATGAATGTCCACAAAGATATAAAAAAGAGAATAATAAAATAATAAGACA   2956
```

Figure S4

```
CLR01-43699      ACATTATGCGAATGAATGTCCACAAAGATATAAAAAAGAGAATAATAAAATAATAAGACA   5627
APR16-68         ACATTATGCGAATGAATGTCCACAAAGATATAAAAAAGAGAATAATAAAATAATAAGACA   2984
APR18-62         ACATTATGCGAATGAATGTCCACAAAGATATAAAAAAGAGAATAATAAAATAATAAGACA   3026
APR19-43         ACATTATGCGAATGAATGTCCACAAAGATATAAAAAAGAGAATAATAAAATAATAAGACA   3151
APR20-70         ACATTATGCGAATGAATGTCCACAAAGATATAAAAAAGAGAATAATAAAATAATAAGACA   2871
DEC10-249        ACATTATGCGAATGAATGTCCACAAAGATATAAAAAAGAGAATAATAAAATAATAAGACA   1035
DEC08-241        ACATTATGCGAATGAATGTCCACAAAGATATAAAAAAGAGAATAATAAAATAATAAGACA   1324
DEC06-81         ACATTATGCGAATGAATGTCCACAAAGATATAAAAAAGAGAATAATAAAATAATAAGACA   3036
DEC04-13db       ACATTATGCGAATGAATGTCCACAAAGATATAAAAAAGAGAATAATAAAATAATAAGACA   10560
MAY17-11         ACATTATGCGAATGAATGTCCACAAAGATATAAAAAAGAGAATAATAAAATAATAAGACA   5214
MAY15-78         ACATTATGCGAATGAATGTCCACAAAGATATAAAAAAGAGAATAATAAAATAATAAGACA   2792
JUL05-102        ACATTATGCGAATGAATGTCCACAAAGATATAAAAAAGAGAATAATAAAATAATAAGACA   2781
JUL04-107        ACATTATGCGAATGAATGTCCACAAAGATATAAAAAAGAGAATAATAAAATAATAAGACA   2792
JUL03-55         ACATTATGCGAATGAATGTCCACAAAGATATAAAAAAGAGAATAATAAAATAATAAGACA   2890
JUL02-106        ACATTATGCGAATGAATGTCCACAAAGATATAAAAAAGAGAATAATAAAATAATAAGACA   2781
JUL01-85         ACATTATGCGAATGAATGTCCACAAAGATATAAAAAAGAGAATAATAAAATAATAAGACA   2995
DEC03-186        ACATTATGCGAATGAATGTCCACAAAGATATAAAAAAGAGAATAATAAAATAATAAGACA   1239
DEC07-98         ACATTATGCGAATGAATGTCCACAAAGATATAAAAAAGAGAATAATAAAATAATAAGACA   1758
APR17-291        ACATTATGCGAATGAATGTCCACAAAGATATAAAAAAGAGAATAATAAAATAATAAGACA   620
                 ************************************************************

DEC02-76         GTTAGAATACATAAACAGTATATGATATGAACCAGTAGAGTCAGACCCCGACTCAGATGA   3950
CLR03-38395      GTTAGAATACATAAACAGTATATGATATGAACCAGTAGAGTCAGACCCCGACTCAGATGA   3016
CLR01-43699      GTTAGAATACATAAACAGTATATGATATGAACCAGTAGAGTCAGACCCCGACTCAGATGA   5687
APR16-68         GTTAGAATACATAAACAGTATATGATATGAACCAGTAGAGTCAGACCCCGACTCAGATGA   3044
APR18-62         GTTAGAATACATAAACAGTATATGATATGAACCAGTAGAGTCAGACCCCGACTCAGATGA   3086
APR19-43         GTTAGAATACATAAACAGTATATGATATGAACCAGTAGAGTCAGACCCCGACTCAGATGA   3211
APR20-70         GTTAGAATACATAAACAGTATATGATATGAACCAGTAGAGTCAGACCCCGACTCAGATGA   2931
DEC10-249        GTTAGAATACATAAACAGTATATGATATGAACCAGTAGAGTCAGACCCCGACTCAGATGA   1095
DEC08-241        GTTAGAATACATAAACAGTATATGATATGAACCAGTAGAGTCAGACCCCGACTCAGATGA   1384
DEC06-81         GTTAGAATACATAAACAGTATATGATATGAACCAGTAGAGTCAGACCCCGACTCAGATGA   3096
DEC04-13db       GTTAGAATACATAAACAGTATATGATATGAACCAGTAGAGTCAGACCCCGACTCAGATGA   10620
MAY17-11         GTTAGAATACATAAACAGTATATGATATGAACCAGTAGAGTCAGACCCCGACTCAGATGA   5274
MAY15-78         GTTAGAATACATAAACAGTATATGATATGAACCAGTAGAGTCAGACCCCGACTCAGATGA   2852
JUL05-102        GTTAGAATACATAAACAGTATATGATATGAACCAGTAGAGTCAGACCCCGACTCAGATGA   2841
JUL04-107        GTTAGAATACATAAACAGTATATGATATGAACCAGTAGAGTCAGACCCCGACTCAGATGA   2852
JUL03-55         GTTAGAATACATAAACAGTATATGATATGAACCAGTAGAGTCAGACCCCGACTCAGATGA   2950
JUL02-106        GTTAGAATACATAAACAGTATATGATATGAACCAGTAGAGTCAGACCCCGACTCAGATGA   2841
JUL01-85         GTTAGAATACATAAACAGTATATGATATGAACCAGTAGAGTCAGACCCCGACTCAGATGA   3055
DEC03-186        GTTAGAATACATAAACAGTATATGATATGAACCAGTAGAGTCAGACCCCGACTCAGATGA   1299
DEC07-98         GTTAGAATACATAAACAGTATATGATATGAACCAGTAGAGTCAGACCCCGACTCAGATGA   1818
APR17-291        GTTAGAATACATAAACAGTATATGATATGAACCAGTAGAGTCAGACCCCGACTCAGATGA   680
                 ************************************************************

DEC02-76         ATATGTGTATGAATATGACACAGAAGAATAAATAAGAGAGTTTTCTGATGATCCCGGATC   4010
CLR03-38395      ATATGTGTATGAATATGACACAGAAGAATAAATAAGAGAGTTTTCTGATGATCCCGGATC   3076
CLR01-43699      ATATGTGTATGAATATGACACAGAAGAATAAATAAGAGAGTTTTCTGATGATCCCGGATC   5747
APR16-68         ATATGTGTATGAATATGACACAGAAGAATAAATAAGAGAGTTTTCTGATGATCCCGGATC   3104
APR18-62         ATATGTGTATGAATATGACACAGAAGAATAAATAAGAGAGTTTTCTGATGATCCCGGATC   3146
APR19-43         ATATGTGTATGAATATGACACAGAAGAATAAATAAGAGAGTTTTCTGATGATCCCGGATC   3271
APR20-70         ATATGTGTATGAATATGACACAGAAGAATAAATAAGAGAGTTTTCTGATGATCCCGGATC   2991
DEC10-249        ATATGTGTATGAATATGACACAGAAGAATAAATAAGAGAGTTTTCTGATGATCCCGGATC   1155
DEC08-241        ATATGTGTATGAATATGACACAGAAGAATAAATAAGAGAGTTTTCTGATGATCCCGGATC   1444
DEC06-81         ATATGTGTATGAATATGACACAGAAGAATAAATAAGAGAGTTTTCTGATGATCCCGGATC   3156
DEC04-13db       ATATGTGTATGAATATGACACAGAAGAATAAATAAGAGAGTTTTCTGATGATCCCGGATC   10680
MAY17-11         ATATGTGTATGAATATGACACAGAAGAATAAATAAGAGAGTTTTCTGATGATCCCGGATC   5334
MAY15-78         ATATGTGTATGAATATGACACAGAAGAATAAATAAGAGAGTTTTCTGATGATCCCGGATC   2912
JUL05-102        ATATGTGTATGAATATGACACAGAAGAATAAATAAGAGAGTTTTCTGATGATCCCGGATC   2901
JUL04-107        ATATGTGTATGAATATGACACAGAAGAATAAATAAGAGAGTTTTCTGATGATCCCGGATC   2912
JUL03-55         ATATGTGTATGAATATGACACAGAAGAATAAATAAGAGAGTTTTCTGATGATCCCGGATC   3010
JUL02-106        ATATGTGTATGAATATGACACAGAAGAATAAATAAGAGAGTTTTCTGATGATCCCGGATC   2901
JUL01-85         ATATGTGTATGAATATGACACAGAAGAATAAATAAGAGAGTTTTCTGATGATCCCGGATC   3115
DEC03-186        ATATGTGTATGAATATGACACAGAAGAATAAATAAGAGAGTTTTCTGATGATCCCGGATC   1359
```

```
DEC07-98        ATATGTGTATGAATATGACACAGAAGAATAAATAAGAGAGTTTTCTGATGATCCCGGATC   1878
APR17-291       ATATGTGTATGAATATGACACAGAAGAATAAATAAGAGAGTTTTCTGATGATCCCGGATC   740
                ************************************************************

DEC02-76        AGAATGGGAACTCATAGATTAAGACTCGATAAAATTTATCTAGAACAATATCAGGATTAT   4070
CLR03-38395     AGAATGGGAACTCATAGATTAAGACTCGATAAAATTTATCTAGAACAATATCAGGATTAT   3136
CLR01-43699     AGAATGGGAACTCATAGATTAAGACTCGATAAAATTTATCTAGAACAATATCAGGATTAT   5807
APR16-68        AGAATGGGAACTCATAGATTAAGACTCGATAAAATTTATCTAGAACAATATCAGGATTAT   3164
APR18-62        AGAATGGGAACTCATAGATTAAGACTCGATAAAATTTATCTAGAACAATATCAGGATTAT   3206
APR19-43        AGAATGGGAACTCATAGATTAAGACTCGATAAAATTTATCTAGAACAATATCAGGATTAT   3331
APR20-70        AGAATGGGAACTCATAGATTAAGACTCGATAAAATTTATCTAGAACAATATCAGGATTAT   3051
DEC10-249       AGAATGGGAACTCATAGATTAAGACTCGATAAAATTTATCTAGAACAATATCAGGATTAT   1215
DEC08-241       AGAATGGGAACTCATAGATTAAGACTCGATAAAATTTATCTAGAACAATATCAGGATTAT   1504
DEC06-81        AGAATGGGAACTCATAGATTAAGACTCGATAAAATTTATCTAGAACAATATCAGGATTAT   3216
DEC04-13db      AGAATGGGAACTCATAGATTAAGACTCGATAAAATTTATCTAGAACAATATCAGGATTAT   10740
MAY17-11        AGAATGGGAACTCATAGATTAAGACTCGATAAAATTTATCTAGAACAATATCAGGATTAT   5394
MAY15-78        AGAATGGGAACTCATAGATTAAGACTCGATAAAATTTATCTAGAACAATATCAGGATTAT   2972
JUL05-102       AGAATGGGAACTCATAGATTAAGACTCGATAAAATTTATCTAGAACAATATCAGGATTAT   2961
JUL04-107       AGAATGGGAACTCATAGATTAAGACTCGATAAAATTTATCTAGAACAATATCAGGATTAT   2972
JUL03-55        AGAATGGGAACTCATAGATTAAGACTCGATAAAATTTATCTAGAACAATATCAGGATTAT   3070
JUL02-106       AGAATGGGAACTCATAGATTAAGACTCGATAAAATTTATCTAGAACAATATCAGGATTAT   2961
JUL01-85        AGAATGGGAACTCATAGATTAAGACTCGATAAAATTTATCTAGAACAATATCAGGATTAT   3175
DEC03-186       AGAATGGGAACTCATAGATTAAGACTCGATAAAATTTATCTAGAACAATATCAGGATTAT   1419
DEC07-98        AGAATGGGAACTCATAGATTAAGACTCGATAAAATTTATCTAGAACAATATCAGGATTAT   1938
APR17-291       AGAATGGGAACTCATAGATTAAGACTCGATAAAATTTATCTAGAACAATATCAGGATTAT   800
                ************************************************************

DEC02-76        GAATATAATATAACAAATCCTAATAGCACCTACATCAAAGTAGGATTAAAATTCAGAGGA   4130
CLR03-38395     GAATATAATATAACAAATCCTAATAGCACCTACATCAAAGTAGGATTAAAATTCAGAGGA   3196
CLR01-43699     GAATATAATATAACAAATCCTAATAGCACCTACATCAAAGTAGGATTAAAATTCAGAGGA   5867
APR16-68        GAATATAATATAACAAATCCTAATAGCACCTACATCAAAGTAGGATTAAAATTCAGAGGA   3224
APR18-62        GAATATAATATAACAAATCCTAATAGCACCTACATCAAAGTAGGATTAAAATTCAGAGGA   3266
APR19-43        GAATATAATATAACAAATCCTAATAGCACCTACATCAAAGTAGGATTAAAATTCAGAGGA   3391
APR20-70        GAATATAATATAACAAATCCTAATAGCACCTACATCAAAGTAGGATTAAAATTCAGAGGA   3111
DEC10-249       GAATATAATATAACAAATCCTAATAGCACCTACATCAAAGTAGGATTAAAATTCAGAGGA   1275
DEC08-241       GAATATAATATAACAAATCCTAATAGCACCTACATCAAAGTAGGATTAAAATTCAGAGGA   1564
DEC06-81        GAATATAATATAACAAATCCTAATAGCACCTACATCAAAGTAGGATTAAAATTCAGAGGA   3276
DEC04-13db      GAATATAATATAACAAATCCTAATAGCACCTACATCAAAGTAGGATTAAAATTCAGAGGA   10800
MAY17-11        GAATATAATATAACAAATCCTAATAGCACCTACATCAAAGTAGGATTAAAATTCAGAGGA   5454
MAY15-78        GAATATAATATAACAAATCCTAATAGCACCTACATCAAAGTAGGATTAAAATTCAGAGGA   3032
JUL05-102       GAATATAATATAACAAATCCTAATAGCACCTACATCAAAGTAGGATTAAAATTCAGAGGA   3021
JUL04-107       GAATATAATATAACAAATCCTAATAGCACCTACATCAAAGTAGGATTAAAATTCAGAGGA   3032
JUL03-55        GAATATAATATAACAAATCCTAATAGCACCTACATCAAAGTAGGATTAAAATTCAGAGGA   3130
JUL02-106       GAATATAATATAACAAATCCTAATAGCACCTACATCAAAGTAGGATTAAAATTCAGAGGA   3021
JUL01-85        GAATATAATATAACAAATCCTAATAGCACCTACATCAAAGTAGGATTAAAATTCAGAGGA   3235
DEC03-186       GAATATAATATAACAAATCCTAATAGCACCTACATCAAAGTAGGATTAAAATTCAGAGGA   1479
DEC07-98        GAATATAATATAACAAATCCTAATAGCACCTACATCAAAGTAGGATTAAAATTCAGAGGA   1998
APR17-291       GAATATAATATAACAAATCCTAATAGCACCTACATCAAAGTAGGATTAAAATTCAGAGGA   860
                ************************************************************

DEC02-76        TTTAGGTATTATCATTTACATGCCTATATAGATACTGGAGCCAGTATTTGTGTAGCTCAT   4190
CLR03-38395     TTTAGGTATTATCATTTACATGCCTATATAGATACTGGAGCCAGTATTTGTGTAGCTCAT   3256
CLR01-43699     TTTAGGTATTATCATTTACATGCCTATATAGATACTGGAGCCAGTATTTGTGTAGCTCAT   5927
APR16-68        TTTAGGTATTATCATTTACATGCCTATATAGATACTGGAGCCAGTATTTGTGTAGCTCAT   3284
APR18-62        TTTAGGTATTATCATTTACATGCCTATATAGATACTGGAGCCAGTATTTGTGTAGCTCAT   3326
APR19-43        TTTAGGTATTATCATTTACATGCCTATATAGATACTGGAGCCAGTATTTGTGTAGCTCAT   3451
APR20-70        TTTAGGTATTATCATTTACATGCCTATATAGATACTGGAGCCAGTATTTGTGTAGCTCAT   3171
DEC10-249       TTTAGGTATTATCATTTACATGCCTATATAGATACTGGAGCCAGTATTTGTGTAGCTCAT   1335
DEC08-241       TTTAGGTATTATCATTTACATGCCTATATAGATACTGGAGCCAGTATTTGTGTAGCTCAT   1624
DEC06-81        TTTAGGTATTATCATTTACATGCCTATATAGATACTGGAGCCAGTATTTGTGTAGCTCAT   3336
DEC04-13db      TTTAGGTATTATCATTTACATGCCTATATAGATACTGGAGCCAGTATTTGTGTAGCTCAT   10860
MAY17-11        TTTAGGTATTATCATTTACATGCCTATATAGATACTGGAGCCAGTATTTGTGTAGCTCAT   5514
MAY15-78        TTTAGGTATTATCATTTACATGCCTATATAGATACTGGAGCCAGTATTTGTGTAGCTCAT   3092
```

```
JUL05-102        TTTAGGTATTATCATTTACATGCCTATATAGATACTGGAGCCAGTATTTGTGTAGCTCAT   3081
JUL04-107        TTTAGGTATTATCATTTACATGCCTATATAGATACTGGAGCCAGTATTTGTGTAGCTCAT   3092
JUL03-55         TTTAGGTATTATCATTTACATGCCTATATAGATACTGGAGCCAGTATTTGTGTAGCTCAT   3190
JUL02-106        TTTAGGTATTATCATTTACATGCCTATATAGATACTGGAGCCAGTATTTGTGTAGCTCAT   3081
JUL01-85         TTTAGGTATTATCATTTACATGCCTATATAGATACTGGAGCCAGTATTTGTGTAGCTCAT   3295
DEC03-186        TTTAGGTATTATCATTTACATGCCTATATAGATACTGGAGCCAGTATTTGTGTAGCTCAT   1539
DEC07-98         TTTAGGTATTATCATTTACATGCCTATATAGATACTGGAGCCAGTATTTGTGTAGCTCAT   2058
APR17-291        TTTAGGTATTATCATTTACATGCCTATATAGATACTGGAGCCAGTATTTGTGTAGCTCAT   920
                 ************************************************************

DEC02-76         AAGGATGTAATACCAGCAGAAAAATGGGAGGATACGCTGTTTGACATTAATGTTCAAATA   4250
CLR03-38395      AAGGATGTAATACCAGCAGAAAAATGGGAGGATACGCTGTTTGACATTAATGTTCAAATA   3316
CLR01-43699      AAGGATGTAATACCAGCAGAAAAATGGGAGGATACGCTGTTTGACATTAATGTTCAAATA   5987
APR16-68         AAGGATGTAATACCAGCAGAAAAATGGGAGGATACGCTGTTTGACATTAATGTTCAAATA   3344
APR18-62         AAGGATGTAATACCAGCAGAAAAATGGGAGGATACGCTGTTTGACATTAATGTTCAAATA   3386
APR19-43         AAGGATGTAATACCAGCAGAAAAATGGGAGGATACGCTGTTTGACATTAATGTTCAAATA   3511
APR20-70         AAGGATGTAATACCAGCAGAAAAATGGGAGGATACGCTGTTTGACATTAATGTTCAAATA   3231
DEC10-249        AAGGATGTAATACCAGCAGAAAAATGGGAGGATACGCTGTTTGACATTAATGTTCAAATA   1395
DEC08-241        AAGGATGTAATACCAGCAGAAAAATGGGAGGATACGCTGTTTGACATTAATGTTCAAATA   1684
DEC06-81         AAGGATGTAATACCAGCAGAAAAATGGGAGGATACGCTGTTTGACATTAATGTTCAAATA   3396
DEC04-13db       AAGGATGTAATACCAGCAGAAAAATGGGAGGATACGCTGTTTGACATTAATGTTCAAATA   10920
MAY17-11         AAGGATGTAATACCAGCAGAAAAATGGGAGGATACGCTGTTTGACATTAATGTTCAAATA   5574
MAY15-78         AAGGATGTAATACCAGCAGAAAAATGGGAGGATACGCTGTTTGACATTAATGTTCAAATA   3152
JUL05-102        AAGGATGTAATACCAGCAGAAAAATGGGAGGATACGCTGTTTGACATTAATGTTCAAATA   3141
JUL04-107        AAGGATGTAATACCAGCAGAAAAATGGGAGGATACGCTGTTTGACATTAATGTTCAAATA   3152
JUL03-55         AAGGATGTAATACCAGCAGAAAAATGGGAGGATACGCTGTTTGACATTAATGTTCAAATA   3250
JUL02-106        AAGGATGTAATACCAGCAGAAAAATGGGAGGATACGCTGTTTGACATTAATGTTCAAATA   3141
JUL01-85         AAGGATGTAATACCAGCAGAAAAATGGGAGGATACGCTGTTTGACATTAATGTTCAAATA   3355
DEC03-186        AAGGATGTAATACCAGCAGAAAAATGGGAGGATACGCTGTTTGACATTAATGTTCAAATA   1599
DEC07-98         AAGGATGTAATACCAGCAGAAAAATGGGAGGATACGCTGTTTGACATTAATGTTCAAATA   2118
APR17-291        AAGGATGTAATACCAGCAGAAAAATGGGAGGATACGCTGTTTGACATTAATGTTCAAATA   980
                 ************************************************************

DEC02-76         GCTGATAAGAGTATAATCAAGATTAATAAAGTTGCGAGAGATATCTATCTCGAGTTGCAA   4310
CLR03-38395      GCTGATAAGAGTATAATCAAGATTAATAAAGTTGCGAGAGATATCTATCTCGAGTTGCAA   3376
CLR01-43699      GCTGATAAGAGTATAATCAAGATTAATAAAGTTGCGAGAGATATCTATCTCGAGTTGCAA   6047
APR16-68         GCTGATAAGAGTATAATCAAGATTAATAAAGTTGCGAGAGATATCTATCTCGAGTTGCAA   3404
APR18-62         GCTGATAAGAGTATAATCAAGATTAATAAAGTTGCGAGAGATATCTATCTCGAGTTGCAA   3446
APR19-43         GCTGATAAGAGTATAATCAAGATTAATAAAGTTGCGAGAGATATCTATCTCGAGTTGCAA   3571
APR20-70         GCTGATAAGAGTATAATCAAGATTAATAAAGTTGCGAGAGATATCTATCTCGAGTTGCAA   3291
DEC10-249        GCTGATAAGAGTATAATCAAGATTAATAAAGTTGCGAGAGATATCTATCTCGAGTTGCAA   1455
DEC08-241        GCTGATAAGAGTATAATCAAGATTAATAAAGTTGCGAGAGATATCTATCTCGAGTTGCAA   1744
DEC06-81         GCTGATAAGAGTATAATCAAGATTAATAAAGTTGCGAGAGATATCTATCTCGAGTTGCAA   3456
DEC04-13db       GCTGATAAGAGTATAATCAAGATTAATAAAGTTGCGAGAGATATCTATCTCGAGTTGCAA   10980
MAY17-11         GCTGATAAGAGTATAATCAAGATTAATAAAGTTGCGAGAGATATCTATCTCGAGTTGCAA   5634
MAY15-78         GCTGATAAGAGTATAATCAAGATTAATAAAGTTGCGAGAGATATCTATCTCGAGTTGCAA   3212
JUL05-102        GCTGATAAGAGTATAATCAAGATTAATAAAGTTGCGAGAGATATCTATCTCGAGTTGCAA   3201
JUL04-107        GCTGATAAGAGTATAATCAAGATTAATAAAGTTGCGAGAGATATCTATCTCGAGTTGCAA   3212
JUL03-55         GCTGATAAGAGTATAATCAAGATTAATAAAGTTGCGAGAGATATCTATCTCGAGTTGCAA   3310
JUL02-106        GCTGATAAGAGTATAATCAAGATTAATAAAGTTGCGAGAGATATCTATCTCGAGTTGCAA   3201
JUL01-85         GCTGATAAGAGTATAATCAAGATTAATAAAGTTGCGAGAGATATCTATCTCGAGTTGCAA   3415
DEC03-186        GCTGATAAGAGTATAATCAAGATTAATAAAGTTGCGAGAGATATCTATCTCGAGTTGCAA   1659
DEC07-98         GCTGATAAGAGTATAATCAAGATTAATAAAGTTGCGAGAGATATCTATCTCGAGTTGCAA   2178
APR17-291        GCTGATAAGAGTATAATCAAGATTAATAAAGTTGCGAGAGATATCTATCTCGAGTTGCAA   1040
                 ************************************************************

DEC02-76         GGAACGTTGTTTAAGGTCCAAACCTTATATCAACAAAACACAGGAATGGATATTTTAATA   4370
CLR03-38395      GGAACGTTGTTTAAGGTCCAAACCTTATATCAACAAAACACAGGAATGGATATTTTAATA   3436
CLR01-43699      GGAACGTTGTTTAAGGTCCAAACCTTATATCAACAAAACACAGGAATGGATATTTTAATA   6107
APR16-68         GGAACGTTGTTTAAGGTCCAAACCTTATATCAACAAAACACAGGAATGGATATTTTAATA   3464
APR18-62         GGAACGTTGTTTAAGGTCCAAACCTTATATCAACAAAACACAGGAATGGATATTTTAATA   3506
APR19-43         GGAACGTTGTTTAAGGTCCAAACCTTATATCAACAAAACACAGGAATGGATATTTTAATA   3631
APR20-70         GGAACGTTGTTTAAGGTCCAAACCTTATATCAACAAAACACAGGAATGGATATTTTAATA   3351
```

```
DEC10-249       GGAACGTTGTTTAAGGTCCAAACCTTATATCAACAAAACACAGGAATGGATATTTTAATA   1515
DEC08-241       GGAACGTTGTTTAAGGTCCAAACCTTATATCAACAAAACACAGGAATGGATATTTTAATA   1804
DEC06-81        GGAACGTTGTTTAAGGTCCAAACCTTATATCAACAAAACACAGGAATGGATATTTTAATA   3516
DEC04-13db      GGAACGTTGTTTAAGGTCCAAACCTTATATCAACAAAACACAGGAATGGATATTTTAATA   11040
MAY17-11        GGAACGTTGTTTAAGGTCCAAACCTTATATCAACAAAACACAGGAATGGATATTTTAATA   5694
MAY15-78        GGAACGTTGTTTAAGGTCCAAACCTTATATCAACAAAACACAGGAATGGATATTTTAATA   3272
JUL05-102       GGAACGTTGTTTAAGGTCCAAACCTTATATCAACAAAACACAGGAATGGATATTTTAATA   3261
JUL04-107       GGAACGTTGTTTAAGGTCCAAACCTTATATCAACAAAACACAGGAATGGATATTTTAATA   3272
JUL03-55        GGAACGTTGTTTAAGGTCCAAACCTTATATCAACAAAACACAGGAATGGATATTTTAATA   3370
JUL02-106       GGAACGTTGTTTAAGGTCCAAACCTTATATCAACAAAACACAGGAATGGATATTTTAATA   3261
JUL01-85        GGAACGTTGTTTAAGGTCCAAACCTTATATCAACAAAACACAGGAATGGATATTTTAATA   3475
DEC03-186       GGAACGTTGTTTAAGGTCCAAACCTTATATCAACAAAACACAGGAATGGATATTTTAATA   1719
DEC07-98        GGAACGTTGTTTAAGGTCCAAACCTTATATCAACAAAACACAGGAATGGATATTTTAATA   2238
APR17-291       GGAACGTTGTTTAAGGTCCAAACCTTATATCAACAAAACACAGGAATGGATATTTTAATA   1100
                ************************************************************

DEC02-76        TGAAATAATTTCTTACATTTATATCATCCTTTTGTTCAACATTTGGATTATATAACATTA   4430
CLR03-38395     TGAAATAATTTCTTACATTTATATCATCCTTTTGTTCAACATTTGGATTATATAACATTA   3496
CLR01-43699     TGAAATAATTTCTTACATTTATATCATCCTTTTGTTCAACATTTGGATTATATAACATTA   6167
APR16-68        TGAAATAATTTCTTACATTTATATCATCCTTTTGTTCAACATTTGGATTATATAACATTA   3524
APR18-62        TGAAATAATTTCTTACATTTATATCATCCTTTTGTTCAACATTTGGATTATATAACATTA   3566
APR19-43        TGAAATAATTTCTTACATTTATATCATCCTTTTGTTCAACATTTGGATTATATAACATTA   3691
APR20-70        TGAAATAATTTCTTACATTTATATCATCCTTTTGTTCAACATTTGGATTATATAACATTA   3411
DEC10-249       TGAAATAATTTCTTACATTTATATCATCCTTTTGTTCAACATTTGGATTATATAACATTA   1575
DEC08-241       TGAAATAATTTCTTACATTTATATCATCCTTTTGTTCAACATTTGGATTATATAACATTA   1864
DEC06-81        TGAAATAATTTCTTACATTTATATCATCCTTTTGTTCAACATTTGGATTATATAACATTA   3576
DEC04-13db      TGAAATAATTTCTTACATTTATATCATCCTTTTGTTCAACATTTGGATTATATAACATTA   11100
MAY17-11        TGAAATAATTTCTTACATTTATATCATCCTTTTGTTCAACATTTGGATTATATAACATTA   5754
MAY15-78        TGAAATAATTTCTTACATTTATATCATCCTTTTGTTCAACATTTGGATTATATAACATTA   3332
JUL05-102       TGAAATAATTTCTTACATTTATATCATCCTTTTGTTCAACATTTGGATTATATAACATTA   3321
JUL04-107       TGAAATAATTTCTTACATTTATATCATCCTTTTGTTCAACATTTGGATTATATAACATTA   3332
JUL03-55        TGAAATAATTTCTTACATTTATATCATCCTTTTGTTCAACATTTGGATTATATAACATTA   3430
JUL02-106       TGAAATAATTTCTTACATTTATATCATCCTTTTGTTCAACATTTGGATTATATAACATTA   3321
JUL01-85        TGAAATAATTTCTTACATTTATATCATCCTTTTGTTCAACATTTGGATTATATAACATTA   3535
DEC03-186       TGAAATAATTTCTTACATTTATATCATCCTTTTGTTCAACATTTGGATTATATAACATTA   1779
DEC07-98        TGAAATAATTTCTTACATTTATATCATCCTTTTGTTCAACATTTGGATTATATAACATTA   2298
APR17-291       TGAAATAATTTCTTACATTTATATCATCCTTTTGTTCAACATTTGGATTATATAACATTA   1160
                ************************************************************

DEC02-76        AGAAATAATAACAGGACTATTAAAATCCCTAAAGTTAGGAAAGCCTACAGTTGGCATCGA   4490
CLR03-38395     AGAAATAATAACAGGACTATTAAAATCCCTAAAGTTAGGAAAGCCTACAGTTGGCATCGA   3556
CLR01-43699     AGAAATAATAACAGGACTATTAAAATCCCTAAAGTTAGGAAAGCCTACAGTTGGCATCGA   6227
APR16-68        AGAAATAATAACAGGACTATTAAAATCCCTAAAGTTAGGAAAGCCTACAGTTGGCATCGA   3584
APR18-62        AGAAATAATAACAGGACTATTAAAATCCCTAAAGTTAGGAAAGCCTACAGTTGGCATCGA   3626
APR19-43        AGAAATAATAACAGGACTATTAAAATCCCTAAAGTTAGGAAAGCCTACAGTTGGCATCGA   3751
APR20-70        AGAAATAATAACAGGACTATTAAAATCCCTAAAGTTAGGAAAGCCTACAGTTGGCATCGA   3471
DEC10-249       AGAAATAATAACAGGACTATTAAAATCCCTAAAGTTAGGAAAGCCTACAGTTGGCATCGA   1635
DEC08-241       AGAAATAATAACAGGACTATTAAAATCCCTAAAGTTAGGAAAGCCTACAGTTGGCATCGA   1924
DEC06-81        AGAAATAATAACAGGACTATTAAAATCCCTAAAGTTAGGAAAGCCTACAGTTGGCATCGA   3636
DEC04-13db      AGAAATAATAACAGGACTATTAAAATCCCTAAAGTTAGGAAAGCCTACAGTTGGCATCGA   11160
MAY17-11        AGAAATAATAACAGGACTATTAAAATCCCTAAAGTTAGGAAAGCCTACAGTTGGCATCGA   5814
MAY15-78        AGAAATAATAACAGGACTATTAAAATCCCTAAAGTTAGGAAAGCCTACAGTTGGCATCGA   3392
JUL05-102       AGAAATAATAACAGGACTATTAAAATCCCTAAAGTTAGGAAAGCCTACAGTTGGCATCGA   3381
JUL04-107       AGAAATAATAACAGGACTATTAAAATCCCTAAAGTTAGGAAAGCCTACAGTTGGCATCGA   3392
JUL03-55        AGAAATAATAACAGGACTATTAAAATCCCTAAAGTTAGGAAAGCCTACAGTTGGCATCGA   3490
JUL02-106       AGAAATAATAACAGGACTATTAAAATCCCTAAAGTTAGGAAAGCCTACAGTTGGCATCGA   3381
JUL01-85        AGAAATAATAACAGGACTATTAAAATCCCTAAAGTTAGGAAAGCCTACAGTTGGCATCGA   3595
DEC03-186       AGAAATAATAACAGGACTATTAAAATCCCTAAAGTTAGGAAAGCCTACAGTTGGCATCGA   1839
DEC07-98        AGAAATAATAACAGGACTATTAAAATCCCTAAAGTTAGGAAAGCCTACAGTTGGCATCGA   2358
APR17-291       AGAAATAATAACAGGACTATTAAAATCCCTAAAGTTAGGAAAGCCTACAGTTGGCATCGA   1220
                ************************************************************

DEC02-76        CCCGACTTCCTATCAAAGTTACGCATACCGCGAAAGCGTGGTGGGAATAGGAAAAATGAA   4550
```

Figure S4

```
CLR03-38395      CCCGACTTCCTATCAAAGTTACGCATACCGCGAAAGCGTGGTGGGAATAGGAAAAATGAA   3616
CLR01-43699      CCCGACTTCCTATCAAAGTTACGCATACCGCGAAAGCGTGGTGGGAATAGGAAAAATGAA   6287
APR16-68         CCCGACTTCCTATCAAAGTTACGCATACCGCGAAAGCGTGGTGGGAATAGGAAAAATGAA   3644
APR18-62         CCCGACTTCCTATCAAAGTTACGCATACCGCGAAAGCGTGGTGGGAATAGGAAAAATGAA   3686
APR19-43         CCCGACTTCCTATCAAAGTTACGCATACCGCGAAAGCGTGGTGGGAATAGGAAAAATGAA   3811
APR20-70         CCCGACTTCCTATCAAAGTTACGCATACCGCGAAAGCGTGGTGGGAATAGGAAAAATGAA   3531
DEC10-249        CCCGACTTCCTATCAAAGTTACGCATACCGCGAAAGCGTGGTGGGAATAGGAAAAATGAA   1695
DEC08-241        CCCGACTTCCTATCAAAGTTACGCATACCGCGAAAGCGTGGTGGGAATAGGAAAAATGAA   1984
DEC06-81         CCCGACTTCCTATCAAAGTTACGCATACCGCGAAAGCGTGGTGGGAATAGGAAAAATGAA   3696
DEC04-13db       CCCGACTTCCTATCAAAGTTACGCATACCGCGAAAGCGTGGTGGGAATAGGAAAAATGAA   11220
MAY17-11         CCCGACTTCCTATCAAAGTTACGCATACCGCGAAAGCGTGGTGGGAATAGGAAAAATGAA   5874
MAY15-78         CCCGACTTCCTATCAAAGTTACGCATACCGCGAAAGCGTGGTGGGAATAGGAAAAATGAA   3452
JUL05-102        CCCGACTTCCTATCAAAGTTACGCATACCGCGAAAGCGTGGTGGGAATAGGAAAAATGAA   3441
JUL04-107        CCCGACTTCCTATCAAAGTTACGCATACCGCGAAAGCGTGGTGGGAATAGGAAAAATGAA   3452
JUL03-55         CCCGACTTCCTATCAAAGTTACGCATACCGCGAAAGCGTGGTGGGAATAGGAAAAATGAA   3550
JUL02-106        CCCGACTTCCTATCAAAGTTACGCATACCGCGAAAGCGTGGTGGGAATAGGAAAAATGAA   3441
JUL01-85         CCCGACTTCCTATCAAAGTTACGCATACCGCGAAAGCGTGGTGGGAATAGGAAAAATGAA   3655
DEC03-186        CCCGACTTCCTATCAAAGTTACGCATACCGCGAAAGCGTGGTGGGAATAGGAAAAATGAA   1899
DEC07-98         CCCGACTTCCTATCAAAGTTACGCATACCGCGAAAGCGTGGTGGGAATAGGAAAAATGAA   2418
APR17-291        CCCGACTTCCTATCAAAGTTACGCATACCGCGAAAGCGTGGTGGGAATAGGAAAAATGAA   1280
                 ************************************************************

DEC02-76         AATTCAAAAATAAAACAGGATGACTTACCAAAAGTCAATACTACGCCTAATAAAGAAAAT   4610
CLR03-38395      AATTCAAAAATAAAACAGGATGACTTACCAAAAGTCAATACTACGCCTAATAAAGAAAAT   3676
CLR01-43699      AATTCAAAAATAAAACAGGATGACTTACCAAAAGTCAATACTACGCCTAATAAAGAAAAT   6347
APR16-68         AATTCAAAAATAAAACAGGATGACTTACCAAAAGTCAATACTACGCCTAATAAAGAAAAT   3704
APR18-62         AATTCAAAAATAAAACAGGATGACTTACCAAAAGTCAATACTACGCCTAATAAAGAAAAT   3746
APR19-43         AATTCAAAAATAAAACAGGATGACTTACCAAAAGTCAATACTACGCCTAATAAAGAAAAT   3871
APR20-70         AATTCAAAAATAAAACAGGATGACTTACCAAAAGTCAATACTACGCCTAATAAAGAAAAT   3591
DEC10-249        AATTCAAAAATAAAACAGGATGACTTACCAAAAGTCAATACTACGCCTAATAAAGAAAAT   1755
DEC08-241        AATTCAAAAATAAAACAGGATGACTTACCAAAAGTCAATACTACGCCTAATAAAGAAAAT   2044
DEC06-81         AATTCAAAAATAAAACAGGATGACTTACCAAAAGTCAATACTACGCCTAATAAAGAAAAT   3756
DEC04-13db       AATTCAAAAATAAAACAGGATGACTTACCAAAAGTCAATACTACGCCTAATAAAGAAAAT   11280
MAY17-11         AATTCAAAAATAAAACAGGATGACTTACCAAAAGTCAATACTACGCCTAATAAAGAAAAT   5934
MAY15-78         AATTCAAAAATAAAACAGGATGACTTACCAAAAGTCAATACTACGCCTAATAAAGAAAAT   3512
JUL05-102        AATTCAAAAATAAAACAGGATGACTTACCAAAAGTCAATACTACGCCTAATAAAGAAAAT   3501
JUL04-107        AATTCAAAAATAAAACAGGATGACTTACCAAAAGTCAATACTACGCCTAATAAAGAAAAT   3512
JUL03-55         AATTCAAAAATAAAACAGGATGACTTACCAAAAGTCAATACTACGCCTAATAAAGAAAAT   3610
JUL02-106        AATTCAAAAATAAAACAGGATGACTTACCAAAAGTCAATACTACGCCTAATAAAGAAAAT   3501
JUL01-85         AATTCAAAAATAAAACAGGATGACTTACCAAAAGTCAATACTACGCCTAATAAAGAAAAT   3715
DEC03-186        AATTCAAAAATAAAACAGGATGACTTACCAAAAGTCAATACTACGCCTAATAAAGAAAAT   1959
DEC07-98         AATTCAAAAATAAAACAGGATGACTTACCAAAAGTCAATACTACGCCTAATAAAGAAAAT   2478
APR17-291        AATTCAAAAATAAAACAGGATGACTTACCAAAAGTCAATACTACGCCTAATAAAGAAAAT   1340
                 ************************************************************

DEC02-76         GAGGAGTCACAGGAAAATTTAAATCTAGAACAAATGTTCATGATTGAGGATAAAGTCAGA   4670
CLR03-38395      GAGGAGTCACAGGAAAATTTAAATCTAGAACAAATGTTCATGATTGAGGATAAAGTCAGA   3736
CLR01-43699      GAGGAGTCACAGGAAAATTTAAATCTAGAACAAATGTTCATGATTGAGGATAAAGTCAGA   6407
APR16-68         GAGGAGTCACAGGAAAATTTAAATCTAGAACAAATGTTCATGATTGAGGATAAAGTCAGA   3764
APR18-62         GAGGAGTCACAGGAAAATTTAAATCTAGAACAAATGTTCATGATTGAGGATAAAGTCAGA   3806
APR19-43         GAGGAGTCACAGGAAAATTTAAATCTAGAACAAATGTTCATGATTGAGGATAAAGTCAGA   3931
APR20-70         GAGGAGTCACAGGAAAATTTAAATCTAGAACAAATGTTCATGATTGAGGATAAAGTCAGA   3651
DEC10-249        GAGGAGTCACAGGAAAATTTAAATCTAGAACAAATGTTCATGATTGAGGATAAAGTCAGA   1815
DEC08-241        GAGGAGTCACAGGAAAATTTAAATCTAGAACAAATGTTCATGATTGAGGATAAAGTCAGA   2104
DEC06-81         GAGGAGTCACAGGAAAATTTAAATCTAGAACAAATGTTCATGATTGAGGATAAAGTCAGA   3816
DEC04-13db       GAGGAGTCACAGGAAAATTTAAATCTAGAACAAATGTTCATGATTGAGGATAAAGTCAGA   11340
MAY17-11         GAGGAGTCACAGGAAAATTTAAATCTAGAACAAATGTTCATGATTGAGGATAAAGTCAGA   5994
MAY15-78         GAGGAGTCACAGGAAAATTTAAATCTAGAACAAATGTTCATGATTGAGGATAAAGTCAGA   3572
JUL05-102        GAGGAGTCACAGGAAAATTTAAATCTAGAACAAATGTTCATGATTGAGGATAAAGTCAGA   3561
JUL04-107        GAGGAGTCACAGGAAAATTTAAATCTAGAACAAATGTTCATGATTGAGGATAAAGTCAGA   3572
JUL03-55         GAGGAGTCACAGGAAAATTTAAATCTAGAACAAATGTTCATGATTGAGGATAAAGTCAGA   3670
JUL02-106        GAGGAGTCACAGGAAAATTTAAATCTAGAACAAATGTTCATGATTGAGGATAAAGTCAGA   3561
JUL01-85         GAGGAGTCACAGGAAAATTTAAATCTAGAACAAATGTTCATGATTGAGGATAAAGTCAGA   3775
```

Figure S4

```
DEC03-186        GAGGAGTCACAGGAAAATTTAAATCTAGAACAAATGTTCATGATTGAGGATAAAGTCAGA   2019
DEC07-98         GAGGAGTCACAGGAAAATTTAAATCTAGAACAAATGTTCATGATTGAGGATAAAGTCAGA   2538
APR17-291        GAGGAGTCACAGGAAAATTTAAATCTAGAACAAATGTTCATGATTGAGGATAAAGTCAGA   1400
                 ************************************************************

DEC02-76         CAAATATTAAAAGAAATAAGTTCATTGAATCCACTAGATTCAAGGATAACGTGTTGTAAT   4730
CLR03-38395      CAAATATTAAAAGAAATAAGTTCATTGAATCCACTAGATTCAAGGATAACGTGTTGTAAT   3796
CLR01-43699      CAAATATTAAAAGAAATAAGTTCATTGAATCCACTAGATTCAAGGATAACGTGTTGTAAT   6467
APR16-68         CAAATATTAAAAGAAATAAGTTCATTGAATCCACTAGATTCAAGGATAACGTGTTGTAAT   3824
APR18-62         CAAATATTAAAAGAAATAAGTTCATTGAATCCACTAGATTCAAGGATAACGTGTTGTAAT   3866
APR19-43         CAAATATTAAAAGAAATAAGTTCATTGAATCCACTAGATTCAAGGATAACGTGTTGTAAT   3991
APR20-70         CAAATATTAAAAGAAATAAGTTCATTGAATCCACTAGATTCAAGGATAACGTGTTGTAAT   3711
DEC10-249        CAAATATTAAAAGAAATAAGTTCATTGAATCCACTAGATTCAAGGATAACGTGTTGTAAT   1875
DEC08-241        CAAATATTAAAAGAAATAAGTTCATTGAATCCACTAGATTCAAGGATAACGTGTTGTAAT   2164
DEC06-81         CAAATATTAAAAGAAATAAGTTCATTGAATCCACTAGATTCAAGGATAACGTGTTGTAAT   3876
DEC04-13db       CAAATATTAAAAGAAATAAGTTCATTGAATCCACTAGATTCAAGGATAACGTGTTGTAAT   11400
MAY17-11         CAAATATTAAAAGAAATAAGTTCATTGAATCCACTAGATTCAAGGATAACGTGTTGTAAT   6054
MAY15-78         CAAATATTAAAAGAAATAAGTTCATTGAATCCACTAGATTCAAGGATAACGTGTTGTAAT   3632
JUL05-102        CAAATATTAAAAGAAATAAGTTCATTGAATCCACTAGATTCAAGGATAACGTGTTGTAAT   3621
JUL04-107        CAAATATTAAAAGAAATAAGTTCATTGAATCCACTAGATTCAAGGATAACGTGTTGTAAT   3632
JUL03-55         CAAATATTAAAAGAAATAAGTTCATTGAATCCACTAGATTCAAGGATAACGTGTTGTAAT   3730
JUL02-106        CAAATATTAAAAGAAATAAGTTCATTGAATCCACTAGATTCAAGGATAACGTGTTGTAAT   3621
JUL01-85         CAAATATTAAAAGAAATAAGTTCATTGAATCCACTAGATTCAAGGATAACGTGTTGTAAT   3835
DEC03-186        CAAATATTAAAAGAAATAAGTTCATTGAATCCACTAGATTCAAGGATAACGTGTTGTAAT   2079
DEC07-98         CAAATATTAAAAGAAATAAGTTCATTGAATCCACTAGATTCAAGGATAACGTGTTGTAAT   2598
APR17-291        CAAATATTAAAAGAAATAAGTTCATTGAATCCACTAGATTCAAGGATAACGTGTTGTAAT   1460
                 ************************************************************

DEC02-76         CTTTTTAAAAGATCACGACTCGAAGCAGAGATAAAACTAAAAGATGAAAAGAAAGTCATT   4790
CLR03-38395      CTTTTTAAAAGATCACGACTCGAAGCAGAGATAAAACTAAAAGATGAAAAGAAAGTCATT   3856
CLR01-43699      CTTTTTAAAAGATCACGACTCGAAGCAGAGATAAAACTAAAAGATGAAAAGAAAGTCATT   6527
APR16-68         CTTTTTAAAAGATCACGACTCGAAGCAGAGATAAAACTAAAAGATGAAAAGAAAGTCATT   3884
APR18-62         CTTTTTAAAAGATCACGACTCGAAGCAGAGATAAAACTAAAAGATGAAAAGAAAGTCATT   3926
APR19-43         CTTTTTAAAAGATCACGACTCGAAGCAGAGATAAAACTAAAAGATGAAAAGAAAGTCATT   4051
APR20-70         CTTTTTAAAAGATCACGACTCGAAGCAGAGATAAAACTAAAAGATGAAAAGAAAGTCATT   3771
DEC10-249        CTTTTTAAAAGATCACGACTCGAAGCAGAGATAAAACTAAAAGATGAAAAGAAAGTCATT   1935
DEC08-241        CTTTTTAAAAGATCACGACTCGAAGCAGAGATAAAACTAAAAGATGAAAAGAAAGTCATT   2224
DEC06-81         CTTTTTAAAAGATCACGACTCGAAGCAGAGATAAAACTAAAAGATGAAAAGAAAGTCATT   3936
DEC04-13db       CTTTTTAAAAGATCACGACTCGAAGCAGAGATAAAACTAAAAGATGAAAAGAAAGTCATT   11460
MAY17-11         CTTTTTAAAAGATCACGACTCGAAGCAGAGATAAAACTAAAAGATGAAAAGAAAGTCATT   6114
MAY15-78         CTTTTTAAAAGATCACGACTCGAAGCAGAGATAAAACTAAAAGATGAAAAGAAAGTCATT   3692
JUL05-102        CTTTTTAAAAGATCACGACTCGAAGCAGAGATAAAACTAAAAGATGAAAAGAAAGTCATT   3681
JUL04-107        CTTTTTAAAAGATCACGACTCGAAGCAGAGATAAAACTAAAAGATGAAAAGAAAGTCATT   3692
JUL03-55         CTTTTTAAAAGATCACGACTCGAAGCAGAGATAAAACTAAAAGATGAAAAGAAAGTCATT   3790
JUL02-106        CTTTTTAAAAGATCACGACTCGAAGCAGAGATAAAACTAAAAGATGAAAAGAAAGTCATT   3681
JUL01-85         CTTTTTAAAAGATCACGACTCGAAGCAGAGATAAAACTAAAAGATGAAAAGAAAGTCATT   3895
DEC03-186        CTTTTTAAAAGATCACGACTCGAAGCAGAGATAAAACTAAAAGATGAAAAGAAAGTCATT   2139
DEC07-98         CTTTTTAAAAGATCACGACTCGAAGCAGAGATAAAACTAAAAGATGAAAAGAAAGTCATT   2658
APR17-291        CTTTTTAAAAGATCACGACTCGAAGCAGAGATAAAACTAAAAGATGAAAAGAAAGTCATT   1520
                 ************************************************************

DEC02-76         AGGGTAAAACCAATGATTTATACATCTCAGGATAAGATTGAGTTTAATAAACAAATCATG   4850
CLR03-38395      AGGGTAAAACCAATGATTTATACATCTCAGGATAAGATTGAGTTTAATAAACAAATCATG   3916
CLR01-43699      AGGGTAAAACCAATGATTTATACATCTCAGGATAAGATTGAGTTTAATAAACAAATCATG   6587
APR16-68         AGGGTAAAACCAATGATTTATACATCTCAGGATAAGATTGAGTTTAATAAACAAATCATG   3944
APR18-62         AGGGTAAAACCAATGATTTATACATCTCAGGATAAGATTGAGTTTAATAAACAAATCATG   3986
APR19-43         AGGGTAAAACCAATGATTTATACATCTCAGGATAAGATTGAGTTTAATAAACAAATCATG   4111
APR20-70         AGGGTAAAACCAATGATTTATACATCTCAGGATAAGATTGAGTTTAATAAACAAATCATG   3831
DEC10-249        AGGGTAAAACCAATGATTTATACATCTCAGGATAAGATTGAGTTTAATAAACAAATCATG   1995
DEC08-241        AGGGTAAAACCAATGATTTATACATCTCAGGATAAGATTGAGTTTAATAAACAAATCATG   2284
DEC06-81         AGGGTAAAACCAATGATTTATACATCTCAGGATAAGATTGAGTTTAATAAACAAATCATG   3996
DEC04-13db       AGGGTAAAACCAATGATTTATACATCTCAGGATAAGATTGAGTTTAATAAACAAATCATG   11520
MAY17-11         AGGGTAAAACCAATGATTTATACATCTCAGGATAAGATTGAGTTTAATAAACAAATCATG   6174
```

```
MAY15-78        AGGGTAAAACCAATGATTTATACATCTCAGGATAAGATTGAGTTTAATAAACAAATCATG   3752
JUL05-102       AGGGTAAAACCAATGATTTATACATCTCAGGATAAGATTGAGTTTAATAAACAAATCATG   3741
JUL04-107       AGGGTAAAACCAATGATTTATACATCTCAGGATAAGATTGAGTTTAATAAACAAATCATG   3752
JUL03-55        AGGGTAAAACCAATGATTTATACATCTCAGGATAAGATTGAGTTTAATAAACAAATCATG   3850
JUL02-106       AGGGTAAAACCAATGATTTATACATCTCAGGATAAGATTGAGTTTAATAAACAAATCATG   3741
JUL01-85        AGGGTAAAACCAATGATTTATACATCTCAGGATAAGATTGAGTTTAATAAACAAATCATG   3955
DEC03-186       AGGGTAAAACCAATGATTTATACATCTCAGGATAAGATTGAGTTTAATAAACAAATCATG   2199
DEC07-98        AGGGTAAAACCAATGATTTATACATCTCAGGATAAGATTGAGTTTAATAAACAAATCATG   2718
APR17-291       AGGGTAAAACCAATGATTTATACATCTCAGGATAAGATTGAGTTTAATAAACAAATCATG   1580
                ************************************************************

DEC02-76        GAACTACTAGAGTTAGATTTAATCCGACCTAGTAAATCACCACACAGTTCACCAGCATTT   4910
CLR03-38395     GAACTACTAGAGTTAGATTTAATCCGACCTAGTAAATCACCACACAGTTCACCAGCATTT   3976
CLR01-43699     GAACTACTAGAGTTAGATTTAATCCGACCTAGTAAATCACCACACAGTTCACCAGCATTT   6647
APR16-68        GAACTACTAGAGTTAGATTTAATCCGACCTAGTAAATCACCACACAGTTCACCAGCATTT   4004
APR18-62        GAACTACTAGAGTTAGATTTAATCCGACCTAGTAAATCACCACACAGTTCACCAGCATTT   4046
APR19-43        GAACTACTAGAGTTAGATTTAATCCGACCTAGTAAATCACCACACAGTTCACCAGCATTT   4171
APR20-70        GAACTACTAGAGTTAGATTTAATCCGACCTAGTAAATCACCACACAGTTCACCAGCATTT   3891
DEC10-249       GAACTACTAGAGTTAGATTTAATCCGACCTAGTAAATCACCACACAGTTCACCAGCATTT   2055
DEC08-241       GAACTACTAGAGTTAGATTTAATCCGACCTAGTAAATCACCACACAGTTCACCAGCATTT   2344
DEC06-81        GAACTACTAGAGTTAGATTTAATCCGACCTAGTAAATCACCACACAGTTCACCAGCATTT   4056
DEC04-13db      GAACTACTAGAGTTAGATTTAATCCGACCTAGTAAATCACCACACAGTTCACCAGCATTT   11580
MAY17-11        GAACTACTAGAGTTAGATTTAATCCGACCTAGTAAATCACCACACAGTTCACCAGCATTT   6234
MAY15-78        GAACTACTAGAGTTAGATTTAATCCGACCTAGTAAATCACCACACAGTTCACCAGCATTT   3812
JUL05-102       GAACTACTAGAGTTAGATTTAATCCGACCTAGTAAATCACCACACAGTTCACCAGCATTT   3801
JUL04-107       GAACTACTAGAGTTAGATTTAATCCGACCTAGTAAATCACCACACAGTTCACCAGCATTT   3812
JUL03-55        GAACTACTAGAGTTAGATTTAATCCGACCTAGTAAATCACCACACAGTTCACCAGCATTT   3910
JUL02-106       GAACTACTAGAGTTAGATTTAATCCGACCTAGTAAATCACCACACAGTTCACCAGCATTT   3801
JUL01-85        GAACTACTAGAGTTAGATTTAATCCGACCTAGTAAATCACCACACAGTTCACCAGCATTT   4015
DEC03-186       GAACTACTAGAGTTAGATTTAATCCGACCTAGTAAATCACCACACAGTTCACCAGCATTT   2259
DEC07-98        GAACTACTAGAGTTAGATTTAATCCGACCTAGTAAATCACCACACAGTTCACCAGCATTT   2778
APR17-291       GAACTACTAGAGTTAGATTTAATCCGACCTAGTAAATCACCACACAGTTCACCAGCATTT   1640
                ************************************************************

DEC02-76        CTCGTAGAGAATCATGCTGAGAAGAAAAGGAATAAGAAAAGAATGGTAATAAATTATAAA   4970
CLR03-38395     CTCGTAGAGAATCATGCTGAGAAGAAAAGGAATAAGAAAAGAATGGTAATAAATTATAAA   4036
CLR01-43699     CTCGTAGAGAATCATGCTGAGAAGAAAAGGAATAAGAAAAGAATGGTAATAAATTATAAA   6707
APR16-68        CTCGTAGAGAATCATGCTGAGAAGAAAAGGAATAAGAAAAGAATGGTAATAAATTATAAA   4064
APR18-62        CTCGTAGAGAATCATGCTGAGAAGAAAAGGAATAAGAAAAGAATGGTAATAAATTATAAA   4106
APR19-43        CTCGTAGAGAATCATGCTGAGAAGAAAAGGAATAAGAAAAGAATGGTAATAAATTATAAA   4231
APR20-70        CTCGTAGAGAATCATGCTGAGAAGAAAAGGAATAAGAAAAGAATGGTAATAAATTATAAA   3951
DEC10-249       CTCGTAGAGAATCATGCTGAGAAGAAAAGGAATAAGAAAAGAATGGTAATAAATTATAAA   2115
DEC08-241       CTCGTAGAGAATCATGCTGAGAAGAAAAGGAATAAGAAAAGAATGGTAATAAATTATAAA   2404
DEC06-81        CTCGTAGAGAATCATGCTGAGAAGAAAAGGAATAAGAAAAGAATGGTAATAAATTATAAA   4116
DEC04-13db      CTCGTAGAGAATCATGCTGAGAAGAAAAGGAATAAGAAAAGAATGGTAATAAATTATAAA   11640
MAY17-11        CTCGTAGAGAATCATGCTGAGAAGAAAAGGAATAAGAAAAGAATGGTAATAAATTATAAA   6294
MAY15-78        CTCGTAGAGAATCATGCTGAGAAGAAAAGGAATAAGAAAAGAATGGTAATAAATTATAAA   3872
JUL05-102       CTCGTAGAGAATCATGCTGAGAAGAAAAGGAATAAGAAAAGAATGGTAATAAATTATAAA   3861
JUL04-107       CTCGTAGAGAATCATGCTGAGAAGAAAAGGAATAAGAAAAGAATGGTAATAAATTATAAA   3872
JUL03-55        CTCGTAGAGAATCATGCTGAGAAGAAAAGGAATAAGAAAAGAATGGTAATAAATTATAAA   3970
JUL02-106       CTCGTAGAGAATCATGCTGAGAAGAAAAGGAATAAGAAAAGAATGGTAATAAATTATAAA   3861
JUL01-85        CTCGTAGAGAATCATGCTGAGAAGAAAAGGAATAAGAAAAGAATGGTAATAAATTATAAA   4075
DEC03-186       CTCGTAGAGAATCATGCTGAGAAGAAAAGGAATAAGAAAAGAATGGTAATAAATTATAAA   2319
DEC07-98        CTCGTAGAGAATCATGCTGAGAAGAAAAGGAATAAGAAAAGAATGGTAATAAATTATAAA   2838
APR17-291       CTCGTAGAGAATCATGCTGAGAAGAAAAGGAATAAGAAAAGAATGGTAATAAATTATAAA   1700
                ************************************************************

DEC02-76        GCATTAAACAAAGAAACAATTGATGATGGGTATTATTTACCTAAAAAAGATGAATTAATA   5030
CLR03-38395     GCATTAAACAAAGAAACAATTGATGATGGGTATTATTTACCTAAAAAAGATGAATTAATA   4096
CLR01-43699     GCATTAAACAAAGAAACAATTGATGATGGGTATTATTTACCTAAAAAAGATGAATTAATA   6767
APR16-68        GCATTAAACAAAGAAACAATTGATGATGGGTATTATTTACCTAAAAAAGATGAATTAATA   4124
APR18-62        GCATTAAACAAAGAAACAATTGATGATGGGTATTATTTACCTAAAAAAGATGAATTAATA   4166
APR19-43        GCATTAAACAAAGAAACAATTGATGATGGGTATTATTTACCTAAAAAAGATGAATTAATA   4291
```

```
APR20-70        GCATTAAACAAAGAAACAATTGATGATGGGTATTATTTACCTAAAAAAGATGAATTAATA   4011
DEC10-249       GCATTAAACAAAGAAACAATTGATGATGGGTATTATTTACCTAAAAAAGATGAATTAATA   2175
DEC08-241       GCATTAAACAAAGAAACAATTGATGATGGGTATTATTTACCTAAAAAAGATGAATTAATA   2464
DEC06-81        GCATTAAACAAAGAAACAATTGATGATGGGTATTATTTACCTAAAAAAGATGAATTAATA   4176
DEC04-13db      GCATTAAACAAAGAAACAATTGATGATGGGTATTATTTACCTAAAAAAGATGAATTAATA   11700
MAY17-11        GCATTAAACAAAGAAACAATTGATGATGGGTATTATTTACCTAAAAAAGATGAATTAATA   6354
MAY15-78        GCATTAAACAAAGAAACAATTGATGATGGGTATTATTTACCTAAAAAAGATGAATTAATA   3932
JUL05-102       GCATTAAACAAAGAAACAATTGATGATGGGTATTATTTACCTAAAAAAGATGAATTAATA   3921
JUL04-107       GCATTAAACAAAGAAACAATTGATGATGGGTATTATTTACCTAAAAAAGATGAATTAATA   3932
JUL03-55        GCATTAAACAAAGAAACAATTGATGATGGGTATTATTTACCTAAAAAAGATGAATTAATA   4030
JUL02-106       GCATTAAACAAAGAAACAATTGATGATGGGTATTATTTACCTAAAAAAGATGAATTAATA   3921
JUL01-85        GCATTAAACAAAGAAACAATTGATGATGGGTATTATTTACCTAAAAAAGATGAATTAATA   4135
DEC03-186       GCATTAAACAAAGAAACAATTGATGATGGGTATTATTTACCTAAAAAAGATGAATTAATA   2379
DEC07-98        GCATTAAACAAAGAAACAATTGATGATGGGTATTATTTACCTAAAAAAGATGAATTAATA   2898
APR17-291       GCATTAAACAAAGAAACAATTGATGATGGGTATTATTTACCTAAAAAAGATGAATTAATA   1760
                ************************************************************

DEC02-76        AAGTTAATCTCAGGAAAACAATGGTATAGCAGTTTCGACTGTAAGTCAGGATTCTGGCAG   5090
CLR03-38395     AAGTTAATCTCAGGAAAACAATGGTATAGCAGTTTCGACTGTAAGTCAGGATTCTGGCAG   4156
CLR01-43699     AAGTTAATCTCAGGAAAACAATGGTATAGCAGTTTCGACTGTAAGTCAGGATTCTGGCAG   6827
APR16-68        AAGTTAATCTCAGGAAAACAATGGTATAGCAGTTTCGACTGTAAGTCAGGATTCTGGCAG   4184
APR18-62        AAGTTAATCTCAGGAAAACAATGGTATAGCAGTTTCGACTGTAAGTCAGGATTCTGGCAG   4226
APR19-43        AAGTTAATCTCAGGAAAACAATGGTATAGCAGTTTCGACTGTAAGTCAGGATTCTGGCAG   4351
APR20-70        AAGTTAATCTCAGGAAAACAATGGTATAGCAGTTTCGACTGTAAGTCAGGATTCTGGCAG   4071
DEC10-249       AAGTTAATCTCAGGAAAACAATGGTATAGCAGTTTCGACTGTAAGTCAGGATTCTGGCAG   2235
DEC08-241       AAGTTAATCTCAGGAAAACAATGGTATAGCAGTTTCGACTGTAAGTCAGGATTCTGGCAG   2524
DEC06-81        AAGTTAATCTCAGGAAAACAATGGTATAGCAGTTTCGACTGTAAGTCAGGATTCTGGCAG   4236
DEC04-13db      AAGTTAATCTCAGGAAAACAATGGTATAGCAGTTTCGACTGTAAGTCAGGATTCTGGCAG   11760
MAY17-11        AAGTTAATCTCAGGAAAACAATGGTATAGCAGTTTCGACTGTAAGTCAGGATTCTGGCAG   6414
MAY15-78        AAGTTAATCTCAGGAAAACAATGGTATAGCAGTTTCGACTGTAAGTCAGGATTCTGGCAG   3992
JUL05-102       AAGTTAATCTCAGGAAAACAATGGTATAGCAGTTTCGACTGTAAGTCAGGATTCTGGCAG   3981
JUL04-107       AAGTTAATCTCAGGAAAACAATGGTATAGCAGTTTCGACTGTAAGTCAGGATTCTGGCAG   3992
JUL03-55        AAGTTAATCTCAGGAAAACAATGGTATAGCAGTTTCGACTGTAAGTCAGGATTCTGGCAG   4090
JUL02-106       AAGTTAATCTCAGGAAAACAATGGTATAGCAGTTTCGACTGTAAGTCAGGATTCTGGCAG   3981
JUL01-85        AAGTTAATCTCAGGAAAACAATGGTATAGCAGTTTCGACTGTAAGTCAGGATTCTGGCAG   4195
DEC03-186       AAGTTAATCTCAGGAAAACAATGGTATAGCAGTTTCGACTGTAAGTCAGGATTCTGGCAG   2439
DEC07-98        AAGTTAATCTCAGGAAAACAATGGTATAGCAGTTTCGACTGTAAGTCAGGATTCTGGCAG   2958
APR17-291       AAGTTAATCTCAGGAAAACAATGGTATAGCAGTTTCGACTGTAAGTCAGGATTCTGGCAG   1820
                ************************************************************

DEC02-76        GTACCATTAAAGGAAAGTTGTAAAAAACTAACAGCTTTTAGCTGTCCCTCAGGACAATTT   5150
CLR03-38395     GTACCATTAAAGGAAAGTTGTAAAAAACTAACAGCTTTTAGCTGTCCCTCAGGACAATTT   4216
CLR01-43699     GTACCATTAAAGGAAAGTTGTAAAAAACTAACAGCTTTTAGCTGTCCCTCAGGACAATTT   6887
APR16-68        GTACCATTAAAGGAAAGTTGTAAAAAACTAACAGCTTTTAGCTGTCCCTCAGGACAATTT   4244
APR18-62        GTACCATTAAAGGAAAGTTGTAAAAAACTAACAGCTTTTAGCTGTCCCTCAGGACAATTT   4286
APR19-43        GTACCATTAAAGGAAAGTTGTAAAAAACTAACAGCTTTTAGCTGTCCCTCAGGACAATTT   4411
APR20-70        GTACCATTAAAGGAAAGTTGTAAAAAACTAACAGCTTTTAGCTGTCCCTCAGGACAATTT   4131
DEC10-249       GTACCATTAAAGGAAAGTTGTAAAAAACTAACAGCTTTTAGCTGTCCCTCAGGACAATTT   2295
DEC08-241       GTACCATTAAAGGAAAGTTGTAAAAAACTAACAGCTTTTAGCTGTCCCTCAGGACAATTT   2584
DEC06-81        GTACCATTAAAGGAAAGTTGTAAAAAACTAACAGCTTTTAGCTGTCCCTCAGGACAATTT   4296
DEC04-13db      GTACCATTAAAGGAAAGTTGTAAAAAACTAACAGCTTTTAGCTGTCCCTCAGGACAATTT   11820
MAY17-11        GTACCATTAAAGGAAAGTTGTAAAAAACTAACAGCTTTTAGCTGTCCCTCAGGACAATTT   6474
MAY15-78        GTACCATTAAAGGAAAGTTGTAAAAAACTAACAGCTTTTAGCTGTCCCTCAGGACAATTT   4052
JUL05-102       GTACCATTAAAGGAAAGTTGTAAAAAACTAACAGCTTTTAGCTGTCCCTCAGGACAATTT   4041
JUL04-107       GTACCATTAAAGGAAAGTTGTAAAAAACTAACAGCTTTTAGCTGTCCCTCAGGACAATTT   4052
JUL03-55        GTACCATTAAAGGAAAGTTGTAAAAAACTAACAGCTTTTAGCTGTCCCTCAGGACAATTT   4150
JUL02-106       GTACCATTAAAGGAAAGTTGTAAAAAACTAACAGCTTTTAGCTGTCCCTCAGGACAATTT   4041
JUL01-85        GTACCATTAAAGGAAAGTTGTAAAAAACTAACAGCTTTTAGCTGTCCCTCAGGACAATTT   4255
DEC03-186       GTACCATTAAAGGAAAGTTGTAAAAAACTAACAGCTTTTAGCTGTCCCTCAGGACAATTT   2499
DEC07-98        GTACCATTAAAGGAAAGTTGTAAAAAACTAACAGCTTTTAGCTGTCCCTCAGGACAATTT   3018
APR17-291       GTACCATTAAAGGAAAGTTGTAAAAAACTAACAGCTTTTAGCTGTCCCTCAGGACAATTT   1880
                ************************************************************
```

```
DEC02-76        GAGTGGAATGTACTTCCATTTGGATTAAAACAAGCGCCAGGAATATTTCAAAGAAAAATG   5210
CLR03-38395     GAGTGGAATGTACTTCCATTTGGATTAAAACAAGCGCCAGGAATATTTCAAAGAAAAATG   4276
CLR01-43699     GAGTGGAATGTACTTCCATTTGGATTAAAACAAGCGCCAGGAATATTTCAAAGAAAAATG   6947
APR16-68        GAGTGGAATGTACTTCCATTTGGATTAAAACAAGCGCCAGGAATATTTCAAAGAAAAATG   4304
APR18-62        GAGTGGAATGTACTTCCATTTGGATTAAAACAAGCGCCAGGAATATTTCAAAGAAAAATG   4346
APR19-43        GAGTGGAATGTACTTCCATTTGGATTAAAACAAGCGCCAGGAATATTTCAAAGAAAAATG   4471
APR20-70        GAGTGGAATGTACTTCCATTTGGATTAAAACAAGCGCCAGGAATATTTCAAAGAAAAATG   4191
DEC10-249       GAGTGGAATGTACTTCCATTTGGATTAAAACAAGCGCCAGGAATATTTCAAAGAAAAATG   2355
DEC08-241       GAGTGGAATGTACTTCCATTTGGATTAAAACAAGCGCCAGGAATATTTCAAAGAAAAATG   2644
DEC06-81        GAGTGGAATGTACTTCCATTTGGATTAAAACAAGCGCCAGGAATATTTCAAAGAAAAATG   4356
DEC04-13db      GAGTGGAATGTACTTCCATTTGGATTAAAACAAGCGCCAGGAATATTTCAAAGAAAAATG   11880
MAY17-11        GAGTGGAATGTACTTCCATTTGGATTAAAACAAGCGCCAGGAATATTTCAAAGAAAAATG   6534
MAY15-78        GAGTGGAATGTACTTCCATTTGGATTAAAACAAGCGCCAGGAATATTTCAAAGAAAAATG   4112
JUL05-102       GAGTGGAATGTACTTCCATTTGGATTAAAACAAGCGCCAGGAATATTTCAAAGAAAAATG   4101
JUL04-107       GAGTGGAATGTACTTCCATTTGGATTAAAACAAGCGCCAGGAATATTTCAAAGAAAAATG   4112
JUL03-55        GAGTGGAATGTACTTCCATTTGGATTAAAACAAGCGCCAGGAATATTTCAAAGAAAAATG   4210
JUL02-106       GAGTGGAATGTACTTCCATTTGGATTAAAACAAGCGCCAGGAATATTTCAAAGAAAAATG   4101
JUL01-85        GAGTGGAATGTACTTCCATTTGGATTAAAACAAGCGCCAGGAATATTTCAAAGAAAAATG   4315
DEC03-186       GAGTGGAATGTACTTCCATTTGGATTAAAACAAGCGCCAGGAATATTTCAAAGAAAAATG   2559
DEC07-98        GAGTGGAATGTACTTCCATTTGGATTAAAACAAGCGCCAGGAATATTTCAAAGAAAAATG   3078
APR17-291       GAGTGGAATGTACTTCCATTTGGATTAAAACAAGCGCCAGGAATATTTCAAAGAAAAATG   1940
                ************************************************************

DEC02-76        GATGATGCATTAGAATTAAAAAATATAACAGACAAGAGCAAACAATTTGTAAGTCTTTAT   5270
CLR03-38395     GATGATGCATTAGAATTAAAAAATATAACAGACAAGAGCAAACAATTTGTAAGTCTTTAT   4336
CLR01-43699     GATGATGCATTAGAATTAAAAAATATAACAGACAAGAGCAAACAATTTGTAAGTCTTTAT   7007
APR16-68        GATGATGCATTAGAATTAAAAAATATAACAGACAAGAGCAAACAATTTGTAAGTCTTTAT   4364
APR18-62        GATGATGCATTAGAATTAAAAAATATAACAGACAAGAGCAAACAATTTGTAAGTCTTTAT   4406
APR19-43        GATGATGCATTAGAATTAAAAAATATAACAGACAAGAGCAAACAATTTGTAAGTCTTTAT   4531
APR20-70        GATGATGCATTAGAATTAAAAAATATAACAGACAAGAGCAAACAATTTGTAAGTCTTTAT   4251
DEC10-249       GATGATGCATTAGAATTAAAAAATATAACAGACAAGAGCAAACAATTTGTAAGTCTTTAT   2415
DEC08-241       GATGATGCATTAGAATTAAAAAATATAACAGACAAGAGCAAACAATTTGTAAGTCTTTAT   2704
DEC06-81        GATGATGCATTAGAATTAAAAAATATAACAGACAAGAGCAAACAATTTGTAAGTCTTTAT   4416
DEC04-13db      GATGATGCATTAGAATTAAAAAATATAACAGACAAGAGCAAACAATTTGTAAGTCTTTAT   11940
MAY17-11        GATGATGCATTAGAATTAAAAAATATAACAGACAAGAGCAAACAATTTGTAAGTCTTTAT   6594
MAY15-78        GATGATGCATTAGAATTAAAAAATATAACAGACAAGAGCAAACAATTTGTAAGTCTTTAT   4172
JUL05-102       GATGATGCATTAGAATTAAAAAATATAACAGACAAGAGCAAACAATTTGTAAGTCTTTAT   4161
JUL04-107       GATGATGCATTAGAATTAAAAAATATAACAGACAAGAGCAAACAATTTGTAAGTCTTTAT   4172
JUL03-55        GATGATGCATTAGAATTAAAAAATATAACAGACAAGAGCAAACAATTTGTAAGTCTTTAT   4270
JUL02-106       GATGATGCATTAGAATTAAAAAATATAACAGACAAGAGCAAACAATTTGTAAGTCTTTAT   4161
JUL01-85        GATGATGCATTAGAATTAAAAAATATAACAGACAAGAGCAAACAATTTGTAAGTCTTTAT   4375
DEC03-186       GATGATGCATTAGAATTAAAAAATATAACAGACAAGAGCAAACAATTTGTAAGTCTTTAT   2619
DEC07-98        GATGATGCATTAGAATTAAAAAATATAACAGACAAGAGCAAACAATTTGTAAGTCTTTAT   3138
APR17-291       GATGATGCATTAGAATTAAAAAATATAACAGACAAGAGCAAACAATTTGTAAGTCTTTAT   2000
                ************************************************************

DEC02-76        GTAGATGATATTATCGTTTACAGTAACACAAGACAAGAACATGATAATCATCTTTTACAA   5330
CLR03-38395     GTAGATGATATTATCGTTTACAGTAACACAAGACAAGAACATGATAATCATCTTTTACAA   4396
CLR01-43699     GTAGATGATATTATCGTTTACAGTAACACAAGACAAGAACATGATAATCATCTTTTACAA   7067
APR16-68        GTAGATGATATTATCGTTTACAGTAACACAAGACAAGAACATGATAATCATCTTTTACAA   4424
APR18-62        GTAGATGATATTATCGTTTACAGTAACACAAGACAAGAACATGATAATCATCTTTTACAA   4466
APR19-43        GTAGATGATATTATCGTTTACAGTAACACAAGACAAGAACATGATAATCATCTTTTACAA   4591
APR20-70        GTAGATGATATTATCGTTTACAGTAACACAAGACAAGAACATGATAATCATCTTTTACAA   4311
DEC10-249       GTAGATGATATTATCGTTTACAGTAACACAAGACAAGAACATGATAATCATCTTTTACAA   2475
DEC08-241       GTAGATGATATTATCGTTTACAGTAACACAAGACAAGAACATGATAATCATCTTTTACAA   2764
DEC06-81        GTAGATGATATTATCGTTTACAGTAACACAAGACAAGAACATGATAATCATCTTTTACAA   4476
DEC04-13db      GTAGATGATATTATCGTTTACAGTAACACAAGACAAGAACATGATAATCATCTTTTACAA   12000
MAY17-11        GTAGATGATATTATCGTTTACAGTAACACAAGACAAGAACATGATAATCATCTTTTACAA   6654
MAY15-78        GTAGATGATATTATCGTTTACAGTAACACAAGACAAGAACATGATAATCATCTTTTACAA   4232
JUL05-102       GTAGATGATATTATCGTTTACAGTAACACAAGACAAGAACATGATAATCATCTTTTACAA   4221
JUL04-107       GTAGATGATATTATCGTTTACAGTAACACAAGACAAGAACATGATAATCATCTTTTACAA   4232
JUL03-55        GTAGATGATATTATCGTTTACAGTAACACAAGACAAGAACATGATAATCATCTTTTACAA   4330
JUL02-106       GTAGATGATATTATCGTTTACAGTAACACAAGACAAGAACATGATAATCATCTTTTACAA   4221
```

```
JUL01-85         GTAGATGATATTATCGTTTACAGTAACACAAGACAAGAACATGATAATCATCTTTTACAA   4435
DEC03-186        GTAGATGATATTATCGTTTACAGTAACACAAGACAAGAACATGATAATCATCTTTTACAA   2679
DEC07-98         GTAGATGATATTATCGTTTACAGTAACACAAGACAAGAACATGATAATCATCTTTTACAA   3198
APR17-291        GTAGATGATATTATCGTTTACAGTAACACAAGACAAGAACATGATAATCATCTTTTACAA   2060
                 ************************************************************

DEC02-76         ACATTGTTAAGATGTAAGAAAAATGGTATTGTTCTCAGTACCAAAAAGACACAATTATAC   5390
CLR03-38395      ACATTGTTAAGATGTAAGAAAAATGGTATTGTTCTCAGTACCAAAAAGACACAATTATAC   4456
CLR01-43699      ACATTGTTAAGATGTAAGAAAAATGGTATTGTTCTCAGTACCAAAAAGACACAATTATAC   7127
APR16-68         ACATTGTTAAGATGTAAGAAAAATGGTATTGTTCTCAGTACCAAAAAGACACAATTATAC   4484
APR18-62         ACATTGTTAAGATGTAAGAAAAATGGTATTGTTCTCAGTACCAAAAAGACACAATTATAC   4526
APR19-43         ACATTGTTAAGATGTAAGAAAAATGGTATTGTTCTCAGTACCAAAAAGACACAATTATAC   4651
APR20-70         ACATTGTTAAGATGTAAGAAAAATGGTATTGTTCTCAGTACCAAAAAGACACAATTATAC   4371
DEC10-249        ACATTGTTAAGATGTAAGAAAAATGGTATTGTTCTCAGTACCAAAAAGACACAATTATAC   2535
DEC08-241        ACATTGTTAAGATGTAAGAAAAATGGTATTGTTCTCAGTACCAAAAAGACACAATTATAC   2824
DEC06-81         ACATTGTTAAGATGTAAGAAAAATGGTATTGTTCTCAGTACCAAAAAGACACAATTATAC   4536
DEC04-13db       ACATTGTTAAGATGTAAGAAAAATGGTATTGTTCTCAGTACCAAAAAGACACAATTATAC   12060
MAY17-11         ACATTGTTAAGATGTAAGAAAAATGGTATTGTTCTCAGTACCAAAAAGACACAATTATAC   6714
MAY15-78         ACATTGTTAAGATGTAAGAAAAATGGTATTGTTCTCAGTACCAAAAAGACACAATTATAC   4292
JUL05-102        ACATTGTTAAGATGTAAGAAAAATGGTATTGTTCTCAGTACCAAAAAGACACAATTATAC   4281
JUL04-107        ACATTGTTAAGATGTAAGAAAAATGGTATTGTTCTCAGTACCAAAAAGACACAATTATAC   4292
JUL03-55         ACATTGTTAAGATGTAAGAAAAATGGTATTGTTCTCAGTACCAAAAAGACACAATTATAC   4390
JUL02-106        ACATTGTTAAGATGTAAGAAAAATGGTATTGTTCTCAGTACCAAAAAGACACAATTATAC   4281
JUL01-85         ACATTGTTAAGATGTAAGAAAAATGGTATTGTTCTCAGTACCAAAAAGACACAATTATAC   4495
DEC03-186        ACATTGTTAAGATGTAAGAAAAATGGTATTGTTCTCAGTACCAAAAAGACACAATTATAC   2739
DEC07-98         ACATTGTTAAGATGTAAGAAAAATGGTATTGTTCTCAGTACCAAAAAGACACAATTATAC   3258
APR17-291        ACATTGTTAAGATGTAAGAAAAATGGTATTGTTCTCAGTACCAAAAAGACACAATTATAC   2120
                 ************************************************************

DEC02-76         TTAAACAAGATTAATTTTCTAGGACTTGAAATAACTGAAGGAACACATAAATTACAACCA   5450
CLR03-38395      TTAAACAAGATTAATTTTCTAGGACTTGAAATAACTGAAGGAACACATAAATTACAACCA   4516
CLR01-43699      TTAAACAAGATTAATTTTCTAGGACTTGAAATAACTGAAGGAACACATAAATTACAACCA   7187
APR16-68         TTAAACAAGATTAATTTTCTAGGACTTGAAATAACTGAAGGAACACATAAATTACAACCA   4544
APR18-62         TTAAACAAGATTAATTTTCTAGGACTTGAAATAACTGAAGGAACACATAAATTACAACCA   4586
APR19-43         TTAAACAAGATTAATTTTCTAGGACTTGAAATAACTGAAGGAACACATAAATTACAACCA   4711
APR20-70         TTAAACAAGATTAATTTTCTAGGACTTGAAATAACTGAAGGAACACATAAATTACAACCA   4431
DEC10-249        TTAAACAAGATTAATTTTCTAGGACTTGAAATAACTGAAGGAACACATAAATTACAACCA   2595
DEC08-241        TTAAACAAGATTAATTTTCTAGGACTTGAAATAACTGAAGGAACACATAAATTACAACCA   2884
DEC06-81         TTAAACAAGATTAATTTTCTAGGACTTGAAATAACTGAAGGAACACATAAATTACAACCA   4596
DEC04-13db       TTAAACAAGATTAATTTTCTAGGACTTGAAATAACTGAAGGAACACATAAATTACAACCA   12120
MAY17-11         TTAAACAAGATTAATTTTCTAGGACTTGAAATAACTGAAGGAACACATAAATTACAACCA   6774
MAY15-78         TTAAACAAGATTAATTTTCTAGGACTTGAAATAACTGAAGGAACACATAAATTACAACCA   4352
JUL05-102        TTAAACAAGATTAATTTTCTAGGACTTGAAATAACTGAAGGAACACATAAATTACAACCA   4341
JUL04-107        TTAAACAAGATTAATTTTCTAGGACTTGAAATAACTGAAGGAACACATAAATTACAACCA   4352
JUL03-55         TTAAACAAGATTAATTTTCTAGGACTTGAAATAACTGAAGGAACACATAAATTACAACCA   4450
JUL02-106        TTAAACAAGATTAATTTTCTAGGACTTGAAATAACTGAAGGAACACATAAATTACAACCA   4341
JUL01-85         TTAAACAAGATTAATTTTCTAGGACTTGAAATAACTGAAGGAACACATAAATTACAACCA   4555
DEC03-186        TTAAACAAGATTAATTTTCTAGGACTTGAAATAACTGAAGGAACACATAAATTACAACCA   2799
DEC07-98         TTAAACAAGATTAATTTTCTAGGACTTGAAATAACTGAAGGAACACATAAATTACAACCA   3318
APR17-291        TTAAACAAGATTAATTTTCTAGGACTTGAAATAACTGAAGGAACACATAAATTACAACCA   2180
                 ************************************************************

DEC02-76         CACATACTAATAAATTTACATAAATTCCCAGAGAAAATATGTGATAAAAAACAATTACAA   5510
CLR03-38395      CACATACTAATAAATTTACATAAATTCCCAGAGAAAATATGTGATAAAAAACAATTACAA   4576
CLR01-43699      CACATACTAATAAATTTACATAAATTCCCAGAGAAAATATGTGATAAAAAACAATTACAA   7247
APR16-68         CACATACTAATAAATTTACATAAATTCCCAGAGAAAATATGTGATAAAAAACAATTACAA   4604
APR18-62         CACATACTAATAAATTTACATAAATTCCCAGAGAAAATATGTGATAAAAAACAATTACAA   4646
APR19-43         CACATACTAATAAATTTACATAAATTCCCAGAGAAAATATGTGATAAAAAACAATTACAA   4771
APR20-70         CACATACTAATAAATTTACATAAATTCCCAGAGAAAATATGTGATAAAAAACAATTACAA   4491
DEC10-249        CACATACTAATAAATTTACATAAATTCCCAGAGAAAATATGTGATAAAAAACAATTACAA   2655
DEC08-241        CACATACTAATAAATTTACATAAATTCCCAGAGAAAATATGTGATAAAAAACAATTACAA   2944
DEC06-81         CACATACTAATAAATTTACATAAATTCCCAGAGAAAATATGTGATAAAAAACAATTACAA   4656
DEC04-13db       CACATACTAATAAATTTACATAAATTCCCAGAGAAAATATGTGATAAAAAACAATTACAA   12180
```

```
MAY17-11        CACATACTAATAAATTTACATAAATTCCCAGAGAAAATATGTGATAAAAAACAATTACAA   6834
MAY15-78        CACATACTAATAAATTTACATAAATTCCCAGAGAAAATATGTGATAAAAAACAATTACAA   4412
JUL05-102       CACATACTAATAAATTTACATAAATTCCCAGAGAAAATATGTGATAAAAAACAATTACAA   4401
JUL04-107       CACATACTAATAAATTTACATAAATTCCCAGAGAAAATATGTGATAAAAAACAATTACAA   4412
JUL03-55        CACATACTAATAAATTTACATAAATTCCCAGAGAAAATATGTGATAAAAAACAATTACAA   4510
JUL02-106       CACATACTAATAAATTTACATAAATTCCCAGAGAAAATATGTGATAAAAAACAATTACAA   4401
JUL01-85        CACATACTAATAAATTTACATAAATTCCCAGAGAAAATATGTGATAAAAAACAATTACAA   4615
DEC03-186       CACATACTAATAAATTTACATAAATTCCCAGAGAAAATATGTGATAAAAAACAATTACAA   2859
DEC07-98        CACATACTAATAAATTTACATAAATTCCCAGAGAAAATATGTGATAAAAAACAATTACAA   3378
APR17-291       CACATACTAATAAATTTACATAAATTCCCAGAGAAAATATGTGATAAAAAACAATTACAA   2240
                ************************************************************

DEC02-76        AGATTTTTGGGTTGCTTGACGTACGCAGAGTGCTATATAGCTAAGTTGGCTGAAATCAGG   5570
CLR03-38395     AGATTTTTGGGTTGCTTGACGTACGCAGAGTGCTATATAGCTAAGTTGGCTGAAATCAGG   4636
CLR01-43699     AGATTTTTGGGTTGCTTGACGTACGCAGAGTGCTATATAGCTAAGTTGGCTGAAATCAGG   7307
APR16-68        AGATTTTTGGGTTGCTTGACGTACGCAGAGTGCTATATAGCTAAGTTGGCTGAAATCAGG   4664
APR18-62        AGATTTTTGGGTTGCTTGACGTACGCAGAGTGCTATATAGCTAAGTTGGCTGAAATCAGG   4706
APR19-43        AGATTTTTGGGTTGCTTGACGTACGCAGAGTGCTATATAGCTAAGTTGGCTGAAATCAGG   4831
APR20-70        AGATTTTTGGGTTGCTTGACGTACGCAGAGTGCTATATAGCTAAGTTGGCTGAAATCAGG   4551
DEC10-249       AGATTTTTGGGTTGCTTGACGTACGCAGAGTGCTATATAGCTAAGTTGGCTGAAATCAGG   2715
DEC08-241       AGATTTTTGGGTTGCTTGACGTACGCAGAGTGCTATATAGCTAAGTTGGCTGAAATCAGG   3004
DEC06-81        AGATTTTTGGGTTGCTTGACGTACGCAGAGTGCTATATAGCTAAGTTGGCTGAAATCAGG   4716
DEC04-13db      AGATTTTTGGGTTGCTTGACGTACGCAGAGTGCTATATAGCTAAGTTGGCTGAAATCAGG   12240
MAY17-11        AGATTTTTGGGTTGCTTGACGTACGCAGAGTGCTATATAGCTAAGTTGGCTGAAATCAGG   6894
MAY15-78        AGATTTTTGGGTTGCTTGACGTACGCAGAGTGCTATATAGCTAAGTTGGCTGAAATCAGG   4472
JUL05-102       AGATTTTTGGGTTGCTTGACGTACGCAGAGTGCTATATAGCTAAGTTGGCTGAAATCAGG   4461
JUL04-107       AGATTTTTGGGTTGCTTGACGTACGCAGAGTGCTATATAGCTAAGTTGGCTGAAATCAGG   4472
JUL03-55        AGATTTTTGGGTTGCTTGACGTACGCAGAGTGCTATATAGCTAAGTTGGCTGAAATCAGG   4570
JUL02-106       AGATTTTTGGGTTGCTTGACGTACGCAGAGTGCTATATAGCTAAGTTGGCTGAAATCAGG   4461
JUL01-85        AGATTTTTGGGTTGCTTGACGTACGCAGAGTGCTATATAGCTAAGTTGGCTGAAATCAGG   4675
DEC03-186       AGATTTTTGGGTTGCTTGACGTACGCAGAGTGCTATATAGCTAAGTTGGCTGAAATCAGG   2919
DEC07-98        AGATTTTTGGGTTGCTTGACGTACGCAGAGTGCTATATAGCTAAGTTGGCTGAAATCAGG   3438
APR17-291       AGATTTTTGGGTTGCTTGACGTACGCAGAGTGCTATATAGCTAAGTTGGCTGAAATCAGG   2300
                ************************************************************

DEC02-76        AAGCCACTCCAAAAGAAACTCAAAAAAGATTATGTATGGCAATGGACCCAAGAGGATACC   5630
CLR03-38395     AAGCCACTCCAAAAGAAACTCAAAAAAGATTATGTATGGCAATGGACCCAAGAGGATACC   4696
CLR01-43699     AAGCCACTCCAAAAGAAACTCAAAAAAGATTATGTATGGCAATGGACCCAAGAGGATACC   7367
APR16-68        AAGCCACTCCAAAAGAAACTCAAAAAAGATTATGTATGGCAATGGACCCAAGAGGATACC   4724
APR18-62        AAGCCACTCCAAAAGAAACTCAAAAAAGATTATGTATGGCAATGGACCCAAGAGGATACC   4766
APR19-43        AAGCCACTCCAAAAGAAACTCAAAAAAGATTATGTATGGCAATGGACCCAAGAGGATACC   4891
APR20-70        AAGCCACTCCAAAAGAAACTCAAAAAAGATTATGTATGGCAATGGACCCAAGAGGATACC   4611
DEC10-249       AAGCCACTCCAAAAGAAACTCAAAAAAGATTATGTATGGCAATGGACCCAAGAGGATACC   2775
DEC08-241       AAGCCACTCCAAAAGAAACTCAAAAAAGATTATGTATGGCAATGGACCCAAGAGGATACC   3064
DEC06-81        AAGCCACTCCAAAAGAAACTCAAAAAAGATTATGTATGGCAATGGACCCAAGAGGATACC   4776
DEC04-13db      AAGCCACTCCAAAAGAAACTCAAAAAAGATTATGTATGGCAATGGACCCAAGAGGATACC   12300
MAY17-11        AAGCCACTCCAAAAGAAACTCAAAAAAGATTATGTATGGCAATGGACCCAAGAGGATACC   6954
MAY15-78        AAGCCACTCCAAAAGAAACTCAAAAAAGATTATGTATGGCAATGGACCCAAGAGGATACC   4532
JUL05-102       AAGCCACTCCAAAAGAAACTCAAAAAAGATTATGTATGGCAATGGACCCAAGAGGATACC   4521
JUL04-107       AAGCCACTCCAAAAGAAACTCAAAAAAGATTATGTATGGCAATGGACCCAAGAGGATACC   4532
JUL03-55        AAGCCACTCCAAAAGAAACTCAAAAAAGATTATGTATGGCAATGGACCCAAGAGGATACC   4630
JUL02-106       AAGCCACTCCAAAAGAAACTCAAAAAAGATTATGTATGGCAATGGACCCAAGAGGATACC   4521
JUL01-85        AAGCCACTCCAAAAGAAACTCAAAAAAGATTATGTATGGCAATGGACCCAAGAGGATACC   4735
DEC03-186       AAGCCACTCCAAAAGAAACTCAAAAAAGATTATGTATGGCAATGGACCCAAGAGGATACC   2979
DEC07-98        AAGCCACTCCAAAAGAAACTCAAAAAAGATTATGTATGGCAATGGACCCAAGAGGATACC   3498
APR17-291       AAGCCACTCCAAAAGAAACTCAAAAAAGATTATGTATGGCAATGGACCCAAGAGGATACC   2360
                ************************************************************

DEC02-76        GCCTACATAAGGAAAATAAAAAACAAATTGAAAGATTTTCCAACGTTATACCAACCACAG   5690
CLR03-38395     GCCTACATAAGGAAAATAAAAAACAAATTGAAAGATTTTCCAACGTTATACCAACCACAG   4756
CLR01-43699     GCCTACATAAGGAAAATAAAAAACAAATTGAAAGATTTTCCAACGTTATACCAACCACAG   7427
APR16-68        GCCTACATAAGGAAAATAAAAAACAAATTGAAAGATTTTCCAACGTTATACCAACCACAG   4784
APR18-62        GCCTACATAAGGAAAATAAAAAACAAATTGAAAGATTTTCCAACGTTATACCAACCACAG   4826
```

```
APR19-43        GCCTACATAAGGAAAATAAAAAACAAATTGAAAGATTTTCCAACGTTATACCAACCACAG   4951
APR20-70        GCCTACATAAGGAAAATAAAAAACAAATTGAAAGATTTTCCAACGTTATACCAACCACAG   4671
DEC10-249       GCCTACATAAGGAAAATAAAAAACAAATTGAAAGATTTTCCAACGTTATACCAACCACAG   2835
DEC08-241       GCCTACATAAGGAAAATAAAAAACAAATTGAAAGATTTTCCAACGTTATACCAACCACAG   3124
DEC06-81        GCCTACATAAGGAAAATAAAAAACAAATTGAAAGATTTTCCAACGTTATACCAACCACAG   4836
DEC04-13db      GCCTACATAAGGAAAATAAAAAACAAATTGAAAGATTTTCCAACGTTATACCAACCACAG   12360
MAY17-11        GCCTACATAAGGAAAATAAAAAACAAATTGAAAGATTTTCCAACGTTATACCAACCACAG   7014
MAY15-78        GCCTACATAAGGAAAATAAAAAACAAATTGAAAGATTTTCCAACGTTATACCAACCACAG   4592
JUL05-102       GCCTACATAAGGAAAATAAAAAACAAATTGAAAGATTTTCCAACGTTATACCAACCACAG   4581
JUL04-107       GCCTACATAAGGAAAATAAAAAACAAATTGAAAGATTTTCCAACGTTATACCAACCACAG   4592
JUL03-55        GCCTACATAAGGAAAATAAAAAACAAATTGAAAGATTTTCCAACGTTATACCAACCACAG   4690
JUL02-106       GCCTACATAAGGAAAATAAAAAACAAATTGAAAGATTTTCCAACGTTATACCAACCACAG   4581
JUL01-85        GCCTACATAAGGAAAATAAAAAACAAATTGAAAGATTTTCCAACGTTATACCAACCACAG   4795
DEC03-186       GCCTACATAAGGAAAATAAAAAACAAATTGAAAGATTTTCCAACGTTATACCAACCACAG   3039
DEC07-98        GCCTACATAAGGAAAATAAAAAACAAATTGAAAGATTTTCCAACGTTATACCAACCACAG   3558
APR17-291       GCCTACATAAGGAAAATAAAAAACAAATTGAAAGATTTTCCAACGTTATACCAACCACAG   2420
                ************************************************************

DEC02-76        GATGAGGATTTAATGATACTTGAAACAGATGCTAGCCAAGAATATTGGTCTGGTGTTTTA   5750
CLR03-38395     GATGAGGATTTAATGATACTTGAAACAGATGCTAGCCAAGAATATTGGTCTGGTGTTTTA   4816
CLR01-43699     GATGAGGATTTAATGATACTTGAAACAGATGCTAGCCAAGAATATTGGTCTGGTGTTTTA   7487
APR16-68        GATGAGGATTTAATGATACTTGAAACAGATGCTAGCCAAGAATATTGGTCTGGTGTTTTA   4844
APR18-62        GATGAGGATTTAATGATACTTGAAACAGATGCTAGCCAAGAATATTGGTCTGGTGTTTTA   4886
APR19-43        GATGAGGATTTAATGATACTTGAAACAGATGCTAGCCAAGAATATTGGTCTGGTGTTTTA   5011
APR20-70        GATGAGGATTTAATGATACTTGAAACAGATGCTAGCCAAGAATATTGGTCTGGTGTTTTA   4731
DEC10-249       GATGAGGATTTAATGATACTTGAAACAGATGCTAGCCAAGAATATTGGTCTGGTGTTTTA   2895
DEC08-241       GATGAGGATTTAATGATACTTGAAACAGATGCTAGCCAAGAATATTGGTCTGGTGTTTTA   3184
DEC06-81        GATGAGGATTTAATGATACTTGAAACAGATGCTAGCCAAGAATATTGGTCTGGTGTTTTA   4896
DEC04-13db      GATGAGGATTTAATGATACTTGAAACAGATGCTAGCCAAGAATATTGGTCTGGTGTTTTA   12420
MAY17-11        GATGAGGATTTAATGATACTTGAAACAGATGCTAGCCAAGAATATTGGTCTGGTGTTTTA   7074
MAY15-78        GATGAGGATTTAATGATACTTGAAACAGATGCTAGCCAAGAATATTGGTCTGGTGTTTTA   4652
JUL05-102       GATGAGGATTTAATGATACTTGAAACAGATGCTAGCCAAGAATATTGGTCTGGTGTTTTA   4641
JUL04-107       GATGAGGATTTAATGATACTTGAAACAGATGCTAGCCAAGAATATTGGTCTGGTGTTTTA   4652
JUL03-55        GATGAGGATTTAATGATACTTGAAACAGATGCTAGCCAAGAATATTGGTCTGGTGTTTTA   4750
JUL02-106       GATGAGGATTTAATGATACTTGAAACAGATGCTAGCCAAGAATATTGGTCTGGTGTTTTA   4641
JUL01-85        GATGAGGATTTAATGATACTTGAAACAGATGCTAGCCAAGAATATTGGTCTGGTGTTTTA   4855
DEC03-186       GATGAGGATTTAATGATACTTGAAACAGATGCTAGCCAAGAATATTGGTCTGGTGTTTTA   3099
DEC07-98        GATGAGGATTTAATGATACTTGAAACAGATGCTAGCCAAGAATATTGGTCTGGTGTTTTA   3618
APR17-291       GATGAGGATTTAATGATACTTGAAACAGATGCTAGCCAAGAATATTGGTCTGGTGTTTTA   2480
                ************************************************************

DEC02-76        AAAGCTAAATCATTAAAAAAATGATAATCAGGAAATGCTTTGTAGATATACCTCAGGTACA   5810
CLR03-38395     AAAGCTAAATCATTAAAAAAATGATAATCAGGAAATGCTTTGTAGATATACCTCAGGTACA   4876
CLR01-43699     AAAGCTAAATCATTAAAAAAATGATAATCAGGAAATGCTTTGTAGATATACCTCAGGTACA   7547
APR16-68        AAAGCTAAATCATTAAAAAAATGATAATCAGGAAATGCTTTGTAGATATACCTCAGGTACA   4904
APR18-62        AAAGCTAAATCATTAAAAAAATGATAATCAGGAAATGCTTTGTAGATATACCTCAGGTACA   4946
APR19-43        AAAGCTAAATCATTAAAAAAATGATAATCAGGAAATGCTTTGTAGATATACCTCAGGTACA   5071
APR20-70        AAAGCTAAATCATTAAAAAAATGATAATCAGGAAATGCTTTGTAGATATACCTCAGGTACA   4791
DEC10-249       AAAGCTAAATCATTAAAAAAATGATAATCAGGAAATGCTTTGTAGATATACCTCAGGTACA   2955
DEC08-241       AAAGCTAAATCATTAAAAAAATGATAATCAGGAAATGCTTTGTAGATATACCTCAGGTACA   3244
DEC06-81        AAAGCTAAATCATTAAAAAAATGATAATCAGGAAATGCTTTGTAGATATACCTCAGGTACA   4956
DEC04-13db      AAAGCTAAATCATTAAAAAAATGATAATCAGGAAATGCTTTGTAGATATACCTCAGGTACA   12480
MAY17-11        AAAGCTAAATCATTAAAAAAATGATAATCAGGAAATGCTTTGTAGATATACCTCAGGTACA   7134
MAY15-78        AAAGCTAAATCATTAAAAAAATGATAATCAGGAAATGCTTTGTAGATATACCTCAGGTACA   4712
JUL05-102       AAAGCTAAATCATTAAAAAAATGATAATCAGGAAATGCTTTGTAGATATACCTCAGGTACA   4701
JUL04-107       AAAGCTAAATCATTAAAAAAATGATAATCAGGAAATGCTTTGTAGATATACCTCAGGTACA   4712
JUL03-55        AAAGCTAAATCATTAAAAAAATGATAATCAGGAAATGCTTTGTAGATATACCTCAGGTACA   4810
JUL02-106       AAAGCTAAATCATTAAAAAAATGATAATCAGGAAATGCTTTGTAGATATACCTCAGGTACA   4701
JUL01-85        AAAGCTAAATCATTAAAAAAATGATAATCAGGAAATGCTTTGTAGATATACCTCAGGTACA   4915
DEC03-186       AAAGCTAAATCATTAAAAAAATGATAATCAGGAAATGCTTTGTAGATATACCTCAGGTACA   3159
DEC07-98        AAAGCTAAATCATTAAAAAAATGATAATCAGGAAATGCTTTGTAGATATACCTCAGGTACA   3678
APR17-291       AAAGCTAAATCATTAAAAAAATGATAATCAGGAAATGCTTTGTAGATATACCTCAGGTACA   2540
                ************************************************************
```

Figure S4

```
DEC02-76        TTTACAGGAGCTGAACTAAATTATCACAGTAATGAAAAGGAATGGCTCGCTGTAAAGAAA   5870
CLR03-38395     TTTACAGGAGCTGAACTAAATTATCACAGTAATGAAAAGGAATGGCTCGCTGTAAAGAAA   4936
CLR01-43699     TTTACAGGAGCTGAACTAAATTATCACAGTAATGAAAAGGAATGGCTCGCTGTAAAGAAA   7607
APR16-68        TTTACAGGAGCTGAACTAAATTATCACAGTAATGAAAAGGAATGGCTCGCTGTAAAGAAA   4964
APR18-62        TTTACAGGAGCTGAACTAAATTATCACAGTAATGAAAAGGAATGGCTCGCTGTAAAGAAA   5006
APR19-43        TTTACAGGAGCTGAACTAAATTATCACAGTAATGAAAAGGAATGGCTCGCTGTAAAGAAA   5131
APR20-70        TTTACAGGAGCTGAACTAAATTATCACAGTAATGAAAAGGAATGGCTCGCTGTAAAGAAA   4851
DEC10-249       TTTACAGGAGCTGAACTAAATTATCACAGTAATGAAAAGGAATGGCTCGCTGTAAAGAAA   3015
DEC08-241       TTTACAGGAGCTGAACTAAATTATCACAGTAATGAAAAGGAATGGCTCGCTGTAAAGAAA   3304
DEC06-81        TTTACAGGAGCTGAACTAAATTATCACAGTAATGAAAAGGAATGGCTCGCTGTAAAGAAA   5016
DEC04-13db      TTTACAGGAGCTGAACTAAATTATCACAGTAATGAAAAGGAATGGCTCGCTGTAAAGAAA   12540
MAY17-11        TTTACAGGAGCTGAACTAAATTATCACAGTAATGAAAAGGAATGGCTCGCTGTAAAGAAA   7194
MAY15-78        TTTACAGGAGCTGAACTAAATTATCACAGTAATGAAAAGGAATGGCTCGCTGTAAAGAAA   4772
JUL05-102       TTTACAGGAGCTGAACTAAATTATCACAGTAATGAAAAGGAATGGCTCGCTGTAAAGAAA   4761
JUL04-107       TTTACAGGAGCTGAACTAAATTATCACAGTAATGAAAAGGAATGGCTCGCTGTAAAGAAA   4772
JUL03-55        TTTACAGGAGCTGAACTAAATTATCACAGTAATGAAAAGGAATGGCTCGCTGTAAAGAAA   4870
JUL02-106       TTTACAGGAGCTGAACTAAATTATCACAGTAATGAAAAGGAATGGCTCGCTGTAAAGAAA   4761
JUL01-85        TTTACAGGAGCTGAACTAAATTATCACAGTAATGAAAAGGAATGGCTCGCTGTAAAGAAA   4975
DEC03-186       TTTACAGGAGCTGAACTAAATTATCACAGTAATGAAAAGGAATGGCTCGCTGTAAAGAAA   3219
DEC07-98        TTTACAGGAGCTGAACTAAATTATCACAGTAATGAAAAGGAATGGCTCGCTGTAAAGAAA   3738
APR17-291       TTTACAGGAGCTGAACTAAATTATCACAGTAATGAAAAGGAATGGCTCGCTGTAAAGAAA   2600
                ************************************************************

DEC02-76        GCCATAGGAAAATTCAGGATTTACTACCTAAGGAGTTTGTTGTCCGAACGGATAATAAAC   5930
CLR03-38395     GCCATAGGAAAATTCAGGATTTACTACCTAAGGAGTTTGTTGTCCGAACGGATAATAAAC   4996
CLR01-43699     GCCATAGGAAAATTCAGGATTTACTACCTAAGGAGTTTGTTGTCCGAACGGATAATAAAC   7667
APR16-68        GCCATAGGAAAATTCAGGATTTACTACCTAAGGAGTTTGTTGTCCGAACGGATAATAAAC   5024
APR18-62        GCCATAGGAAAATTCAGGATTTACTACCTAAGGAGTTTGTTGTCCGAACGGATAATAAAC   5066
APR19-43        GCCATAGGAAAATTCAGGATTTACTACCTAAGGAGTTTGTTGTCCGAACGGATAATAAAC   5191
APR20-70        GCCATAGGAAAATTCAGGATTTACTACCTAAGGAGTTTGTTGTCCGAACGGATAATAAAC   4911
DEC10-249       GCCATAGGAAAATTCAGGATTTACTACCTAAGGAGTTTGTTGTCCGAACGGATAATAAAC   3075
DEC08-241       GCCATAGGAAAATTCAGGATTTACTACCTAAGGAGTTTGTTGTCCGAACGGATAATAAAC   3364
DEC06-81        GCCATAGGAAAATTCAGGATTTACTACCTAAGGAGTTTGTTGTCCGAACGGATAATAAAC   5076
DEC04-13db      GCCATAGGAAAATTCAGGATTTACTACCTAAGGAGTTTGTTGTCCGAACGGATAATAAAC   12600
MAY17-11        GCCATAGGAAAATTCAGGATTTACTACCTAAGGAGTTTGTTGTCCGAACGGATAATAAAC   7254
MAY15-78        GCCATAGGAAAATTCAGGATTTACTACCTAAGGAGTTTGTTGTCCGAACGGATAATAAAC   4832
JUL05-102       GCCATAGGAAAATTCAGGATTTACTACCTAAGGAGTTTGTTGTCCGAACGGATAATAAAC   4821
JUL04-107       GCCATAGGAAAATTCAGGATTTACTACCTAAGGAGTTTGTTGTCCGAACGGATAATAAAC   4832
JUL03-55        GCCATAGGAAAATTCAGGATTTACTACCTAAGGAGTTTGTTGTCCGAACGGATAATAAAC   4930
JUL02-106       GCCATAGGAAAATTCAGGATTTACTACCTAAGGAGTTTGTTGTCCGAACGGATAATAAAC   4821
JUL01-85        GCCATAGGAAAATTCAGGATTTACTACCTAAGGAGTTTGTTGTCCGAACGGATAATAAAC   5035
DEC03-186       GCCATAGGAAAATTCAGGATTTACTACCTAAGGAGTTTGTTGTCCGAACGGATAATAAAC   3279
DEC07-98        GCCATAGGAAAATTCAGGATTTACTACCTAAGGAGTTTGTTGTCCGAACGGATAATAAAC   3798
APR17-291       GCCATAGGAAAATTCAGGATTTACTACCTAAGGAGTTTGTTGTCCGAACGGATAATAAAC   2660
                ************************************************************

DEC02-76        AATTTGGACCTTTTATAAGGAACAATATTACAGGAGACTATAAACAAGGACGATTATTAA   5990
CLR03-38395     AATTTGGACCTTTTATAAGGAACAATATTACAGGAGACTATAAACAAGGACGATTATTAA   5056
CLR01-43699     AATTTGGACCTTTTATAAGGAACAATATTACAGGAGACTATAAACAAGGACGATTATTAA   7727
APR16-68        AATTTGGACCTTTTATAAGGAACAATATTACAGGAGACTATAAACAAGGACGATTATTAA   5084
APR18-62        AATTTGGACCTTTTATAAGGAACAATATTACAGGAGACTATAAACAAGGACGATTATTAA   5126
APR19-43        AATTTGGACCTTTTATAAGGAACAATATTACAGGAGACTATAAACAAGGACGATTATTAA   5251
APR20-70        AATTTGGACCTTTTATAAGGAACAATATTACAGGAGACTATAAACAAGGACGATTATTAA   4971
DEC10-249       AATTTGGACCTTTTATAAGGAACAATATTACAGGAGACTATAAACAAGGACGATTATTAA   3135
DEC08-241       AATTTGGACCTTTTATAAGGAACAATATTACAGGAGACTATAAACAAGGACGATTATTAA   3424
DEC06-81        AATTTGGACCTTTTATAAGGAACAATATTACAGGAGACTATAAACAAGGACGATTATTAA   5136
DEC04-13db      AATTTGGACCTTTTATAAGGAACAATATTACAGGAGACTATAAACAAGGACGATTATTAA   12660
MAY17-11        AATTTGGACCTTTTATAAGGAACAATATTACAGGAGACTATAAACAAGGACGATTATTAA   7314
MAY15-78        AATTTGGACCTTTTATAAGGAACAATATTACAGGAGACTATAAACAAGGACGATTATTAA   4892
JUL05-102       AATTTGGACCTTTTATAAGGAACAATATTACAGGAGACTATAAACAAGGACGATTATTAA   4881
JUL04-107       AATTTGGACCTTTTATAAGGAACAATATTACAGGAGACTATAAACAAGGACGATTATTAA   4892
JUL03-55        AATTTGGACCTTTTATAAGGAACAATATTACAGGAGACTATAAACAAGGACGATTATTAA   4990
```

```
JUL02-106        AATTTGGACCTTTTATAAGGAACAATATTACAGGAGACTATAAACAAGGACGATTATTAA   4881
JUL01-85         AATTTGGACCTTTTATAAGGAACAATATTACAGGAGACTATAAACAAGGACGATTATTAA   5095
DEC03-186        AATTTGGACCTTTTATAAGGAACAATATTACAGGAGACTATAAACAAGGACGATTATTAA   3339
DEC07-98         AATTTGGACCTTTTATAAGGAACAATATTACAGGAGACTATAAACAAGGACGATTATTAA   3858
APR17-291        AATTTGGACCTTTTATAAGGAACAATATTACAGGAGACTATAAACAAGGACGATTATTAA   2720
                 ************************************************************

DEC02-76         GATGGCAACAGTGGTTTAATTACTACAAGTTCACCATCGAACACATTCGAGGAGAAGAGA   6050
CLR03-38395      GATGGCAACAGTGGTTTAATTACTACAAGTTCACCATCGAACACATTCGAGGAGAAGAGA   5116
CLR01-43699      GATGGCAACAGTGGTTTAATTACTACAAGTTCACCATCGAACACATTCGAGGAGAAGAGA   7787
APR16-68         GATGGCAACAGTGGTTTAATTACTACAAGTTCACCATCGAACACATTCGAGGAGAAGAGA   5144
APR18-62         GATGGCAACAGTGGTTTAATTACTACAAGTTCACCATCGAACACATTCGAGGAGAAGAGA   5186
APR19-43         GATGGCAACAGTGGTTTAATTACTACAAGTTCACCATCGAACACATTCGAGGAGAAGAGA   5311
APR20-70         GATGGCAACAGTGGTTTAATTACTACAAGTTCACCATCGAACACATTCGAGGAGAAGAGA   5031
DEC10-249        GATGGCAACAGTGGTTTAATTACTACAAGTTCACCATCGAACACATTCGAGGAGAAGAGA   3195
DEC08-241        GATGGCAACAGTGGTTTAATTACTACAAGTTCACCATCGAACACATTCGAGGAGAAGAGA   3484
DEC06-81         GATGGCAACAGTGGTTTAATTACTACAAGTTCACCATCGAACACATTCGAGGAGAAGAGA   5196
DEC04-13db       GATGGCAACAGTGGTTTAATTACTACAAGTTCACCATCGAACACATTCGAGGAGAAGAGA   12720
MAY17-11         GATGGCAACAGTGGTTTAATTACTACAAGTTCACCATCGAACACATTCGAGGAGAAGAGA   7374
MAY15-78         GATGGCAACAGTGGTTTAATTACTACAAGTTCACCATCGAACACATTCGAGGAGAAGAGA   4952
JUL05-102        GATGGCAACAGTGGTTTAATTACTACAAGTTCACCATCGAACACATTCGAGGAGAAGAGA   4941
JUL04-107        GATGGCAACAGTGGTTTAATTACTACAAGTTCACCATCGAACACATTCGAGGAGAAGAGA   4952
JUL03-55         GATGGCAACAGTGGTTTAATTACTACAAGTTCACCATCGAACACATTCGAGGAGAAGAGA   5050
JUL02-106        GATGGCAACAGTGGTTTAATTACTACAAGTTCACCATCGAACACATTCGAGGAGAAGAGA   4941
JUL01-85         GATGGCAACAGTGGTTTAATTACTACAAGTTCACCATCGAACACATTCGAGGAGAAGAGA   5155
DEC03-186        GATGGCAACAGTGGTTTAATTACTACAAGTTCACCATCGAACACATTCGAGGAGAAGAGA   3399
DEC07-98         GATGGCAACAGTGGTTTAATTACTACAAGTTCACCATCGAACACATTCGAGGAGAAGAGA   3918
APR17-291        GATGGCAACAGTGGTTTAATTACTACAAGTTCACCATCGAACACATTCGAGGAGAAGAGA   2780
                 ************************************************************

DEC02-76         ATTATCTCGCAGATCTATTGACTCGAGAGTTCGCTTCATAA-------------------   6091
CLR03-38395      ATTATCTCGCAGATCTATTGACTCGAGAGTTCGCTTCATAATAAAATTTAATCAACTAAA   5176
CLR01-43699      ATTATCTCGCAGATCTATTGACTCGAGAGTTCGCTTCATAATAAAATTTAATCAACTAAA   7847
APR16-68         ATTATCTCGCAGATCTATTGACTCGAGAGTTCGCTTCATAATAAAATTTAATCAACTAAA   5204
APR18-62         ATTATCTCGCAGATCTATTGACTCGAGAGTTCGCTTCATAATAAAATTTAATCAACTAAA   5246
APR19-43         ATTATCTCGCAGATCTATTGACTCGAGAGTTCGCTTCATAATAAAATTTAATCAACTAAA   5371
APR20-70         ATTATCTCGCAGATCTATTGACTCGAGAGTTCGCTTCATAATAAAATTTAATCAACTAAA   5091
DEC10-249        ATTATCTCGCAGATCTATTGACTCGAGAGTTCGCTTCATAATAAAATTTAATCAACTAAA   3255
DEC08-241        ATTATCTCGCAGATCTATTGACTCGAGAGTTCGCTTCATAATAAAATTTAATCAACTAAA   3544
DEC06-81         ATTATCTCGCAGATCTATTGACTCGAGAGTTCGCTTCATAATAAAATTTAATCAACTAAA   5256
DEC04-13db       ATTATCTCGCAGATCTATTGACTCGAGAGTTCGCTTCATAATAAAATTTAATCAACTAAA   12780
MAY17-11         ATTATCTCGCAGATCTATTGACTCGAGAGTTCGCTTCATAATAAAATTTAATCAACTAAA   7434
MAY15-78         ATTATCTCGCAGATCTATTGACTCGAGAGTTCGCTTCATAATAAAATTTAATCAACTAAA   5012
JUL05-102        ATTATCTCGCAGATCTATTGACTCGAGAGTTCGCTTCATAATAAAATTTAATCAACTAAA   5001
JUL04-107        ATTATCTCGCAGATCTATTGACTCGAGAGTTCGCTTCATAATAAAATTTAATCAACTAAA   5012
JUL03-55         ATTATCTCGCAGATCTATTGACTCGAGAGTTCGCTTCATAATAAAATTTAATCAACTAAA   5110
JUL02-106        ATTATCTCGCAGATCTATTGACTCGAGAGTTCGCTTCATAATAAAATTTAATCAACTAAA   5001
JUL01-85         ATTATCTCGCAGATCTATTGACTCGAGAGTTCGCTTCATAATAAAATTTAATCAACTAAA   5215
DEC03-186        ATTATCTCGCAGATCTATTGACTCGAGAGTTCGCTTCATAATAAAATTTAATCAACTAAA   3459
DEC07-98         ATTATCTCGCAGATCTATTGACTCGAGAGTTCGCTTCATAATAAAATTTAATCAACTAAA   3978
APR17-291        ATTATCTCGCAGATCTATTGACTCGAGAGTTCGCTTCATAATAAAATTTAATCAACTAAA   2840
                 *****************************************

DEC02-76         ------------------------------------------------------------   6091
CLR03-38395      ATCTGTAAAGATAAAAATTGATTAAAAATTGATAAAATACGAAGGAACCCAAGGAATCGG   5236
CLR01-43699      ATCTGTAAAGATAAAAATTGATTAAAAATTGATAAAATACGAAGGAACCCAAGGAATCGG   7907
APR16-68         ATCTGTAAAGATAAAAATTGATTAAAAATTGATAAAATACGAAGGAACCCAAGGAATCGG   5264
APR18-62         ATCTGTAAAGATAAAAATTGATTAAAAATTGATAAAATACGAAGGAACCCAAGGAATCGG   5306
APR19-43         ATCTGTAAAGATAAAAATTGATTAAAAATTGATAAAATACGAAGGAACCCAAGGAATCGG   5431
APR20-70         ATCTGTAAAGATAAAAATTGATTAAAAATTGATAAAATACGAAGGAACCCAAGGAATCGG   5151
DEC10-249        ATCTGTAAAGATAAAAATTGATTAAAAATTGATAAAATACGAAGGAACCCAAGGAATCGG   3315
DEC08-241        ATCTGTAAAGATAAAAATTGATTAAAAATTGATAAAATACGAAGGAACCCAAGGAATCGG   3604
DEC06-81         ATCTGTAAAGATAAAAATTGATTAAAAATTGATAAAATACGAAGGAACCCAAGGAATCGG   5316
```

Figure S4

```
DEC04-13db      ATCTGTAAAGATAAAAATTGATTAAAAATTGATAAAATACGAAGGAACCCAAGGAATCGG   12840
MAY17-11        ATCTGTAAAGATAAAAATTGATTAAAAATTGATAAAATACGAAGGAACCCAAGGAATCGG   7494
MAY15-78        ATCTGTAAAGATAAAAATTGATTAAAAATTGATAAAATACGAAGGAACCCAAGGAATCGG   5072
JUL05-102       ATCTGTAAAGATAAAAATTGATTAAAAATTGATAAAATACGAAGGAACCCAAGGAATCGG   5061
JUL04-107       ATCTGTAAAGATAAAAATTGATTAAAAATTGATAAAATACGAAGGAACCCAAGGAATCGG   5072
JUL03-55        ATCTGTAAAGATAAAAATTGATTAAAAATTGATAAAATACGAAGGAACCCAAGGAATCGG   5170
JUL02-106       ATCTGTAAAGATAAAAATTGATTAAAAATTGATAAAATACGAAGGAACCCAAGGAATCGG   5061
JUL01-85        ATCTGTAAAGATAAAAATTGATTAAAAATTGATAAAATACGAAGGAACCCAAGGAATCGG   5275
DEC03-186       ATCTGTAAAGATAAAAATTGATTAAAAATTGATAAAATACGAAGGAACCCAAGGAATCGG   3519
DEC07-98        ATCTGTAAAGATAAAAATTGATTAAAAATTGATAAAATACGAAGGAACCCAAGGAATCGG   4038
APR17-291       ATCTGTAAAGATAAAAATTGATTAAAAATTGATAAAATACGAAGGAACCCAAGGAATCGG   2900


DEC02-76        ------------------------------------------------------------   6091
CLR03-38395     AACCCAAGGAGCTAAAGGAGCAGCAAGGAGTCCAAGCAGCAAAGGAAAGGAGAATTTTTA   5296
CLR01-43699     AACCCAAGGAGCTAAAGGAGCAGCAAGGAGTCCAAGCAGCAAAGGAAAGGAGAATTTTTA   7967
APR16-68        AACCCAAGGAGCTAAAGGAGCAGCAAGGAGTCCAAGCAGCAAAGGAAAGGAGAATTTTTA   5324
APR18-62        AACCCAAGGAGCTAAAGGAGCAGCAAGGAGTCCAAGCAGCAAAGGAAAGGAGAATTTTTA   5366
APR19-43        AACCCAAGGAGCTAAAGGAGCAGCAAGGAGTCCAAGCAGCAAAGGAAAGGAGAATTTTTA   5491
APR20-70        AACCCAAGGAGCTAAAGGAGCAGCAAGGAGTCCAAGCAGCAAAGGAAAGGAGAATTTTTA   5211
DEC10-249       AACCCAAGGAGCTAAAGGAGCAGCAAGGAGTCCAAGCAGCAAAGGAAAGGAGAATTTTTA   3375
DEC08-241       AACCCAAGGAGCTAAAGGAGCAGCAAGGAGTCCAAGCAGCAAAGGAAAGGAGAATTTTTA   3664
DEC06-81        AACCCAAGGAGCTAAAGGAGCAGCAAGGAGTCCAAGCAGCAAAGGAAAGGAGAATTTTTA   5376
DEC04-13db      AACCCAAGGAGCTAAAGGAGCAGCAAGGAGTCCAAGCAGCAAAGGAAAGGAGAATTTTTA   12900
MAY17-11        AACCCAAGGAGCTAAAGGAGCAGCAAGGAGTCCAAGCAGCAAAGGAAAGGAGAATTTTTA   7554
MAY15-78        AACCCAAGGAGCTAAAGGAGCAGCAAGGAGTCCAAGCAGCAAAGGAAAGGAGAATTTTTA   5132
JUL05-102       AACCCAAGGAGCTAAAGGAGCAGCAAGGAGTCCAAGCAGCAAAGGAAAGGAGAATTTTTA   5121
JUL04-107       AACCCAAGGAGCTAAAGGAGCAGCAAGGAGTCCAAGCAGCAAAGGAAAGGAGAATTTTTA   5132
JUL03-55        AACCCAAGGAGCTAAAGGAGCAGCAAGGAGTCCAAGCAGCAAAGGAAAGGAGAATTTTTA   5230
JUL02-106       AACCCAAGGAGCTAAAGGAGCAGCAAGGAGTCCAAGCAGCAAAGGAAAGGAGAATTTTTA   5121
JUL01-85        AACCCAAGGAGCTAAAGGAGCAGCAAGGAGTCCAAGCAGCAAAGGAAAGGAGAATTTTTA   5335
DEC03-186       AACCCAAGGAGCTAAAGGAGCAGCAAGGAGTCCAAGCAGCAAAGGAAAGGAGAATTTTTA   3579
DEC07-98        AACCCAAGGAGCTAAAGGAGCAGCAAGGAGTCCAAGCAGCAAAGGAAAGGAGAATTTTTA   4098
APR17-291       AACCCAAGGAGCTAAAGGAGCAGCAAGGAGTCCAAGCAGCAAAGGAAAGGAGAATTTTTA   2960


DEC02-76        ------------------------------------------------------------   6091
CLR03-38395     AGAGGATCAAAGATAAACAGGAGAATTTTTATCCTGTAGTAAGTTCTTTCGAATTTTTCA   5356
CLR01-43699     AGAGGATCAAAGATAAACAGGAGAATTTTTATCCTGTAGTAAGTTCTTTCGAATTTTTCA   8027
APR16-68        AGAGGATCAAAGATAAACAGGAGAATTTTTATCCTGTAGTAAGTTCTTTCGAATTTTTCA   5384
APR18-62        AGAGGATCAAAGATAAACAGGAGAATTTTTATCCTGTAGTAAGTTCTTTCGAATTTTTCA   5426
APR19-43        AGAGGATCAAAGATAAACAGGAGAATTTTTATCCTGTAGTAAGTTCTTTCGAATTTTTCA   5551
APR20-70        AGAGGATCAAAGATAAACAGGAGAATTTTTATCCTGTAGTAAGTTCTTTCGAATTTTTCA   5271
DEC10-249       AGAGGATCAAAGATAAACAGGAGAATTTTTATCCTGTAGTAAGTTCTTTCGAATTTTTCA   3435
DEC08-241       AGAGGATCAAAGATAAACAGGAGAATTTTTATCCTGTAGTAAGTTCTTTCGAATTTTTCA   3724
DEC06-81        AGAGGATCAAAGATAAACAGGAGAATTTTTATCCTGTAGTAAGTTCTTTCGAATTTTTCA   5436
DEC04-13db      AGAGGATCAAAGATAAACAGGAGAATTTTTATCCTGTAGTAAGTTCTTTCGAATTTTTCA   12960
MAY17-11        AGAGGATCAAAGATAAACAGGAGAATTTTTATCCTGTAGTAAGTTCTTTCGAATTTTTCA   7614
MAY15-78        AGAGGATCAAAGATAAACAGGAGAATTTTTATCCTGTAGTAAGTTCTTTCGAATTTTTCA   5192
JUL05-102       AGAGGATCAAAGATAAACAGGAGAATTTTTATCCTGTAGTAAGTTCTTTCGAATTTTTCA   5181
JUL04-107       AGAGGATCAAAGATAAACAGGAGAATTTTTATCCTGTAGTAAGTTCTTTCGAATTTTTCA   5192
JUL03-55        AGAGGATCAAAGATAAACAGGAGAATTTTTATCCTGTAGTAAGTTCTTTCGAATTTTTCA   5290
JUL02-106       AGAGGATCAAAGATAAACAGGAGAATTTTTATCCTGTAGTAAGTTCTTTCGAATTTTTCA   5181
JUL01-85        AGAGGATCAAAGATAAACAGGAGAATTTTTATCCTGTAGTAAGTTCTTTCGAATTTTTCA   5395
DEC03-186       AGAGGATCAAAGATAAACAGGAGAATTTTTATCCTGTAGTAAGTTCTTTCGAATTTTTCA   3639
DEC07-98        AGAGGATCAAAGATAAACAGGAGAATTTTTATCCTGTAGTAAGTTCTTTCGAATTTTTCA   4158
APR17-291       AGAGGATCAAAGATAAACAGGAGAATTTTTATCCTGTAGTAAGTTCTTTCGAATTTTTCA   3020


DEC02-76        ------------------------------------------------------------   6091
CLR03-38395     ACAGTTTCATCCTGAAATTTTTGTAAATGATTTCTTAATCTTATTAGCTGTTCAATGCTT   5416
CLR01-43699     ACAGTTTCATCCTGAAATTTTTGTAAATGATTTCTTAATCTTATTAGCTGTTCAATGCTT   8087
APR16-68        ACAGTTTCATCCTGAAATTTTTGTAAATGATTTCTTAATCTTATTAGCTGTTCAATGCTT   5444
```

```
APR18-62        ACAGTTTCATCCTGAAATTTTTGTAAATGATTTCTTAATCTTATTAGCTGTTCAATGCTT   5486
APR19-43        ACAGTTTCATCCTGAAATTTTTGTAAATGATTTCTTAATCTTATTAGCTGTTCAATGCTT   5611
APR20-70        ACAGTTTCATCCTGAAATTTTTGTAAATGATTTCTTAATCTTATTAGCTGTTCAATGCTT   5331
DEC10-249       ACAGTTTCATCCTGAAATTTTTGTAAATGATTTCTTAATCTTATTAGCTGTTCAATGCTT   3495
DEC08-241       ACAGTTTCATCCTGAAATTTTTGTAAATGATTTCTTAATCTTATTAGCTGTTCAATGCTT   3784
DEC06-81        ACAGTTTCATCCTGAAATTTTTGTAAATGATTTCTTAATCTTATTAGCTGTTCAATGCTT   5496
DEC04-13db      ACAGTTTCATCCTGAAATTTTTGTAAATGATTTCTTAATCTTATTAGCTGTTCAATGCTT   13020
MAY17-11        ACAGTTTCATCCTGAAATTTTTGTAAATGATTTCTTAATCTTATTAGCTGTTCAATGCTT   7674
MAY15-78        ACAGTTTCATCCTGAAATTTTTGTAAATGATTTCTTAATCTTATTAGCTGTTCAATGCTT   5252
JUL05-102       ACAGTTTCATCCTGAAATTTTTGTAAATGATTTCTTAATCTTATTAGCTGTTCAATGCTT   5241
JUL04-107       ACAGTTTCATCCTGAAATTTTTGTAAATGATTTCTTAATCTTATTAGCTGTTCAATGCTT   5252
JUL03-55        ACAGTTTCATCCTGAAATTTTTGTAAATGATTTCTTAATCTTATTAGCTGTTCAATGCTT   5350
JUL02-106       ACAGTTTCATCCTGAAATTTTTGTAAATGATTTCTTAATCTTATTAGCTGTTCAATGCTT   5241
JUL01-85        ACAGTTTCATCCTGAAATTTTTGTAAATGATTTCTTAATCTTATTAGCTGTTCAATGCTT   5455
DEC03-186       ACAGTTTCATCCTGAAATTTTTGTAAATGATTTCTTAATCTTATTAGCTGTTCAATGCTT   3699
DEC07-98        ACAGTTTCATCCTGAAATTTTTGTAAATGATTTCTTAATCTTATTAGCTGTTCAATGCTT   4218
APR17-291       ACAGTTTCATCCTGAAATTTTTGTAAATGATTTCTTAATCTTATTAGCTGTTCAATGCTT   3080


DEC02-76        ------------------------------------------------------------   6091
CLR03-38395     TCTTCAGGTAAATTATTAGGATTATATCTTGAATAGTTTCCTTTTTGTACTAGTTCTGGA   5476
CLR01-43699     TCTTCAGGTAAATTATTAGGATTATATCTTGAATAGTTTCCTTTTTGTACTAGTTCTGGA   8147
APR16-68        TCTTCAGGTAAATTATTAGGATTATATCTTGAATAGTTTCCTTTTTGTACTAGTTCTGGA   5504
APR18-62        TCTTCAGGTAAATTATTAGGATTATATCTTGAATAGTTTCCTTTTTGTACTAGTTCTGGA   5546
APR19-43        TCTTCAGGTAAATTATTAGGATTATATCTTGAATAGTTTCCTTTTTGTACTAGTTCTGGA   5671
APR20-70        TCTTCAGGTAAATTATTAGGATTATATCTTGAATAGTTTCCTTTTTGTACTAGTTCTGGA   5391
DEC10-249       TCTTCAGGTAAATTATTAGGATTATATCTTGAATAGTTTCCTTTTTGTACTAGTTCTGGA   3555
DEC08-241       TCTTCAGGTAAATTATTAGGATTATATCTTGAATAGTTTCCTTTTTGTACTAGTTCTGGA   3844
DEC06-81        TCTTCAGGTAAATTATTAGGATTATATCTTGAATAGTTTCCTTTTTGTACTAGTTCTGGA   5556
DEC04-13db      TCTTCAGGTAAATTATTAGGATTATATCTTGAATAGTTTCCTTTTTGTACTAGTTCTGGA   13080
MAY17-11        TCTTCAGGTAAATTATTAGGATTATATCTTGAATAGTTTCCTTTTTGTACTAGTTCTGGA   7734
MAY15-78        TCTTCAGGTAAATTATTAGGATTATATCTTGAATAGTTTCCTTTTTGTACTAGTTCTGGA   5312
JUL05-102       TCTTCAGGTAAATTATTAGGATTATATCTTGAATAGTTTCCTTTTTGTACTAGTTCTGGA   5301
JUL04-107       TCTTCAGGTAAATTATTAGGATTATATCTTGAATAGTTTCCTTTTTGTACTAGTTCTGGA   5312
JUL03-55        TCTTCAGGTAAATTATTAGGATTATATCTTGAATAGTTTCCTTTTTGTACTAGTTCTGGA   5410
JUL02-106       TCTTCAGGTAAATTATTAGGATTATATCTTGAATAGTTTCCTTTTTGTACTAGTTCTGGA   5301
JUL01-85        TCTTCAGGTAAATTATTAGGATTATATCTTGAATAGTTTCCTTTTTGTACTAGTTCTGGA   5515
DEC03-186       TCTTCAGGTAAATTATTAGGATTATATCTTGAATAGTTTCCTTTTTGTACTAGTTCTGGA   3759
DEC07-98        TCTTCAGGTAAATTATTAGGATTATATCTTGAATAGTTTCCTTTTTGTACTAGTTCTGGA   4278
APR17-291       TCTTCAGGTAAATTATTAGGATTATATCTTGAATAGTTTCCTTTTTGTACTAGTTCTGGA   3140


DEC02-76        ------------------------------------------------------------   6091
CLR03-38395     AATGAACTGTACTCCTGTATGTCTTGTATTGTCTGAGTATTTACTTGTTTTTCTTTTTGT   5536
CLR01-43699     AATGAACTGTACTCCTGTATGTCTTGTATTGTCTGAGTATTTACTTGTTTTTCTTTTTGT   8207
APR16-68        AATGAACTGTACTCCTGTATGTCTTGTATTGTCTGAGTATTTACTTGTTTTTCTTTTTGT   5564
APR18-62        AATGAACTGTACTCCTGTATGTCTTGTATTGTCTGAGTATTTACTTGTTTTTCTTTTTGT   5606
APR19-43        AATGAACTGTACTCCTGTATGTCTTGTATTGTCTGAGTATTTACTTGTTTTTCTTTTTGT   5731
APR20-70        AATGAACTGTACTCCTGTATGTCTTGTATTGTCTGAGTATTTACTTGTTTTTCTTTTTGT   5451
DEC10-249       AATGAACTGTACTCCTGTATGTCTTGTATTGTCTGAGTATTTACTTGTTTTTCTTTTTGT   3615
DEC08-241       AATGAACTGTACTCCTGTATGTCTTGTATTGTCTGAGTATTTACTTGTTTTTCTTTTTGT   3904
DEC06-81        AATGAACTGTACTCCTGTATGTCTTGTATTGTCTGAGTATTTACTTGTTTTTCTTTTTGT   5616
DEC04-13db      AATGAACTGTACTCCTGTATGTCTTGTATTGTCTGAGTATTTACTTGTTTTTCTTTTTGT   13140
MAY17-11        AATGAACTGTACTCCTGTATGTCTTGTATTGTCTGAGTATTTACTTGTTTTTCTTTTTGT   7794
MAY15-78        AATGAACTGTACTCCTGTATGTCTTGTATTGTCTGAGTATTTACTTGTTTTTCTTTTTGT   5372
JUL05-102       AATGAACTGTACTCCTGTATGTCTTGTATTGTCTGAGTATTTACTTGTTTTTCTTTTTGT   5361
JUL04-107       AATGAACTGTACTCCTGTATGTCTTGTATTGTCTGAGTATTTACTTGTTTTTCTTTTTGT   5372
JUL03-55        AATGAACTGTACTCCTGTATGTCTTGTATTGTCTGAGTATTTACTTGTTTTTCTTTTTGT   5470
JUL02-106       AATGAACTGTACTCCTGTATGTCTTGTATTGTCTGAGTATTTACTTGTTTTTCTTTTTGT   5361
JUL01-85        AATGAACTGTACTCCTGTATGTCTTGTATTGTCTGAGTATTTACTTGTTTTTCTTTTTGT   5575
DEC03-186       AATGAACTGTACTCCTGTATGTCTTGTATTGTCTGAGTATTTACTTGTTTTTCTTTTTGT   3819
DEC07-98        AATGAACTGTACTCCTGTATGTCTTGTATTGTCTGAGTATTTACTTGTTTTTCTTTTTGT   4338
APR17-291       AATGAACTGTACTCCTGTATGTCTTGTATTGTCTGAGTATTTACTTGTTTTTCTTTTTGT   3200
```

Figure S4

```
DEC02-76        ------------------------------------------------------------  6091
CLR03-38395     TTATAAGGTCCTCTTTTATAAGCAACTTGTTTTTGTTTTCCTTTATTATTCATTTTAACT  5596
CLR01-43699     TTATAAGGTCCTCTTTTATAAGCAACTTGTTTTTGTTTTCCTTTATTATTCATTTTAACT  8267
APR16-68        TTATAAGGTCCTCTTTTATAAGCAACTTGTTTTTGTTTTCCTTTATTATTCATTTTAACT  5624
APR18-62        TTATAAGGTCCTCTTTTATAAGCAACTTGTTTTTGTTTTCCTTTATTATTCATTTTAACT  5666
APR19-43        TTATAAGGTCCTCTTTTATAAGCAACTTGTTTTTGTTTTCCTTTATTATTCATTTTAACT  5791
APR20-70        TTATAAGGTCCTCTTTTATAAGCAACTTGTTTTTGTTTTCCTTTATTATTCATTTTAACT  5511
DEC10-249       TTATAAGGTCCTCTTTTATAAGCAACTTGTTTTTGTTTTCCTTTATTATTCATTTTAACT  3675
DEC08-241       TTATAAGGTCCTCTTTTATAAGCAACTTGTTTTTGTTTTCCTTTATTATTCATTTTAACT  3964
DEC06-81        TTATAAGGTCCTCTTTTATAAGCAACTTGTTTTTGTTTTCCTTTATTATTCATTTTAACT  5676
DEC04-13db      TTATAAGGTCCTCTTTTATAAGCAACTTGTTTTTGTTTTCCTTTATTATTCATTTTAACT  13200
MAY17-11        TTATAAGGTCCTCTTTTATAAGCAACTTGTTTTTGTTTTCCTTTATTATTCATTTTAACT  7854
MAY15-78        TTATAAGGTCCTCTTTTATAAGCAACTTGTTTTTGTTTTCCTTTATTATTCATTTTAACT  5432
JUL05-102       TTATAAGGTCCTCTTTTATAAGCAACTTGTTTTTGTTTTCCTTTATTATTCATTTTAACT  5421
JUL04-107       TTATAAGGTCCTCTTTTATAAGCAACTTGTTTTTGTTTTCCTTTATTATTCATTTTAACT  5432
JUL03-55        TTATAAGGTCCTCTTTTATAAGCAACTTGTTTTTGTTTTCCTTTATTATTCATTTTAACT  5530
JUL02-106       TTATAAGGTCCTCTTTTATAAGCAACTTGTTTTTGTTTTCCTTTATTATTCATTTTAACT  5421
JUL01-85        TTATAAGGTCCTCTTTTATAAGCAACTTGTTTTTGTTTTCCTTTATTATTCATTTTAACT  5635
DEC03-186       TTATAAGGTCCTCTTTTATAAGCAACTTGTTTTTGTTTTCCTTTATTATTCATTTTAACT  3879
DEC07-98        TTATAAGGTCCTCTTTTATAAGCAACTTGTTTTTGTTTTCCTTTATTATTCATTTTAACT  4398
APR17-291       TTATAAGGTCCTCTTTTATAAGCAACTTGTTTTTGTTTTCCTTTATTATTCATTTTAACT  3260

DEC02-76        ------------------------------------------------------------  6091
CLR03-38395     GTTTGCATTCGAATGAGGTAGTTGAGCACTATCCATTGAATCAAAACTTCCTGGTGTTCC  5656
CLR01-43699     GTTTGCATTCGAATGAGGTAGTTGAGCACTATCCATTGAATCAAAACTTCCTGGTGTTCC  8327
APR16-68        GTTTGCATTCGAATGAGGTAGTTGAGCACTATCCATTGAATCAAAACTTCCTGGTGTTCC  5684
APR18-62        GTTTGCATTCGAATGAGGTAGTTGAGCACTATCCATTGAATCAAAACTTCCTGGTGTTCC  5726
APR19-43        GTTTGCATTCGAATGAGGTAGTTGAGCACTATCCATTGAATCAAAACTTCCTGGTGTTCC  5851
APR20-70        GTTTGCATTCGAATGAGGTAGTTGAGCACTATCCATTGAATCAAAACTTCCTGGTGTTCC  5571
DEC10-249       GTTTGCATTCGAATGAGGTAGTTGAGCACTATCCATTGAATCAAAACTTCCTGGTGTTCC  3735
DEC08-241       GTTTGCATTCGAATGAGGTAGTTGAGCACTATCCATTGAATCAAAACTTCCTGGTGTTCC  4024
DEC06-81        GTTTGCATTCGAATGAGGTAGTTGAGCACTATCCATTGAATCAAAACTTCCTGGTGTTCC  5736
DEC04-13db      GTTTGCATTCGAATGAGGTAGTTGAGCACTATCCATTGAATCAAAACTTCCTGGTGTTCC  13260
MAY17-11        GTTTGCATTCGAATGAGGTAGTTGAGCACTATCCATTGAATCAAAACTTCCTGGTGTTCC  7914
MAY15-78        GTTTGCATTCGAATGAGGTAGTTGAGCACTATCCATTGAATCAAAACTTCCTGGTGTTCC  5492
JUL05-102       GTTTGCATTCGAATGAGGTAGTTGAGCACTATCCATTGAATCAAAACTTCCTGGTGTTCC  5481
JUL04-107       GTTTGCATTCGAATGAGGTAGTTGAGCACTATCCATTGAATCAAAACTTCCTGGTGTTCC  5492
JUL03-55        GTTTGCATTCGAATGAGGTAGTTGAGCACTATCCATTGAATCAAAACTTCCTGGTGTTCC  5590
JUL02-106       GTTTGCATTCGAATGAGGTAGTTGAGCACTATCCATTGAATCAAAACTTCCTGGTGTTCC  5481
JUL01-85        GTTTGCATTCGAATGAGGTAGTTGAGCACTATCCATTGAATCAAAACTTCCTGGTGTTCC  5695
DEC03-186       GTTTGCATTCGAATGAGGTAGTTGAGCACTATCCATTGAATCAAAACTTCCTGGTGTTCC  3939
DEC07-98        GTTTGCATTCGAATGAGGTAGTTGAGCACTATCCATTGAATCAAAACTTCCTGGTGTTCC  4458
APR17-291       GTTTGCATTCGAATGAGGTAGTTGAGCACTATCCATTGAATCAAAACTTCCTGGTGTTCC  3320

DEC02-76        ------------------------------------------------------------  6091
CLR03-38395     TGGTGTTCCTGGTGGTGAAGGTATTTCTGGGTAGAAATCCTTTCTTAATTCATTTAAATT  5716
CLR01-43699     TGGTGTTCCTGGTGGTGAAGGTATTTCTGGGTAGAAATCCTTTCTTAATTCATTTAAATT  8387
APR16-68        TGGTGTTCCTGGTGGTGAAGGTATTTCTGGGTAGAAATCCTTTCTTAATTCATTTAAATT  5744
APR18-62        TGGTGTTCCTGGTGGTGAAGGTATTTCTGGGTAGAAATCCTTTCTTAATTCATTTAAATT  5786
APR19-43        TGGTGTTCCTGGTGGTGAAGGTATTTCTGGGTAGAAATCCTTTCTTAATTCATTTAAATT  5911
APR20-70        TGGTGTTCCTGGTGGTGAAGGTATTTCTGGGTAGAAATCCTTTCTTAATTCATTTAAATT  5631
DEC10-249       TGGTGTTCCTGGTGGTGAAGGTATTTCTGGGTAGAAATCCTTTCTTAATTCATTTAAATT  3795
DEC08-241       TGGTGTTCCTGGTGGTGAAGGTATTTCTGGGTAGAAATCCTTTCTTAATTCATTTAAATT  4084
DEC06-81        TGGTGTTCCTGGTGGTGAAGGTATTTCTGGGTAGAAATCCTTTCTTAATTCATTTAAATT  5796
DEC04-13db      TGGTGTTCCTGGTGGTGAAGGTATTTCTGGGTAGAAATCCTTTCTTAATTCATTTAAATT  13320
MAY17-11        TGGTGTTCCTGGTGGTGAAGGTATTTCTGGGTAGAAATCCTTTCTTAATTCATTTAAATT  7974
MAY15-78        TGGTGTTCCTGGTGGTGAAGGTATTTCTGGGTAGAAATCCTTTCTTAATTCATTTAAATT  5552
JUL05-102       TGGTGTTCCTGGTGGTGAAGGTATTTCTGGGTAGAAATCCTTTCTTAATTCATTTAAATT  5541
JUL04-107       TGGTGTTCCTGGTGGTGAAGGTATTTCTGGGTAGAAATCCTTTCTTAATTCATTTAAATT  5552
```

Figure S4

```
JUL03-55        TGGTGTTCCTGGTGGTGAAGGTATTTCTGGGTAGAAATCCTTTCTTAATTCATTTAAATT   5650
JUL02-106       TGGTGTTCCTGGTGGTGAAGGTATTTCTGGGTAGAAATCCTTTCTTAATTCATTTAAATT   5541
JUL01-85        TGGTGTTCCTGGTGGTGAAGGTATTTCTGGGTAGAAATCCTTTCTTAATTCATTTAAATT   5755
DEC03-186       TGGTGTTCCTGGTGGTGAAGGTATTTCTGGGTAGAAATCCTTTCTTAATTCATTTAAATT   3999
DEC07-98        TGGTGTTCCTGGTGGTGAAGGTATTTCTGGGTAGAAATCCTTTCTTAATTCATTTAAATT   4518
APR17-291       TGGTGTTCCTGGTGGTGAAGGTATTTCTGGGTAGAAATCCTTTCTTAATTCATTTAAATT   3380

DEC02-76        ------------------------------------------------------------   6091
CLR03-38395     TTCATTTATTAAATTTATTTTATGTTCGAGATTATAAATCTTTATCCTCATCTTTGTTAT   5776
CLR01-43699     TTCATTTATTAAATTTATTTTATGTTCGAGATTATAAATCTTTATCCTCATCTTTGTTAT   8447
APR16-68        TTCATTTATTAAATTTATTTTATGTTCGAGATTATAAATCTTTATCCTCATCTTTGTTAT   5804
APR18-62        TTCATTTATTAAATTTATTTTATGTTCGAGATTATAAATCTTTATCCTCATCTTTGTTAT   5846
APR19-43        TTCATTTATTAAATTTATTTTATGTTCGAGATTATAAATCTTTATCCTCATCTTTGTTAT   5971
APR20-70        TTCATTTATTAAATTTATTTTATGTTCGAGATTATAAATCTTTATCCTCATCTTTGTTAT   5691
DEC10-249       TTCATTTATTAAATTTATTTTATGTTCGAGATTATAAATCTTTATCCTCATCTTTGTTAT   3855
DEC08-241       TTCATTTATTAAATTTATTTTATGTTCGAGATTATAAATCTTTATCCTCATCTTTGTTAT   4144
DEC06-81        TTCATTTATTAAATTTATTTTATGTTCGAGATTATAAATCTTTATCCTCATCTTTGTTAT   5856
DEC04-13db      TTCATTTATTAAATTTATTTTATGTTCGAGATTATAAATCTTTATCCTCATCTTTGTTAT   13380
MAY17-11        TTCATTTATTAAATTTATTTTATGTTCGAGATTATAAATCTTTATCCTCATCTTTGTTAT   8034
MAY15-78        TTCATTTATTAAATTTATTTTATGTTCGAGATTATAAATCTTTATCCTCATCTTTGTTAT   5612
JUL05-102       TTCATTTATTAAATTTATTTTATGTTCGAGATTATAAATCTTTATCCTCATCTTTGTTAT   5601
JUL04-107       TTCATTTATTAAATTTATTTTATGTTCGAGATTATAAATCTTTATCCTCATCTTTGTTAT   5612
JUL03-55        TTCATTTATTAAATTTATTTTATGTTCGAGATTATAAATCTTTATCCTCATCTTTGTTAT   5710
JUL02-106       TTCATTTATTAAATTTATTTTATGTTCGAGATTATAAATCTTTATCCTCATCTTTGTTAT   5601
JUL01-85        TTCATTTATTAAATTTATTTTATGTTCGAGATTATAAATCTTTATCCTCATCTTTGTTAT   5815
DEC03-186       TTCATTTATTAAATTTATTTTATGTTCGAGATTATAAATCTTTATCCTCATCTTTGTTAT   4059
DEC07-98        TTCATTTATTAAATTTATTTTATGTTCGAGATTATAAATCTTTATCCTCATCTTTGTTAT   4578
APR17-291       TTCATTTATTAAATTTATTTTATGTTCGAGATTATAAATCTTTATCCTCATCTTTGTTAT   3440

DEC02-76        ------------------------------------------------------------   6091
CLR03-38395     TAATTCTTGAGTTTGGGTAAACCCTTGCTCAATTTGATTACTTATTTGCATCATTAATCC   5836
CLR01-43699     TAATTCTTGAGTTTGGGTAAACCCTTGCTCAATTTGATTACTTATTTGCATCATTAATCC   8507
APR16-68        TAATTCTTGAGTTTGGGTAAACCCTTGCTCAATTTGATTACTTATTTGCATCATTAATCC   5864
APR18-62        TAATTCTTGAGTTTGGGTAAACCCTTGCTCAATTTGATTACTTATTTGCATCATTAATCC   5906
APR19-43        TAATTCTTGAGTTTGGGTAAACCCTTGCTCAATTTGATTACTTATTTGCATCATTAATCC   6031
APR20-70        TAATTCTTGAGTTTGGGTAAACCCTTGCTCAATTTGATTACTTATTTGCATCATTAATCC   5751
DEC10-249       TAATTCTTGAGTTTGGGTAAACCCTTGCTCAATTTGATTACTTATTTGCATCATTAATCC   3915
DEC08-241       TAATTCTTGAGTTTGGGTAAACCCTTGCTCAATTTGATTACTTATTTGCATCATTAATCC   4204
DEC06-81        TAATTCTTGAGTTTGGGTAAACCCTTGCTCAATTTGATTACTTATTTGCATCATTAATCC   5916
DEC04-13db      TAATTCTTGAGTTTGGGTAAACCCTTGCTCAATTTGATTACTTATTTGCATCATTAATCC   13440
MAY17-11        TAATTCTTGAGTTTGGGTAAACCCTTGCTCAATTTGATTACTTATTTGCATCATTAATCC   8094
MAY15-78        TAATTCTTGAGTTTGGGTAAACCCTTGCTCAATTTGATTACTTATTTGCATCATTAATCC   5672
JUL05-102       TAATTCTTGAGTTTGGGTAAACCCTTGCTCAATTTGATTACTTATTTGCATCATTAATCC   5661
JUL04-107       TAATTCTTGAGTTTGGGTAAACCCTTGCTCAATTTGATTACTTATTTGCATCATTAATCC   5672
JUL03-55        TAATTCTTGAGTTTGGGTAAACCCTTGCTCAATTTGATTACTTATTTGCATCATTAATCC   5770
JUL02-106       TAATTCTTGAGTTTGGGTAAACCCTTGCTCAATTTGATTACTTATTTGCATCATTAATCC   5661
JUL01-85        TAATTCTTGAGTTTGGGTAAACCCTTGCTCAATTTGATTACTTATTTGCATCATTAATCC   5875
DEC03-186       TAATTCTTGAGTTTGGGTAAACCCTTGCTCAATTTGATTACTTATTTGCATCATTAATCC   4119
DEC07-98        TAATTCTTGAGTTTGGGTAAACCCTTGCTCAATTTGATTACTTATTTGCATCATTAATCC   4638
APR17-291       TAATTCTTGAGTTTGGGTAAACCCTTGCTCAATTTGATTACTTATTTGCATCATTAATCC   3500

DEC02-76        ------------------------------------------------------------   6091
CLR03-38395     TGTTGTACTTTTAGTTTCATTTATATTTGCTGAATCATGCAAATATTTCCTCAATTTTTC   5896
CLR01-43699     TGTTGTACTTTTAGTTTCATTTATATTTGCTGAATCATGCAAATATTTCCTCAATTTTTC   8567
APR16-68        TGTTGTACTTTTAGTTTCATTTATATTTGCTGAATCATGCAAATATTTCCTCAATTTTTC   5924
APR18-62        TGTTGTACTTTTAGTTTCATTTATATTTGCTGAATCATGCAAATATTTCCTCAATTTTTC   5966
APR19-43        TGTTGTACTTTTAGTTTCATTTATATTTGCTGAATCATGCAAATATTTCCTCAATTTTTC   6091
APR20-70        TGTTGTACTTTTAGTTTCATTTATATTTGCTGAATCATGCAAATATTTCCTCAATTTTTC   5811
DEC10-249       TGTTGTACTTTTAGTTTCATTTATATTTGCTGAATCATGCAAATATTTCCTCAATTTTTC   3975
DEC08-241       TGTTGTACTTTTAGTTTCATTTATATTTGCTGAATCATGCAAATATTTCCTCAATTTTTC   4264
```

Figure S4

```
DEC06-81        TGTTGTACTTTTAGTTTCATTTATATTTGCTGAATCATGCAAATATTTCCTCAATTTTTC   5976
DEC04-13db      TGTTGTACTTTTAGTTTCATTTATATTTGCTGAATCATGCAAATATTTCCTCAATTTTTC   13500
MAY17-11        TGTTGTACTTTTAGTTTCATTTATATTTGCTGAATCATGCAAATATTTCCTCAATTTTTC   8154
MAY15-78        TGTTGTACTTTTAGTTTCATTTATATTTGCTGAATCATGCAAATATTTCCTCAATTTTTC   5732
JUL05-102       TGTTGTACTTTTAGTTTCATTTATATTTGCTGAATCATGCAAATATTTCCTCAATTTTTC   5721
JUL04-107       TGTTGTACTTTTAGTTTCATTTATATTTGCTGAATCATGCAAATATTTCCTCAATTTTTC   5732
JUL03-55        TGTTGTACTTTTAGTTTCATTTATATTTGCTGAATCATGCAAATATTTCCTCAATTTTTC   5830
JUL02-106       TGTTGTACTTTTAGTTTCATTTATATTTGCTGAATCATGCAAATATTTCCTCAATTTTTC   5721
JUL01-85        TGTTGTACTTTTAGTTTCATTTATATTTGCTGAATCATGCAAATATTTCCTCAATTTTTC   5935
DEC03-186       TGTTGTACTTTTAGTTTCATTTATATTTGCTGAATCATGCAAATATTTCCTCAATTTTTC   4179
DEC07-98        TGTTGTACTTTTAGTTTCATTTATATTTGCTGAATCATGCAAATATTTCCTCAATTTTTC   4698
APR17-291       TGTTGTACTTTTAGTTTCATTTATATTTGCTGAATCATGCAAATATTTCCTCAATTTTTC   3560


DEC02-76        ------------------------------------------------------------   6091
CLR03-38395     CATTTTAAATGTGTGGGTAGTAGAGTGGGTCGAAGAACCTCTACTTCCTGTAGGTTGATC   5956
CLR01-43699     CATTTTAAATGTGTGGGTAGTAGAGTGGGTCGAAGAACCTCTACTTCCTGTAGGTTGATC   8627
APR16-68        CATTTTAAATGTGTGGGTAGTAGAGTGGGTCGAAGAACCTCTACTTCCTGTAGGTTGATC   5984
APR18-62        CATTTTAAATGTGTGGGTAGTAGAGTGGGTCGAAGAACCTCTACTTCCTGTAGGTTGATC   6026
APR19-43        CATTTTAAATGTGTGGGTAGTAGAGTGGGTCGAAGAACCTCTACTTCCTGTAGGTTGATC   6151
APR20-70        CATTTTAAATGTGTGGGTAGTAGAGTGGGTCGAAGAACCTCTACTTCCTGTAGGTTGATC   5871
DEC10-249       CATTTTAAATGTGTGGGTAGTAGAGTGGGTCGAAGAACCTCTACTTCCTGTAGGTTGATC   4035
DEC08-241       CATTTTAAATGTGTGGGTAGTAGAGTGGGTCGAAGAACCTCTACTTCCTGTAGGTTGATC   4324
DEC06-81        CATTTTAAATGTGTGGGTAGTAGAGTGGGTCGAAGAACCTCTACTTCCTGTAGGTTGATC   6036
DEC04-13db      CATTTTAAATGTGTGGGTAGTAGAGTGGGTCGAAGAACCTCTACTTCCTGTAGGTTGATC   13560
MAY17-11        CATTTTAAATGTGTGGGTAGTAGAGTGGGTCGAAGAACCTCTACTTCCTGTAGGTTGATC   8214
MAY15-78        CATTTTAAATGTGTGGGTAGTAGAGTGGGTCGAAGAACCTCTACTTCCTGTAGGTTGATC   5792
JUL05-102       CATTTTAAATGTGTGGGTAGTAGAGTGGGTCGAAGAACCTCTACTTCCTGTAGGTTGATC   5781
JUL04-107       CATTTTAAATGTGTGGGTAGTAGAGTGGGTCGAAGAACCTCTACTTCCTGTAGGTTGATC   5792
JUL03-55        CATTTTAAATGTGTGGGTAGTAGAGTGGGTCGAAGAACCTCTACTTCCTGTAGGTTGATC   5890
JUL02-106       CATTTTAAATGTGTGGGTAGTAGAGTGGGTCGAAGAACCTCTACTTCCTGTAGGTTGATC   5781
JUL01-85        CATTTTAAATGTGTGGGTAGTAGAGTGGGTCGAAGAACCTCTACTTCCTGTAGGTTGATC   5995
DEC03-186       CATTTTAAATGTGTGGGTAGTAGAGTGGGTCGAAGAACCTCTACTTCCTGTAGGTTGATC   4239
DEC07-98        CATTTTAAATGTGTGGGTAGTAGAGTGGGTCGAAGAACCTCTACTTCCTGTAGGTTGATC   4758
APR17-291       CATTTTAAATGTGTGGGTAGTAGAGTGGGTCGAAGAACCTCTACTTCCTGTAGGTTGATC   3620


DEC02-76        ------------------------------------------------------------   6091
CLR03-38395     AGACATTTCGGTCTGGATCGAGATAGTTTATCATAGAGATAGTACGGCACTGCCGTTCCT   6016
CLR01-43699     AGACATTTCGGTCTGGATCGAGATAGTTTATCATAGAGATAGTACGGCACTGCCGTTCCT   8687
APR16-68        AGACATTTCGGTCTGGATCGAGATAGTTTATCATAGAGATAGTACGGCACTGCCGTTCCT   6044
APR18-62        AGACATTTCGGTCTGGATCGAGATAGTTTATCATAGAGATAGTACGGCACTGCCGTTCCT   6086
APR19-43        AGACATTTCGGTCTGGATCGAGATAGTTTATCATAGAGATAGTACGGCACTGCCGTTCCT   6211
APR20-70        AGACATTTCGGTCTGGATCGAGATAGTTTATCATAGAGATAGTACGGCACTGCCGTTCCT   5931
DEC10-249       AGACATTTCGGTCTGGATCGAGATAGTTTATCATAGAGATAGTACGGCACTGCCGTTCCT   4095
DEC08-241       AGACATTTCGGTCTGGATCGAGATAGTTTATCATAGAGATAGTACGGCACTGCCGTTCCT   4384
DEC06-81        AGACATTTCGGTCTGGATCGAGATAGTTTATCATAGAGATAGTACGGCACTGCCGTTCCT   6096
DEC04-13db      AGACATTTCGGTCTGGATCGAGATAGTTTATCATAGAGATAGTACGGCACTGCCGTTCCT   13620
MAY17-11        AGACATTTCGGTCTGGATCGAGATAGTTTATCATAGAGATAGTACGGCACTGCCGTTCCT   8274
MAY15-78        AGACATTTCGGTCTGGATCGAGATAGTTTATCATAGAGATAGTACGGCACTGCCGTTCCT   5852
JUL05-102       AGACATTTCGGTCTGGATCGAGATAGTTTATCATAGAGATAGTACGGCACTGCCGTTCCT   5841
JUL04-107       AGACATTTCGGTCTGGATCGAGATAGTTTATCATAGAGATAGTACGGCACTGCCGTTCCT   5852
JUL03-55        AGACATTTCGGTCTGGATCGAGATAGTTTATCATAGAGATAGTACGGCACTGCCGTTCCT   5950
JUL02-106       AGACATTTCGGTCTGGATCGAGATAGTTTATCATAGAGATAGTACGGCACTGCCGTTCCT   5841
JUL01-85        AGACATTTCGGTCTGGATCGAGATAGTTTATCATAGAGATAGTACGGCACTGCCGTTCCT   6055
DEC03-186       AGACATTTCGGTCTGGATCGAGATAGTTTATCATAGAGATAGTACGGCACTGCCGTTCCT   4299
DEC07-98        AGACATTTCGGTCTGGATCGAGATAGTTTATCATAGAGATAGTACGGCACTGCCGTTCCT   4818
APR17-291       AGACATTTCGGTCTGGATCGAGATAGTTTATCATAGAGATAGTACGGCACTGCCGTTCCT   3680


DEC02-76        ------------------------------------------------------------   6091
CLR03-38395     CAGATAAATATTTCAGATTACTCTGAATCCTCAGATATGACTCCTGTATAAAATTTAAGT   6076
CLR01-43699     CAGATAAATATTTCAGATTACTCTGAATCCTCAGATATGACTCCTGTATAAAATTTAAGT   8747
```

Figure S4

```
APR16-68        CAGATAAATATTTCAGATTACTCTGAATCCTCAGATATGACTCCTGTATAAAATTTAAGT   6104
APR18-62        CAGATAAATATTTCAGATTACTCTGAATCCTCAGATATGACTCCTGTATAAAATTTAAGT   6146
APR19-43        CAGATAAATATTTCAGATTACTCTGAATCCTCAGATATGACTCCTGTATAAAATTTAAGT   6271
APR20-70        CAGATAAATATTTCAGATTACTCTGAATCCTCAGATATGACTCCTGTATAAAATTTAAGT   5991
DEC10-249       CAGATAAATATTTCAGATTACTCTGAATCCTCAGATATGACTCCTGTATAAAATTTAAGT   4155
DEC08-241       CAGATAAATATTTCAGATTACTCTGAATCCTCAGATATGACTCCTGTATAAAATTTAAGT   4444
DEC06-81        CAGATAAATATTTCAGATTACTCTGAATCCTCAGATATGACTCCTGTATAAAATTTAAGT   6156
DEC04-13db      CAGATAAATATTTCAGATTACTCTGAATCCTCAGATATGACTCCTGTATAAAATTTAAGT   13680
MAY17-11        CAGATAAATATTTCAGATTACTCTGAATCCTCAGATATGACTCCTGTATAAAATTTAAGT   8334
MAY15-78        CAGATAAATATTTCAGATTACTCTGAATCCTCAGATATGACTCCTGTATAAAATTTAAGT   5912
JUL05-102       CAGATAAATATTTCAGATTACTCTGAATCCTCAGATATGACTCCTGTATAAAATTTAAGT   5901
JUL04-107       CAGATAAATATTTCAGATTACTCTGAATCCTCAGATATGACTCCTGTATAAAATTTAAGT   5912
JUL03-55        CAGATAAATATTTCAGATTACTCTGAATCCTCAGATATGACTCCTGTATAAAATTTAAGT   6010
JUL02-106       CAGATAAATATTTCAGATTACTCTGAATCCTCAGATATGACTCCTGTATAAAATTTAAGT   5901
JUL01-85        CAGATAAATATTTCAGATTACTCTGAATCCTCAGATATGACTCCTGTATAAAATTTAAGT   6115
DEC03-186       CAGATAAATATTTCAGATTACTCTGAATCCTCAGATATGACTCCTGTATAAAATTTAAGT   4359
DEC07-98        CAGATAAATATTTCAGATTACTCTGAATCCTCAGATATGACTCCTGTATAAAATTTAAGT   4878
APR17-291       CAGATAAATATTTCAGATTACTCTGAATCCTCAGATATGACTCCTGTATAAAATTTAAGT   3740


DEC02-76        ------------------------------------------------------------   6091
CLR03-38395     TTTTTATACCTTCCATCGTTCTTCTGGATTTGTTTGGTTGGAATGATTCTCCTGTTTCTG   6136
CLR01-43699     TTTTTATACCTTCCATCGTTCTTCTGGATTTGTTTGGTTGGAATGATTCTCCTGTTTCTG   8807
APR16-68        TTTTTATACCTTCCATCGTTCTTCTGGATTTGTTTGGTTGGAATGATTCTCCTGTTTCTG   6164
APR18-62        TTTTTATACCTTCCATCGTTCTTCTGGATTTGTTTGGTTGGAATGATTCTCCTGTTTCTG   6206
APR19-43        TTTTTATACCTTCCATCGTTCTTCTGGATTTGTTTGGTTGGAATGATTCTCCTGTTTCTG   6331
APR20-70        TTTTTATACCTTCCATCGTTCTTCTGGATTTGTTTGGTTGGAATGATTCTCCTGTTTCTG   6051
DEC10-249       TTTTTATACCTTCCATCGTTCTTCTGGATTTGTTTGGTTGGAATGATTCTCCTGTTTCTG   4215
DEC08-241       TTTTTATACCTTCCATCGTTCTTCTGGATTTGTTTGGTTGGAATGATTCTCCTGTTTCTG   4504
DEC06-81        TTTTTATACCTTCCATCGTTCTTCTGGATTTGTTTGGTTGGAATGATTCTCCTGTTTCTG   6216
DEC04-13db      TTTTTATACCTTCCATCGTTCTTCTGGATTTGTTTGGTTGGAATGATTCTCCTGTTTCTG   13740
MAY17-11        TTTTTATACCTTCCATCGTTCTTCTGGATTTGTTTGGTTGGAATGATTCTCCTGTTTCTG   8394
MAY15-78        TTTTTATACCTTCCATCGTTCTTCTGGATTTGTTTGGTTGGAATGATTCTCCTGTTTCTG   5972
JUL05-102       TTTTTATACCTTCCATCGTTCTTCTGGATTTGTTTGGTTGGAATGATTCTCCTGTTTCTG   5961
JUL04-107       TTTTTATACCTTCCATCGTTCTTCTGGATTTGTTTGGTTGGAATGATTCTCCTGTTTCTG   5972
JUL03-55        TTTTTATACCTTCCATCGTTCTTCTGGATTTGTTTGGTTGGAATGATTCTCCTGTTTCTG   6070
JUL02-106       TTTTTATACCTTCCATCGTTCTTCTGGATTTGTTTGGTTGGAATGATTCTCCTGTTTCTG   5961
JUL01-85        TTTTTATACCTTCCATCGTTCTTCTGGATTTGTTTGGTTGGAATGATTCTCCTGTTTCTG   6175
DEC03-186       TTTTTATACCTTCCATCGTTCTTCTGGATTTGTTTGGTTGGAATGATTCTCCTGTTTCTG   4419
DEC07-98        TTTTTATACCTTCCATCGTTCTTCTGGATTTGTTTGGTTGGAATGATTCTCCTGTTTCTG   4938
APR17-291       TTTTTATACCTTCCATCGTTCTTCTGGATTTGTTTGGTTGGAATGATTCTCCTGTTTCTG   3800


DEC02-76        ------------------------------------------------------------   6091
CLR03-38395     CTGATTTTCTCTTTTCAAGCGGATGGCTACTGGAATTTACTGCCGCCTCTCGGGTCTTAC   6196
CLR01-43699     CTGATTTTCTCTTTTCAAGCGGATGGCTACTGGAATTTACTGCCGCCTCTCGGGTCTTAC   8867
APR16-68        CT----------------------------------------------------------   6166
APR18-62        CT----------------------------------------------------------   6208
APR19-43        CT----------------------------------------------------------   6333
APR20-70        CT----------------------------------------------------------   6053
DEC10-249       CT----------------------------------------------------------   4217
DEC08-241       CT----------------------------------------------------------   4506
DEC06-81        CT----------------------------------------------------------   6218
DEC04-13db      CT----------------------------------------------------------   13742
MAY17-11        CTGATTTTCTCTTTTCAAGCGGATGGCTACTGGAATTTACTGCCGCCTCTCGGGTCTTAC   8454
MAY15-78        CTGATTTTCTCTTTTCAAGCGGATGGCTACTGGAATTTACTGCCGCCTCTCGGGTCTTAC   6032
JUL05-102       CT----------------------------------------------------------   5963
JUL04-107       CT----------------------------------------------------------   5974
JUL03-55        CT----------------------------------------------------------   6072
JUL02-106       CT----------------------------------------------------------   5963
JUL01-85        CT----------------------------------------------------------   6177
DEC03-186       CT----------------------------------------------------------   4421
DEC07-98        CT----------------------------------------------------------   4940
```

Figure S4

```
APR17-291        CT----------------------------------------------------------   3802


DEC02-76         ------------------------------------------------------------   6091
CLR03-38395      CACCAAGCCGTATCAACGCCTCCAGAAAAGATCTCTGAAAATTTTCAGAAACTCTTTGAA   6256
CLR01-43699      CACCAAGCCGTATCAACGCCTCCAGAAAAGATCTCTGAAAATTTTCAGAAACTCTTTGAA   8927
APR16-68         ------------------------------------------------------------   6166
APR18-62         ------------------------------------------------------------   6208
APR19-43         ------------------------------------------------------------   6333
APR20-70         ------------------------------------------------------------   6053
DEC10-249        ------------------------------------------------------------   4217
DEC08-241        ------------------------------------------------------------   4506
DEC06-81         ------------------------------------------------------------   6218
DEC04-13db       ------------------------------------------------------------   13742
MAY17-11         CACCAAGCCGTATCAACGCCTCCAGAAAAGATCTCTGAAAATTTTCAGAAACTCTTTGAA   8514
MAY15-78         CACCAAGCCGTATCAACGCCTCCAGAAAAGATCTCTGAAAATTTTCAGAAACTCTTTGAA   6092
JUL05-102        ------------------------------------------------------------   5963
JUL04-107        ------------------------------------------------------------   5974
JUL03-55         ------------------------------------------------------------   6072
JUL02-106        ------------------------------------------------------------   5963
JUL01-85         ------------------------------------------------------------   6177
DEC03-186        ------------------------------------------------------------   4421
DEC07-98         ------------------------------------------------------------   4940
APR17-291        ------------------------------------------------------------   3802


DEC02-76         ------------------------------------------------------------   6091
CLR03-38395      TCTCCTAAATCCTAGATTTTGGCTATAAGCCTCCTGAGAATAGTTTTACTGGAAATTCTC   6316
CLR01-43699      TCTCCTAAATCCTAGATTTTGGCTATAAGCCTCCTGAGAATAGTTTTACTGGAAATTCTC   8987
APR16-68         ------------------------------------------------------------   6166
APR18-62         ------------------------------------------------------------   6208
APR19-43         ------------------------------------------------------------   6333
APR20-70         ------------------------------------------------------------   6053
DEC10-249        ------------------------------------------------------------   4217
DEC08-241        ------------------------------------------------------------   4506
DEC06-81         ------------------------------------------------------------   6218
DEC04-13db       ------------------------------------------------------------   13742
MAY17-11         TCTCCTAAATCCTAGATTTTGGCTATAAGCCTCCTGAGAATAGTTTTACTGGAAATTCTC   8574
MAY15-78         TCTCCTAAATCCTAGATTTTGGCTATAAGCCTCCTGAGAATAGTTTTACTGGAAATTCTC   6152
JUL05-102        ------------------------------------------------------------   5963
JUL04-107        ------------------------------------------------------------   5974
JUL03-55         ------------------------------------------------------------   6072
JUL02-106        ------------------------------------------------------------   5963
JUL01-85         ------------------------------------------------------------   6177
DEC03-186        ------------------------------------------------------------   4421
DEC07-98         ------------------------------------------------------------   4940
APR17-291        ------------------------------------------------------------   3802


DEC02-76         ------------------------------------------------------------   6091
CLR03-38395      CGTGAGGGAGATTTTTTATTGATTTCTTGAAATGCTTACAAAGCTCCTAAAACATATGCT   6376
CLR01-43699      CGTGAGGGAGATTTTTTATTGATTTCTTGAAATGCTTACAAAGCTCCTAAAACATATGCT   9047
APR16-68         ------------------------------------------------------------   6166
APR18-62         ------------------------------------------------------------   6208
APR19-43         ------------------------------------------------------------   6333
APR20-70         ------------------------------------------------------------   6053
DEC10-249        ------------------------------------------------------------   4217
DEC08-241        ------------------------------------------------------------   4506
DEC06-81         ------------------------------------------------------------   6218
DEC04-13db       ------------------------------------------------------------   13742
MAY17-11         CGTGAGGGAGATTTTTTATTGATTTCTTGAAATGCTTACAAAGCTCCTAAAACATATGCT   8634
MAY15-78         CGTGAGGGAGATTTTTTATTGATTTCTTGAAATGCTTACAAAGCTCCTAAAACATATGCT   6212
JUL05-102        ------------------------------------------------------------   5963
```

Figure S4

```
JUL04-107       ------------------------------------------------------------   5974
JUL03-55        ------------------------------------------------------------   6072
JUL02-106       ------------------------------------------------------------   5963
JUL01-85        ------------------------------------------------------------   6177
DEC03-186       ------------------------------------------------------------   4421
DEC07-98        ------------------------------------------------------------   4940
APR17-291       ------------------------------------------------------------   3802


DEC02-76        ---------------------  6091
CLR03-38395     CTCACTTTTCCTCTCTCA---  6394
CLR01-43699     CTCACTTTTCCTCTCTCATTT  9068
APR16-68        ---------------------  6166
APR18-62        ---------------------  6208
APR19-43        ---------------------  6333
APR20-70        ---------------------  6053
DEC10-249       ---------------------  4217
DEC08-241       ---------------------  4506
DEC06-81        ---------------------  6218
DEC04-13db      ---------------------  13742
MAY17-11        CTCACTTTTCCTCTCTCATT-  8654
MAY15-78        CTCACTTTTCCTCTCTCA---  6230
JUL05-102       ---------------------  5963
JUL04-107       ---------------------  5974
JUL03-55        ---------------------  6072
JUL02-106       ---------------------  5963
JUL01-85        ---------------------  6177
DEC03-186       ---------------------  4421
DEC07-98        ---------------------  4940
APR17-291       ---------------------  3802
```
